# Supplementary material for: Multi-Fused S,N-Heterocyclic Compounds for Targeting α-Synuclein Aggregates
Source: Cells. 2025 Sep 30;14(19):1531. doi: 10.3390/cells14191531 (PMC12523303; doi:10.3390/cells14191531)

Supplementary Materials

for

Multi-Fused S,N-Heterocyclic Compounds for Targeting α-Synuclein Aggregates

Chao Zheng,[1] Jeffrey S. Stehouwer,[2] Goverdhan Reddy Ummenthala,[3] Yogeshkumar S. Munot[3] and Neil Vasdev[1,*]

[1] MedChem Imaging, Inc. Boston, MA, 02210, USA.
[2] Department of Radiology, University of Pittsburgh, Pittsburgh, PA 15213, USA.
[3] Laxai Life Sciences, Hyderabad, Telangana, 500078, India.
* Correspondence: neil@medchemimaging.com

Contents

1 Synthesis of LMD-001 to LMD-070 2
2 *In vitro* assays 75
2.1 Preliminary 2-point screening assay 75
2.2 K_i Determination 77
2.3 Summary of Structural Variants and Their 2-Point Screening and Ki Profiles 77
3 Chromatographic Characteristics of LMD compounds 82
3.1 Chromatographic Analysis of Metabolic Stability of LMD Compounds in Human Liver Microsomes 82
3.2 Chromatographic Analysis of LMD and Control Compounds in the MDR1-MDCK Permeability Assay 86
4 Characterization by ^{1}H NMR spectroscopy 87

1 Synthesis of LMD-001 to LMD-070

Scheme:

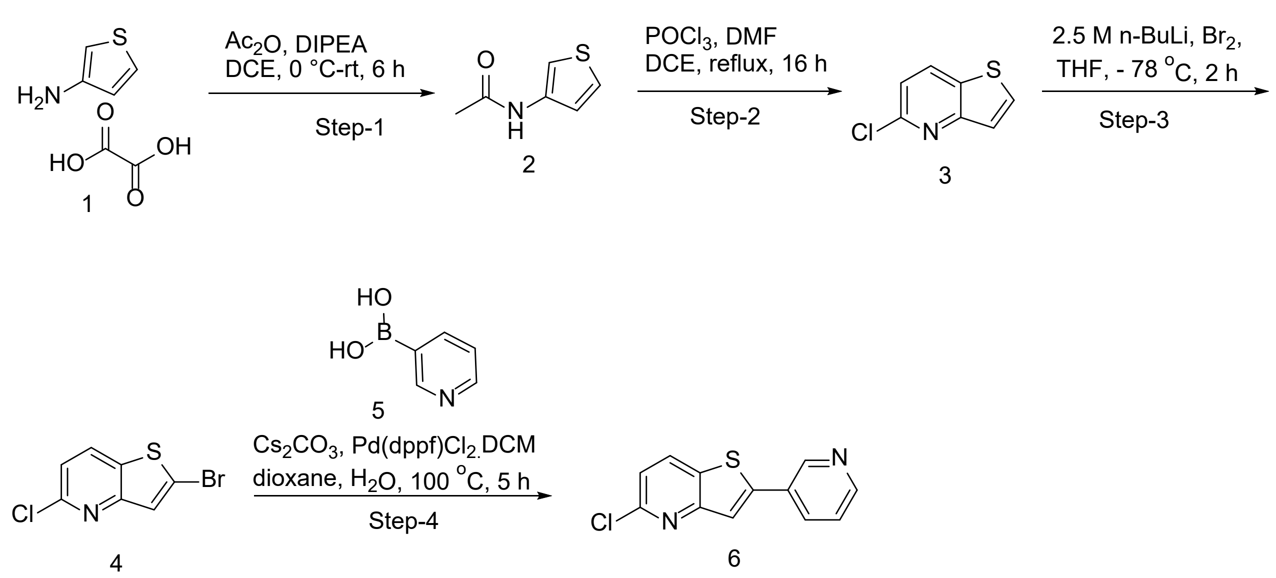

Step-1: Synthesis of *N*-(thiophen-3-yl) acetamide (2):

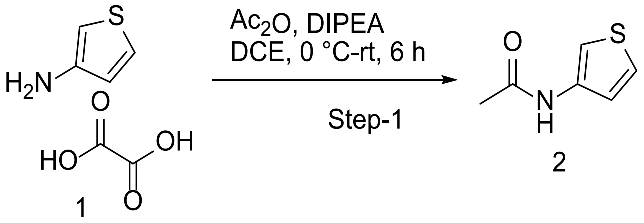

To a stirred solution of thiophen-3-amine oxalate (70.0 g, 0.37 mol) in DCM (350 mL) was added *N*-ethyldiisopropylamine (200 mL, 3.0 eq, 1.11 mol) at 0 ºC and stirred for 30 min. After that, acetic anhydride (70.0 mL, 2.0 eq, 0.74 mol) was added and allowed to stir at room temperature for 2 h. The reaction progress was monitor by TLC. After completion, the reaction mixture was basified with saturated sodium bicarbonate solution and solid was observed. The obtained solid was filtered and washed with water (250 mL), dried under vacuum to obtained *N*-(thiophen-3-yl) acetamide (28.0 g, 53.61%) as an off white solid. ^{1}H NMR ($CDCl_3$, 400 MHz): δ 10.29 (s, 1H), 7.48 (d, J = 2.8 Hz, 1H), 7.42 - 7.40 (m, 1H), 7.02 (d, J = 5.2 Hz, 1H), 2.00 (s, 3H).

Step-2: Synthesis of 5-chlorothieno[3,2-*b*] pyridine (3):

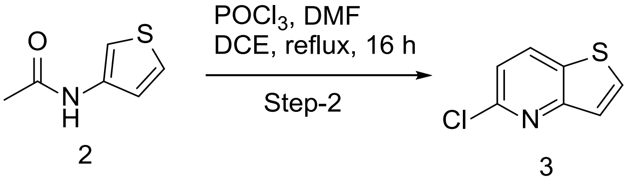

To a 0 °C cooled solution of phosphoryl trichloride (18.5 mL, 198 mmol) in DMF (77 mL) was added *N*-3-thienylacetamide (28.0 g, 198 mmol) in 1,2-dichloroethane (280 mL) dropwise over a period of 30 min. The resulting mixture was heated to 110 °C for 16 h. The progress of the reaction monitored by TLC. After completion, reaction was concentrated under reduced pressure to get crude compound. The crude was cooled to 0 °C and basified with saturated $NaHCO_3$ solution (200 mL) results formation of solid. The obtained solid was filtered and washed with water, dried under vacuum to get crude. The crude was purified by column chromatography using 20% EtOAc/hexane as eluent to afford 5-chlorothieno[3,2-*b*] pyridine (14.0 g, 41.62%) as an off white solid. ^{1}H NMR (DMSO-d_6, 400 MHz): δ 8.57 (d, J = 8.4 Hz, 1H), 8.26 (d, J = 5.2 Hz, 1H), 7.56 (d, J = 5.6 Hz, 1H), 7.47 (d, J = 8.4 Hz, 1H).

Step-3: Synthesis of 2-bromo-5-chlorothieno[3,2-*b*] pyridine (4):

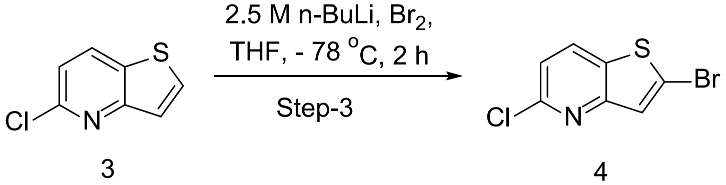

To a -78 °C cooled solution of 5-chlorothieno [3,2-*b*] pyridine (8.0 g, 47.2 mmol) in THF (40 mL) was added 2.5 M solution of *n*-BuLi in hexane (21.22 mL, 1.12 eq, 53.05 mmol) and allowed to stir at - 40 °C for 30 minutes. After that, the reaction was again cooled to -78 °C, followed by addition of Br_2 (2.92 mL, 1.2 eq, 56.6 mmol) dropwise, stirred for 1 h and allowed to stir at room temperature for 3 h. The progress of the reaction was monitor by TLC. After completion, the reaction mixture was quenched with Sat NH_4Cl (50 mL) and separated the organic layer. The aqueous layer was extracted with ethyl acetate (3 x 50 mL). The combined organic layers were dried over Na_2SO_4 and concentrated under reduced pressure to get crude compound. The crude compound was washed with *n*-pentane to afford 2-bromo-5-chlorothieno [3,2-*b*] pyridine (7.5 g, 63.99 %) as a brown solid. This was used in the next step without any further purification.

^{1}H-NMR (DMSO-d_6, 400 MHz): 8.50 (d, J = 8.8 Hz, 1H), 7.79 (s, 1H), 7.48 (d, J = 8.8 Hz, 1H).

Step-4: Synthesis of 5-chloro-2-(pyridin-3-yl) thieno [3,2-*b*] pyridine (6):

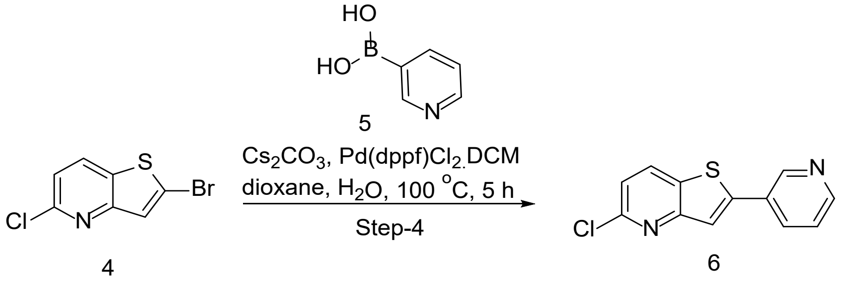

To a stirred solution of 2-bromo-5-chlorothieno [3,2-*b*] pyridine (4.0 g, 16.12 mmol) and 3-(4,4,5,54-tetramethy1,3,2-dioxaborolan-2-yl) pyridine (2.9 g, 0.9 eq, 14.5 mmol) in 1,4-dioxane (36 mL), H_2O (12 mL), was added cesium carbonate (15.57 g, 3.0 eq, 48.3 mmol.) at room temperature and degassed with argon for 20 min. After that, Pd(dppf)Cl_2.DCM (1.31 g, 0.1 eq, 1.61 mmol) was added under argon atmosphere, and heated at 100 ºC for 12 h. The progress of the reaction was monitor by TLC. After completion, the reaction was filtered through celite pad, washed with EtOAc (100 mL). The filtrate was washed with water (2 x 50 mL) and brine (50 mL). The organic layer was dried over Na_2SO_4, and concentrated under reduced pressure to get crude. The residue was purified on column chromatography using 10% EtOAc/n-hexane as eluent to afford 5-chloro-2-(pyridin-3-yl) thieno [3,2-*b*] pyridine (2.4 g, 60.44%) as an off yellow solid.

^{1}H NMR ($CDCl_3$, 400 MHz): δ 8.99 (s, 1H), 8.66 (d, *J* = 4.0 Hz, 1H), 8.10 (d, *J* = 8.4 Hz, 1H), 8.01 - 7.98 (m, 1H), 7.74 (s, 1H), 7.43 - 7.40 (m, 1H), 7.29 (d, *J* = 8.4 Hz, 1H).

LCMS(ESI): m/z = 247.01 $[M+H]^+$

LMD-001 & 002

Scheme:

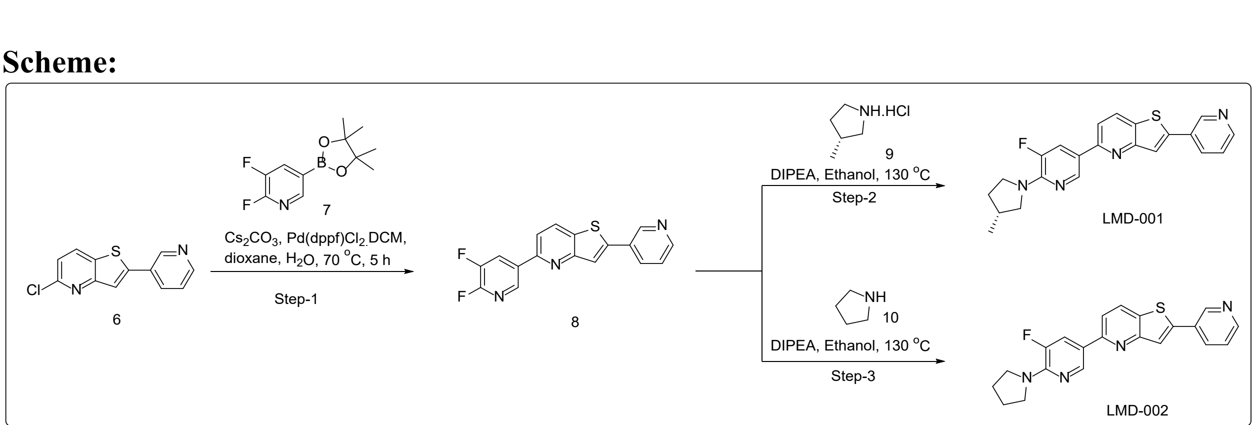

Step-1: Synthesis of 5-(5,6-difluoropyridin-3-yl)-2-(pyridin-3-yl)thieno[3,2-*b*]pyridine (8):

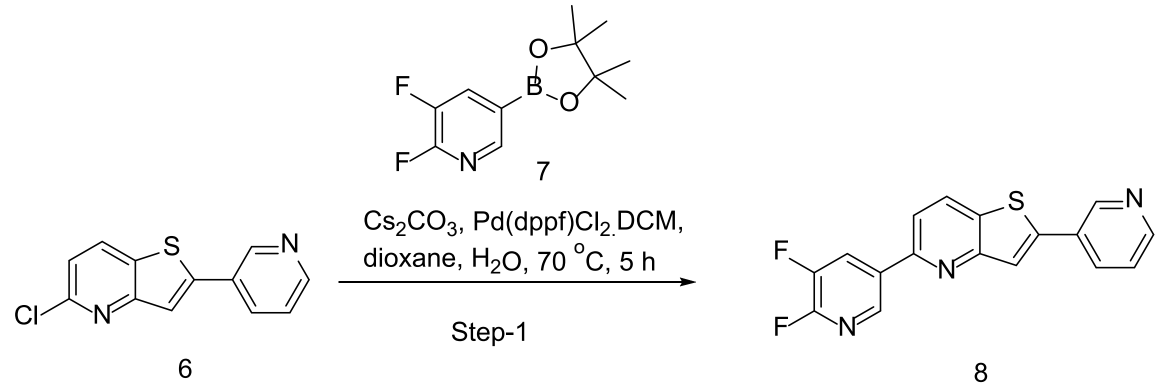

To a solution of 5-chloro-2-(pyridin-3-yl)thieno[3,2-*b*]pyridine (200 mg, 1.0 eq, 811 µmol) and 2,3-difluoro-5-(4,4,5,5-tetramethyl-1,3,2-dioxaborolan-2-yl)pyridine (258 mg, 2.0 eq.,1.62 mmol) in dioxane (3 mL), H_2O (0.6 mL) was added cesium carbonate (792 mg, 3.0 eq, 2.43 mmol) at room temperature and purged with argon gas for 20 min. After that, Pd(dppf)Cl_2.DCM (66 mg, 0.1 eq., 81.1 µmol) was added under argon atmosphere and heated at 100 °C for 5 h. The reaction progress was monitored by TLC. After completion of reaction, the reaction filtered through celite pad and washed with EtOAc (20 mL). The filtrate was washed with water (20 mL) followed by brine solution (20 mL). The organic layer was dried over Na_2SO_4, and concentrated under reduced pressure to get residue. The residue was purified by silica gel column chromatography using EtOAc/*n*-hexane as eluent to afford 5-(5,6-difluoropyridin-3-yl)-2-(pyridin-3-yl)thieno[3,2-*b*]pyridine (200 mg, 75% yield) as an off white solid.

Step-2: Synthesis of (*R*)-5-(5-Fluoro-6-(3-methylpyrrolidin-1-yl)pyridin-3-yl)-2-(pyridin-3-yl)thieno[3,2-*b*]pyridine (LMD-001):

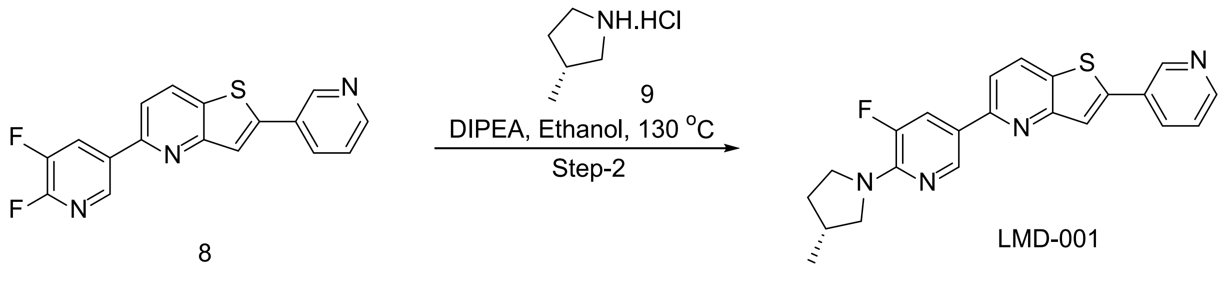

To a stirred solution of 5-(5,6-difluoropyridin-3-yl)-2-(pyridin-3-yl)thieno[3,2-*b*]pyridine (100 mg, 1.0 eq, 307 µmol) in EtOH (4 mL) was added (*R*)-3-methylpyrrolidine—hydrogen chloride

(1/1) (75 mg, 2.0 eq., 615 µmol) and *N*-ethyldiisopropylamine (199 mg, 5.0 eq., 1.54 mmol) at room temperature and stirred at 130 °C for 16 h. The progress of the reaction mixture was monitored by TLC. After completion of the reaction, solvent was removed the under reduced pressure to get crude. The crude was purified by silica gel column chromatography using 15% EtOAc/hexane as eluent to afford (*R*)-5-(5-fluoro-6-(3-methylpyrrolidin-1-yl)pyridin-3-yl)-2-(pyridin-3-yl)thieno[3,2-*b*]pyridine (105 mg, 83% yield) as a white solid. ^{1}H NMR (DMSO-d_6, 400 MHz): δ 9.12 (d, J = 1.6 Hz, 1H), 8.75 (s, 1H), 8.63 (d, J = 4.4 Hz, 1H), 8.51 (d, J = 8.8 Hz, 1H), 8.28 (d, J = 8.0 Hz, 1H), 8.19 (s, 1H), 8.12 (dd, J = 14.0, 1.2 Hz, 1H), 7.94 (d, J = 8.4 Hz, 1H), 7.58-7.53 (m, 1H), 3.88-3.80 (m, 1H), 3.80-3.72 (m, 1H), 3.67-3.55 (m, 1H), 3.25-3.15 (m, 1H), 2.40-2.26 (m, 1H), 2.10-2.04 (m, 1H), 1.62-1.49 (m, 1H), 1.09 (d, J = 6.8 Hz, 3H).
^{19}F NMR: -136.9 ppm. LCMS (m/z): 391.1 (M+H). HPLC purity: 97.67%

Step-3: Synthesis of 5-(5-Fluoro-6-(pyrrolidin-1-yl)pyridin-3-yl)-2-(pyridin-3-yl)thieno[3,2-*b*]pyridine (LMD-002):

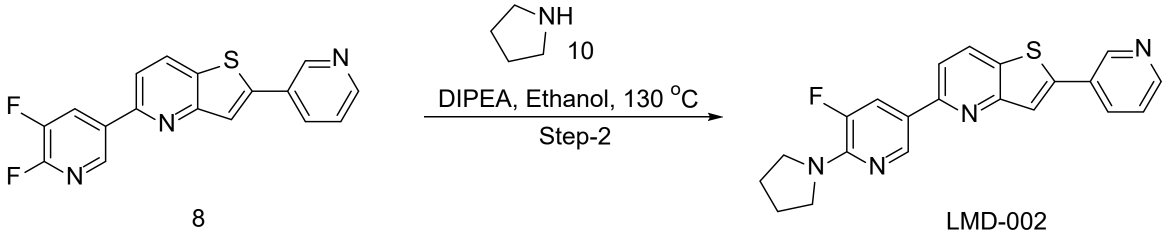

To a stirred solution of 5-(5,6-difluoropyridin-3-yl)-2-(pyridin-3-yl)thieno[3,2-*b*]pyridine (100 mg, 1.0 eq, 307 µmol) in EtOH (10 mL) was added pyrrolidine (44 mg, 2.0 eq., 615 µmol) and *N*-ethyldiisopropylamine (199 mg, 5.0 eq., 1.54 mmol) at room temperature and stirred at 130 °C for 16 h. The progress of the reaction mixture was monitored by TLC. After completion of reaction, solvent was removed the under reduced pressure to get crude. The crude was purified on silica gel column chromatography using 15% EtOAc/hexane to afford (*R*)-5-(5-fluoro-6-(3-methylpyrrolidin-1-yl)pyridin-3-yl)-2-(pyridin-3-yl)thieno[3,2-*b*]pyridine (105 g, 86% yield) as a white solid. ^{1}H NMR (DMSO-d_6, 400 MHz): δ 9.12 (s, 1H), 8.76 (s, 1H), 8.63 (d, J = 4.8 Hz, 1H), 8.51 (d, J = 8.8 Hz, 1H), 8.28 (d, J = 8.0 Hz, 1H), 8.19 (s, 1H), 8.13 (dd, J = 15.2 Hz, 1H), 7.94 (d, J = 8.1 Hz, 1H), 7.58 - 7.51 (m, 1H), 3.64 (d, J = 8.8 Hz, 4H), 1.93 (t, J = 5.6 Hz, 4H).
^{19}F NMR: -136.6 ppm. LCMS (m/z): 377.1 (M+H). HPLC purity: 98.42%

LMD-009

Scheme:

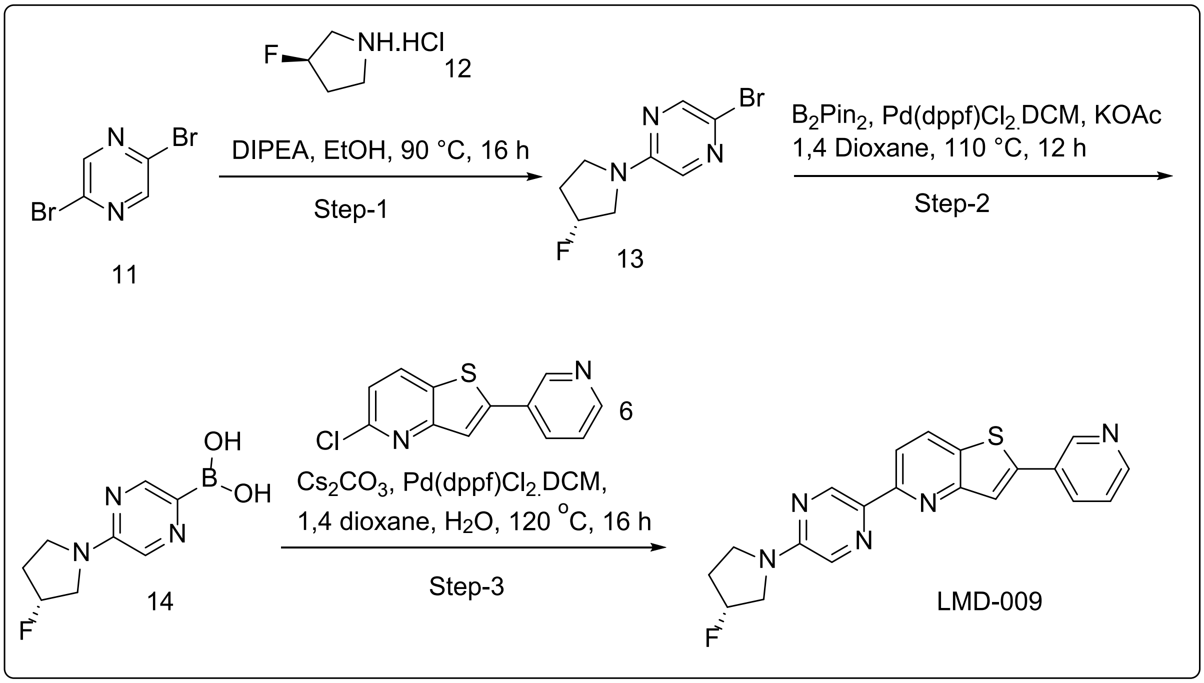

Step-1: Synthesis of (*R*)-2-bromo-5-(3-fluoropyrrolidin-1-yl) pyrazine (13):

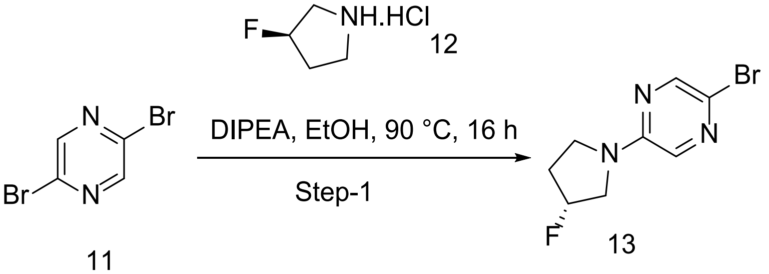

To a stirred solution of 2,5-dibromopyrazine (10.0 g, 1.0 eq, 42.4 mmol) in EtOH (50 mL) was added (*R*)-3-fluoropyrrolidine hydrochloride (1/1) (6.3 g, 1.2 eq, 50.9 mmol) and *N*-ethyldiisopropylamine (14.78 mL, 3.0 eq, 127.2 mmol) at room temperature and stirred at 90 °C for 16 h. The progress of the reaction mixture was monitored by TLC. After completion of the reaction, solvent was removed the under reduced pressure to get crude. The crude was purified by silica gel column chromatography using 15% EtOAc/hexane as eluent to afford (*R*)-2-bromo-5-(3-fluoropyrrolidin-1-yl) pyrazine (7 g, 67.67%) as an off white solid. ^{1}H NMR (DMSO-d_6, 400 MHz): δ 8.12 (d, J = 1.6 Hz, 1H), 7.64 (d, J = 1.2 Hz, 1H), 5.46 - 5.31 (m, 1H), 3.86 - 3.76 (m, 1H), 3.69 - 3.53 (m, 3H), 2.48 - 2.38 (m, 1H), 2.26 - 2.06 (m, 1H). ^{19}F NMR: -176.39 ppm

LCMS (m/z): $[M+H]^+$: 248.08/246.05

Step-2: Synthesis of (*R*)-(5-(3-fluoropyrrolidin-1-yl)pyrazin-2-yl)boronic acid (14):

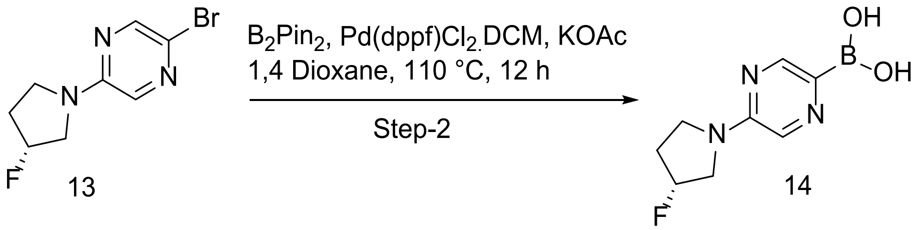

To a solution of (*R*)-2-bromo-5-(3-fluoropyrrolidin-1-yl) pyrazine (7.0 g, 1.0 eq, 28.6 mmol) and B_2Pin_2 (14.5 g, 2.0 eq, 57.14 mmol) in 1,4-dioxane (70.0 mL) was added KOAc (5.6 g, 2.0 eq, 57.14 mmol) at room temperature and purged with argon gas for 20 min. After that, Pd(dppf)Cl_2.DCM (1.16 g, 0.05 eq, 1.43 mmol) was added under argon atmosphere and heated at 110 °C for 12 h. The reaction progress was monitored by TLC. After completion of the reaction, filtered through celite pad and washed with hexane (30 x 3 mL). The filtrate was dried over Na_2SO_4, and concentrated under reduced pressure to afford (*R*)-(5-(3-fluoropyrrolidin-1-yl)pyrazin-2-yl)boronic acid (18 g, crude) as a brown liquid. This was used in the next step without further purification. LCMS (m/z): 211.9 $[M+H]^+$

Step-3: Synthesis of (*R*)-5-(5-(3-fluoropyrrolidin-1-yl)pyrazin-2-yl)-2-(pyridin-3-yl)thieno[3,2-*b*]pyridine (LMD-009):

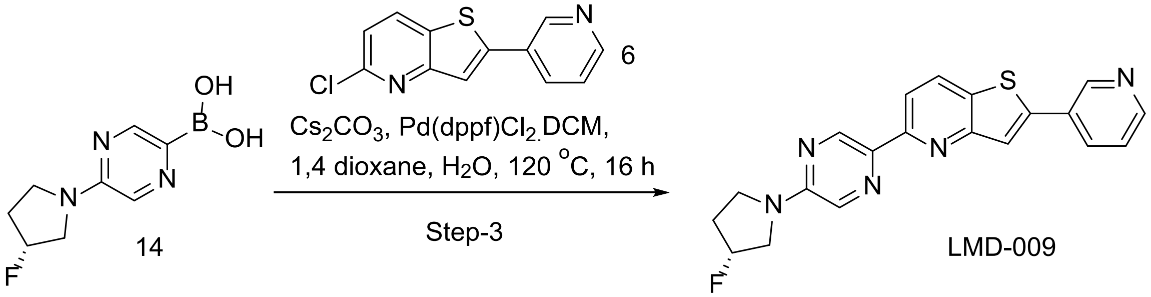

To a solution of 5-chloro-2-(pyridin-3-yl) thieno[3,2-*b*]pyridine (0.60 g, 1.0 eq, 2.44 mmol) and (*R*)-(5-(3-fluoropyrrolidin-1-yl)pyrazin-2-yl)boronic acid (1.03 g, 2.0 eq, 4.9 mmol) in 1,4 dioxane (10.0 mL), H_2O (2.0 mL) was added Cs_2CO_3 (2.37 g, 3.0 eq, 7.31 mmol) at room temperature and purged with argon gas for 20 min. After that, Pd(dppf)Cl_2.DCM (99 mg, 0.05 eq, 0.12 mmol) was added under argon atmosphere and heated at 120 °C for 16 h. The reaction progress was monitored by TLC. After completion of the reaction, the reaction filtered through celite pad and washed with EtOAc (25 mL). The filtrate was washed with water (20 mL) followed by brine solution (20 mL).

The organic layer was dried over Na_2SO_4, and concentrated under reduced pressure to get residue. The residue was purified by silica gel column chromatography using EtOAc/*n*-hexane as eluent, then re-purified by prep-HPLC to afford (*R*)-5-(5-(3-fluoropyrrolidin-1-yl) pyrazin-2-yl)-2-(pyridin-3-yl) thieno[3,2-*b*] pyridine (5.42 mg, 0.59%) as a pale-yellow solid. ^{1}H NMR (DMSO-d_6, 400 MHz): δ 9.12 (d, *J* = 11.6 Hz, 2H), 8.64 (d, *J* = 4.8 Hz, 1H), 8.55 (d, *J* = 8.4 Hz, 1H), 8.29 (d, *J* = 8.0 Hz, 1H), 8.24 (s, 1H), 8.17 (d, *J* = 8.8 Hz, 1H), 8.14 (s, 1H), 7.57 - 7.54 (m, 1H), 5.51 (d, *J* = 52.4 Hz, 1H), 3.93 - 3.77 (m, 3H), 3.70 - 3.53 (m, 2H), 2.30 - 2.28 (s, 1H). ^{19}F NMR: -175.30 ppm LCMS (m/z): 378.3 $[M+H]^+$. HPLC purity: 95.37%

LMD-011

Scheme:

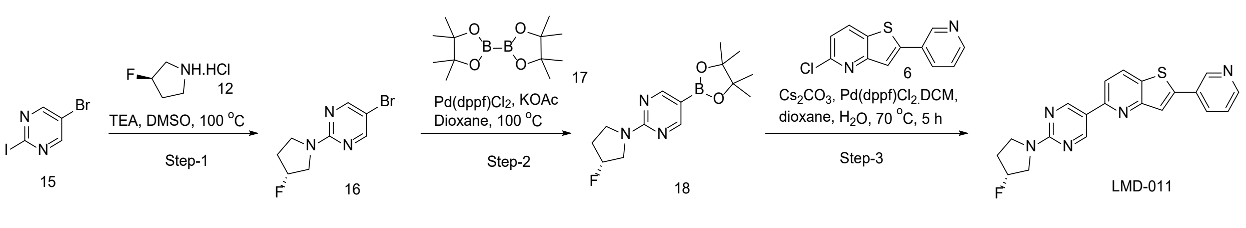

Step-1: Synthesis of 5-bromo-2-[(*R*)-3-fluoro-1-pyrrolidinyl] pyrimidine (16):

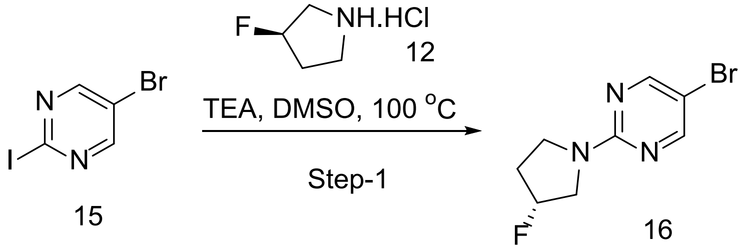

To a stirred solution of 5-bromo-2-iodopyrimidine (2.0 g, 1.0 eq, 7.02 mmol) in DMSO (20 mL) was added 3-fluoropyrrolidine-hydrogen chloride (1/1) (1.76 g, 2.0 eq., 14 mmol) followed by triethylamine (3.55 g, 5.0 eq, 35.1 mmol) at room temperature and stirred at 100 °C for 12 h. The progress of reaction was monitor by TLC. After completion, the reaction was cooled to room temperature, concentrated under reduce pressure to get crude residue. The crude was purified by flash column chromatography using 0-50% EtOAc/hexane as eluent to afford 5-bromo-2-[(*R*)-3-fluoro-1-pyrrolidinyl]pyrimidine (1.5 g, 86.82%) as a white solid. MS (ESI): m/z = 247.0 $[M+H]^+$

Step-2: Synthesis of (*R*)-2-(3-fluoropyrrolidin-1-yl)-5-(4,4,5,5-tetramethyl-1,3,2-dioxaborolan-2-yl) pyrimidine (18):

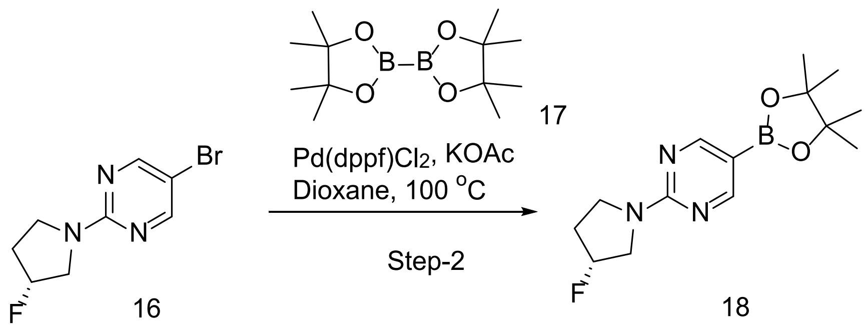

To a stirred solution of 5-bromo-2-[(*R*)-3-fluoro-1-pyrrolidinyl]pyrimidine (1.0 g, 4.06 mmol) in 1,4-dioxane (15 mL) was added bis(pinacolato)diboron (2.06 g, 2.0 eq, 8.13 mmol) and potassium acetate (798 mg, 2.0 eq, 8.13 mmol) at room temperature and purged with argon gas for 20 min. After that, Pd(dppf)Cl_2.DCM (332 mg, 0.1 eq., 406 µmol) was added and the reaction mixture was heated at 100 °C for 16 h. The reaction progress was monitor by TLC. After completion of reaction, the reaction mixture was washed three times with *n*-hexane (30 mL) and concentrated under reduced pressure to obtain the title compound 2-{2-[(*R*)-3-fluoro-1-pyrrolidinyl]-5-pyrimidinyl}-4,4,5,5-tetramethyl-1,3,2-dioxaborolane (1.5 g. 80%) as a brown solid. This was used in the next step without any further purification.

Step-3: Synthesis of (*R*)-5-(2-(3-fluoropyrrolidin-1-yl) pyrimidin-5-yl)-2-(pyridin-3-yl) thieno[3,2-*b*] pyridine (LMD-011):

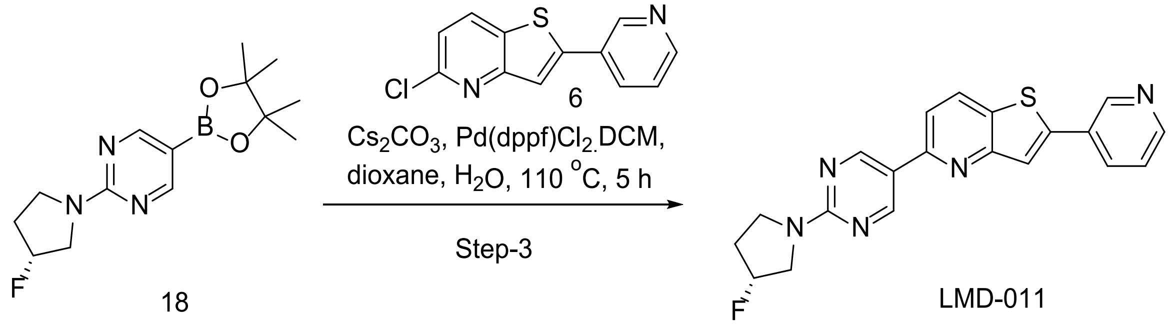

To a stirred solution of 5-chloro-2-(3-pyridyl)-1-thia-4-azaindene (199 mg, 1.0 eq, 805 µmol) and 2-{2-[(*R*)-3-fluoro-1-pyrrolidinyl]-5-pyrimidinyl}-4,4,5,5-tetramethyl-1,3,2-dioxaborolane (708 mg, 3.0 eq, 2.41 mmol) in 1,4-dioxane (9 mL), water (3 mL) was added Cs_2CO_3 (780 mg, 3.0 eq, 2.41 mmol) at room temperature and purged with argon gas for 20 min. After that, Pd(dppf)Cl_2.DCM (33 mg, 0.05 eq, 40.02 µmol) was added under argon atmosphere and heated at 110 °C for 12 h. The reaction progress of was monitored by TLC. After completion of the reaction, filtered through celite pad and washed with EtOAc (20 mL). The filtrate was washed with water

(20 mL) and brine solution (10 mL). The organic layer was dried over Na_2SO_4, and concentrated under reduced pressure to get crude. The residue was purified by silica gel column chromatography using 40% EtOAc/*n*-hexane as eluent to afford the title compound, which was re-purified by using prep-HPLC to afford (*R*)-5-(2-(3-fluoropyrrolidin-1-yl) pyrimidin-5-yl)-2-(pyridin-3-yl) thieno[3,2-*b*] pyridine (4.2 mg, 1.56%) as a light brown solid. ^{1}H NMR (DMSO-d_6, 400 MHz): δ 9.15 - 9.13 (m, 3H), 8.64 (d, *J* = 4.8 Hz, 1H), 8.57 (d, *J* = 8.4 Hz, 1H), 8.30 (d, *J* = 8.0 Hz, 1H), 8.23 (s, 1H), 7.94 (d, *J* = 8.4 Hz, 1H), 7.58 -7.54 (m, 1H), 5.50 (d, *J* = 52.0 Hz, 1H), 4.0 - 3.55 (m, 4H), 2.35 - 2.25 (m, 2H). ^{19}F NMR: -175.64 ppm. MS (ESI): m/z = 378.24 $[M+H]^+$
HPLC purity: 99.30%

LMD-005

Scheme:

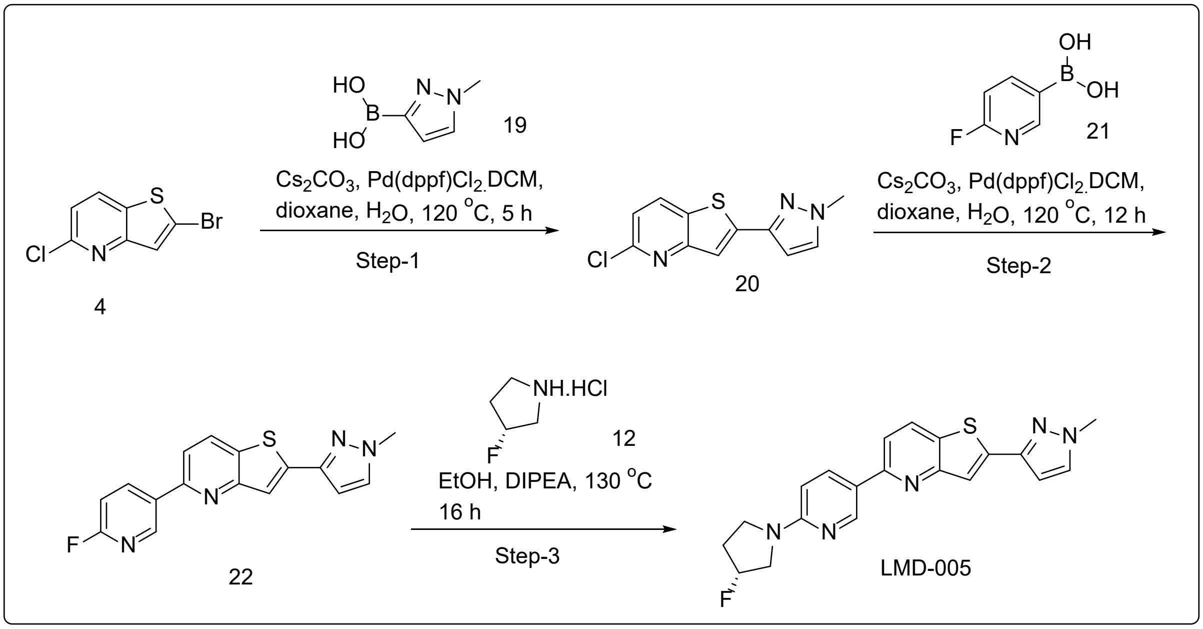

Step-1: Synthesis of 5-chloro-2-(1-methyl-1*H*-pyrazol-3-yl) thieno[3,2-*b*] pyridine (20):

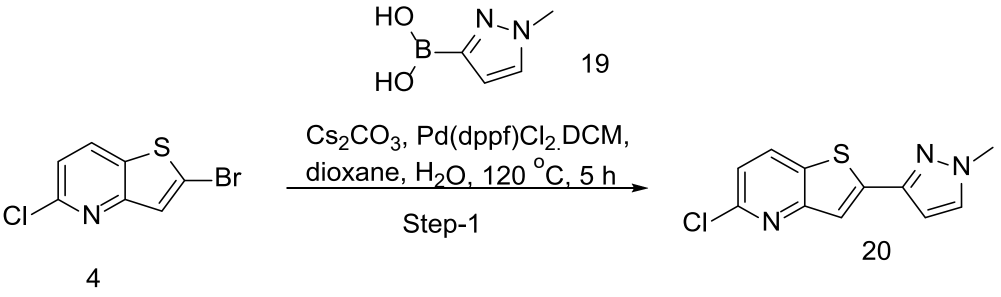

To a stirred solution of 2-brorno-6chlorothieno[3,2-*b*] pyridine (1.0 g, 1.0 eq, 4.1 mmcl) & (1-methyl-1*H*-pyrazol-3-yl) boronic acid (0.46 g, 0.9 eq, 3.7 mmol) in 1,4-dioxane (18 mL) and H_2O (6 mL) was added Cs_2CO_3 (3.9 g, 12.2 mmol) and degassed with argon for 20 min. Then, Pd(dppf)Cl_2.DCM (165 mg, 0.203 mmol) was added under argon and heated at 120 °C for 5 h. The progress of the reaction was monitored b TLC. After that, the reaction mixture was cooled to room temperature, filtered through celite pad and washed with EtOAc (2 x 20 mL). The filtrate was washed with water (2 x 20 mL) and brine (20 mL). The organic layer was dried over Na_2SO_4, and concentrated under reduced pressure to get crude. The crude residue was purified on column chromatography using EtOAc/*n*-hexane as eluent to afford 5-chloro-2-(1-methyl-1*H*-pyrazol-3-yl) thieno[3,2-*b*] pyridine (600 mg, 59.71%) as an off white solid. ^{1}H NMR (DMSO-d_6, 400 MHz): δ 8.04 (d, J = 8.8, Hz, 1H), 7.60 (s,1H), 7.42 (d, J = 2.0 Hz, 1H), 7.21 (d, J = 8.4 Hz, 1H), 6.63 (d, J = 2.0 Hz, 1H) 3.97 (s, 3H). LCMS (ESI): 250.06 m/z $[M+H]^+$

Step-2: Synthesis of 5-(6-fluoropyridin-3-yl)-2-(1-methyl-1*H*-pyrazol-3-yl)thieno[3,2-*b*]pyridine (22):

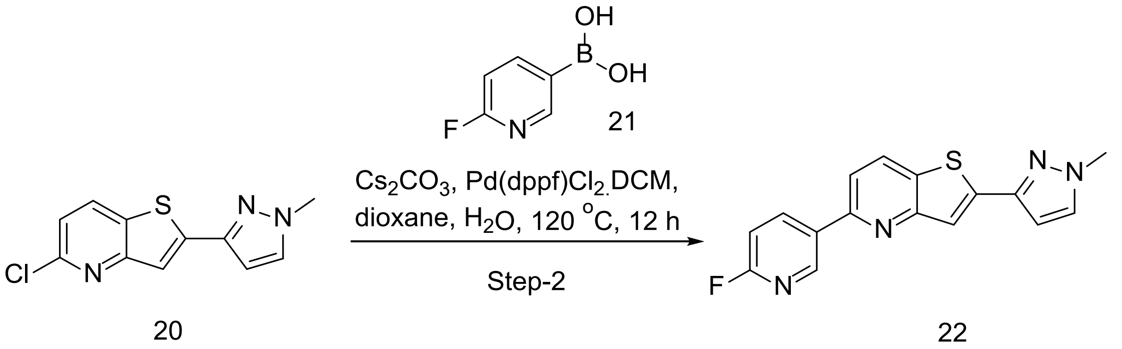

To a stirred solution of 5-chloro-2-(1-methyl-1*H*-pyrazol-3-yl) thieno[3,2-*b*] pyridine (100 mg, 1.0 eq, 0.4 mmol) and (6-fluoropyridin-3-yl) boronic acid (51 mg, 0.9 eq, 0.36 mmol), cesium carbonate (391 mg, 3.0 eq, 1.20 mmol) in 1,4-Dioxane (2 mL) and water (0.3 mL) was degassed with argon for 20 min. Then, Pd(dppf)Cl_2.DCM (16 mg, 0.05 eq, 0.02 mmol) was added under argon and heated at 100 °C for 12 h. The reaction progress was monitored by TLC. Then, the mixture was cooled to room temperature and filtered through celite pad, washed with EtOAc (10 mL). The filtrate was washed with water (2 x 5 mL) and brine (5 mL). The organic layer was dried over Na_2SO_4, and concentrated under reduced pressure to get crude. The crude residue was purified on column chromatography using EtOAc/n-hexane as eluent to afford 5-(6-fluoropyridin-3-yl)-2-

(1-methyl-1*H*-pyrazol-3-yl) thieno [3,2-*b*] pyridine (200 mg, crude). This was used in the next step as a crude.

Step-3: Synthesis of (*R*)-5-(6-(3-fluoropyrrolidin-1-yl) pyridin-3-yl)-2-(1-methyl-1*H*-pyrazol-3-yl) thieno[3,2-*b*] pyridine (LMD-005):

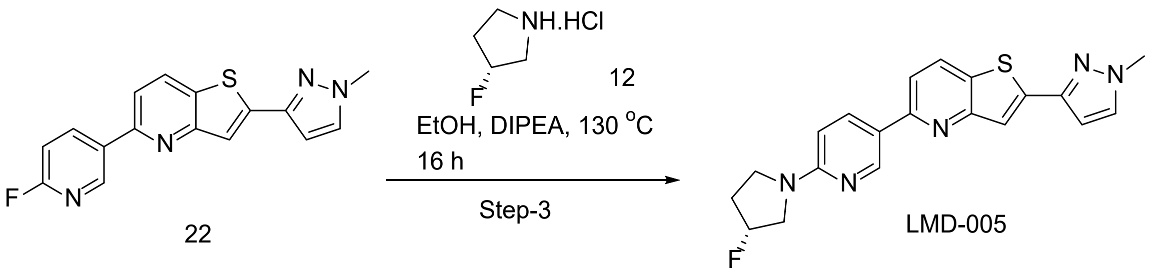

Ta stirred solution of 5-(6-fluoropyridin-3-yl)-2-(1-methyl-1*H*-pyrazol-3-yl) thieno[3,2-*b*]pyridine (200 mg, 1.0 eq, 0.64 mmol), in EtOH (3 mL) in a seal tube was added (*R*)-3-fluoropyrrolidine.HCl (403 mg, 5.0 eq, 3.2 mmol), followed by *N*-diisopropylethylamine (832 mg, 10.0 eq, 6.5 mmol) at room temperature and heated at 130 °C for 16 h. The reaction progress was monitored by TLC. After completion of the reaction, the solvent was removed under reduced pressure to get crude. The crude residue was taken up in DCM (20 mL), washed with ammonium chloride solution (20 mL) and water (20 mL). The organic layer was dried over Na_2SO_4, and concentrated under reduced pressure to get residue. The residue was purified on column chromatography using 20% EtOAc/hexane to afford the (*R*)-5-(6-(3-fluoropyrrolidin-1-yl) pyridin-3-yl)-2-(1-methyl-1*H*-pyrazol-3-yl) thieno [3,2-*b*] pyridine (41 mg, 16.77 %) as an off white solid. ^{1}H NMR (DMSO-d_6, 400 MHz): δ 8.89 (s, 1H), 8.38 (d, *J* = 8.4 Hz, 1H), 8.30 (d, *J* = 8.8 Hz, 1H), 7.86 - 7.80 (m, 3H), 6.91 (s, 1H) 6.64 (d, *J* = 8.8 Hz, 1H), 5.54 (d, *J* = 53 Hz, 1H), 3.91 (s, 3H), 3.85 – 3.60 (m, 3H), 3.52 - 3.45 (m, 1H), 2.31 - 2.15 (m, 2H). ^{19}F NMR: -174.86 ppm MS (ES I); m/z = 380.1 [M+H]$^+$. HPLC purity: 99.3%

LMD-013

Scheme:

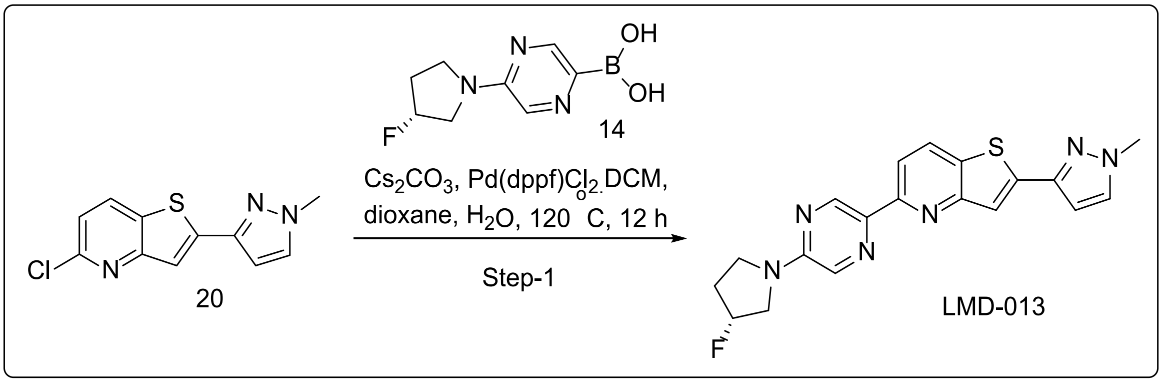

Step-1: Synthesis of (*R*)-5-(5-(3-fluoropyrrolidin-1-yl)pyrazin-2-yl)-2-(1-methyl-1*H*-pyrazol-3-yl)thieno[3,2-*b*]pyridine (LMD-013):

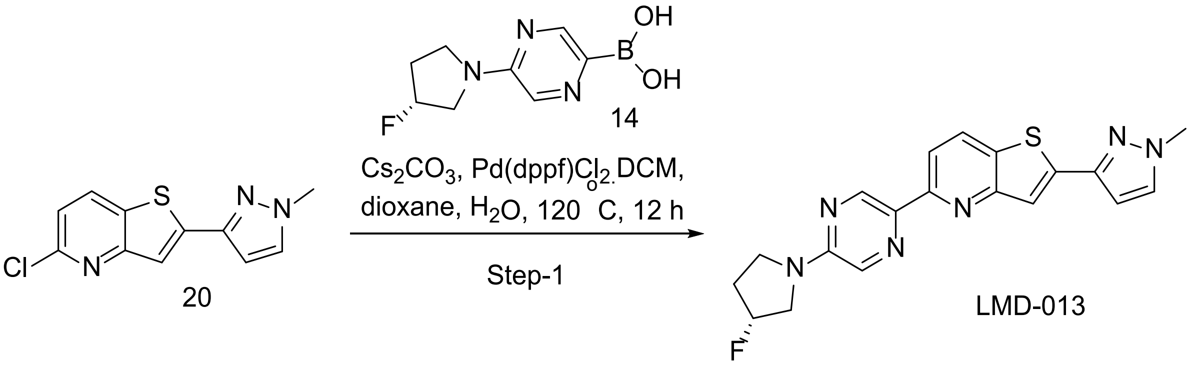

To a stirred solution of 5-chloro-2-(1-methyl-1*H*-pyrazol-3-yl)thieno[3,2-*b*]pyridine (300 mg, 1.0 eq, 1.2 mmol) and (*R*)-(5-(3-fluoropyrrolidin-1-yl)pyrazin-2-yl)boronic acid (508 mg, 2.0 eq, 2.4 mmol), in 1,4-dioxane (5 mL), H_2O (1.2 mL) was added cesium carbonate (1.17 g, 3.0 eq, 3.6 mmol) at room temperature and purged with argon gas for 20 min. After that, Pd(dppf)Cl_2.DCM (49 mg, 0.05 eq, 0.06 mmol) was added under argon atmosphere and heated at 120 °C for 12 h. The reaction progress was monitored by TLC. After completion of reaction, the reaction filtered through celite pad and washed with EtOAc (20 mL). The filtrate was washed with water (20 mL) followed by brine solution (20 mL). The organic layer was dried over Na_2SO_4, and concentrated under reduced pressure to get residue. The residue was purified by silica gel column chromatography using EtOAc/*n*-hexane as eluent to afford title compound, which was re-purified by using prep-HPLC to afford (*R*)-5-(5-(3-fluoropyrrolidin-1-yl)pyrazin-2-yl)-2-(1-methyl-1*H*-pyrazol-3-yl)thieno[3,2-*b*]pyridine (15 mg, 3.28%) as an off white solid.

^{1}H NMR (DMSO-d_6, 400 MHz): δ 9.08 (s,1H), 8.43 (d, J = 8.4 Hz, 1H), 8.12 - 8.09 (m, 2H), 7.90 (s, 1H), 7.83 (s, 1H), 6.93 (s, 1H), 5.57 - 5.44 (d, J = 52 Hz, 1H), 3.91 - 3.69 (m, 6H), 3.57 - 3.55 (m, 1H), 2.32 - 2.28 (m, 1H), 2.19 - 2.16 (m, 1H). ^{19}F NMR: -175.27 ppm

LCMS (ESI): 381.26 m/z [M+H]+. HPLC purity: 98.08%

LMD-060

Scheme:

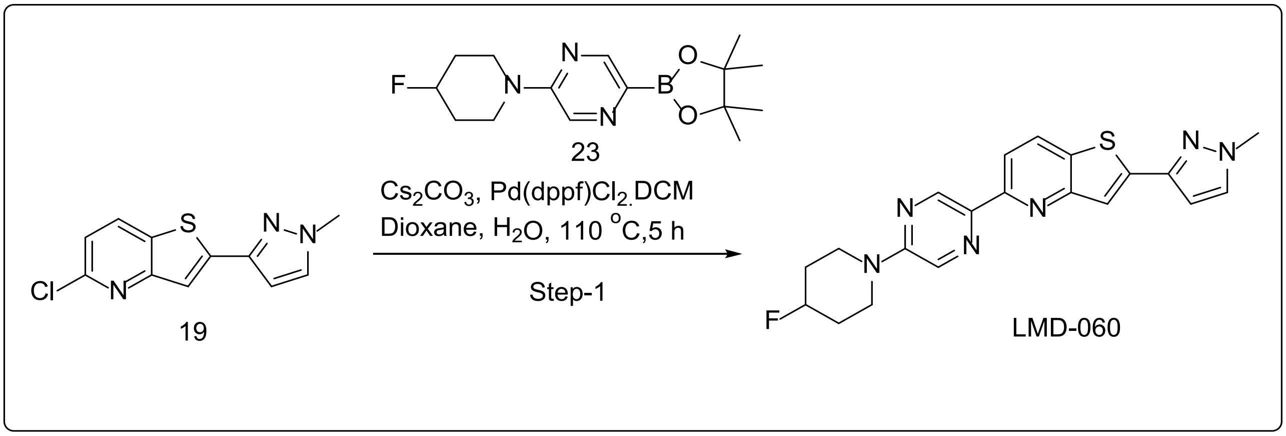

Step-1: Synthesis of 5-(5-(4-fluoropiperidin-1-yl) pyrazin-2-yl)-2-(1-methyl-1*H*-pyrazol-3-yl) thieno[3,2-*b*] pyridine (LMD-060):

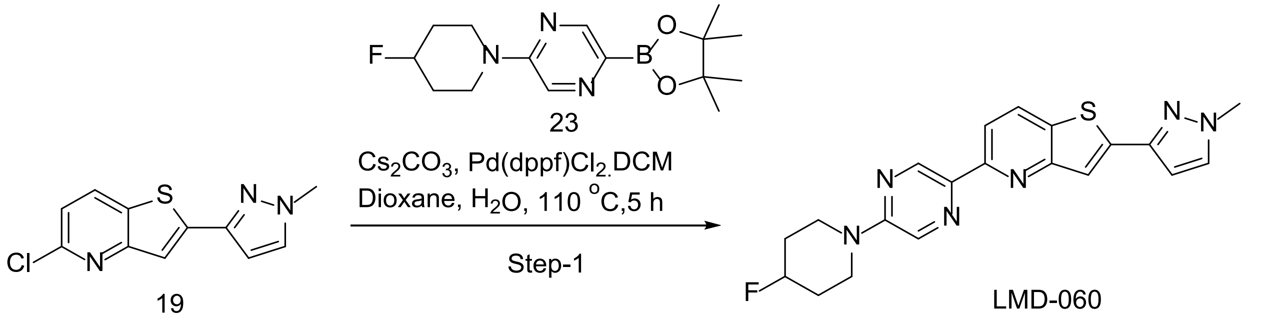

To a stirred solution of 5-chloro-2-(1-methyl-1*H*-pyrazol-3-yl)thieno [3,2-*b*] pyridine (200 mg, 1.0 eq, 0.81 mmol) and 2-(4-fluoropiperidin-1-yl)-5-(4,4,5,5-tetramethyl-1,3,2-dioxaborolan-2-yl)pyrazine (474 mg , 2.0 eq, 1.61 mmol), in Dioxane (3 mL), H_2O (1 mL) was added cesium carbonate (787 mg, 3.0 eq, 2.41 mmol) in a sealed tube at room temperature and degassed with argon gas for 20 min. After that, Pd(dppf)Cl_2.DCM (33.2 mg, 0.05 eq, 0.04 mmol) under argon atmosphere and heated at 100 °C for 12 h. The reaction progress was monitored by TLC. After completion of the reaction, filtered through celite pad and washed with EtOAc (25 mL). The filtrate was washed with water (10 mL) and brine solution (10 mL). The organic layer was dried over

Na_2SO_4, and concentrated under reduced pressure results crude residue. The crude residue was purified by using 50% EtOAc/*n*-heptane as eluent to afford 5-(5-(4-fluoropiperidin-1-yl) pyrazin-2-yl)-2-(1-methyl-1*H*-pyrazol-3-yl) thieno[3,2-*b*] pyridine (9.6 mg, 3.4%) as an off white solid. ^{1}H NMR (DMSO-d_6, 400 MHz): δ 9.07 (s. 1H), 8.47 (s, 1H), 8.44 (d, J = 8.4 Hz, 1H), 8.09 (d, J = 8.4 Hz, 1H), 7.91 (s, 1H), 7.84 (s, 1H), 6.93 (s, 1H), 4.99 (d, J = 54.0 Hz, 1H). 3.91 (s, 3H), 3.89- 3.82 (m, 2H), 3.75 - 3.65 (m, 2H), 2.07 - 1.92 (m, 2H), 1.85 - 1.72 (m, 2H). ^{19}F NMR: -177.54 ppm. LCMS (m/z):395.5 (M+H). HPLC purity: 99.67%

LMD-006

Scheme:

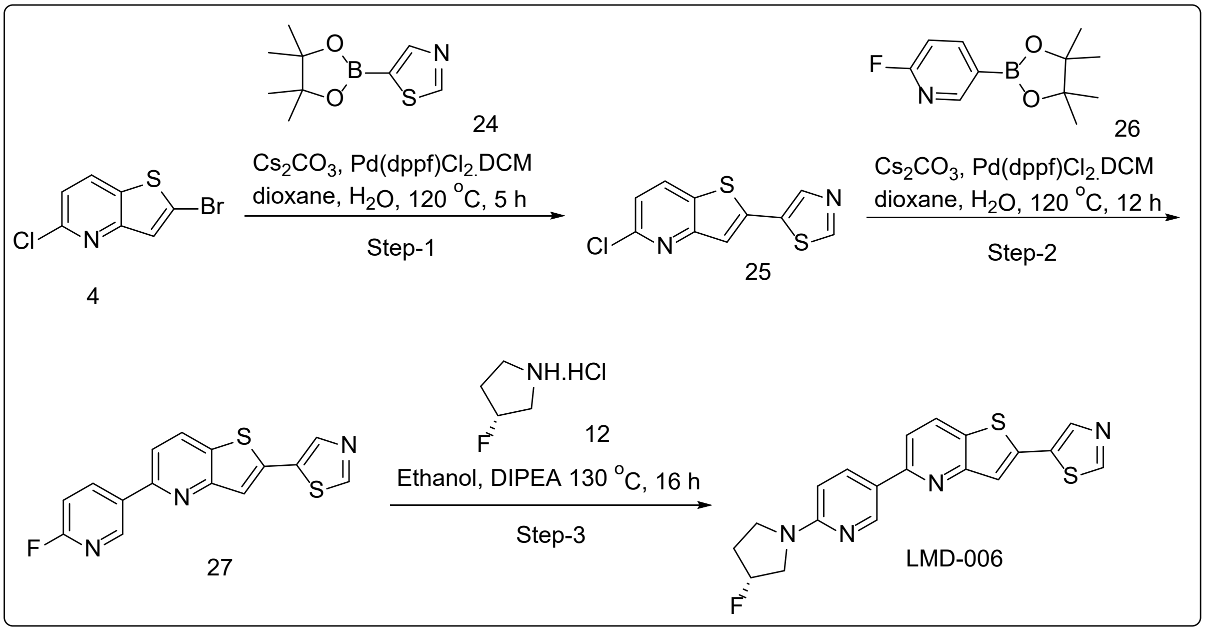

Step-1: Synthesis of 5-chloro-2-(thiazol-5-yl)thieno[3,2-*b*]pyridine (25):

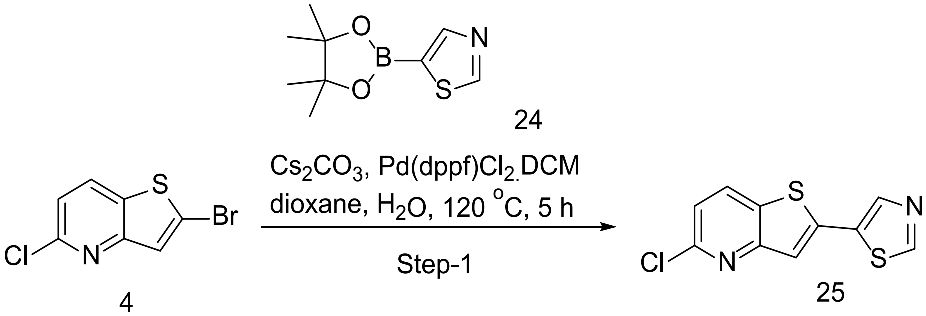

To a solution of 2-bromo-6-chlorothieno[3,2-*b*] pyridine (1.0 g, 1.0 eq, 4.1 mmol) and 5-(4,4,5,5-tetramethyl-1,3,2-dioxaborolan-2-yl) thiazole (767 mg, 0.9 eq, 3.7 mmol) in 1,4-dioxane (9.0 mL), H_2O (2.0 mL) was added Cs_2CO_3 (3.9 g, 3.0 eq, 1.22 mmol) at room temperature and purged with

argon gas for 20 min. After that, Pd(dppf)Cl_2.DCM (16 mg, 0.05 eq, 0.020 mmol) was added under argon atmosphere and heated at 120 °C for 5 h. The reaction progress was monitored by TLC. After completion of reaction, the reaction was filtered through celite pad and washed with EtOAc (20 mL). The filtrate was washed with water (20 mL) followed by brine solution (20 mL). The organic layer was dried over Na_2SO_4, and concentrated under reduced pressure to get residue. The residue was purified by silica gel column chromatography using EtOAc/*n*-hexane as eluent to afford 5-chloro-2-(thiazol-5-yl) thieno[3,2-*b*] pyridine (400 mg, 39.33%) as a yellow solid.

^{1}H NMR ($CDCl_3$, 400 MHz): δ 8.85 (s, 1H), 8.16 (s, 1H), 8.05 (d, J = 8.8 Hz, 1H). 7.57 (s, 1H), 7.27 (d, J = 7.2 Hz, 1H). LCMS (m/z): 252.97 $[M+H]^+$.

Step-2: Synthesis of 5-(6-fluoropyridin-3-yl)-2-(thiazol-5-yl) thieno[3,2-*b*] pyridine (27):

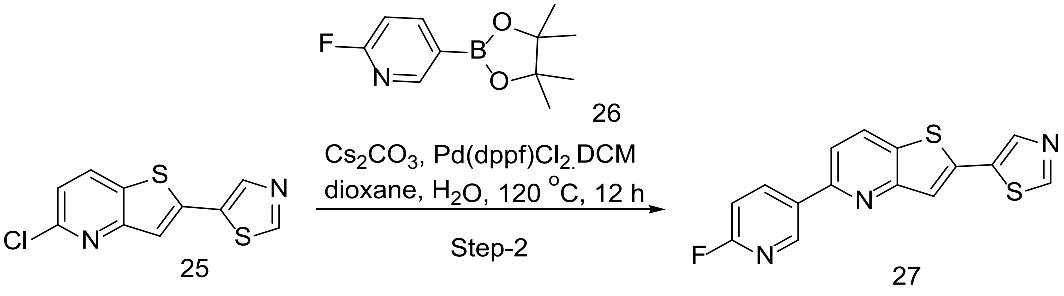

To a solution of 5-chloro-2-(thiazol-5-yl) thieno[3,2-*b*]pyridine (100 mg, 1.0 eq, 0.39 mmol) and 2-fluoro-5-(4,4,5,5-tetramethyl-1,3,2-dioxaborolan-2-yl)pyridine (80 mg, 0.9 eq, 0.35 mmol) in dioxane (4.0 mL), H_2O (0.6 mL) was added cesium carbonate (386 mg, 3.0 eq, 1.2 mmol) at room temperature and purged with argon gas for 20 min. After that, Pd(dppf)Cl_2.DCM (16 mg, 0.05 eq, 0.02 mmol) was added under argon atmosphere and heated at 120 °C for 5 h. The reaction progress was monitored by TLC. After completion of reaction, the reaction filtered through celite pad and washed with EtOAc (15 mL). The filtrate was washed with water (5 mL) followed by brine solution (5 mL). The organic layer was dried over Na_2SO_4, and concentrated under reduced pressure to get residue. The residue was purified by silica gel column chromatography using EtOAc/*n*-hexane as eluent to afford 5-(6-fluoropyridin-3-yl)-2-(thiazol-5-yl) thieno[3,2-*b*] pyridine (200 mg, crude) as yellow gummy solid.

Step-3: Synthesis of (*R*)-5-(6-(3-fluoropyrrolidin-1-yl) pyridin-3-yl)-2-(thiazol-5-yl) thieno[3,2-*b*] pyridine (LMD-006):

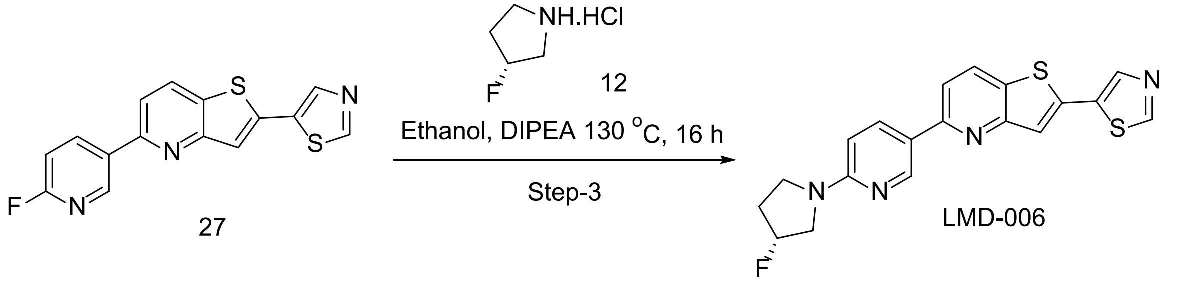

To a stirred solution of 5-(6-fluoropyridin-3-yl)-2-(thiazol-5-yl) thieno [3,2-*b*] pyridine (200 mg, 1.0 eq, 0.64 mmol) in EtOH (10 mL) was added (*R*)-3-fluoropyrrolidine hydrochloride (1/1) (284 mg, 5.0 eq, 3.2 mmol) and *N*-ethyldiisopropylamine (823 mg, 10.0 eq, 6.38 mmol) at room temperature and stirred at 130 °C for 16 h. The progress of the reaction mixture was monitored by TLC. After completion of the reaction, solvent was removed the under reduced pressure to get crude. The crude was purified by silica gel column chromatography using 15% EtOAc/hexane as eluent results title compound, then re-purified by prep-HPLC to afford (*R*)-5-(6-(3-fluoropyrrolidin-1-yl) pyridin-3-yl)-2-(thiazol-5-yl) thieno[3,2-*b*] pyridine (16 mg, 6.55%) as a pale-yellow solid. ^{1}H NMR (DMSO-d_6, 400 MHz): δ 8.84 - 8.82 (m, 2H), 8.28 (dd, J = 2.4 Hz, 8.8 Hz, 1H), 8.16 (s, 1H), 8.11 (d, J = 8.4 Hz, 1H), 7.68 (s, 1H), 7.62 (d, J = 8.8 Hz, 1H), 6.52 (d, J = 9.2 Hz, 1H), 5.42 (d, J = 53.2 Hz, 1H), 3.98 - 3.88 (m, 1H), 3.81 - 3.62 (m, 3H), 2.48 - 2.38 (m, 1H), 2.25 - 2.13 (m, 1H). ^{19}F NMR: -176.10 ppm. LCMS(ESI): m/z = 383.20 $[M+H]^+$.
HPLC purity: 99.36%

LMD-014

Scheme:

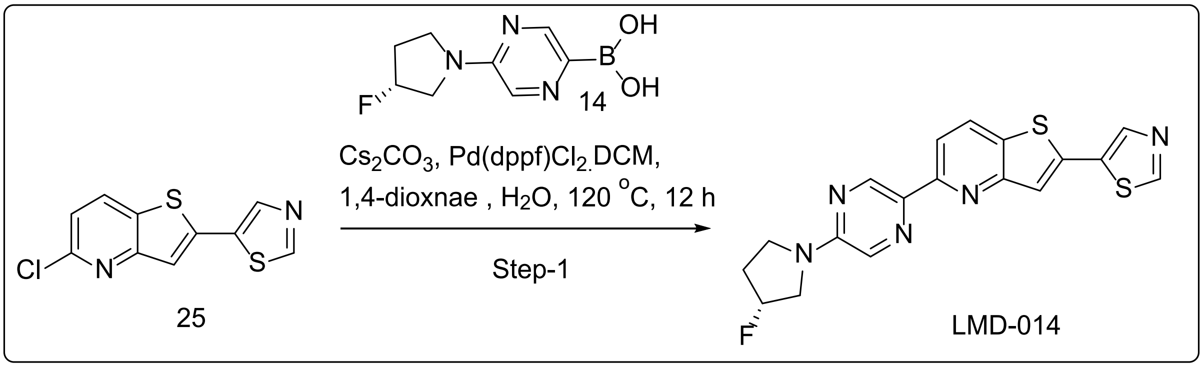

Step-1: Synthesis of (*R*)-5-(5-(3-fluoropyrrolidin-1-yl)pyrazin-2-yl)-2-(thiazol-5-yl)thieno[3,2-*b*]pyridine (LMD-014):

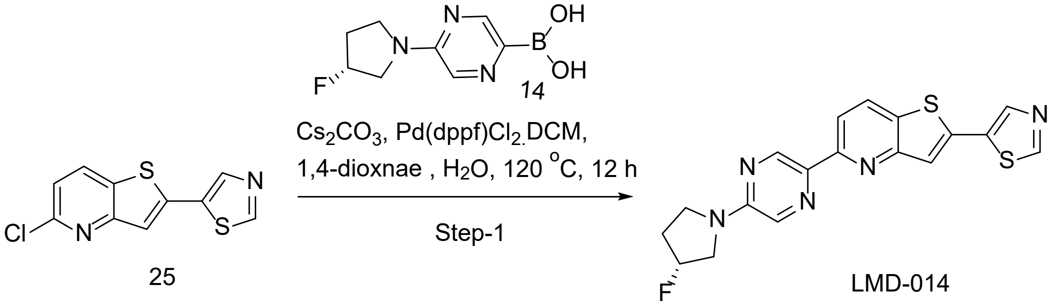

To a stirred solution of 5-chloro-2-(thiazol-5-yl)thieno[3,2-*b*]pyridine (200 mg, 1.0 eq, 0.79 mmol) and (*R*)-(5-(3-fluoropyrrolidin-1-yl)pyrazin-2-yl)boronic acid (343 mg, 2.0 eq, 1.6 mmol) in 1,4-Dioxane (6 mL), H_2O (1 mL) was added Cs_2CO_3 (773 mg, 3.0 eq, 2.4 mmol) at room temperature, purged with argon gas for 20 min. After that, Pd(dppf)Cl_2.DCM (32 mg, 0.05 eq, 0.04 mmol) was added under argon atmosphere and heated at 120 °C for 12 h. The reaction progress was monitor by TLC. After completion of the reaction, the reaction filtered through celite pad and washed with EtOAc (20 mL). The filtrate was washed with water (20 mL) followed by brine solution (20 mL). The organic layer was dried over Na_2SO_4, and concentrated under reduced pressure to get crude residue. The crude residue was purified by silica gel column chromatography using EtOAc/*n*-hexane as eluent to afford the title compound, which was re-purified by using prep-HPLC to obtain (*R*)-5-(5-(3-fluoropyrrolidin-1-yl) pyrazin-2-yl)-2-(thiazol-5-yl) thieno [3,2-*b*] pyridine (9 mg, 2.97%) as a yellow solid. ^{1}H-NMR (DMSO-d_6, 400 MHz): δ 9.23 (s,1H), 9.09 (s, 1H), 8.52 (d, *J* = 8.4 Hz, 1H), 8.45 (s, 1H), 8.16 (d, *J* = 8.4 Hz, 1H), 8.14 (s, 1H), 7.97 (s, 1H), 5.59 - 5.45 (d, *J* = 48.0 Hz, 1H), 3.93 - 3.67 (m, 3H), 3.58 - 3.54 (m, 1H), 2.20 - 2.17 (m, 2H), ^{19}F NMR: -175.31 ppm. LCMS (m/z): 384.24 $[M+H]^+$. HPLC Purity: 96.47%

LMD-062

Scheme:

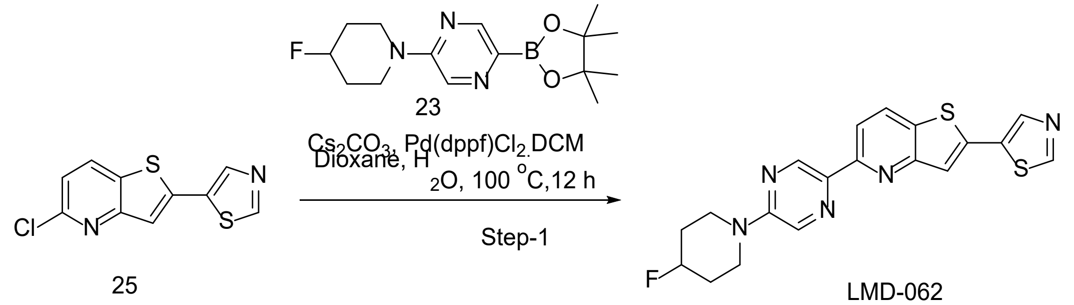

Step-1: Synthesis of 5-(5-(4-fluoropiperidin-1-yl) pyrazin-2-yl)-2-(thiazol-5-yl) thieno[3,2-*b*] pyridine (LMD-062):

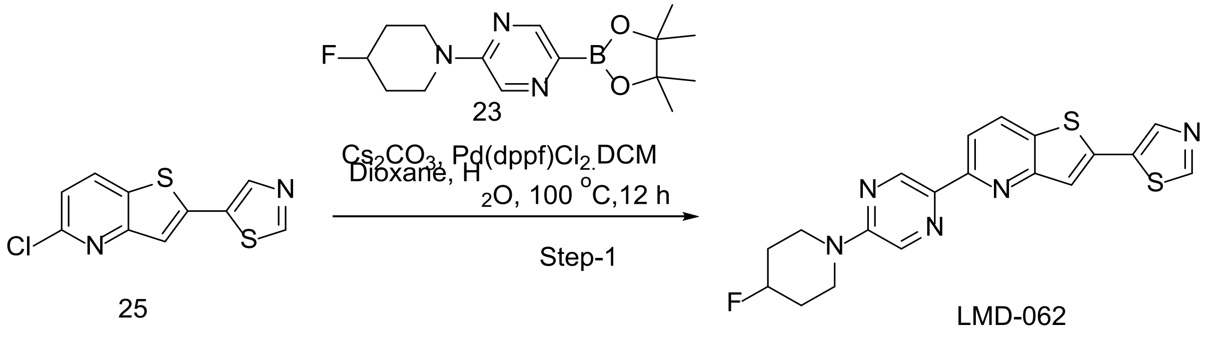

To a stirred solution of 5-chloro-2-(thiazol-5-yl)thieno[3,2-*b*] pyridine (100 mg, 0.4 mmol) and 2-{2-[(R)-3-fluoro-1-pyrrolidinyl]-5-pyrimidinyl}-4,4,5,5-tetramethyl-1,3,2-dioxaborolane (243 mg, 2.0 eq, 0.8 mmol) in 1,4-dioxane (2 mL), H_2O (0.5 mL) was added cesium carbonate (396 mg, 3.0 eq, 1.21 mmol) into a sealed tube and degassed with argon gas for 10 min. After that, Pd(dppf)Cl_2.DCM (16.6 mg, 0.05 eq, 0.023 mmol) was added under argon atmosphere and heated at 100 °C for 12 h. The reaction progress was monitored by TLC. After completion of the reaction, filtered through celite pad and washed with EtOAc (10 mL). The filtrate was washed with water (10 mL) and brine solution (10 mL). The organic layer was dried over Na_2SO_4, and concentrated under reduced pressure. The residue was purified on column chromatography using EtOAc/*n*-hexane as eluent to afford 5-(5-(4-fluoropiperidin-1-yl) pyrazin-2-yl)-2-(thiazol-5-yl) thieno[3,2-*b*] pyridine (1.2 mg, 0.76%) as an off white solid. ^{1}H NMR (DMSO-*d*$_6$, 400 MHz): δ 9.23 (s, 1H), 9.08 (s, 1H), 8.53 (d, *J* = 8.4 Hz, 1H), 8.49 (s, 1H), 8.45 (s, 1H), 8.16 (d, *J* = 8.4 Hz, 1H), 7.97 (s, 1H), 4.90 (d, *J* = 48.4 Hz, 1H), 3.90 - 3.65 (m, 4H), 2.10 - 1.94 (m, 2H), 1.85 - 1.77 (m, 2H).
^{19}F NMR: -177.53 ppm. LCMS (ESI): m/z = 398.2 [M+H]$^+$. HPLC purity: 97.87%

LMD-015

Scheme:

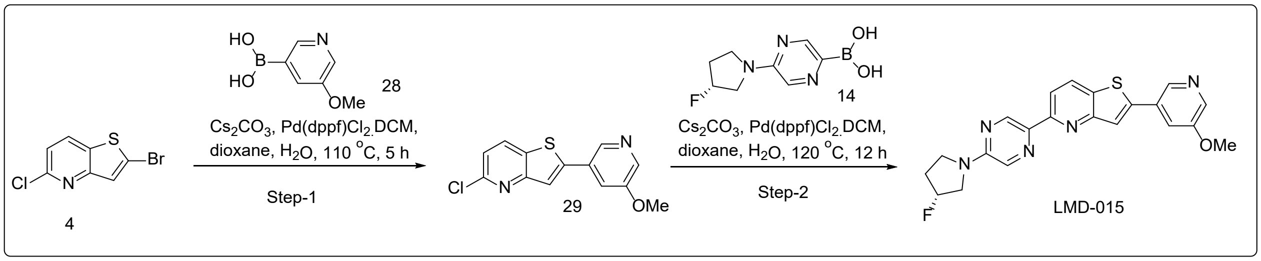

Step-1: Synthesis of 5-chloro-2-(5-methoxypyridin-3-yl) thieno [3,2-*b*] pyridine (29):

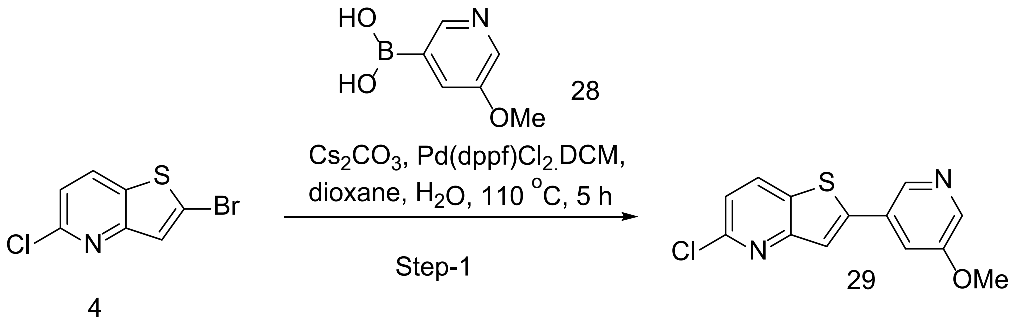

To a solution of 2-bromo-5-chlorothieno[3,2-*b*]pyridine (1.0 g, 1.0 eq, 4.05 mmol) and (5-methoxypyridin-3-yl)boronic acid (557 mg, 0.9 eq, 3.65 mmol) in dioxane (9.0 mL), H_2O (3.0 mL) was added Cs_2CO_3 (3.94 g, 3.0 eq, 12.15 mmol) at room temperature and purged with argon gas for 20 min. After that, Pd(dppf)Cl_2.DCM (165 mg, 0.05 eq, 0.2 mmol) was added under argon atmosphere and heated at 110 °C for 5 h. The reaction progress was monitored by TLC. After completion of the reaction, the reaction filtered through celite pad and washed with EtOAc (30 mL). The filtrate was washed with water (20 mL) followed by brine solution (20 mL). The organic layer was dried over Na_2SO_4, and concentrated under reduced pressure to get residue. The residue was purified by silica gel column chromatography using EtOAc/*n*-hexane as eluent to afford 5-chloro-2-(5-methoxypyridin-3-yl) thieno[3,2-*b*] pyridine (0.75 g, 67.35%) as an off white solid. ^{1}H NMR ($CDCl_3$, 400 MHz): δ 8.61 (s, 1H), 8.37 (d, J = 2.4 Hz, 1H), 8.12 (d, J = 8.4 Hz, 1H), 7.74 (s, 1H), 7.48 - 7.47 (m, 1H), 7.31 (d, J = 8.4 Hz, 1H), 3.97 (s, 3H). LCMS (ESI): m/z = 277.08 $[M+H]^+$

Step-2: Synthesis of (*R*)-5-(5-(3-fluoropyrrolidin-1-yl) pyrazin-2-yl)-2-(5-methoxypyridin-3-yl) thieno[3,2-*b*] pyridine (LMD-015):

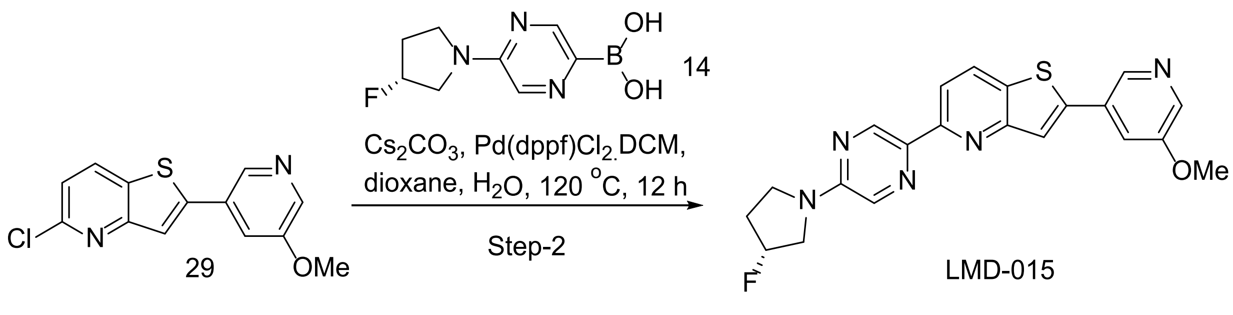

To a solution of 5-chloro-2-(5-methoxypyridin-3-yl) thieno [3,2-*b*] pyridine (600 mg, 1.0 eq, 2.2 mmol) and (*R*)-(5-(3-fluoropyrrolidin-1-yl) pyrazin-2-yl) boronic acid (917 mg, 2.0 eq, 4.35 mmol) in dioxane (6.0 mL), H_2O (2.0 mL) was added Cs_2CO_3 (2.11 g, 3.0 eq, 6.56 mmol) at room

temperature and purged with argon gas for 20 min. After that, Pd(dppf)Cl_2.DCM (89 mg, 0.05 eq, 0.11 mmol) was added under argon atmosphere and heated at 110 °C for 5 h. The reaction progress was monitored by TLC. After completion of reaction, the reaction filtered through celite pad and washed with EtOAc (30 mL). The filtrate was washed with water (20 mL) followed by brine solution (20 mL). The organic layer was dried over Na_2SO_4, and concentrated under reduced pressure to get residue. The residue was purified by silica gel column chromatography using EtOAc/*n*-hexane as eluent, then re-purified by prep-HPLC to afford (*R*)-5-(5-(3-fluoropyrrolidin-1-yl) pyrazin-2-yl)-2-(5-methoxypyridin-3-yl) thieno[3,2-*b*] pyridine (9.84 mg, 1.11%) as a pale-yellow solid. ^{1}H NMR (DMSO-d_6, 400 MHz): δ 9.10 (d, J = 1.2 Hz, 1H), 8.67 (d, J = 1.6 Hz, 1H), 8.54 (d, J = 8.4 Hz, 1H), 8.36 (d, J = 2.4 Hz, 1H), 8.30 (s, 1H), 8.17 (d, J = 8.4 Hz, 1H), 8.14 (d, J = 1.2 Hz, 1H), 7.89 (t, J = 4.4 Hz, 1H), 5.51 (d, J = 52.8 Hz, 1H), 3.95 (s, 3H), 3.93 - 3.67 (m, 3H), 3.60 - 3.53 (m, 1H), 2.32 - 2.17 (s, 2H). 19F NMR: -175.30 ppm. LCMS(ESI): m/z = 408.4 $[M+H]^+$. HPLC purity: 97.70%

LMD-049

Scheme:

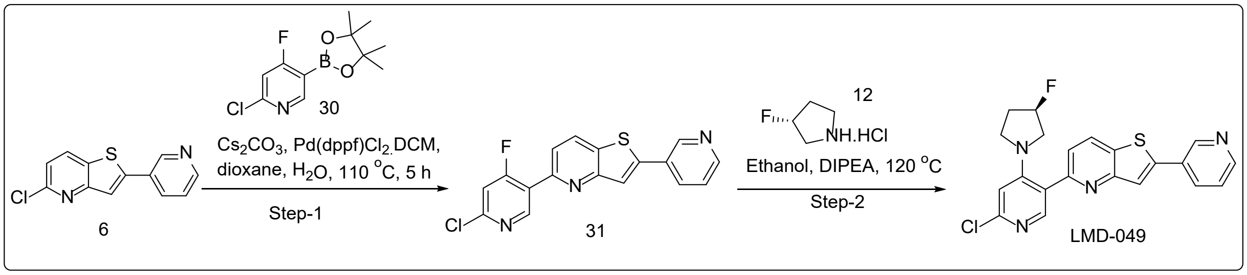

Step-1: Synthesis of 5-(6-chloro-4-fluoropyridin-3-yl)-2-(pyridin-3-yl) thieno[3,2-*b*] pyridine (31):

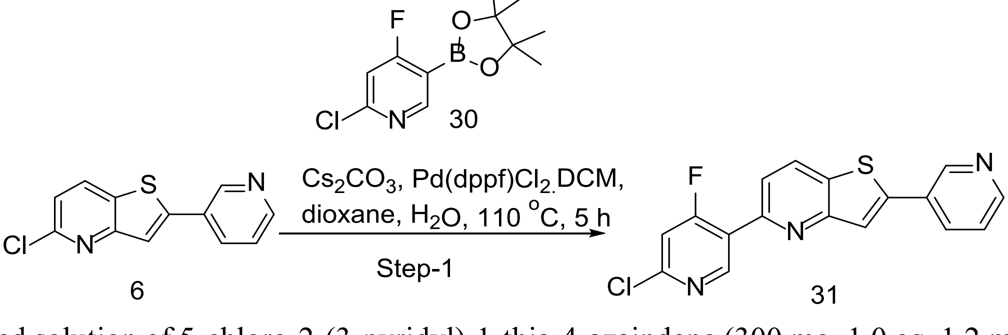

To a stirred solution of 5-chloro-2-(3-pyridyl)-1-thia-4-azaindene (300 mg, 1.0 eq, 1.2 mmol) and 2-(6-chloro-4-fluoro-3-pyridyl)-4,4,5,5-tetramethyl-1,3,2-dioxaborolane (626 mg, 2.0 eq, 2.4 mmol) in 1,4-Dioxane (4 mL), H_2O (1.0 mL) was added Cs_2CO_3 (1.2 g, 3.0 eq, 3.65 mmol) into a

seal tube. The reaction was degassed with argon for 20 min. Then, Pd(dppf)Cl_2.DCM (99.3 mg, 0.1 eq, 122 μmol) was added under argon and heated at 100 °C for 12 h. The progress of the reaction was monitor by TLC. After completion, the reaction was cooled to room temperature, filter through celite pad, washed with EtOAc (20 mL). The filtrate was washed with water (2 x 20 mL) and brine solution (10 mL). The organic layer was dried over Na_2SO_4, and concentrated under reduced pressure to get crude. The crude residue was purified on column chromatography using EtOAc/*n*-hexane as eluent to afford 5-6-chloro-4-fluoropyridin-3-yl)-2-(pyridin-3-yl) thieno[3,2-*b*] pyridine (220 mg, 53.6%) as an off white solid. LCMS(ESI): m/z =342.13 $[M+H]^+$.

Step-2: Synthesis of (*R*)-5-(6-chloro-4-(3-fluoropyrrolidin-1-yl)pyridin-3-yl)-2-(pyridin-3-yl)thieno[3,2-*b*]pyridine (LMD-049):

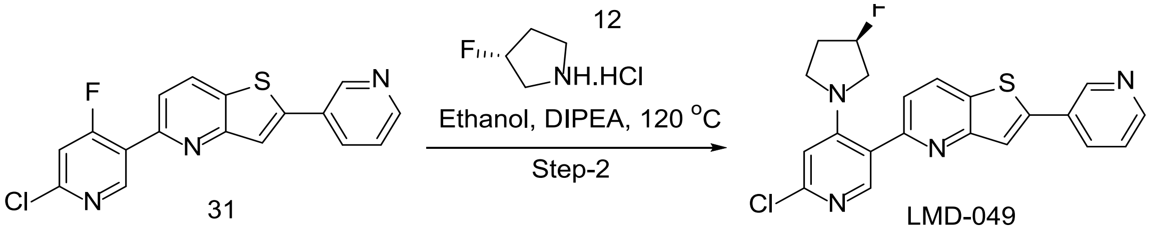

To a stirred solution of 5-(6-chloro-4-fluoro-3-pyridyl)-2-(3-pyridyl)-1-thia-4-azaindene (100 mg, 1.0 eq, 293 μmol) in EtOH (7.44 mL) in a seal tube was added 3-fluoropyrrolidine—hydrogen chloride (1/1) (147 mg, 4.0 eq, 1.17 mmol), followed by *N*-ethyldiisopropylamine (189 mg, 5.0 eq, 1.46 mmol) at room temperature and heated at 130 °C for 16 h. The progress of the reaction was monitored by TLC. After completion, the solvent was removed under reduced pressure to get crude. The crude residue was taken up in dichloromethane (20 mL), was washed with saturated NH_4Cl solution (10 mL) and water (10 mL). The organic layer was dried over Na_2SO_4, and concentrated under reduced pressure to get crude. The crude residue was purified by prep-HPLC to afford (*R*)-5-(6-chloro-4-(3-fluoropyrrolidin-1-yl)pyridin-3-yl)-2-(pyridin-3-yl)thieno[3,2-*b*]pyridine (30 mg, 24.8 %) as an off white solid. ^{1}H NMR (DMSO-*d*$_6$, 400 MHz): δ 9.12 (s, 1H), 8.65 (d, J = 4.4 Hz, 1H), 8.69 (d, J = 8.4 Hz, 1H), 8.28 (d, J = 8.0 Hz, 1H), 8.24 (s, 1H), 7.98 (s, 1H), 7.58 – 7.55 (m, 1H), 7.51 (d, J = 8.0 Hz, 1H), 6.80 (s, 1H), 5.24 (d, J = 53.2 Hz, 1H), 3.30 – 3.03 (m, 4H), 2.13 – 1.91 (m, 2H). ^{19}F NMR: -175.28 ppm. LCMS(ESI): m/z = 411.0 $[M+H]^+$.
HPLC purity: 99.32%

LMD-024

Scheme:

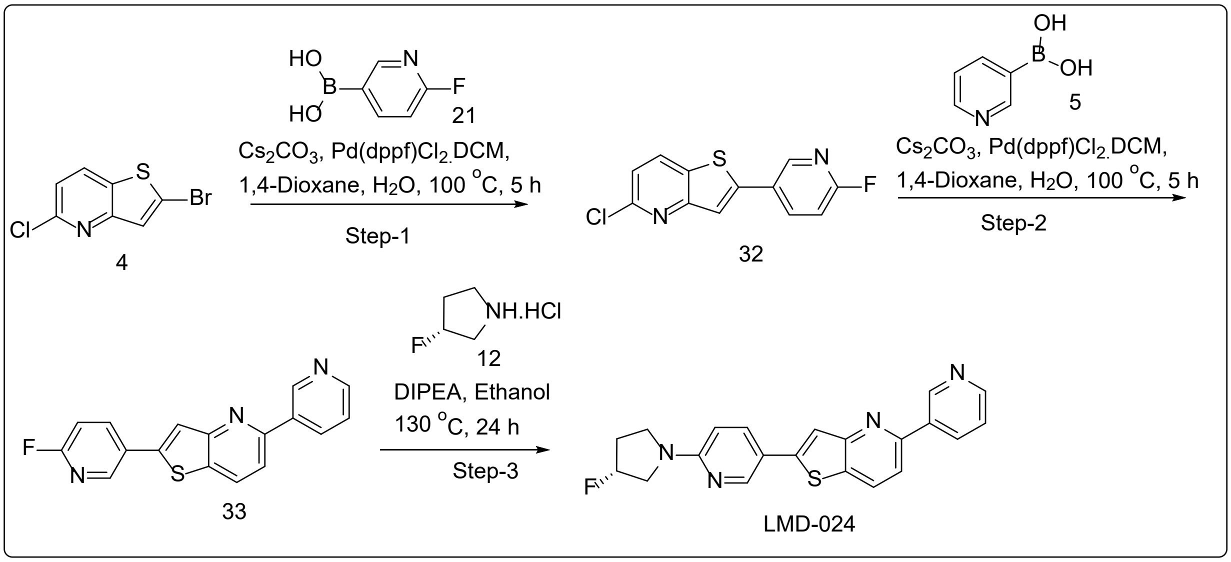

Step-1: **Synthesis of 5-chloro-2-(6-fluoropyridin-3-yl) thieno[3,2-*b*] pyridine (32):**

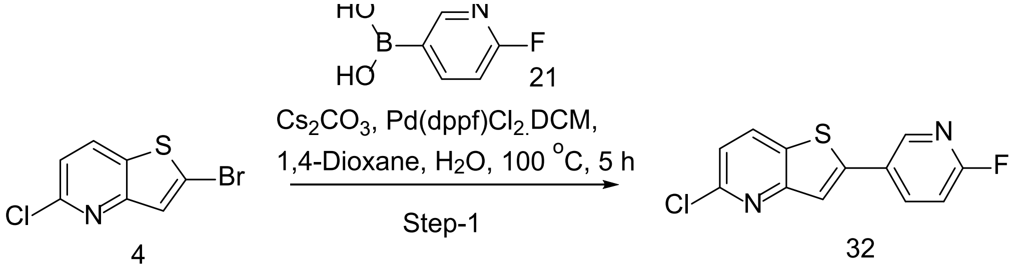

To a stirred solution of 2-bromo-5-chlorothieno[3,2-*b*] pyridine (200 mg, 1.0 eq, 0.81 mmol) and (6-fluoropyridin-3-yl) boronic acid (102 mg, 0.9 eq, 0.73 mmol) in 1,4-Dioxane (6 mL), H_2O (1 mL) was added Cs_2CO_3 (787 mg, 3.0 eq, 24.1 mmol) at room temperature and purged with argon for 20 min. After that, $Pd(dppf)Cl_2$.DCM (33 mg, 0.05 eq, 0.40 mmol) was added under argon atmosphere and heated at 100 °C for 5 h. The reaction progress was monitored by TLC. After completion of the reaction, the reaction was filtered through celite pad and washed with EtOAc (20 mL). The filtrate was washed with water (20 mL) and brine (10 mL). The organic layer was dried over Na_2SO_4, and concentrated under reduced pressure to get crude. The crude residue was purified on column chromatography using 10% EtOAc/*n*-hexane as eluent to afford 5-chloro-2-(6-fluoropyridin-3-yl) thieno[3,2-*b*] pyridine (220 mg, 70%) as an off white solid. ^{1}H NMR

(DMSO-d_6, 400 MHz): δ 8.79 (s. 1H), 8.59 (d, J = 8.4 Hz, 1H), 8.35 - 8.38 (m, 1H), 8.13 (s, 1H), 7.49 (d, J = 8.0 Hz, 1H), 7.40 -7.33 (m, 1H). LCMS(ESI); m/z = 360.06 $[M+H]^+$.

Step-2: Synthesis of 2-(6-fluoropyridin-3-yl)-5-(pyridin-3-yl)thieno[3,2-*b*]pyridine (33):

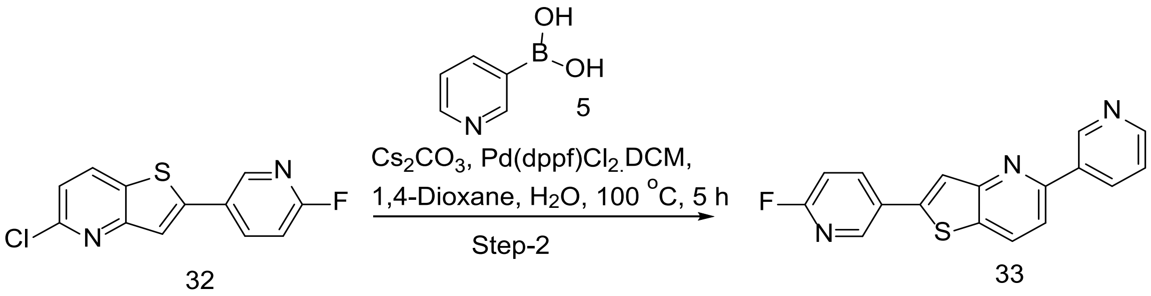

To a stirred solution of 5-chloro-2-(6-fluoropyridin-3-yl) thieno[3,2-*b*]pyridine (150 mg, 1.0 eq, 0.57 mmol) and pyridin-3-ylboronic acid (690 mg, 1.0 eq, 0.57 mmol) in 1,4-Dioxane (5 mL) and water (1 mL) was added Cs_2CO_3 (554 mg, 3.0 eq, 1.7 mmol) at room temperature and purged with argon for 20 min. After that, Pd(dppf)Cl_2.DCM (20 mg, 0.05 eq, 0.03 mmol) was added under argon atmosphere and heated at 100 °C for 5 h. The reaction progress was monitored by TLC. After completion of reaction, the reaction was filtered through celite pad and washed with EtOAc (20 mL). The filtrate was washed with water (20 mL) and brine solution (10 mL). The organic layer was dried over Na_2SO_4, and concentrated under reduced pressure to get crude. The crude residue was purified on column chromatography using 25% EtOAc/hexane as eluent to afford 2-(6-fluoropyridin-3-yl)-5-(pyridin-3-yl) thieno[3,2-*b*] pyridine (130 mg, 74.64%) as an off white solid. ^{1}H NMR (DMSO-d_6, 400 MHz): δ 9.36 (s, 1H), 8.81 (s. 1H), 8.67 - 8.64 (m, 2H), 8.49 - 8.54 (m, 2H),) 8.26 (s, 1H), 8.08 (d, J = 8.0 Hz, 1H), 7.58 - 7.55 (m, 1H), 7.40 - 7.37 (m, 1H).
LCMS(ESI); m/z = 308.22 $[M+H]^+$.

Step-3: Synthesis of (*R*)-2-(6-(3-fluoropyrrolidin-1-yl)pyridin-3-yl)-5-(pyridin-3-yl)thieno[3,2-*b*]pyridine (LMD-024):

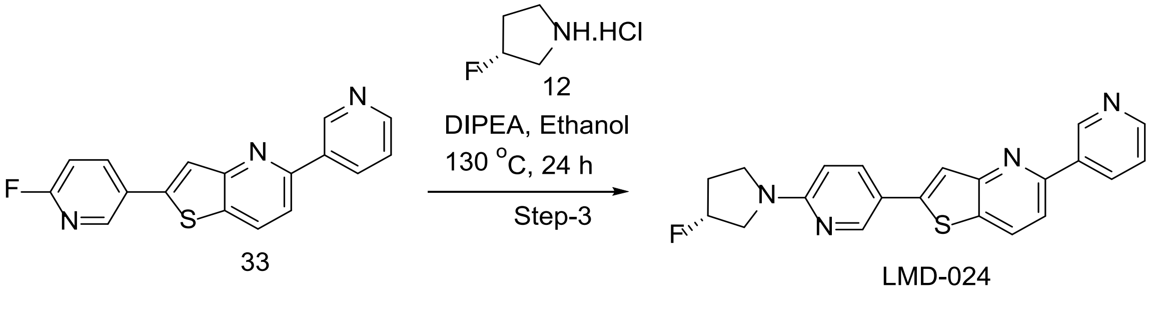

To a solution of 2-(6-fluoropyridin-3-yl)-5-(pyridin-3-yl) thieno[3,2-*b*] pyridine (130 mg, 1.0 eq, 0.42 mmol) and (*R*)-3-fluoropyrrolidine hydrochloride (51 mg, 1.1 eq, 0.49 mmol) in EtOH (5 mL) was added DIPEA (0.118 mL, 1.6 eq, 0.68 mmol). The tube was sealed and heated at 130 °C for 24 h. The reaction progress was monitored by TLC. After completion of reaction, the solvent was removed under reduced pressure and the residue was taken up in DCM (30 mL), washed with saturated NH_4Cl solution (30 mL) and H_2O (20 mL). The organic layer was dried over Na_2SO_4, and concentrated under reduced pressure to get crude. The crude residue was purified by Prep-HPLC to afford (*R*)-2-(6-(3-fluoropyrrolidin-1-yl) pyridin-3-yl)-5-(pyridin-3-yl) thieno[3,2-*b*] pyridine (36 mg, 22%) as a yellow solid. ^1H NMR (DMSO-d_6, 400 MHz): δ 9.34 (s. 1H), 8.66 (d, *J* = 4.0 Hz, 2H), 8.51 (t, *J* = 4.0 Hz, 2H), 8.1 (d, *J* = 4.0 Hz, 1H), 7.94 - 7.98 (d, *J* = 2.0 Hz, 2H), 7.54 -7.57 (m, 1H), 6.66 (d, *J* = 8.8 Hz, 1H), 5.48 (d, *J* = 52.0 Hz, 1H), 3.9 - 3.4 (m, 3H), 3.55 - 3.45 (m, 1H), 2.4 - 2.1 (m, 2H). ^{19}F NMR: -175.03 ppm. LCMS(ESI); m/z = 377.1 $[M+H]^+$
HPLC purity: 96.59%

LMD-036

Scheme:

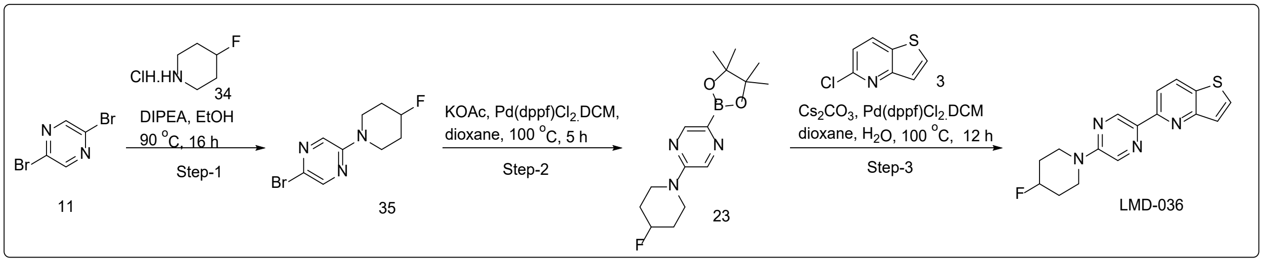

Step-1: Synthesis of 2-bromo-5-(4-fluoropiperidin-1-yl) pyrazine (35):

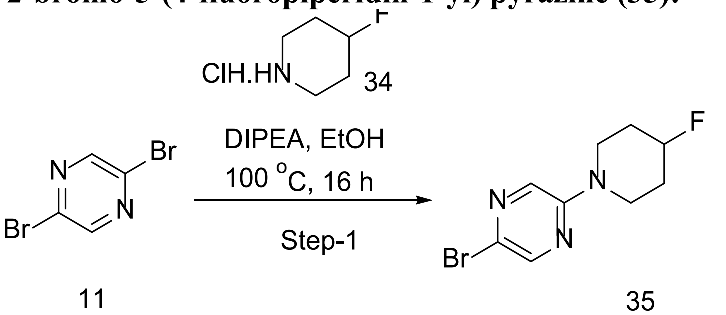

To a solution of 2,5-dibromopyrazine (3.0 g, 1.0 eq, 12 mmol) in EtOH (30 mL) was added 4-fluoropiperidine hydrochloride (3.4 g, 2.0 eq, 25 mmol) and *N*-ethyldiisopropylamine (6.41 mL, 3.0 eq, 36 mmol) at room temperature and heated at 100 °C for 12 h. The progress of reaction was monitor by TLC. After completion of reaction, the solvent was removed under reduced pressure

to get crude residue. The crude residue was taken in dichloromethane (50 mL), washed with saturated ammonium chloride solution (50 mL) and water (2 x 25 mL). The organic layer was dried over Na_2SO_4, and concentrated under reduced pressure to get crude. The crude residue was purified column chromatography using 10% EtOAc/hexane as eluent to afford 2-bromo-5-(4-fluoropiperidin-1-yl)pyrazine (2.05 g, 60%) as an off white solid. LCMS :m/z = 377.1 $[M+H]^+$

Step-2: Synthesis of 2-(4-fluoropiperidin-1-yl)-5-(4,4,5,5-tetramethyl-1,3,2-dioxaborolan-2-yl)pyrazine (23):

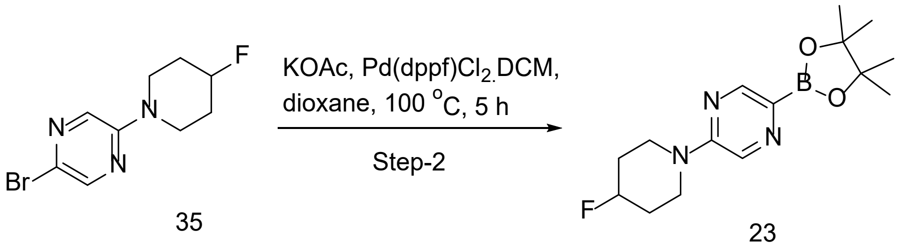

To a stirred solution of 2-bromo-5-(4-fluoropiperidin-1-yl)pyrazine (2.0 g, 1.0 eq, 7.69 mmol) in 1,4-dioxane (20 mL) was added bispinacolatodiboron (3.91 g, 2.0 eq, 15.4 mmol) followed by anhydrous potassium acetate (2.26 g, 3.0 eq, 23.1 mmol) at room temperature After that, the reaction was purged with argon gas for 20 min. Then, $Pd(dppf)Cl_2$.DCM (314 mg, 0.1 eq, 0.08 mmol) was added under argon atmosphere, and heated at 100 °C for 5 h. The reaction progress was monitored by TLC. After completion, the reaction mixture was filtered through celite pad and washed with EtOAc (50 mL). The filtrate was concentrated to get crude residue, which was washed with *n*-hexane (100 mL) and concentrated under reduced pressure to obtain 2-(4-fluoropiperidin-1-yl)-5-(4,4,5,5-tetramethyl-1,3,2-dioxaborolan-2-yl) pyrazine (3 g, crude) as brown color solid. This was used for next step without any further purification. ^{1}H NMR (DMSO-d_6, 400 MHz): δ 8.48 (s, 1H), 8.17 (s, 1H), 4.97 - 4.83 (m, 1H), 3.77 - 3.74 (m, 4H), 2.02 -1.92 (m, 4H).

Step-3: Synthesis of 5-(5-(4-fluoropiperidin-1-yl)pyrazin-2-yl)thieno[3,2-*s*]pyridine (LMD-036):

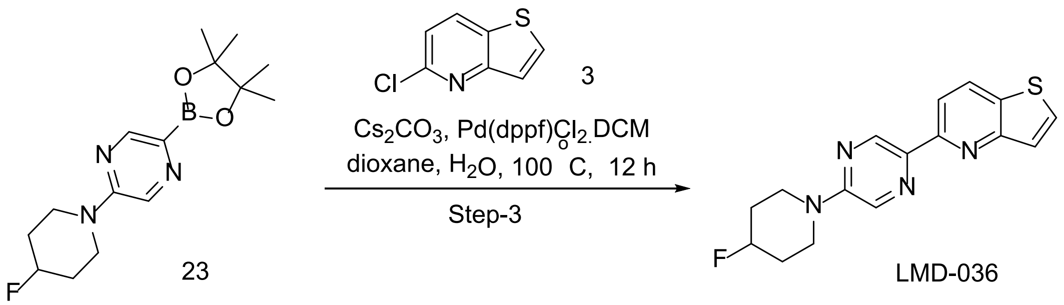

To a stirred solution of 5-chlorothieno[3,2-*b*]pyridine (250 mg, 0.91 eq, 1.47 mmol) and 2-(4-fluoropiperidin-1-yl)-5-(4,4,5,5-tetramethyl-1,3,2-dioxaborolan-2-yl)pyrazine (500 mg, 1.1 eq,

1.63 mmol) in 1,4-Doxane (5 mL), H_2O (0.5 mL) was added cesium carbonate (1.59 g, 3.0 eq, 4.9 mmol) at room temperature and purged with argon gas for 20 min. Then, Pd(dppf)Cl_2.DCM (133 mg, 0.1 eq, 163 µmol) was added under argon atmosphere, and heated at 100 °C for 12 h. The reaction progress was monitor by TLC. After completion of reaction, the reaction was filter through celite pad and washed with EtOAc (25 mL). The filtrate was washed with water (10 mL), followed by brine solution (10 mL). The organic layer was dried over Na_2SO_4, and concentrated under reduced pressure to get residue. The residue was purified on column chromatography using EtOAc/n-hexane as eluent to afford 5-(5-(4-fluoropiperidin-1-yl)pyrazin-2-yl)thieno[3,2-*b*]pyridine (90 mg, 35%) as an off white solid. ^{1}H NMR (DMSO-d_6, 400 MHz): δ 9.08 (s, 1H), 8.53 (d, J = 8.4 Hz, 1H), 8.47 (s, 1H), 8.17 - 8.13 (m, 2H), 7.61 (d, J = 5.6 Hz, 1H), 4.9 (d, J = 49.2 Hz, 1H), 3.95 - 3.85 (m, 2H), 3.75 - 3.65 (m, 2H), 2.10- 1.90 (m, 2H), 1.85 - 1.70 (m, 2H). LCMS (m/z): 315 (M+H). HPLC purity: 99.41%

LMD-039

Scheme:

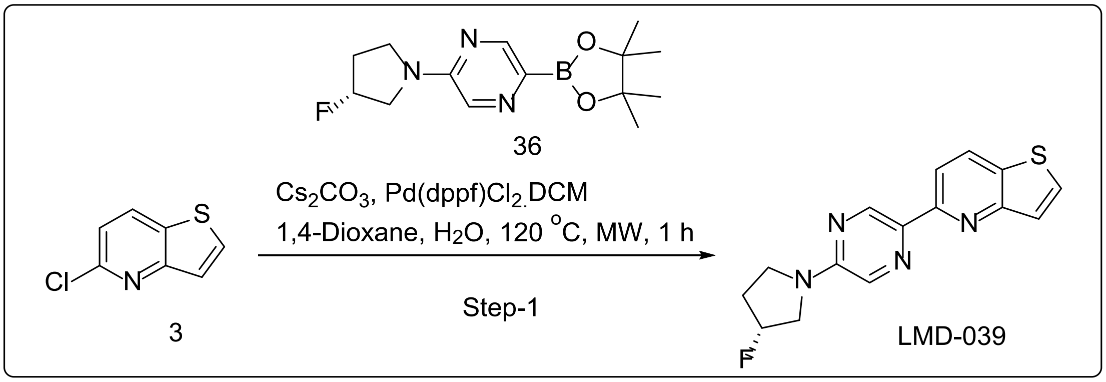

Step-1: Synthesis of (*R*)-5-(5-(3-fluoropyrrolidin-1-yl) pyrazin-2-yl) thieno[3,2-*b*] pyridine (LMD-039):

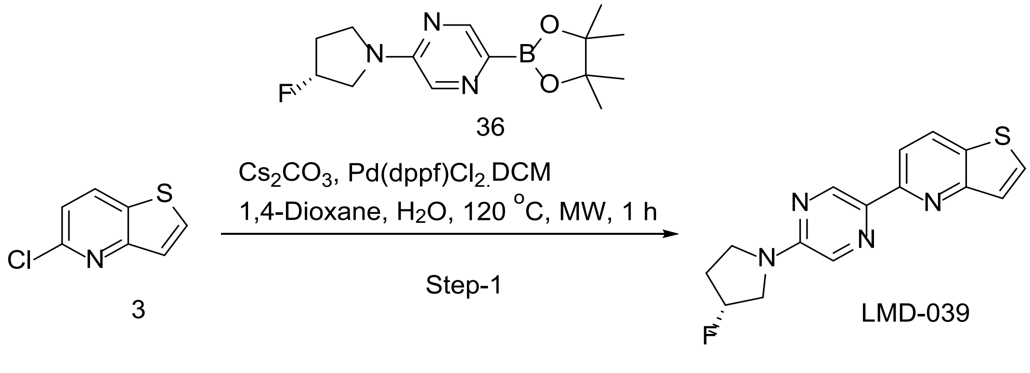

To a stirred solution of 5-chlorothieno[3,2-*b*]pyridine (250 mg, 1.0 eq, 1.47 mmol), and (*R*)-2-(3-fluoropyrrolidin-1-yl)-5-(4,4,5,5-tetramethyl-1,3,2-dioxaborolan-2-yl)pyrazine (647 mg, 1.5 eq, 2.2 mmol) in 1.4-dioxane (4 mL), water (1 mL) was added Cs_2CO_3 (1.43 g, 3.0 eq, 4.41 mmol) at room temperature and purged with argon for 20 min. After that, Pd(dppf)Cl_2.DCM (60 mg, 0.05 eq, 0.073 mmol) was added under argon atmosphere and irradiated at 120 °C under microwave for 1 h. The reaction progress was monitored by TLC. After completion of the reaction, filtered through celite pad and washed with EtOAc (50 mL). The filtrate was washed with water (10 mL) and brine solution (10 mL). The organic layer was dried over Na_2SO_4, and concentrated under reduced pressure to get crude. The residue was purified on column chromatography using 10% EtOAc/hexane as eluent to afford (*R*)-5-(5-(3-fluoropyrrolidin-1-yl) pyrazin-2-yl) thieno [3,2-*b*] pyridine (15 mg, 3.3%) as an off white solid. ^{1}H NMR (DMSO-d_6, 400 MHz): δ 9.10 (s, 1H), 8.53 (d, *J* = 8.8 Hz, 1H), 8.16 - 8.12 (m, 3H), 7.61 (d, *J* = 5.2 Hz, 1H), 5.56 (d, *J* = 48.0 Hz, 1H), 3.95 - 3.5 (m, 4H), 2.35 - 2.10 (m, 2H) ^{19}F NMR: -175.28 ppm. LCMS (ESI): m/z = 301.1 [M+H]$^+$. HPLC purity: 99.51%

LMD-026

Scheme:

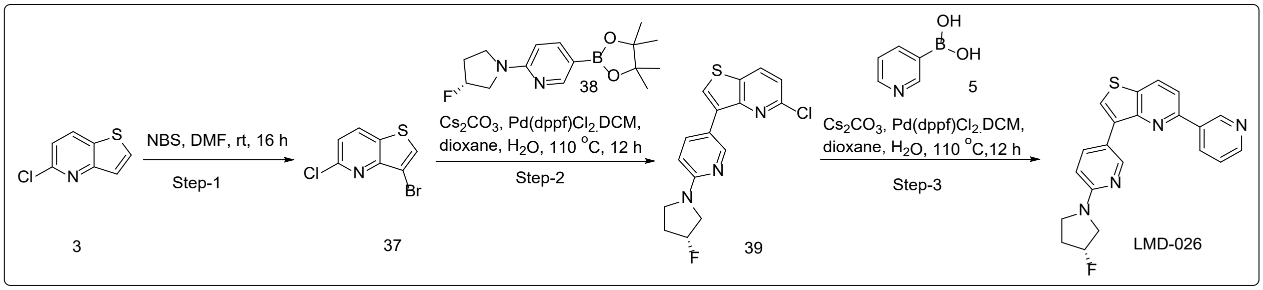

Step-1: Synthesis of 3-bromo-5-chlorothieno[3,2-*b*] pyridine (37):

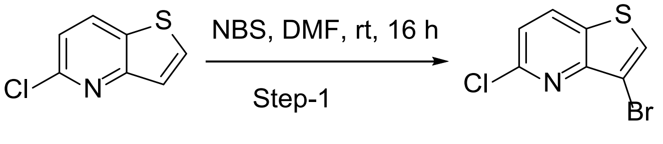

3 37

To a stirred solution of 5-chlorothieno[3,2-*b*]pyridine (1.0 g, 1.0 eq, 5.89 mmol) in DMF (10 mL) was added NBS (1.57 g, 1.5 eq, 8.84 mmol) at 0 °C and allowed to room temperature. The reaction progress was monitored by TLC. After completion of the reaction, ice-cold water was added to reaction and solids formation observed. The obtained solid was filtered and dried under vacuum.

The solid was dissolved in DCM. The organic layer was dried over Na_2SO_4 and concentrate under reduced pressure to afford 3-bromo-5-chloro-1-thia-4-azaindene (1.0 g, 68%) as a yellow solid. ^{1}H-NMR (DMSO-d_6, 400 MHz): δ 8.45 (d, J = 8.4 Hz, 1H), 7.73 (s, 1H), 7.43 (d, J = 8.4 Hz, 1H).

Step-2: Synthesis of (*R*)-5-chloro-3-(6-(3-fluoropyrrolidin-1-yl) pyridin-3-yl) thieno[3,2-*b*] pyridine (39):

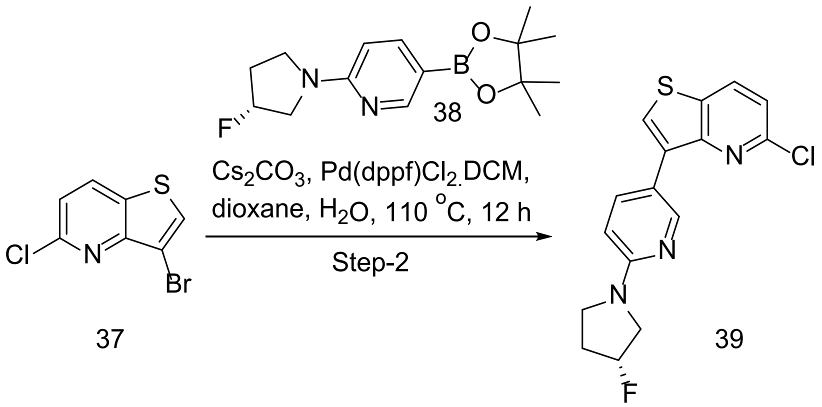

To a stirred solution of 3-bromo-5-chloro-1-thia-4-azaindene (200 mg, 1.0 eq, 805 μmol) in 1,4-dioxane (3 mL) and water (0.6 mL) was added (*R*)-2-(3-fluoropyrrolidin-1-yl)-5-(4,4,5,5-tetramethyl-1,3,2-dioxaborolan-2-yl)pyridine (212 mg, 0.9 eq, 724 μmol) followed by Cs_2CO_3 (787 mg, 3.0 eq, 2.41 mmol) and degassed with argon for 20 min. Then, Pd(dppf)Cl_2.DCM (16 mg, 0.05 eq, 0.02 mmol) was added under argon atmosphere and heated at 110 ºC for 12 h. The reaction progress was monitored by TLC. Then, the mixture was cooled to room temperature and filtered through celite pad, washed with EtOAc (10 mL). The filtrate was washed with water (2 x 5 mL) and brine (5 mL). The organic layer was dried over Na_2SO_4, and concentrated under reduced pressure to get crude. The crude residue was purified on column chromatography using EtOAc/*n*-hexane as eluent to afford (*R*)-5-chloro-3-(6-(3-fluoropyrrolidin-1-yl)pyridin-3-yl)thieno[3,2-*b*]pyridine (100 mg, 37%) a light brown solid. LCMS (ESI); m/z = 334.4 $[M+H]^{+.}$

Step-3: Synthesis of (*R*)-3-(6-(3-fluoropyrrolidin-1-yl) pyridin-3-yl)-5-(pyridin-3-yl) thieno[3,2-*b*] pyridine (LMD-026):

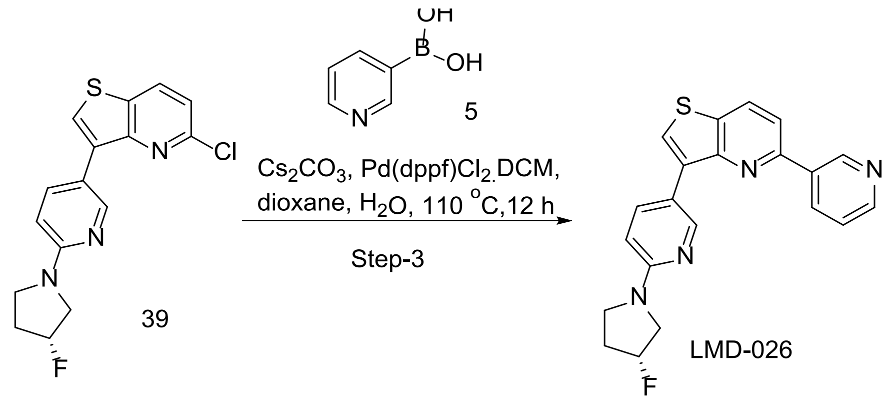

To a stirred solution of (*R*)-5-chloro-3-(6-(3-fluoropyrrolidin-1-yl)pyridin-3-yl)thieno[3,2-*b*]pyridine (100 mg, 1.0 eq, 0.3 mmol) in 1,4-Dioxane (3 mL) and water (1 mL) was added (3-pyridyl)boranediol (44.2 mg, 1.2 eq., 359 µmol) followed by Cs_2CO_3 (391 mg, 3.0 eq, 1.2 mmol) at room temperature and purged with argon gas for 20 min. After that, Pd(dppf)Cl_2.DCM (16 mg, 0.05 eq, 0.02 mmol) was added under argon atmosphere and heated at 110 °C for 12 h. The reaction progress was monitored by TLC. After completion of the reaction, the reaction mixture was cooled to room temperature and filtered through celite pad, washed with EtOAc (10 mL). The filtrate was washed with water (2 x 5 mL) and brine solution (5 mL). The organic layer was dried over Na_2SO_4, and concentrated under reduced pressure to get residue. The residue was purified on column chromatography using 25% EtOAc/n-hexane as eluent to afford title compound, it was re-purified by prep-HPLC to afford 3-{6-[(*R*)-3-fluoro-1-pyrrolidinyl]-3-pyridyl}-5-(3-pyridyl)-1-thia-4-azaindene (11 mg, 9.8 %) as a white solid. ^{1}H NMR (DMSO-*d*6, 400 MHz): δ 9.41 (s, 1H), 8.99 (s, 1H), 8.69 – 8.66 (m, 2H), 8.55 (d, *J* = 8.0 Hz, 1H), 8. 35 – 8.32 (m, 2H), 8.12 (d, *J* = 8.4 Hz, 1H), 7.6 – 7.56 (m, 1H), 6.69 (d, *J* = 8.8 Hz, 1H), 5.48 (d, *J* = 53.0 Hz, 1H), 3.85 – 3.46 (m, 4H), 2.3 – 2.12 (m, 2H). ^{19}F NMR: -174.70 ppm. LCMS (ESI): m/z = 377.46 $[M+H]^+$. HPLC purity: 99.01%

LMD-045

Scheme:

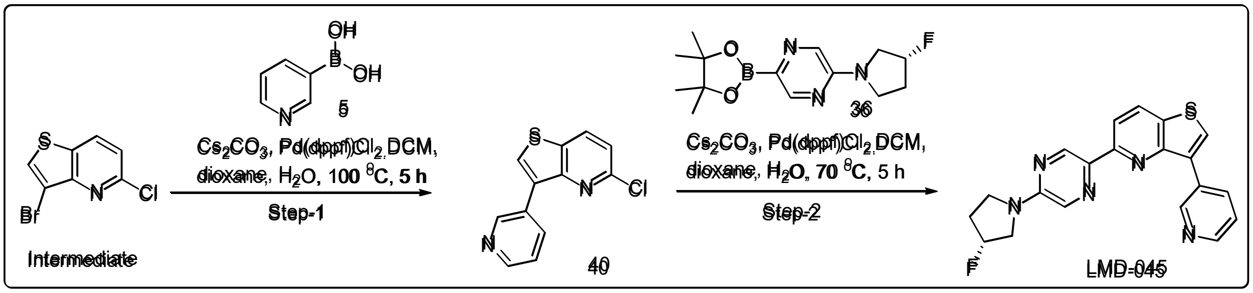

Step-1: Synthesis of 5-chloro-3-(pyridin-3-yl) thieno[3,2-*b*] pyridine (40):

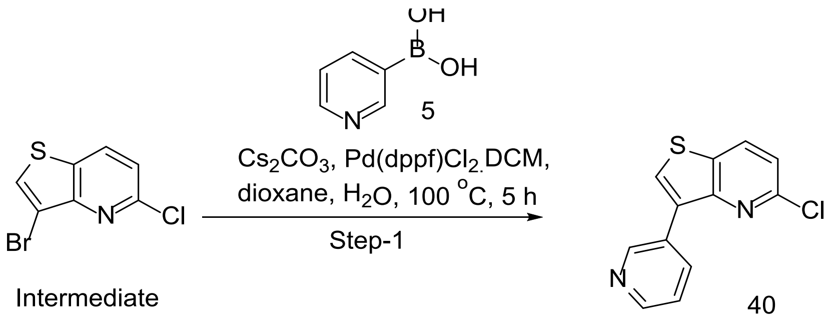

To a solution of 3-bromo-5-chlorothieno[3,2-*b*] pyridine (400 mg, 1.0 eq, 1.61 mmol) and pyridin-3-ylboronic acid (178 mg, 0.9 eq, 1.45 mmol), in 1,4-dioxane (5 mL), H_2O (1.2 mL) was added Cs_2CO_3 (1.57 g, 3.0 eq, 4.83 mmol) at room temperature and purged with argon gas for 20 min. After that, Pd(dppf)Cl_2.DCM (65 mg, 0.05 eq, 0.08 mmol) was added under argon atmosphere and heated at 100 °C for 5 h. The reaction progress was monitor by TLC. After completion, the reaction was filtered through celite pad and washed with EtOAc (20 mL). The filtrate was washed with water (2 x 10 mL), followed by brine solution (10 mL). The organic layer was dried over Na_2SO_4, and concentrated under reduced pressure to get crude. The crude residue was purified on column chromatography using 15% EtOAc/*n*-hexane as eluent to afford 5-chloro-3-(pyridin-3-yl)thieno[3,2-*b*]pyridine (210 mg, 50.37%) as an off white solid. ^{1}H NMR (DMSO-d_6, 400 MHz): δ 9.18 (s, 1H), 8.68 - 8.60 (m, 3H), 8.39 (d, *J* = 8.0 Hz, 1H), 7.59 - 7.54 (m, 2H). LCMS (m/z): 247.11 (M+H).

Step-2: Synthesis of (*R*)-5-(5-(3-fluoropyrrolidin-1-yl) pyrazin-2-yl)-3-(pyridin-3-yl)thieno[3,2-*b*]pyridine (LMD-045):

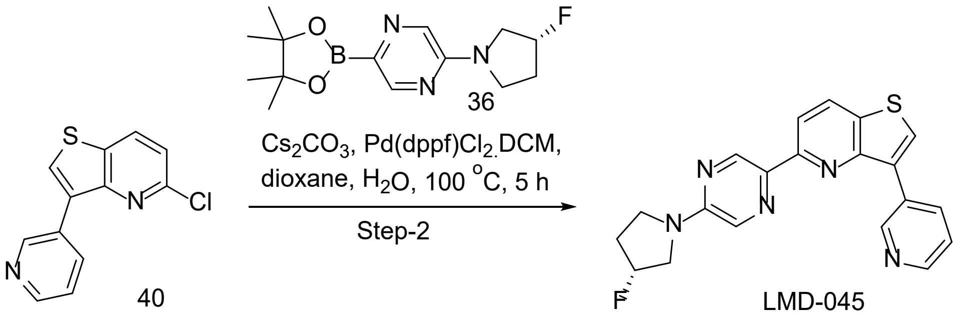

To a stirred solution of 5-chloro-3-(pyridin-3-yl)thieno[3,2-*b*]pyridine (200 mg, 1.0 eq, 0.81 mmol) and (*R*)-2-(5-(3-fluoropyrrolidin-1-yl)pyrazin-2-yl)-4,4,5,5-tetramethyl-1,3,2-dioxazolidine (481 mg, 2.0 eq, 1.62 mmol), in 1,4-dioxane (4 mL), H_2O (0.6 mL) was added cesium carbonate (792 mg, 3.0 eq, 2.43 mmol) at room temperature and purged with argon for 20

min. Then, Pd(dppf)Cl_2.DCM (33 mg, 0.05 eq, 0.04 mmol) was added under argon atmosphere and heated at 100 ºC for 12 h. The progress of the reaction was monitor by TLC. After completion, the reaction was filtered on celite pad, washed with EtOAc (20 mL). The filtrate was washed with water (20 mL), brine solution (10 mL). The organic layer was dried over Na_2SO_4, and concentrated under reduced pressure to get crude. The residue was purified on column chromatography using 25% EtOAc/*n*-hexane as eluent to afford the title compound, which was re-purified by using prep-HPLC. The obtained fractions were concentrated and kept for lyophilization to afford (*R*)-5-(5-(3-fluoropyrrolidin-1-yl) pyrazin-2-yl)-3-(pyridin-3-yl) thieno[3,2-*b*] pyridine (2.4 mg, 0.78%) as an off white solid. ^{1}H NMR (DMSO-d_6, 400 MHz): δ 9.35 (s, 1H), 9.14 (s, 1H), 8.68 - 8.60 (m, 4H), 8.23 (d, *J* = 8.0 Hz, 1H), 8.14 (s.1H), 7.63 -7.60 (m, 1H), 5.50 (d, *J* = 52.0 Hz, 1H), 3.93- 3.53 (m, 5H), 2.38 - 2.15 (m, 2H). ^{19}F NMR: -175.28 ppm. LCMS (m/z): 378.29 (M+H)+. HPLC purity: 98.00%

LMD-040, 041

Scheme:

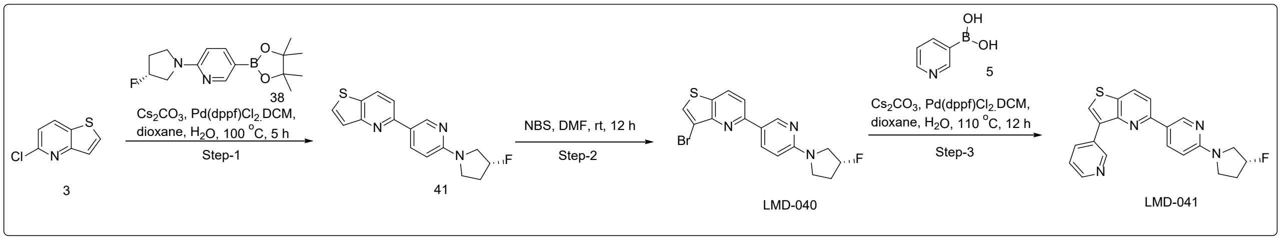

Step-1: Synthesis of (*R*)-5-(6-(3-fluoropyrrolidin-1-yl) pyridin-3-yl) thieno[3,2-*b*] pyridine (41):

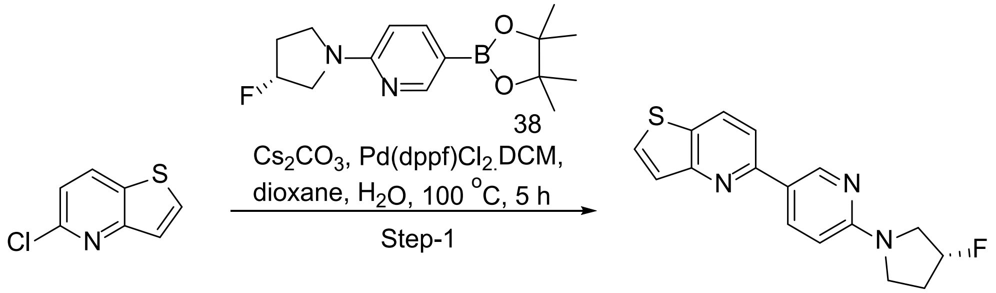

3

41

To a stirred solution of 5-chlorothieno[3,2-*b*]pyridine (2.0 g, 1.0 eq, 11.8 mmol) and (*R*)-2-(3-fluoropyrrolidin-1-yl)-5-(4,4,5,5-tetramethyl-1,3,2-dioxaborolan-2-yl)pyridine (5.1 g, 1.5 eq, 17.75 mmol) in 1,4-dioxane (18 mL) and water (6 mL) was added Cs_2CO_3 (11.6 g, 3.0 eq, 35.4 mmol) at room temperature and degassed with argon gas for 20 min. After that, Pd(dppf)Cl_2.DCM

(704 mg, 0.05 eq, 0.59 mmol,) was added under argon atmosphere and heated at 100 ºC for 5 h. The progress of the reaction was monitored by TLC. After completion, the reaction was filtered through celite pad, washed with EtOAc (100 mL). The filtrate was washed with water (2 x 50 mL) and brine (50 mL). The organic layer was dried over Na_2SO_4, and concentrated under reduced pressure to get crude. The residue was purified on column chromatography using 10% EtOAc/n-hexane as eluent to afford (*R*)-5-(6-(3-fluoropyrrolidin-1-yl)pyridin-3-yl)thieno[3,2-*b*]pyridine (1.5 g, 44%) as an off white solid. LCMS (ESI): m/z = 300.16 $[M+H]^+$.

Step-2: Synthesis of (*R*)-3-bromo-5-(6-(3-fluoropyrrolidin-1-yl) pyridin-3-yl) thieno [3,2-*b*] pyridine (LMD-040):

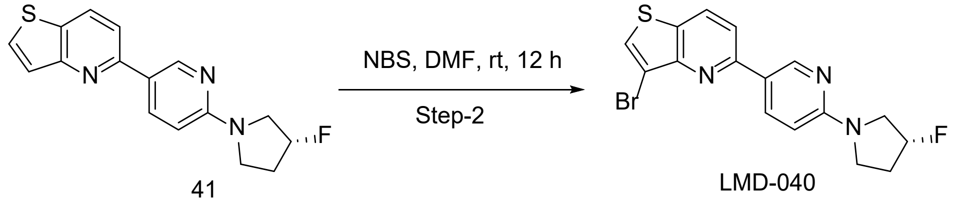

41 LMD-040

To a stirred solution of (*R*)-5-(6-(3-fluoropyrrolidin-1-yl) pyridin-3-yl) thieno[3,2-*b*] pyridine (500 mg, 1.0 eq, 1.67 mmol) in DMF (5 mL) was added NBS (355 mg, 1.2 eq, 2.0 mmol) at room temperature, and stirred for 12 h. The progress of the reaction was monitored by TLC. After completion, the reaction mixture was poured into cold water (20 mL) and extracted with EtOAc (25 mL). The organic layer was washed with brine solution (10 mL), dried over Na_2SO_4 and concentrated under reduced pressure to get crude residue. The residue was purified on column chromatography using 15% EtOAc/*n*-hexane as eluent to afford (*R*)-3-bromo-5-(6-(3-fluoropyrrolidin-1-yl) pyridin-3-yl) thieno[3,2-*b*] pyridine (380 mg, 60%) as a yellow solid. ^{1}H NMR (DMSO-d_6, 400 MHz): δ 8.92 (d, *J* = 2.0 Hz, 1H), 8.62 (d, *J* = 2.0 Hz, 1H), 8.52 (d, *J* = 8.8 Hz, 1H), 8.16 (d, *J* = 5.6 Hz, 1H) , 7.95 (d, *J* = 8.8 Hz, 1H), 7.61 (d, *J* = 5.6 Hz, 1H), 5.41 (d, *J* = 53.6 Hz, 1H), 4.12 - 3.75 (m, 4H), 2.32 - 2.06 (m, 2H). ^{19}F NMR: -176.42 ppm. LCMS (ESI): 378.1 m/z $[M+H]^+$. HPLC purity: 98.09%

Step-3: Synthesis of (*R*)-5-(6-(3-fluoropyrrolidin-1-yl) pyridin-3-yl)-3-(pyridin-3-yl) thieno[3,2-*b*] pyridine (LMD-041):

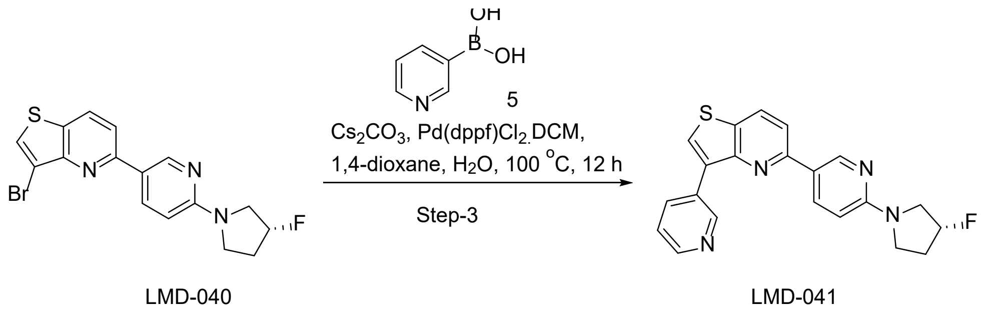

To a stirred solution of (*R*)-3-bromo-5-(6-(3-fluoropyrrolidin-1-yl) pyridin-3-yl) thieno[3,2-*b*] pyridine (200 mg, 1.0 eq, 0.53 mmol) and pyridin-3-ylboronic acid (97 mg, 1.5 eq, 0.79 mmol) in 1,4-dioxane (4 mL), water (1 mL), was added Cs_2CO_3 (518 mg, 3.0 eq, 1.59 mmol) at room temperature and degassed with argon for 20 min. After that, Pd(dppf)Cl_2.DCM (21 mg, 0.05 eq, 0.026 mmol) was added under argon atmosphere, and heated at 100 °C for 12 h. The reaction progress was monitored by TLC. After completion, the reaction was filtered through celite pad, washed with EtOAc (20 mL). The filtrate was washed with water (2 x 10 mL) and brine solution (10 mL). The organic layer was dried over Na_2SO_4, and concentrated under reduced pressure to get crude. The crude was purified on column chromatography using 40% EtOAc/n-hexane as eluent to afford (*R*)-5-(6-(3-fluoropyrrolidin-1-yl) pyridin-3-yl)-3-(pyridin-3-yl) thieno[3,2-*b*] pyridine (89 mg, 40%) as an off white solid. ^{1}H NMR (DMSO-d_6, 400 MHz): δ 8.98 (s, 1H), 8.69 (s, 1H), 8.59 (d, *J* = 4.0 Hz, 1H), 8.51 (d, *J* = 8.4 Hz, 1H), 8.26 (d, *J* = 2.0 Hz, 1H), 8.13 (d, *J* = 5.6 Hz, 1H), 7.99 (d, *J* = 8.4 Hz, 1H), 7.88 (d, *J* = 8.0 Hz, 1H), 7.58 (d, *J* = 5.6 Hz, 1H), 7.52 – 7.49 (m, 1H), 5.27 (d, *J* = 54.0 Hz, 1H), 3.53 - 3.20 (m, 4 H), 2.12 – 1.90 (m, 2H). ^{19}F NMR: -176.69 ppm. LCMS (ESI): m/z = 377.1 $[M+H]^+$. HPLC purity: 99.68%

LMD-063

Scheme:

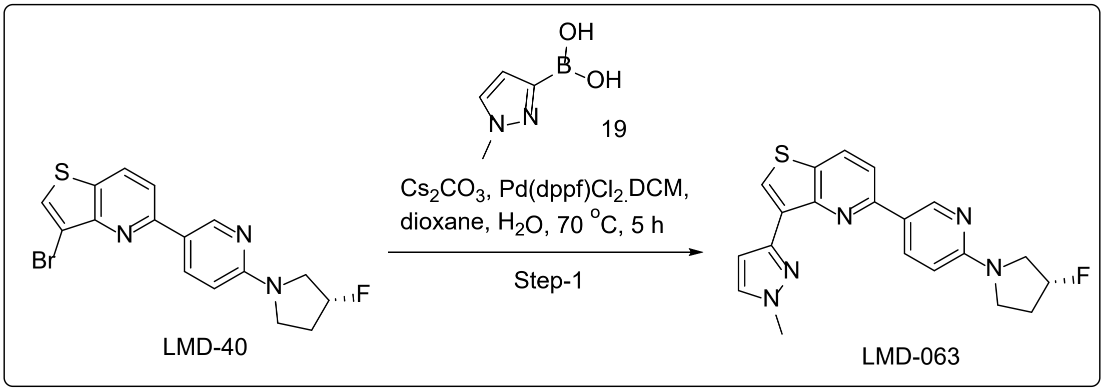

Step-1: Synthesis of (*R*)-5-(6-(3-fluoropyrrolidin-1-yl) pyridin-3-yl)-3-(1-methyl-1*H*-pyrazol-3-yl) thieno[3,2-*b*] pyridine (LMD-063):

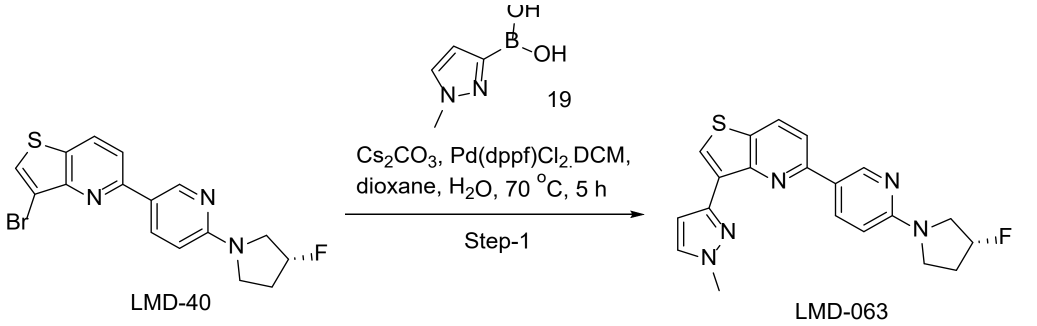

To a stirred solution of 3-bromo-5-{6-[(*R*)-3-fluoro-1-pyrrolidinyl]-3-pyridyl}-1-thia-4-azaindene (150 mg, 1.0 eq, 397 μmol) and (1-methyl-3-pyrazolyl) boranediol (75 mg, 1.5 eq, 595 μmol) in 1,4-dioxane (4 mL), H_2O (1 mL) was added Cs_2CO_3 (388 mg, 3.0 eq, 1.19 mmol) into a seal tube and degassed with argon for 20 min. Then, Pd(dppf)Cl_2.DCM (16.4 mg, 0.05 eq, 19.8 μmol) was added under argon atmosphere and heated at 100 °C for 5 h. The progress of the reaction was monitor by TLC. After completion, filtered through celite pad, washed with EtOAc (10 mL). The filtrate was washed with water (2 x 10 mL) and brine solution (10 mL). The organic layer was dried over Na_2SO_4, and concentrated under reduced pressure to get crude. The crude residue was purified on column chromatography using 40% EtOAc/*n*-hexane as eluent results title compound, which was re-purified on prep-HPLC to obtain (*R*)-5-(6-(3-fluoropyrrolidin-1-yl) pyridin-3-yl)-3-(1-methyl-1*H*-pyrazol-3-yl) thieno[3,2-*b*]pyridine (22 mg, 14%) as an off white solid. ^{1}H NMR (DMSO-*d*$_6$, 400 MHz): δ 8.92 (d, *J* = 2.4 Hz, 1H), 8.49 (d, *J* = 8.8 Hz, 1H), 8.31 (d, *J* = 2.4 Hz, 1H), 8.14 (d, *J* = 5.2 Hz, 1H), 7.79 (d, *J* = 1.6 Hz, 1H), 7.59 (d, *J* = 5.6 Hz, 1H), 6.42 (d, *J* = 2.0 Hz, 1H), 5.37 - 5.24 (d, *J* = 52.4 Hz, 1H), 3.92 (s, 3H), 3.65 - 3.26 (m, 4H), 2.15 - 1.95 (m, 2H). ^{19}F NMR: -176.11 ppm. LCMS (ESI): m/z = 380.1 [M+H]$^+$. HPLC purity: 98.22%

LMD-044

Scheme:

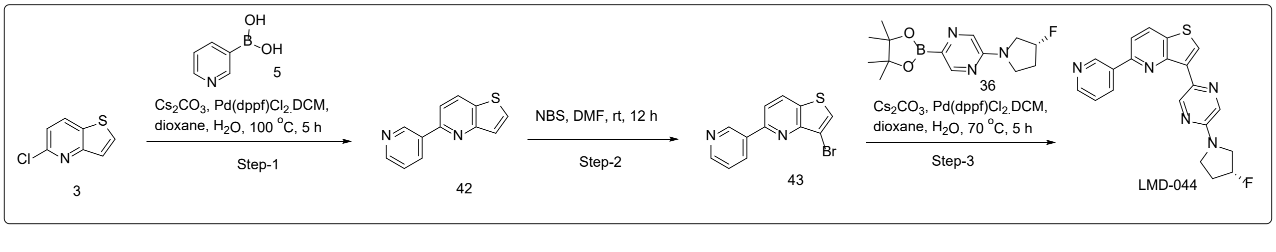

Step-1: Synthesis of 5-(pyridin-3-yl)thieno[3,2-*b*]pyridine (42):

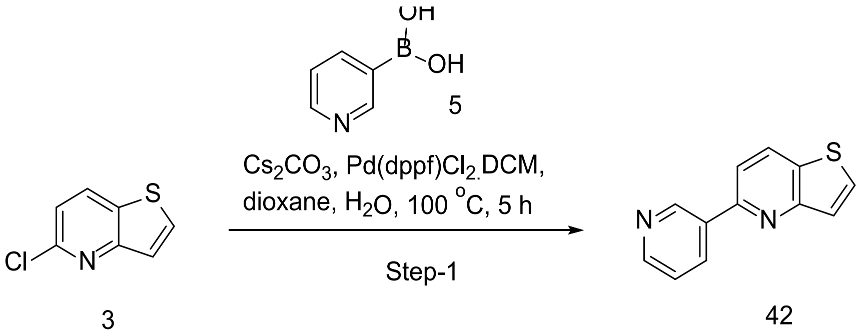

To a stirred solution of 5-chlorothieno[3,2-*b*] pyridine (1.0 g, 1.0 eq, 5.9 mmol) and pyridin-3-ylboronic acid (725 mg, 1.0 eq, 5.9 mmol) in 1,4-Dioxane (9 mL), H_2O (3 mL) was added cesium carbonate (5.76 g, 3.0 eq, 17.7 mmol) in a sealed tube and degassed with argon for 10 min. After that, $Pd(dppf)Cl_2$.DCM (240 mg, 0.05 eq, 0.295 mmol) under argon atmosphere and heated at 100 °C for 5 h. The reaction progress was monitored by TLC. After completion of the reaction, filtered the through celite pad, and washed with EtOAc (50 mL). The filtrate was washed with water (2 x 50 mL) and brine (50 mL). The organic layer was dried over Na_2SO_4, and concentrated under reduced pressure to get crude. The residue was purified on column chromatography using EtOAc/*n*-heptane as eluent afford 5-(pyridin-3-yl)thieno[3,2-*b*]pyridine (1.0 g, 79.91%) as an off white solid. ^{1}H NMR (DMSO-d_6, 400 MHz): δ 9.35 (s, 1H), 8.66 - 8.62 (m, 2H), 8.53 - 8.50 (m, 1H), 8.22 (d, J = 5.6 Hz, 1H), 8.05 (d, J = 8.4 Hz, 1H), 7.66 (d, J = 5.2 Hz, 1H), 7.56 - 7.53 (m, 1H). LCMS (m/z): 213.27 (M+H).

Step-2: Synthesis of 3-bromo-5-(pyridin-3-yl)thieno[3,2-*b*]pyridine (43):

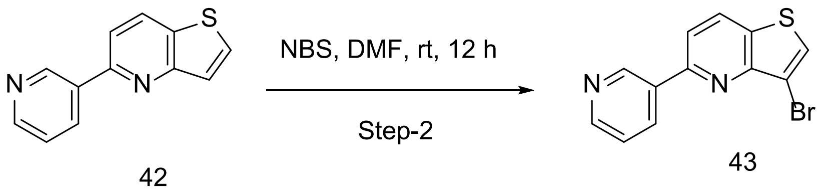

To a stirred solution of 5-(3-pyridyl)-1-thia-4-azaindene (300 mg, 1.0 eq, 1.41 mmol) in DMF (10 mL) was added NBS (252 mg, 1.0 eq, 1.41 mmol) portion wise at room temperature and stirred for 12 h. The progress of the reaction mixture was monitored by TLC. After completion of the reaction, ice cold water was added, solid was obtained. The obtained solid was filtered and washed with water and dried under vacuum to get crude. The crude product was purified by flash column chromatography, over silica gel using 10% EtOAc in hexane as eluent to afford 3-bromo-5-

(pyridin-3-yl)thieno[3,2-*b*]pyridine (200 mg, 48.6%) as an off white solid. ^{1}H NMR (DMSO-d_6, 400 MHz): δ 9.41 (s, 1H), 8.71 (t, J = 5.6 Hz, 2H), 8.59 (d, J = 8.0 Hz, 1H), 8.44 (s, 1H), 8.18 (d, J = 8.4 Hz, 1H), 7.61 - 7.58 (m, 1H). LCMS (m/z): 213.27 [M+H]$^+$.

Step-3: Synthesis (*R*)-3-(5-(3-fluoropyrrolidin-1-yl) pyrazin-2-yl)-5-(pyridin-3-yl) thieno[3,2-*b*]pyridine (LMD-044):

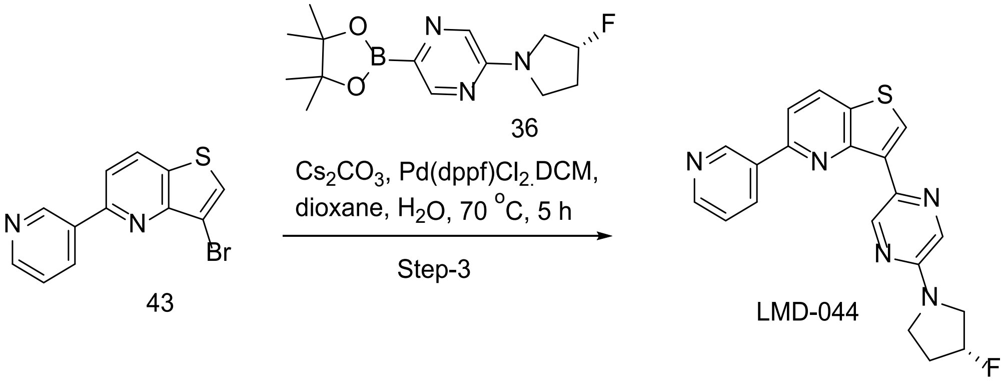

To a stirred solution of 3-bromo-5-(pyridin-3-yl)thieno[3,2-*b*]pyridine (200 mg, 1.0 eq, 0.69 mmol) and (*R*)-2-(3-fluoropyrrolidin-1-yl)-5-(4,4,5,5-tetramethyl-1,3,2-dioxaborolan-2-yl)pyrazine (403 mg, 2.0 eq, 1.4 mmol) in dioxane (3 mL), H_2O (0.6 mL) was added cesium carbonate (671 mg, 3.0 e q, 2.06 mmol) into a sealed tube and degassed with argon for 10 min. After that, Pd(dppf)Cl_2.DCM (28 mg, 0.05 eq, 0.03 mmol) was added under argon atmosphere and heated at 100 °C for 12 h. The reaction progress was monitored by TLC. After completion, filtered through celite pad and washed with EtOAc (20 mL). The filtrate was washed with water (2 x 10 mL) and brine (10 mL). The organic layer was dried over Na_2SO_4, and concentrated under reduced pressure to get crude. The residue was purified on column chromatography using EtOAc/*n*-heptane as eluent to get the desired compound, which was re-purified by prep-HPLC to afford (*R*)-3-(5-(3-fluoropyrrolidin-1-yl) pyrazin-2-yl)-5-(pyridin-3-yl) thieno[3,2-*b*]pyridine (40 mg, 15.43%) as an off white solid. ^{1}H NMR (DMSO-d_6, 400 MHz): δ 9.73 (s, 1H), 9.45 (s, 1H), 8.72 (t, J = 5.2 Hz, 2H), 8.61 (d, J = 8.0 Hz, 2H), 8.17 (t, J = 8.8 Hz, 2H), 7.64 - 7.67 (m, 1H), 5.52 (d, J = 53.0 Hz, 1H), 3.94 - 3.77 (m, 3H), 3.58 - 3.57 (m, 1H), 2.28 - 2.33 (m, 2H). ^{19}F NMR: -175.13 ppm. LCMS (m/z): 378.1 (M+H). HPLC purity: 99.13%

LMD-016 & 017

Scheme:

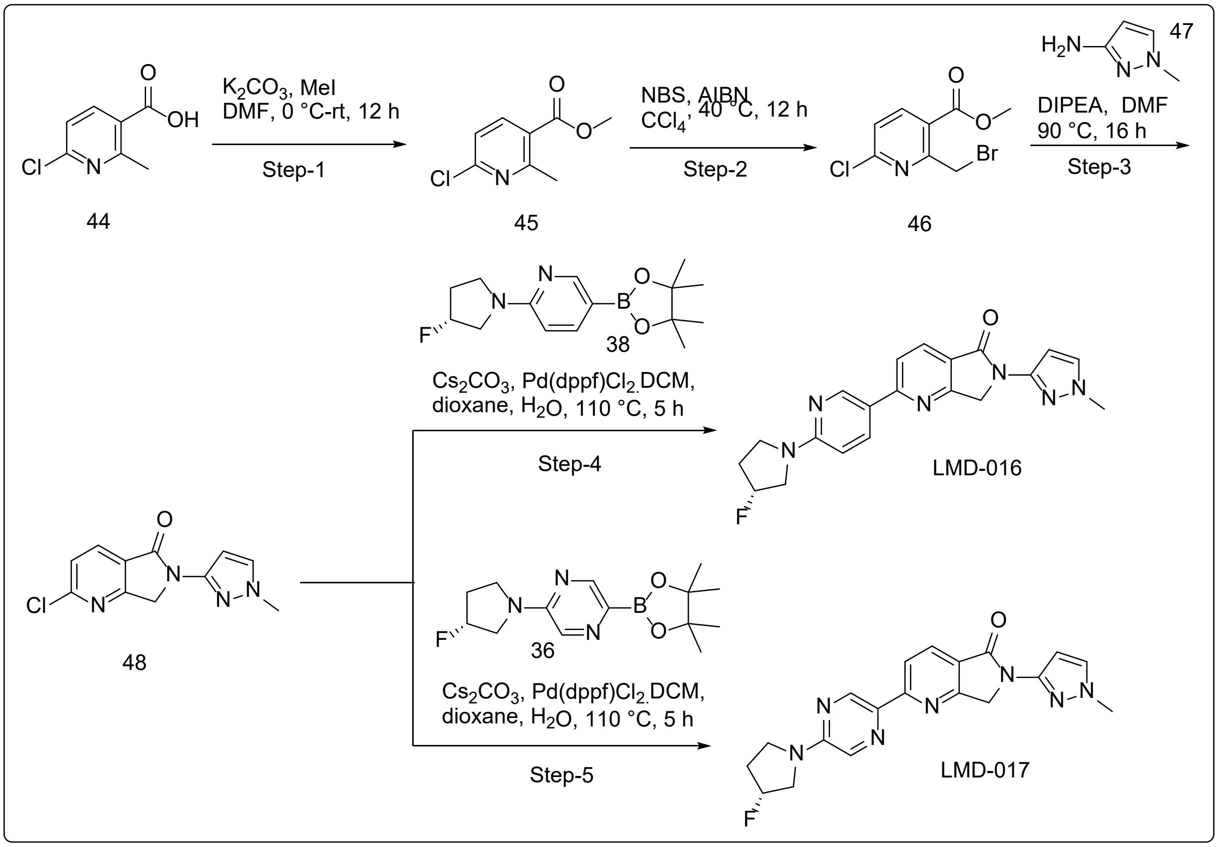

Step-1: Synthesis of methyl 6-chloro-2-methylnicotinate (45):

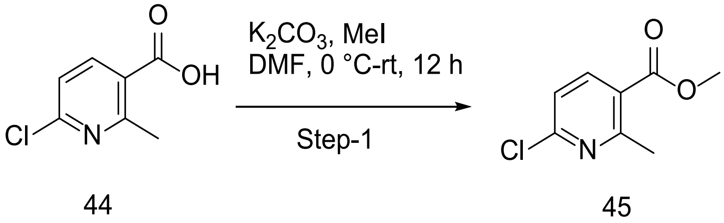

To a 0 ºC cooled solution of 6-chloro-2-methylnicotinic acid (5 g, 1.0 eq, 29.1 mmol) in DMF (60 mL) was added K_2CO_3 (10.0 g, 2.5 eq, 72.8 mmol) followed by CH_3I (2.5 mL, 2.0 eq, 58.2 mmol), and allowed to stir at 25 ºC for 12 h. The progress of the reaction was monitored by TLC. After completion of reaction, cooled to room temperature, diluted with H_2O (50 mL) and extracted with EtOAc (2 x 100 mL). The combined organic layers were washed with brine (2 x 50 mL), dried over anhydrous Na_2SO_4, and concentrated under reduced pressure to afford methyl 6-chloro-2-methyl-pyridine-3-carboxylate (3 g, 60%) as a brown oil. This was used in the next step without further purification. MS (ESI): m/z = 186.1 $[M+H]^+$;

Step-2: Synthesis of methyl 2-(bromomethyl)-6-chloropyridine-3-carboxylate (46):

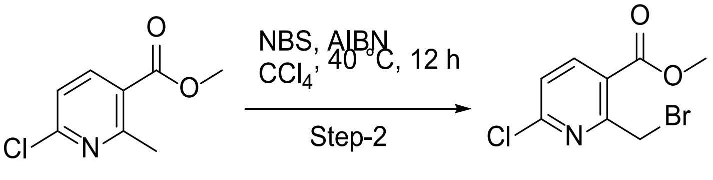

45 46

To a stirred solution of methyl 6-chloro-2-methylpyridine-3-carboxylate (2.0 g, 1.0 eq, 10.8 mmol) in CCl_4 (20 mL) was added NBS (2.88 g, 1.5 eq, 16.2 mmol) followed by AIBN (177 mg, 0.1 eq, 1.08 mmol) under nitrogen atmosphere. The reaction mixture was stirred at 40 °C 12 h. The progress of the reaction was monitored by TLC. The solids were filtered and filtrate was concentrated under reduced pressure to get crude residue. The residue was purified by flash column chromatography using 50% EtOAc/hexane as eluent to afford methyl 2-(bromomethyl)-6-chloropyridine-3-carboxylate (1.2 g, 42%) as a brown oil. MS (ESI): m/z = 264.2 $[M+H]^+$

Step-3: Synthesis of 2-chloro-6-(1-methyl-1*H*-pyrazol-3-yl)-6,7-dihydro-5*H*-pyrrolo[3,4-*b*] pyridin-5-one (48):

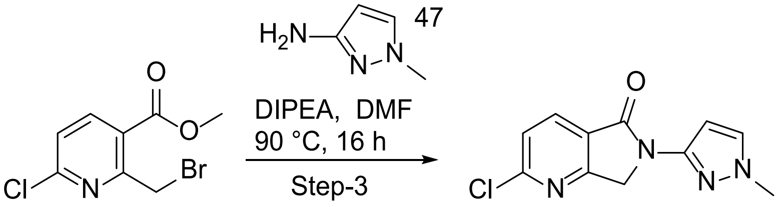

46 48

To a stirred solution of 1-methyl-1*H*-pyrazol-3-amine (1.1 g, 3.0 eq, 11.3 mmol) in DMF (10 mL) was added methyl 6-bromo-2-(chloromethyl) pyridine-3-carboxylate (1 g, 1.0 eq, 3.78 mmol) followed by *N*-ethyldiisopropylamine (4 mL, 6.0 eq, 22.7 mmol) at room temperature and stirred at 90 °C for 12 h. The progress of reaction was monitor by TLC. After completion, the reaction was cooled to room temperature, concentrated under reduce pressure to get crude residue. The crude was purified by flash column chromatography using 0-50% EtOAc/hexane as eluent to afford 2-chloro-6-(1-methyl-1*H*-pyrazol-3-yl)-6,7-dihydro-5*H*-pyrrolo[3,4-*b*] pyridin-5-one (0.6 g, 58%) as a yellow solid. MS (ESI): m/z = 249.0 $[M+H]^+$

Step-4: Synthesis of (*R*)-2-(6-(3-fluoropyrrolidin-1-yl) pyridin-3-yl)-6-(1-methyl-1*H*-pyrazol-3-yl)-6,7-dihydro-5*H*-pyrrolo[3,4-*b*] pyridin-5-one (LMD-016):

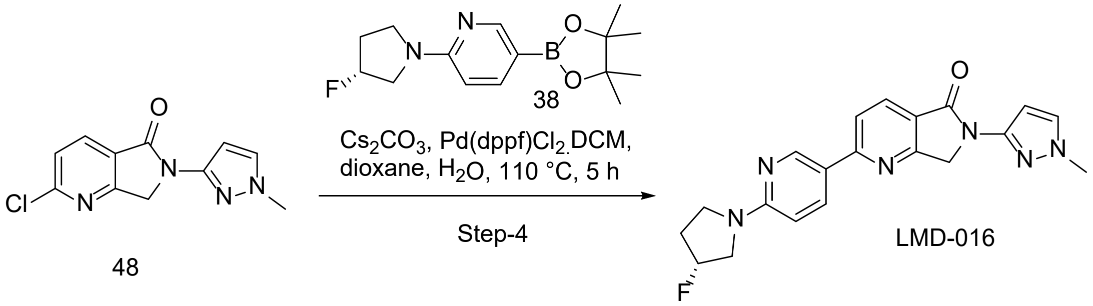

To a stirred solution of 5-chloro-2-(1-methyl-3-pyrazolyl)-2,4-diaza-1-indanone (0.60 g, 1.0 eq, 2.41 mmol) and 2-{6-[(*R*)-3-fluoro-1-pyrrolidinyl]-3-pyridyl}-4,4,5,5-tetramethyl-1,3,2-dioxaborolane (1.41 g, 1.2 eq, 2.9 mmol) in 1,4-dioxane (9 mL),water (3 mL) was added Cs_2CO_3 (2.36 g, 3.0 eq, 7.24 mmol) at room temperature and purged with argon gas for 20 min. After that, Pd(dppf)Cl_2.DCM (197 mg, 0.1 eq, 241 μmol) was added under argon atmosphere and heated at 110 °C for 12 h. The reaction progress of was monitored by TLC. After completion of the reaction, the reaction was filtered through celite pad and washed with EtOAc (30 mL). The filtrate was washed with water (20 mL) and brine (10 mL). The organic layer was dried over Na_2SO_4, and concentrated under reduced pressure to get crude. The residue was purified by silica gel column chromatography using 40% EtOAc/*n*-hexane as eluent to afford title compound, which was re-purified by using prep-HPLC to afford (*R*)-2-(6-(3-fluoropyrrolidin-1-yl) pyridin-3-yl)-6-(1-methyl-1*H*-pyrazol-3-yl)-6,7-dihydro-5*H*-pyrrolo[3,4-*b*] pyridin-5-one (15 mg, 1.6%) as a light brown solid. ^{1}H NMR (DMSO-d_6, 400 MHz): δ 8.96 (s, 1H), 8.36-8.33 (m, 1H), 8.12 (d, *J* = 8.0 Hz 1H), 8.03 (d, *J* = 8.4 Hz, 1H), 7.70 (d, *J* = 2.4 Hz, 1H), 6.76 (d, *J* = 2.0 Hz, 1H), 6.66 (d, *J* = 8.8 Hz, 1H), 5.45 (d, *J* = 48.0 Hz, 1H), 4.94 (s, 2H), 3.83 (s, 3H), 3.80 - 3.49 (m, 4H), 2.26 - 2.15 (m, 2H). ^{19}F NMR: -175.10 ppm. MS (ESI): m/z = 379.1 $[M+H]^+$. HPLC purity: 96.6%

Step-5: Synthesis of (*R*)-2-(5-(3-fluoropyrrolidin-1-yl) pyrazin-2-yl)-6-(1-methyl-1*H*-pyrazol-3-yl)-6,7-dihydro-5*H*-pyrrolo[3,4-*b*] pyridin-5-one (LMD-017):

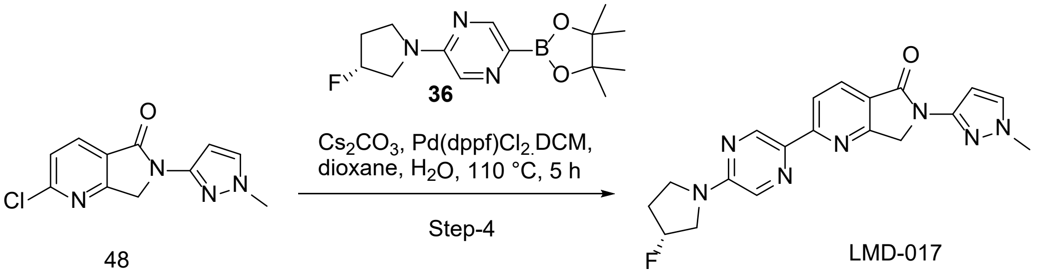

To a solution of 5-chloro-2-(1-methyl-3-pyrazolyl)-2,4-diaza-1-indanone (600 mg, 1.0 eq, 2.41 mmol) and {5-[(*R*)-3-fluoro-1-pyrrolidinyl]-2-pyrazinyl}borane diol (764 mg, 1.5 eq, 3.62 mmol) in 1,4-dioxane (9 mL):water (2 mL) was added Cs_2CO_3 (2.36 g, 3.0 eq, 7.24 mmol) at room temperature and purged with argon for 20 min. After that, $Pd(dppf)Cl_2$.DCM (197 mg, 0.1 eq, 241 µmol) was added under argon atmosphere and heated at 110 °C for 12 h. The reaction progress was monitored by TLC. After completion of the reaction, the reaction was filtered through celite pad and washed with EtOAc (30 mL). The filtrate was washed with water (20 mL) and brine (10 mL). The organic layer was dried over Na_2SO_4, and concentrated under reduced pressure to get crude. The residue was purified on column chromatography using 25% EtOAc/hexane to afford (*R*)-2-(5-(3-fluoropyrrolidin-1-yl) pyrazin-2-yl)-6-(1-methyl-1*H*-pyrazol-3-yl)-6,7-dihydro-5*H*-pyrrolo[3,4-*b*] pyridin-5-one (22 mg, 2%) as an off white solid. ^{1}H NMR (DMSO-*d*6, 400 MHz): δ 9.11 (s, 1H), 8.29 (d, *J* = 8.4 Hz, 1H), 8.21 - 8.18 (m, 2H), 7.72 (d, *J* = 2.0 Hz, 1H), 6.77 (d, *J* = 2.0 Hz, 1H), 5.55 (d, *J* = 48.0 Hz, 1H), 4.98 (s, 2H), 3.83 (s, 3H), 3.80 - 3.49 (m, 4H), 2.26 - 2.15 (m, 2H). MS (ESI): m/z = 380.27.0 $[M+H]^+$. ^{19}F NMR: -175.4 ppm. HPLC purity: 96.63%

LMD-019, 064

Scheme:

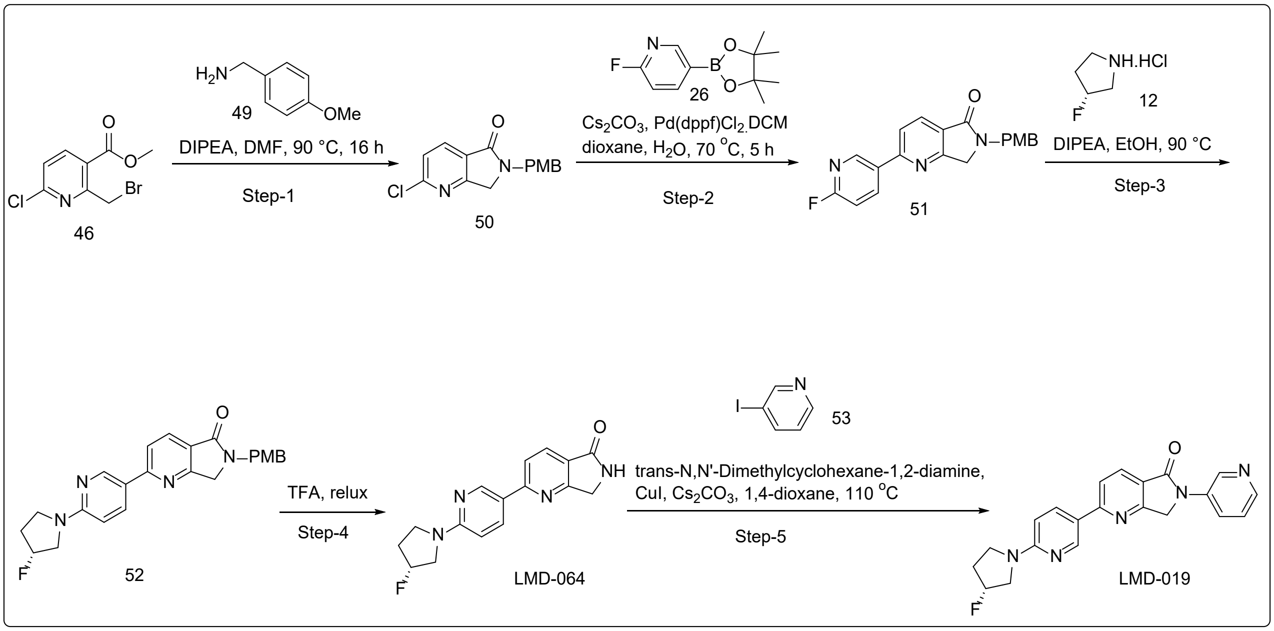

Step-1: Synthesis of 2-chloro-6-(4-methoxybenzyl)-6,7-dihydro-5*H*-pyrrolo[3,4-*b*] pyridin-5-one (50):

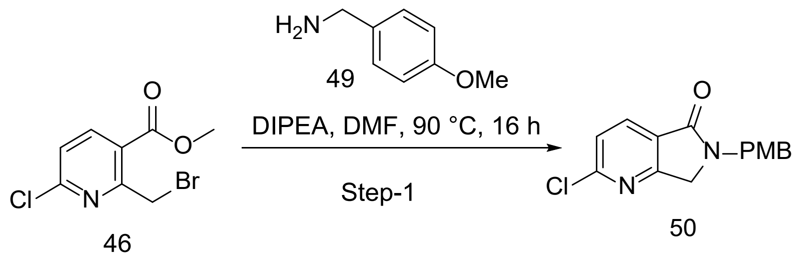

To a solution of [(*p*-methoxyphenyl) methyl] amine (13.0 g, 2.5 eq, 94.5 mmol) in DMF (100 mL) was added DIPEA (32.6 mL, 5.0 eq, 189 mmol) followed by methyl 2-(bromomethyl)-6-chloronicotinate (10.0 g, 1.0 eq, 37.8 mmol) at room temperature and stirred at 40 °C for 16 h. The progress of the reaction was monitored by TLC. After completion, the reaction was diluted with H_2O (100 mL) and extracted with EtOAc (2 x 100 mL). The organic layer was concentrated under reduced pressure to get crude. The crude residue was purified by flash column chromatography on silica gel using 25% EtOAc/*n*-hexane as eluent to afford 2-chloro-6-(4-methoxybenzyl)-6,7-dihydro-5*H*-pyrrolo[3,4-*b*] pyridin-5-one (3.0 g, 27.48%) as a yellow solid. ^{1}H NMR (DMSO-d_6, 400 MHz): δ 8.15 (d, *J* = 8.0 Hz, 1H), 7.64 (d, *J* = 8.0 Hz, 1H), 7.23 (d, *J* = 8.4 Hz, 2H), 7.07 (d, *J* = 8.4 Hz, 2H), 4.66 (s, 2H), 4.41 (s, 2H), 3.72 (s, 3H).

Step-2: Synthesis of 5-(6-fluoro-3-pyridyl)-2-[(*p*-methoxyphenyl) methyl]-2,4-diaza-1-indanone (51):

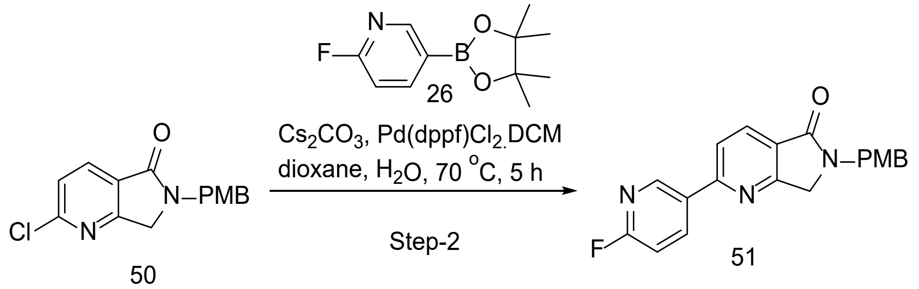

To a solution of 2-chloro-6-(4-methoxybenzyl)-6,7-dihydro-5*H*-pyrrolo[3,4-*b*] pyridin-5-one (700 mg, 1.0 eq, 2.42 mmol) and (6-fluoro-3-pyridyl) butanediol (410 mg, 1.2 eq, 2.91 mmol) in 1,4-dioxane (9 mL), water (3 mL) was added Cs_2CO_3 (728 mg, 3.0 eq, 7.27 mmol) at room temperature and purged with argon for 10 min. After that, Pd(dppf)Cl_2.DCM (198 mg, 0.1 eq, 242 µmol) was added under argon atmosphere and heated at 110 °C for 12 h. The reaction progress was monitored by TLC. After completion of the reaction, the reaction filtered through celite pad and washed with EtOAc (20 mL). The filtrate was washed with water (20 mL) followed by brine solution (20 mL). The organic layer was dried over Na_2SO_4, and concentrated under reduced pressure to get crude

residue. The residue was purified by silica gel column chromatography using 25% EtOAc/hexane as eluent to afford 5-(6-fluoro-3-pyridyl)-2-[(*p*-methoxyphenyl) methyl]-2,4-diaza-1-indanone (350 mg, 41%) as an off white solid. LCMS(ESI); *m/z* = 350.37 [M+H]$^+$

Step-3: Synthesis of (*R*)-2-(6-(3-fluoropyrrolidin-1-yl) pyridin-3-yl)-6-(4-methoxybenzyl)-6,7-dihydro-5*H*-pyrrolo[3,4-*b*] pyridin-5-one (52):

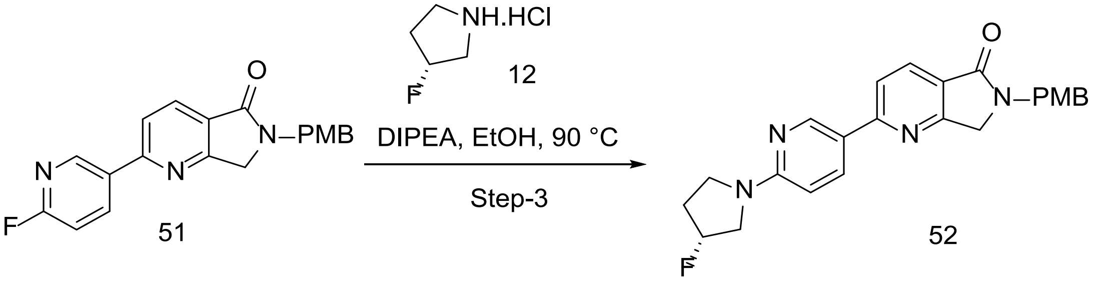

To a solution of 5-(6-fluoro-3-pyridyl)-2-[(*p*-methoxyphenyl) methyl]-2,4-diaza-1-indanone (350 mg, 1.0 eq, 1 mmol) and 3-fluoropyrrolidine-hydrogen chloride (1/1) (151 mg, 1.2 eq, 1.2 mmol), were charged into a sealed tube was added EtOH (12 mL), followed by *N*-ethyldiisopropylamine (388 mg, 3.0 eq, 3.01 mmol) at room temperature and heated at 100 °C for 12 h. The reaction progress was monitored by TLC. After completion of the reaction, the solvent was removed under reduced pressure and the residue was taken up in dichloromethane (20 mL), washed with saturated ammonium chloride solution (20 mL) and water (20 mL), dried over Na_2SO_4, and concentrated under reduced pressure to get crude. The residue was purified by column chromatography using 10% EtOAc/hexane as eluent to afford (*R*)-2-(6-(3-fluoropyrrolidin-1-yl) pyridin-3-yl)-6-(4-methoxybenzyl)-6,7-dihydro-5*H*-pyrrolo[3,4-*b*] pyridin-5-one (250 mg, 59%) as an off white solid.

Step-3: Synthesis of (*R*)-2-(6-(3-fluoropyrrolidin-1-yl) pyridin-3-yl)-6,7-dihydro-5H-pyrrolo[3,4-*b*] pyridin-5-one (LMD-064):

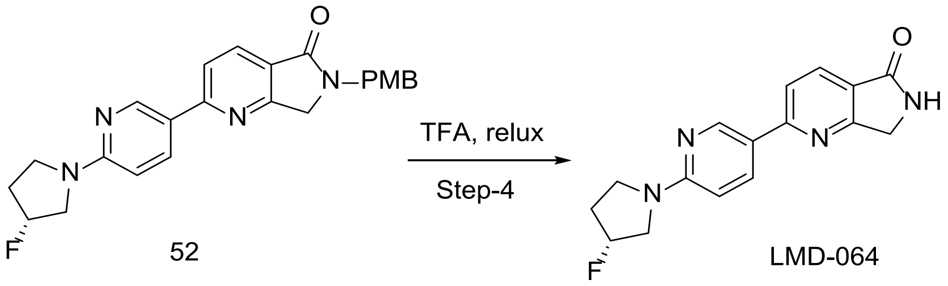

To a solution of (*R*)-2-(6-(3-fluoropyrrolidin-1-yl) pyridin-3-yl)-6-(4-methoxybenzyl)-6,7-dihydro-5*H*-pyrrolo[3,4-*b*] pyridin-5-one (800 mg, 1.0 eq, 1.91 mmol) in TFA (2.18 g, 10 eq, 19.1 mmol) was heated to 120 ºC for 24 h in a sealed tube. The reaction progress was monitored by TLC and LCMS. After that, the reaction mixture was concentrated under reduced pressure, co-distilled with DCM (20 mL) results crude product. The crude product was purified by column chromatography using 10% MeOH/DCM as eluent to afford (*R*)-2-(6-(3-fluoropyrrolidin-1-yl) pyridin-3-yl)-6,7-dihydro-5*H*-pyrrolo[3,4-*b*] pyridin-5-one (350 mg, 61%) as an off white solid. ^{1}H NMR (DMSO-d_6, 400 MHz): δ 8.93 (d, *J* = 2.0 Hz, 1H), 8.68 (s, 1H), 8.32 - 8.29 (dd, *J* = 8.8 Hz, 2.4 Hz, 1H), 8.04 (d, *J* = 8.4 Hz, 1H), 7.98 (d, *J* = 8.4 Hz, 1H), 6.65 (d, *J* = 8.4 Hz, 1H), 5.48 (d, *J* = 53.2 Hz, 1H), 4.45 (s, 2H), 3.86 - 3.60 (m, 3H), 3.53 -3.44 (m, 1H), 2.33 - 2.10 (m, 2H). ^{19}F NMR: -175.06 ppm. MS (ESI): m/z = 299.23 $[M+H]^+$. HPLC purity: 97.88%

Step-5: Synthesis of (*R*)-2-(6-(3-fluoropyrrolidin-1-yl) pyridin-3-yl)-6-(pyridin-3-yl)-6,7-dihydro-5*H*-pyrrolo[3,4-*b*] pyridin-5-one (LMD-019):

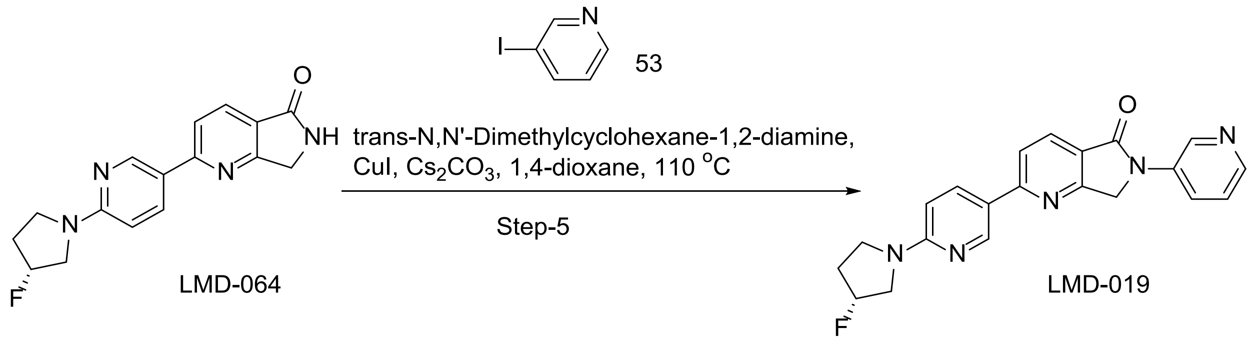

To a stirred solution of 5-{6-[(*R*)-3-fluoro-1-pyrrolidinyl]-3-pyridyl}-2,4-diaza-1-indanone (100 mg, 1.0 eq, 335 µmol), 3-iodopyridine (103 mg, 1.5 eq, 503 µmol) in 1,4-Dioxne (2 mL) at room temperature was added Cs_2CO_3 (328 mg, 3.0 eq, 1.01 mmol), and degassed with argon for 10 min. After that, CuI (13.2 mg, 0.2 eq, 0.07 mmol) was added followed by trans-*N*,*N'*-Dimethylcyclohexane-1,2-diamine (9.5 mg, 0.2 eq, 0.07 mol), under argon atmosphere and stirred at 110 °C for 18 h. The progress of the reaction was monitored by TLC. After completion of the reaction, filtered through celite pad and washed with EtOAc (10 mL). The filtrate was washed with water (10 mL) and brine (10 mL). The organic layer was dried over Na_2SO_4, and concentrated under reduced pressure to get crude. The residue was purified by silica gel column chromatography using EtOAc/*n*-hexane as eluent to afford title compound, which was re-purified by using prep-HPLC to afford (*R*)-2-(6-(3-fluoropyrrolidin-1-yl) pyridin-3-yl)-6-(pyridin-3-yl)-6,7-dihydro-5*H*-

pyrrolo[3,4-*b*] pyridin-5-one (2.3 mg, 1.83%) as an off white solid. ^{1}H NMR (DMSO-d_6, 400 MHz): δ 9.15 (d, *J* = 2.4 Hz, 1H), 8.99 (d, *J* = 2.0 Hz, 1H), 8.40 - 8.34 (m, 3H), 8.17 (d, *J* = 8.4 Hz, 1H), 8.07 (d, *J* = 8.0 Hz, 1H), 7.51 - 7.48 (m, 1H), 6.67 (d, *J* = 8.8 Hz, 1H), 5.48 (d, *J* = 52.0 Hz, 1H), 5.15 (s, 2H), 3.87 - 3.62 (m, 3H), 3.54 - 3.43 (m, 1H), 2.27 - 2.16 (m, 2H). ^{19}F NMR: -175.12 ppm. MS (ESI): m/z = 376.3 [M+H]$^+$. HPLC purity: 98.62%

LMD-022

Scheme:

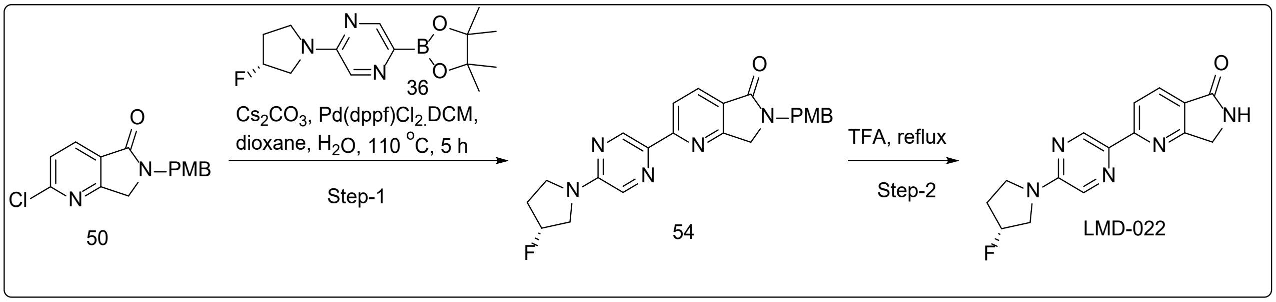

Step-1: Synthesis of (*R*)-2-(5-(3-fluoropyrrolidin-1-yl)pyrazin-2-yl)-6-(4-methoxybenzyl)-6,7-dihydro-5*H*-pyrrolo[3,4-*b*]pyridin-5-one (54):

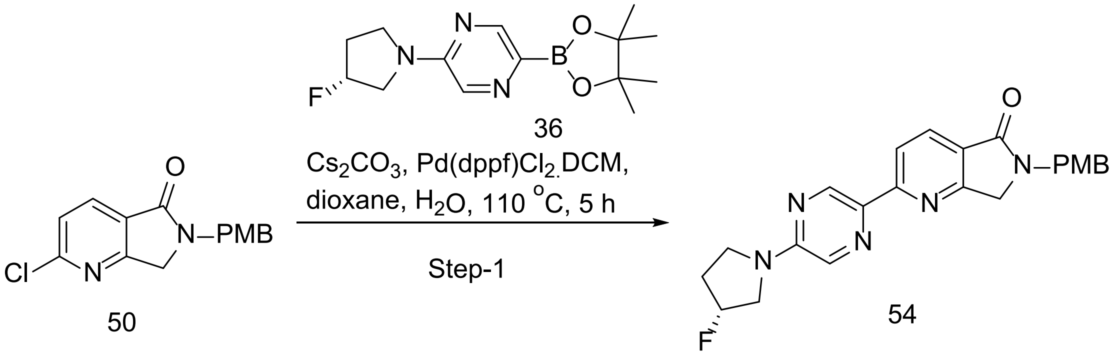

To a solution of 5-chloro-2-[(*p*-methoxyphenyl)methyl]-2,4-diaza-1-indanone (1 g, 1.0 eq, 3.46 mmol) and {5-[(*R*)-3-fluoro-1-pyrrolidinyl]-2-pyrazinyl}boranediol (2.44 g, 2.0 eq, 6.93 mmol) in 1,4-Dioxane (9 mL), water (3 mL) was added Cs_2CO_3 (3.39 g, 3.0 eq, 10.4 mmol) into a sealed tube at room temperature and purged with argon gas for 20 min.. Then, Pd(dppf)Cl_2.DCM (283 mg, 0.1 eq., 346 µmol) was added under argon atmosphere and heated at 110 ºC for 12 h. The reaction progress was monitored by TLC. After completion of the reaction, the reaction was filtered through celite pad and washed with EtOAc (50 mL). The filtrate was washed with water (40 mL) and brine (20 mL). The organic layer was dried over Na_2SO_4, and concentrated under reduced pressure to afford (*R*)-2-(5-(3-fluoropyrrolidin-1-yl) pyrazin-2-yl)-6-(4-methoxybenzyl)-

6,7-dihydro-5*H*-pyrrolo[3,4-*b*] pyridin-5-one (1.2 g, crude) as crude as a brown solid. This was used in the next step without any further purification.
LCMS (ESI): 420 m/z $[M+H]^+$.

Step-2: Synthesis of (*R*)-2-(5-(3-fluoropyrrolidin-1-yl)pyrazin-2-yl)-6,7-dihydro-5*H*-pyrrolo[3,4-*b*]pyridin-5-one (LMD-022):

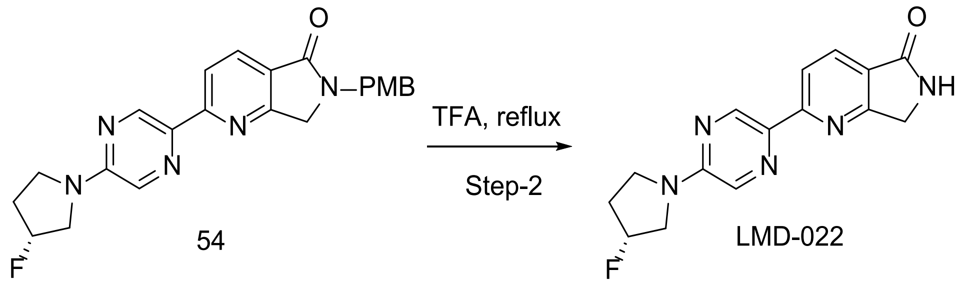

A stirred solution of (*R*)-2-(5-(3-fluoropyrrolidin-1-yl)pyrazin-2-yl)-6-(4-methoxybenzyl)-6,7-dihydro-5*H*-pyrrolo[3,4-*b*]pyridin-5-one (200 mg, 1.0 eq, 477 μmol) in TFA (2 mL) was heated at 120 °C for 18 h. The reaction progress was monitored by LCMS. After that, the reaction mixture was concentrated under reduced pressure to get crude. The crude residue was purified by prep-HPLC purification to afford (*R*)-2-(5-(3-fluoropyrrolidin-1-yl)pyrazin-2-yl)-6,7-dihydro-5*H*-pyrrolo[3,4-*b*]pyridin-5-one (11 mg, 7%) as a white solid. ^{1}H NMR (DMSO-d_6, 400 MHz): δ 9.06 (s, 1H),), 8.75 (s, 1H), 8.24 (d, *J* = 8.0 Hz, 1H), 8.15 (s, 1H), 8.11(d, *J* = 8.0 Hz, 1H), 5.50 (d, *J* = 48.0 Hz, 1H), 4.47 (s, 2H), 3.93 - 3.66 (m, 3H), 3.60 - 3.53 (m, 1H), 2.37 - 2.16 (m, 2H).
LCMS (ESI): 300.17 m/z $[M+H]^+$. ^{19}F NMR: -175.4 ppm. HPLC purity: 99.02%

LMD-023

Scheme:

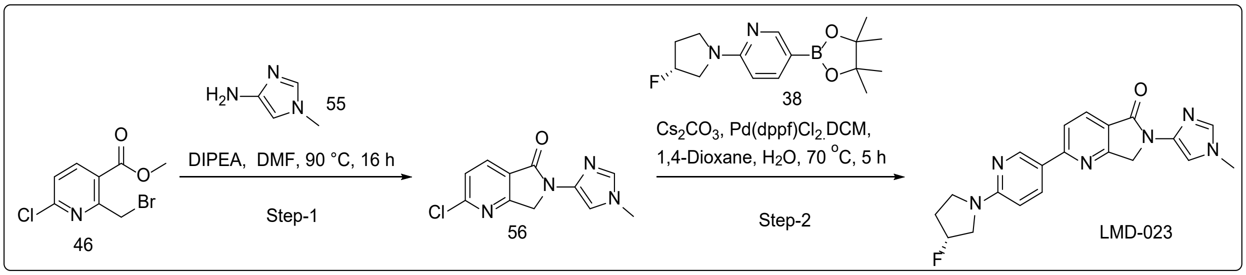

Step-1: Synthesis of 2-chloro-6-(1-methyl-1*H*-imidazol-4-yl)-6,7-dihydro-5*H*-pyrrolo[3,4-*b*] pyridin-5-one (56):

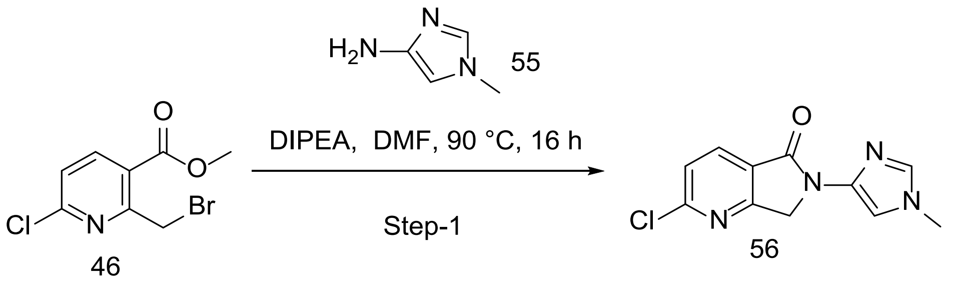

To a solution of 1-methyl-1*H*-imidazol-4-amine (1.1 g, 2.0 eq, 11.3 mmol) in DMF (11 mL) was added DIPEA (5.86 mL, 6.0 eq, 34 mmol), followed by addition of methyl 2-(bromomethyl)-6-chloronicotinate (1.5 g, 1.0 eq, 5.67 mmol) at room temperature and heated at 90 °C for 16 h. The progress of the reaction was monitored by TLC. Then, the reaction mixture was diluted with EtOAc (30 mL), washed with H_2O (20 mL) and brine (10 mL). The organic layer was dried over Na_2SO_4 and concentrated to get 2-chloro-6-(1-methyl-1*H*-imidazol-4-yl)-6,7-dihydro-5*H*-pyrrolo[3,4-*b*] pyridin-5-one (0.6 g, crude) as a brown sticky solid. This was used in the next step without any further purification. LCMS (m/z): 249.24 (M+H).

Step:2: Synthesis of (*R*)-2-(6-(3-fluoropyrrolidin-1-yl) pyridin-3-yl)-6-(1-methyl-1*H*-imidazol-4-yl)-6,7-dihydro-5*H*-pyrrolo[3,4-*b*] pyridin-5-one (LMD-023):

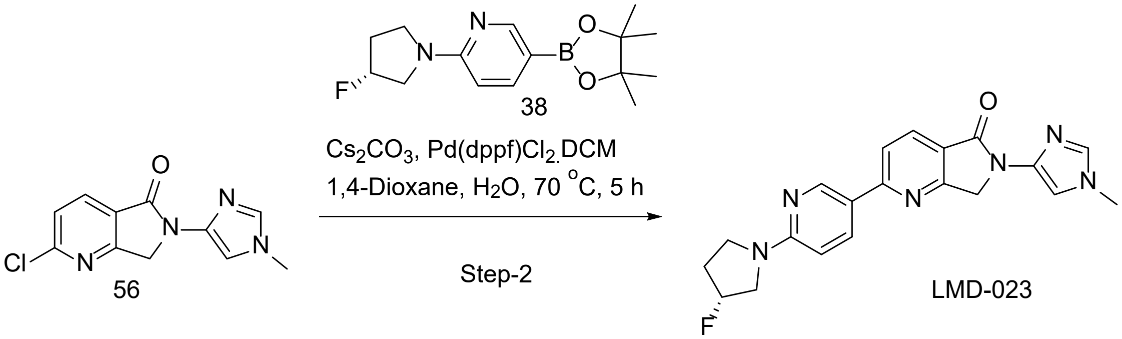

To a stirred solution of 5-chloro-2-(1-methyl-4-imidazolyl)-2,4-diaza-1-indanone (600 mg, 1.0 eq, 2.4 mmol) and {6-[(*R*)-3-fluoro-1-pyrrolidinyl]-3-pyridyl}boranediol (760 mg, 1.5 eq, 3.62 mmol) in 1,4-dioxane (9 mL):water (3 mL) was added Cs_2CO_3 (2.36 g, 3.0 eq., 7.24 mmol) at room temperature and purged with argon for 20 min. After that, Pd(dppf)Cl_2.DCM (197 mg, 0.1 eq., 241 μmol) was added under argon atmosphere and heated at 100 °C for 5 h. The reaction progress was monitored by TLC. After completion of the reaction, the reaction was filtered through celite pad and washed with EtOAc (30 mL). The filtrate was washed with H_2O (20 mL) and brine solution (10 mL). The organic layer was dried over Na_2SO_4, and concentrated under reduced pressure to

get the crude. The crude residue was purified by Prep-HPLC to afford (*R*)-2-(6-(3-fluoropyrrolidin-1-yl)pyridin-3-yl)-6-(1-methyl-1*H*-imidazol-4-yl)-6,7-dihydro-5*H*-pyrrolo[3,4-*b*]pyridin-5-one (8 mg, 1% yield). ^{1}H NMR (DMSO-d_6, 400 MHz): δ 8.96 (s, 1H), 8.36 - 8.33 (m, 1H), 8.11 (d, *J* = 8.4 Hz, 1H), 8.02 (d, *J* = 8.0 Hz, 1H), 7.54 (s, 1H), 7.51 (d, 1H), 6.66 (d, *J* = 9.2 Hz, 1H), 5.50 (d, *J* = 52.0 Hz, 1H), 4.96 (s, 2H), 3.87 - 3.75 (m, 2H), 3.71 (s, 3H), 3.65 - 3.50 (m, 2H), 2.27 - 2.16 (m, 2H). ^{19}F NMR: -175.08 ppm. LCMS(ESI): (m/z) = 379.42 $[M+H]^+$
HPLC purity: 98.07%

LMD-028, 046

Scheme:

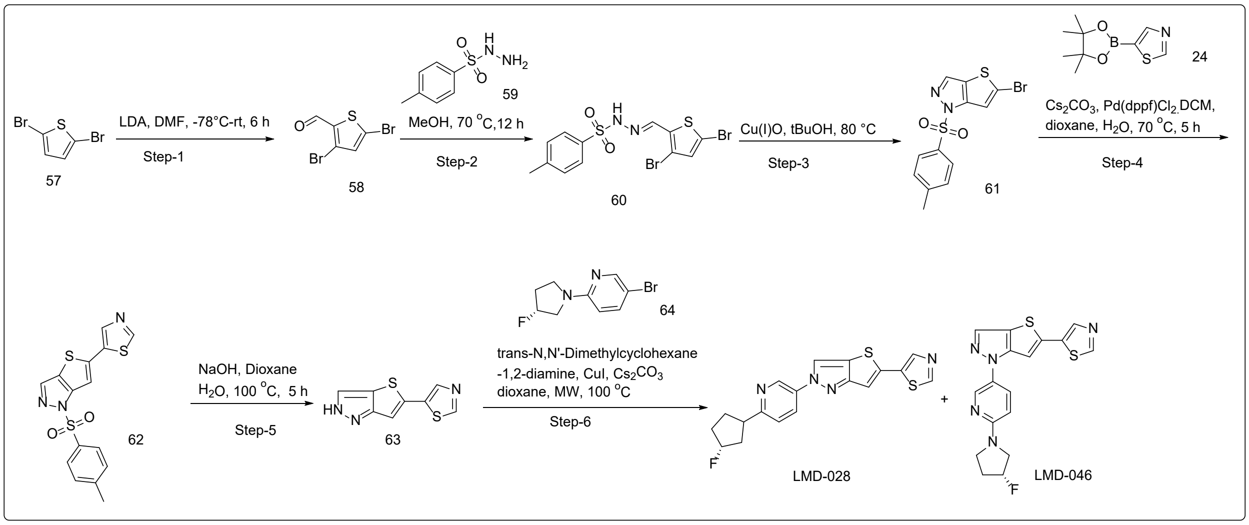

Step-1: Synthesis of 3,5-dibromothiophene-2-carbaldehyde (58):

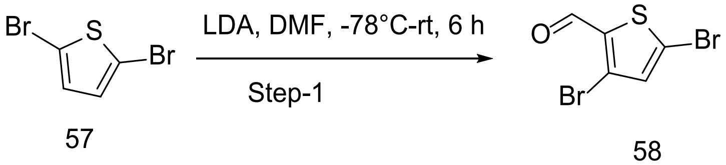

To a -78 °C cooled solution of 2,5-dibromothiophene (60.0 g, 1.0 eq, 248 mmol) in THF (600 mL) was added 2M LDA in THF (186 mL, 1.5 eq, 373 mmol) drop wise over a period of 30 min and stirred for 1h. After that, DMF (58.0 mL, 3.0 eq, 744 mmol) was added at same the temperature and allowed to room temperature for 6 h. The reaction progress was monitored by TLC. After completion of the reaction, the reaction was quenched with saturated NH_4Cl solution (500 mL), results formation of solid. The obtained solid was filtered, filtrate was concentered to get 3, 5-dibromothiophene-2-carbaldehyde (27 g, 40%) as a brown liquid as crude. This was used in the next step without any further purification. ^{1}H NMR ($CDCl_3$, 400 MHz): δ 9.82 (s, 1H), 7.14 (s, 1H).

Step-2: Synthesis of (*E*)-N'-((3,5-dibromothiophen-2-yl) methylene)-4-methylbenzenesulfonohydrazide (60):

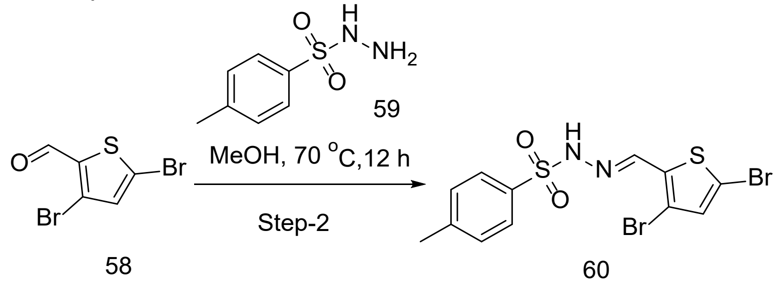

To a stirred solution of 3,5-dibromothiophene-2-carbaldehyde (27.0 g, 1.0 eq, 100 mmol) in MeOH (270 mL) was added 4-methylbenzenesulfonohydrazide (18.6 g, 1.0 eq, 100 mmol) at 0 ºC and heated at 70 °C for 12 h. The reaction progress was monitored by TLC. After completion of the reaction, reaction mixture was evaporated under reduced pressure to get the crude. The crude was, purified on column chromatography using 10% EtOAc/hexane as eluent to afford (*E*)-1-[(3,5-dibromo-2-thienyl) methylene]-2-tosylhydrazine (25.0 g, 57%) as s white solid. ^{1}H NMR (DMSO-d_6, 400 MHz): δ 7.90 (s, 1H), 7.66 (d, *J* = 8.4 Hz, 2H), 7.39 (d, *J* = 8.0 Hz, 2H), 7.26 (s, 1H), 2.33 (s, 3H). LCMS (ESI): m/z = 438.99 $[M+H]^{+\cdot}$

Step-3: Synthesis of 5-bromo-1-tosyl-1*H*-thieno[3,2-*c*] pyrazole (61):

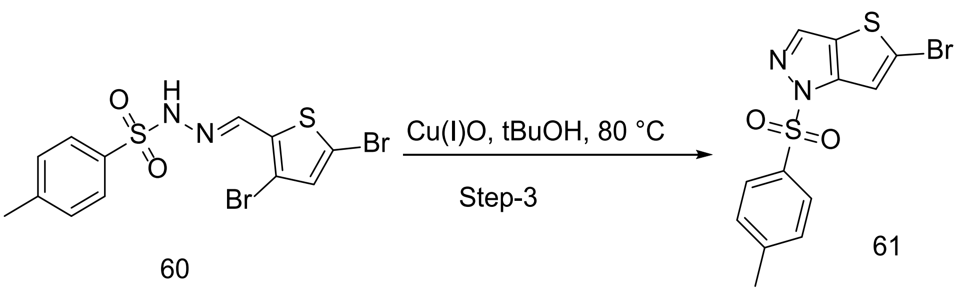

To a stirred solution of (*E*)-1-[(3,5-dibromo-2-thienyl) methylene]-2-tosylhydrazine (25.0 g, 1.0 eq, 57.1 mmol) in *tert*-butanol (250 mL) was added Cu(I)O (8.16 g, 1.0 eq, 57.1 mmol) and stirred at 80 ºC for 16 h. The progress of the reaction was monitored by TLC. After completion, the reaction mixture was cooled to room temperature, filtered through celite pad and filtrate was concentrated to get the crude. The crude residue was purified on column chromatography using 10% EtOAc /hexane as eluent to afford 5-bromo-1-tosyl-1*H*-thieno[3,2-*c*] pyrazole (18.0 g, 88%) as a white solid. ^{1}H-NMR (DMSO-d_6, 400 MHz): δ 8.12 (s, 1H), 7.86 (d, *J* = 8.0 Hz, 2H), 7.70 (s, 1H), 7.44 (d, *J* = 8.0 Hz, 2H), 2.36 (s, 3H).

Step-4: Synthesis 5-(thiazol-5-yl)-1-tosyl-1*H*-thieno[3,2-*c*]pyrazole (62):

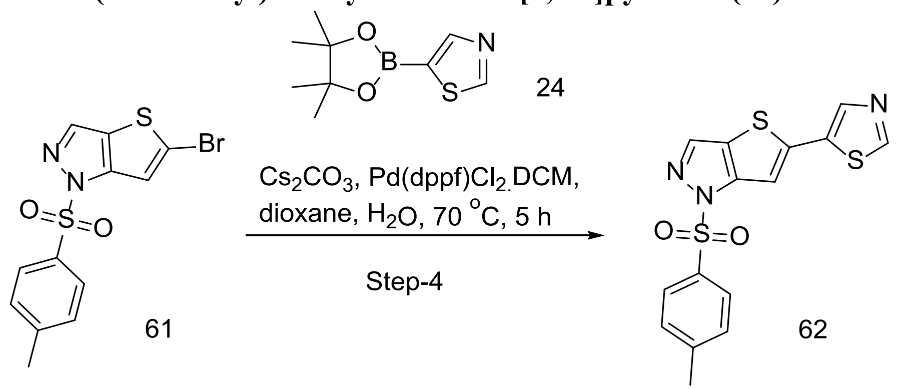

To a stirred solution 5-bromo-1-tosyl-1*H*-thieno[3,2-*c*] pyrazole (18.0 g, 1.0 eq, 50.4 mmol) and 4,4,5,5-tetramethyl-2-(1,3-thiazol-5-yl)-1,3,2-dioxaborolane (10.6 g, 1.0 eq, 50.4 mmol), in Dioxane (162 mL), H_2O (54 mL) was added Cs_2CO_3 (49.5 g, 3.0 eq, 151.2 mmol). at room temperature and purged with argon for 20 min. After that, Pd(dppf)Cl_2.DCM (2.1 g, 0.05 eq, 2.52 mmol) under argon atmosphere and heated at 100 °C for 5 h. The reaction progress was monitored by TLC. After completion of reaction, the reaction filtered through celite pad and washed with EtOAc (100 mL). The filtrate was washed with water (200 mL) and brine (100 mL). The organic layer was dried over Na_2SO_4, and concentrated under reduced pressure to get crude residue. The residue was purified on column chromatography using 10% EtOAc/hexane as eluent to afford 5-(thiazol-5-yl)-1-tosyl-1*H*-thieno[3,2-*c*]pyrazole (5.5 g, 30%) as a brown solid.

LCMS (ESI): m/z = 362.40 $[M+H]^+$.

Step-5: Synthesis of 5-(thiazol-5-yl)-2*H*-thieno [3,2-*c*] pyrazole (63):

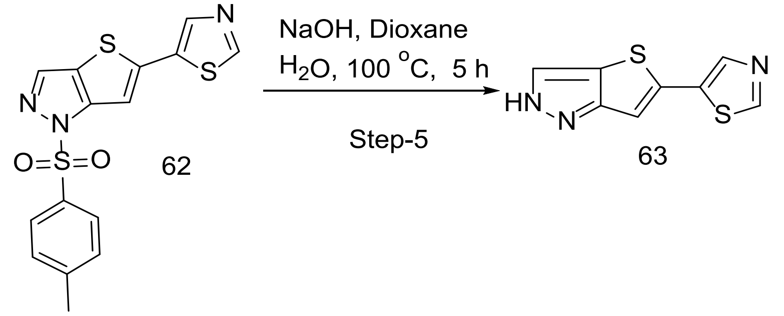

To a stirred solution of 5-(thiazol-5-yl)-1-tosyl-1*H*-thieno[3,2-*c*]pyrazole (5.5 g, 1.0 eq, 15.2 mmol) in 1,4-Dioxane (55 mL), water (55 mL) was added NaOH (1.22 g, 2.0 eq, 30.4 mmol) at 0 ºC and heated the to 100 ºC for 12 h. The progress of the reaction was monitored by TLC. After completion, the reaction was evaporated under reduced pressure to get the crude. The crude residue was purified on column chromatography using 10% MeOH/DCM as eluent to afford 5-(thiazol-5-

yl)-2*H*-thieno [3,2-*c*] pyrazole (2.8 g, 88%) as an off white solid. LCMS (ESI): m/z = 208.08 $[M+H]^{+\cdot}$

Step-6: Synthesis of 2-(6-((3*R*)-3-fluorocyclopentyl) pyridin-3-yl)-5-(thiazol-5-yl)-2*H*-thieno[3,2-*c*] pyrazole (LMD-028) & (*R*)-1-(6-(3-fluoropyrrolidin-1-yl) pyridin-3-yl)-5-(thiazol-5-yl)-1*H*-thieno[3,2-*c*] pyrazole (LMD-046):

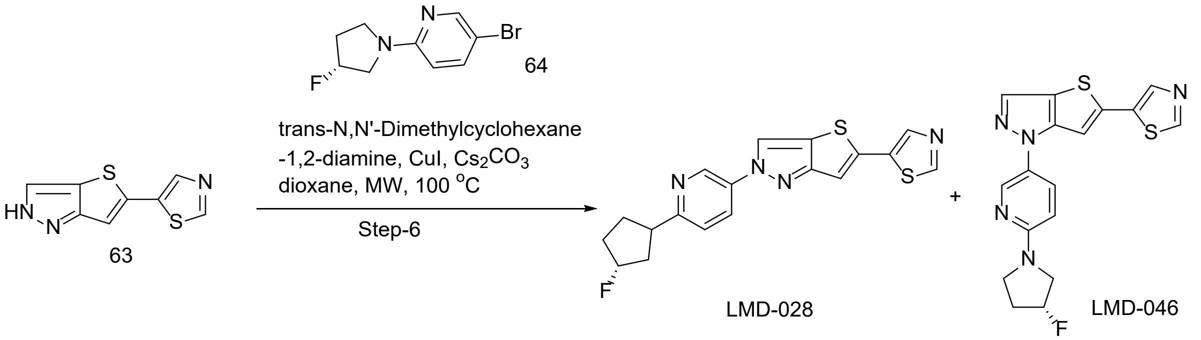

To a stirred solution of 5-(thiazol-5-yl)-2*H*-thieno[3,2-*c*] pyrazole (150 mg, 1.0 eq, 724 µmol) and (*R*)-5-bromo-2-(3-fluoropyrrolidin-1-yl) pyridine (177 mg, 1.0 eq, 724 µmol) in 1,4-dioxane (1.5 mL) was added Cs_2CO_3 (707 mg, 3.0 eq, 2.17 mmol), and degassed with argon for 10 min. After that, Cu(I)I (13.7 mg, 0.1 eq, 72.4 µmol) was added followed by addition of trans-*N*, *N*'-Dimethyl cyclohexane (10.3 mg, 0.1 eq, 72.4 µmol) and irradiated at 100 °C in MW for 1 h. The reaction progress was monitored by TLC. After completion of the reaction, filtered through celite pad, washed with EtOAc (20 mL). The filtrate was concentrated under reduced pressure to get crude. The crude residue was purified by column chromatography using 2% MeOH/DCM as eluent to get desired compound, which was re-purified on prep-HPLC to afford 2-(6-((3*R*)-3-fluorocyclopentyl) pyridin-3-yl)-5-(thiazol-5-yl)-2*H*-thieno[3,2-*c*] pyrazole (12.4 mg, 4.8%) as an off white solid and (*R*)-1-(6-(3-fluoropyrrolidin-1-yl) pyridin-3-yl)-5-(thiazol-5-yl)-1*H*-thieno[3,2-*c*] pyrazole (3.6 mg, 1.8%) as an off white solid.

LMD-028: ^{1}H-NMR (DMSO-*d*$_6$, 400 MHz): δ 9.13 (s, 1H), 8.57 (d, *J* = 8.0 Hz, 1H), 8.32 (s, 1H), 8.03 (s, 1H), 7.97 (dd, *J* = 2.8 Hz, 9.2 Hz, 1H), 7.85 (s, 1H), 6.68 (d, *J* = 9.2 Hz, 1H), 5.54 (d, *J* = 53.2 Hz, 1H), 3.90 - 3.60 (m, 3H), 3.55 - 3.45 (m, 1H), 2.35 - 2.15 (m, 2H). ^{19}F NMR: -174.7 ppm. LCMS (ESI): m/z = 372.38 $[M+H]^+$. HPLC purity: 96.44%

LMD-046: ^{1}H-NMR (DMSO-*d*$_6$, 400 MHz): δ 9.15 (s, 1H), 8.62 (s, 2H), 8.27 (s, 1H), 8.03 (s, 1H), 8.03 (dd, *J* = 2.4 Hz, 8.8 Hz, 1H), 7.61 (s, 1H), 6.67 (d, *J* = 9.2 Hz, 1H), 5.54 (d, *J* = 54.0 Hz, 1H),

3.85 - 3.6 (m, 3H), 3.55 - 3.45 (m, 1H), 2.45 - 2.15 (m, 2H); ^{19}F NMR: -174.7 ppm. LCMS (ESI): m/z = 372.14 $[M+H]^{+\cdot}$ HPLC purity: 97.72%

LMD-029

Scheme:

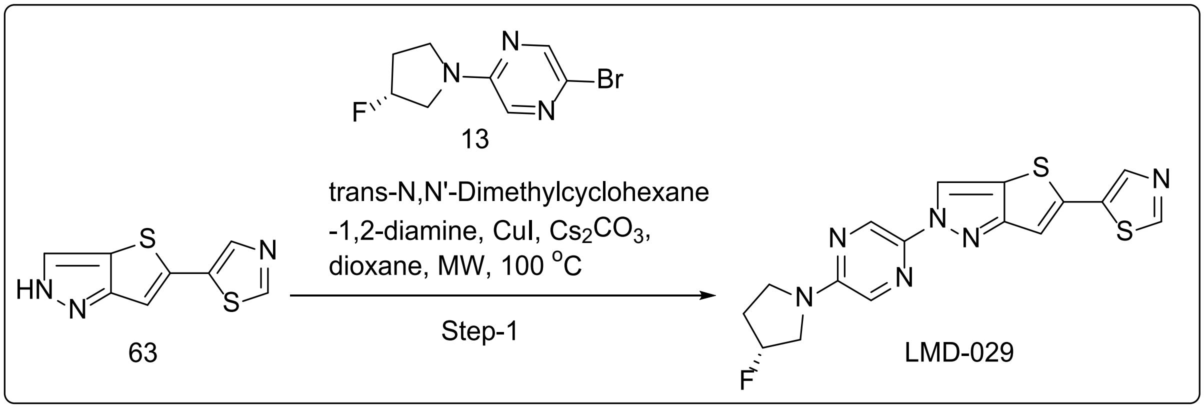

Step-1: Synthesis of (*R*)-2-(5-(3-fluoropyrrolidin-1-yl) pyrazin-2-yl)-5-(thiazol-5-yl)-2*H*-thieno[3,2-*c*] pyrazole (LMD-029):

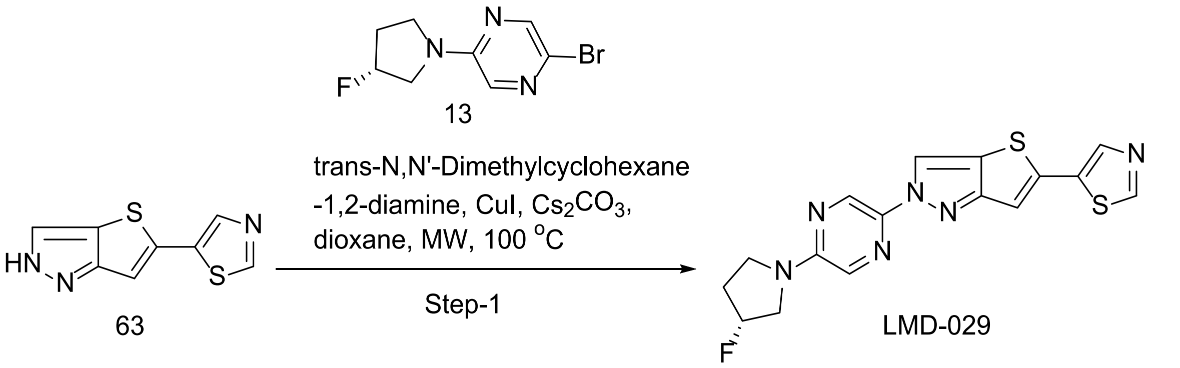

To a stirred solution of 5-(thiazol-5-yl)-2*H*-thieno[3,2-*c*] pyrazole (150 mg, 1.0 eq, 724 µmol) and (*R*)-2-bromo-5-(3-fluoropyrrolidin-1-yl) pyrazine (178 mg, 1.0 eq, 724 µmol) in 1,4-Dioxane (2 ml) was added Cs_2CO_3 (707 mg, 3.0 eq, 2.17 mmol), and degassed with argon gas for 10 mins. After that, trans-*N*, *N'*-Dimethyl cyclohexane (10.3 mg, 0.1 eq, 72.4 µmol) was added followed by Cu(I)I (13.7 mg, 0.1 eq, 724 µmol) under argon atmosphere. The resulting reaction mixture was irradiated under microwave at 100 °C for 1 h. The reaction progress was monitored by TLC. After completion of reaction, filtered through celite pad, and washed with EtOAc (20 mL). The filtrate was concentrated under reduced pressure to get crude. The crude residue was purified by flash column chromatography, using 2% MeOH/DCM as eluent to get desired compound, which was re-purified on prep-HPLC to afford (*R*)-2-(5-(3-fluoropyrrolidin-1-yl) pyrazin-2-yl)-5-(thiazol-5-yl)-2*H*-thieno[3,2-*c*] pyrazole (2 mg, 0.7%) as an off white solid. ^{1}H NMR (DMSO-d_6, 400 MHz):

δ 9.16 (s, 1H), 8.73 (s, 1H), 8.70 (s, 1H), 8.29 (s, 1H), 7.94 (s, 1H), 7.61 (s, 1H), 5.50 (d, *J* = 48.0 Hz, 1H), 3.90 – 3.50 (m, 4H), 2.40 – 1.96 (m, 2H). LCMS (ESI): m/z = 373.2 $[M+H]^+$. ^{19}F NMR: -175.1 ppm. HPLC purity: 97.73%

LMD-031

Scheme:

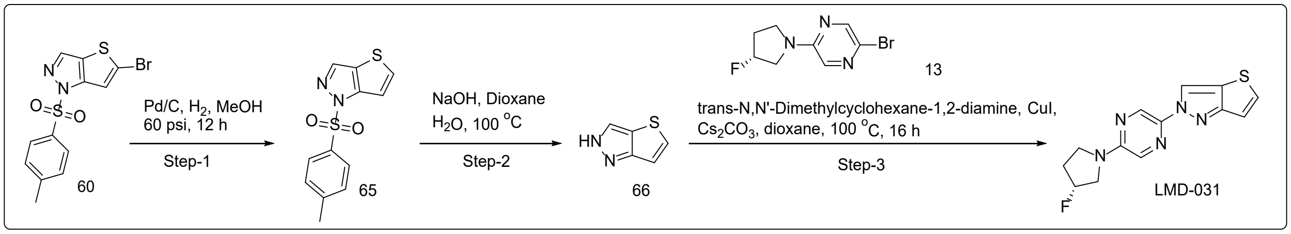

Step-1: Synthesis of 1-tosyl-1*H*-thieno[3,2-*c*]pyrazole (65):

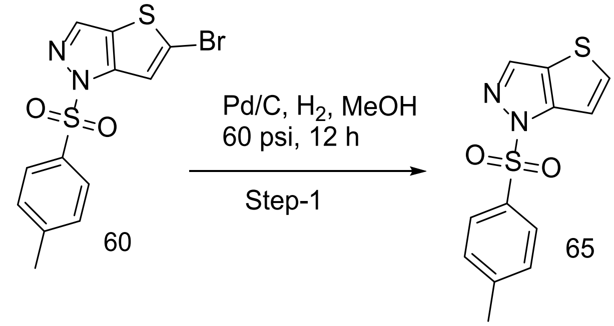

To a stirred solution of 5-bromo-1-tosyl-1*H*-thieno[3,2-*c*] pyrazole (400 mg, 1.0 eq, 1.12 mmol) in MeOH (4 mL) was added Pd/C (200 mg) at room temperature under N_2 atmosphere. The reaction mixture was degassed with N_2 gas, followed by H_2 gas and stirred under H_2 atmosphere at 60 *psi* for 12 h. The reaction progress was monitored by TLC. After completion of the reaction, filtered through celite pad and washed with EtOAc (20 mL). The organic layer was dried over Na_2SO_4, and concentrated under reduced pressure to afford 1-tosyl-1*H*-thieno[3,2-*c*]pyrazole (200 mg, crude) as an off-white solid as crude. This was used in the next step without any further purification. LCMS (ESI): m/z = 279.09 $[M+H]^+$.

Step-2: Synthesis of 2*H*-thieno[3,2-*c*] pyrazole (66):

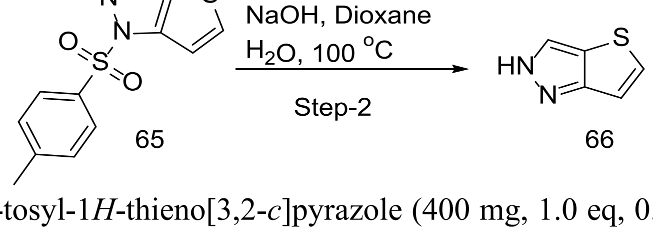

To a stirred solution 1-tosyl-1*H*-thieno[3,2-*c*]pyrazole (400 mg, 1.0 eq, 0.719 mmol) in Dioxane (4 mL), H_2O (4 mL) was added NaOH (114 mg, 2.0 eq, 1.43 mmol) at 0 °C and stirred at 100 °C

for 5 h. The reaction progress was monitored by TLC. After completion of the reaction, the solvent was evaporated under reduced pressure to get the crude. The obtained crude was purified on column chromatography using 10% Methanol/DCM as eluent to afford 2*H*-thieno[3,2-*c*] pyrazole (120 mg, 67%) as an off white solid. ^{1}H NMR (DMSO-d_6, 400 MHz): δ 13.33 (bs, 1H), 7.9 (bs, 1H), 7.74 (s, 1H), 7.57 (d, *J* = 4.8 Hz, 1H), 7.57 (d, *J* = 4.8 Hz, 1H), LCMS (ESI): m/z = 125.0 $[M+H]^{+\cdot}$

Step-3: Synthesis of 2-(5-((3*R*)-3-fluorocyclopentyl) pyrazin-2-yl)-2*H*-thieno[3,2-*c*] pyrazole (LMD-031):

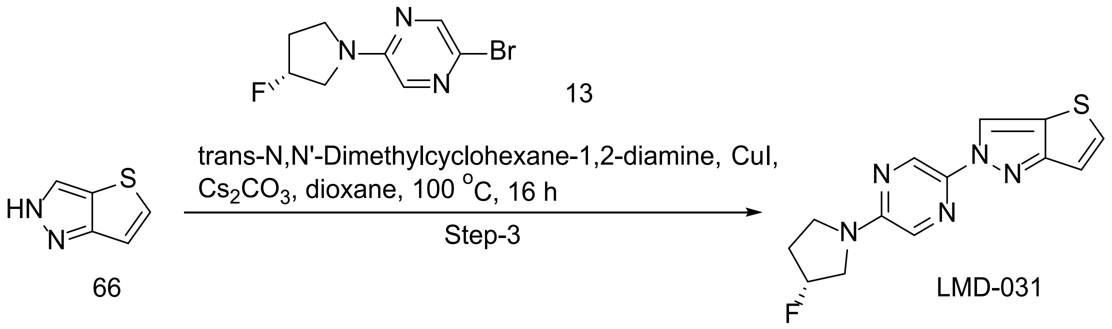

To a stirred solution of 2*H*-thieno[3,2-*c*] pyrazole (120 mg, 1.0 eq, 97 µmol) and 5-bromo-2-[(*R*)-3-fluoro-1-pyrrolidinyl] pyrazine (238 mg, 1.0 eq, 97 µmol) in 1,4-dioxane (1.5 mL) was added Cs_2CO_3 (945 mg, 3.0 eq, 2.9 mmol), and purged with argon for 10 min. Then, trans-*N*, *N*'-Dimethyl cyclohexane (13.7 mg, 0.1 eq, 9.6 µmol) was added followed by Cu(I)I (18 mg, 0.1 eq, 9.6 µmol) under argon atmosphere. The resulting reaction mixture was irradiated at 100 °C in MW for 1 h. The reaction progress was monitored by TLC. After completion of the reaction, filtered through celite pad, and washed with EtOAc (20 mL). The filtrate was concentrated under reduced pressure to get crude. The crude residue was purified by flash column chromatography using 2% MeOH/DCM as eluent to get crude compound, which was re-purified on prep-HPLC to afford 2-(5-((3*R*)-3-fluorocyclopentyl) pyrazin-2-yl)-2*H*-thieno[3,2-*c*] pyrazole (4.3 mg, 1.54%) as an off white solid. ^{1}H NMR ($CDCl_3$, 400 MHz): δ 8.80 (s, 1H), 7.86 (s, 1H), 7.73 (s, 1H), 7.64 (d, *J* = 5.2 Hz, 1H), 7.48 (d, *J* = 5.2 Hz, 1H), 5.42 (d, *J* = 52.0 Hz, 1H), 4.0 – 3.6 (m, 4H), 2.50 - 2.10 (m, 2H). ^{19}F NMR: -174.91 ppm. LCMS (ESI): 290.53 m/z $[M+H]^{+}$. HPLC purity: 97.9%

LMD-032

Scheme:

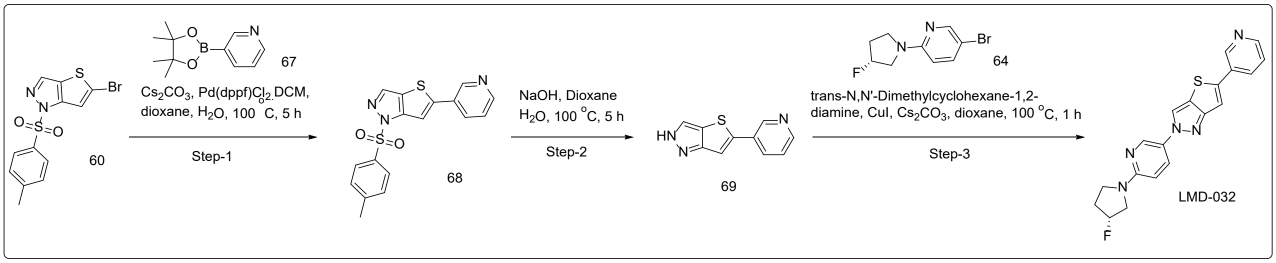

Step-1: Synthesis of 5-(pyridin-3-yl)-1-tosyl-1*H*-thieno[3,2-*c*]pyrazole (68):

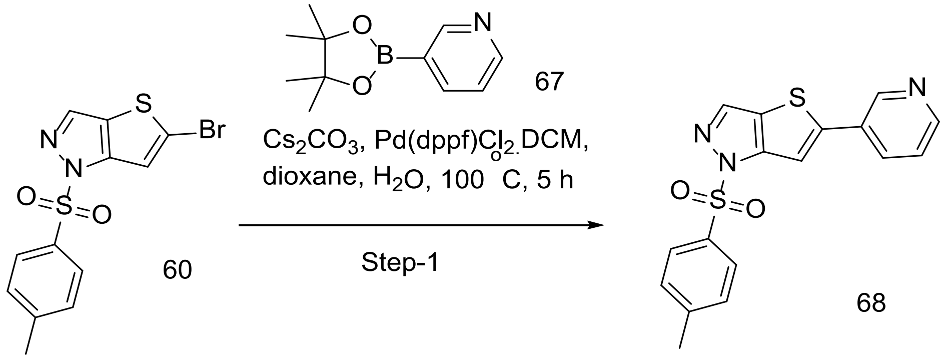

To a stirred solution of 5-bromo-1-tosyl-1*H*-thieno[3,2-*c*]pyrazole (1.0 g, 1.0 eq, 2.8 mmol) and 4,4,5,5-tetramethyl-2-(3-pyridyl)-1,3,2-dioxaborolane (574 mg, 1.0 eq, 2.8 mmol) in 1,4-dioxane (9 mL), water (3 mL) was added Cs_2CO_3 (2.74 g, 3.0 eq, 8.4 mmol) at room temperature and degassed with argon for 10 min. After that, Pd(dppf)Cl_2.DCM) (229 mg, 0.1 eq, 280 µmol) was added under argon atmosphere and heated at 100 °C for 5 h. The reaction progress was monitored by TLC. After completion, the reaction mixture was filtered through celite pad and washed with EtOAc (30 mL). The filtrate was washed with water (20 mL) and brine solution (20 mL). The organic layer was dried over Na_2SO_4, and concentrated under reduced pressure to get crude. The residue was purified on column chromatography using 10% EtOAc/hexane as eluent to afford 5-(pyridin-3-yl)-1-tosyl-1*H*-thieno[3,2-*c*]pyrazole (0.6 g, 60%) as an off white solid. LCMS (ESI): m/z = 356.96 $[M+H]^+$

Step-2: Synthesis of 5-(pyridin-3-yl)-2*H*-thieno[3,2-*c*]pyrazole (69):

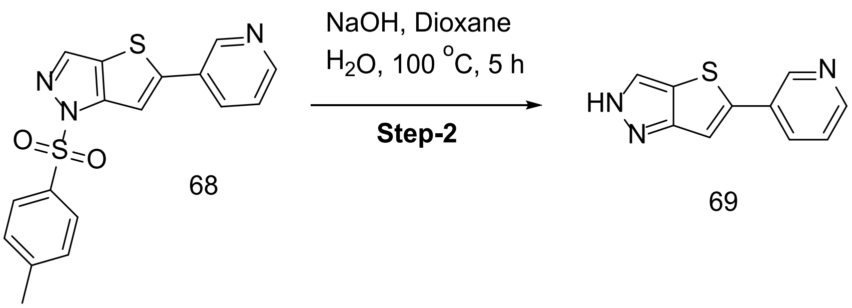

To a stirred solution of 5-(pyridin-3-yl)-1-tosyl-1*H*-thieno[3,2-*c*]pyrazole (600 mg, 1.0 eq, 8.44 mmol) in 1,4-Dioxane (6 mL), H_2O (6 mL) was added, NaOH (675 mg, 2.0 eq, 16.9 mmol) at 0 °C and heated at 100 °C for 5 h. The reaction progress was monitored by TLC. After completion of the reaction, solvent concentrated under reduced pressure to get the crude. The crude was purified on column chromatography using 10% MeOH/DCM as eluent to afford 5-(pyridin-3-yl)-2*H*-thieno[3,2-*c*] pyrazole (120 mg, 70%) as an off white solid. ^{1}H NMR (DMSO-d_6, 400 MHz): δ 13.24 (s, 1H), 8.97 (s, 1H), 8.54 (d, *J* = 3.6 Hz, 1H), 8.10 (d, *J* = 8.0 Hz, 1H), 7.83 (bs, 1H), 7.74 (bs, 1H), 7.49 - 7.46 (m, 1H). LCMS (ESI): m/z = 202.2 $[M+H]^+$

Step-3: Synthesis of (*R*)-2-(6-(3-fluoropyrrolidin-1-yl) pyridin-3-yl)-5-(pyridin-3-yl)-2*H*-thieno[3,2-*c*] pyrazole (LMD-032):

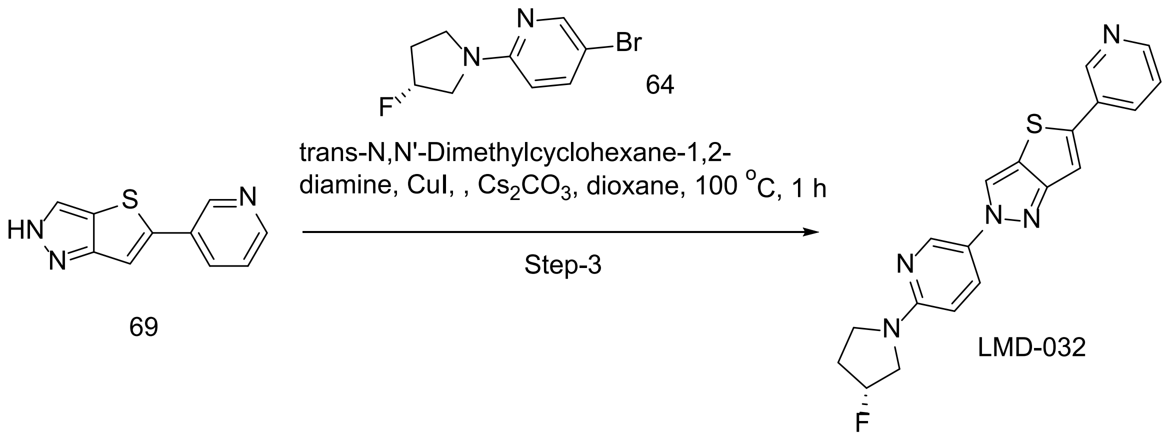

To a stirred solution of 5-(pyridin-3-yl)-2*H*-thieno[3,2-*c*] pyrazole (120 mg, 1.0 eq, 612 μmol) and (*R*)-5-bromo-2-(3-fluoropyrrolidin-1-yl)pyridine (150 mg, 1.0 eq, 612 μmol) in 1,4-Dioxane (2 mL) was added Cs_2CO_3 (707 mg, 3.0 eq, 2.17 mmol), purged with argon for 10 min, Then, Cu(I)I (23 mg, 0.2 eq, 122 μmol) was added followed by trans-*N*, *N'*-Dimethyl cyclohexane (25.4 mg, 0.2 eq, 122 μmol) under argon atmosphere. The resulting reaction mixture was irradiated to 100 °C in MW for 1 h. The reaction progress was monitored by TLC. The reaction mass was filtered through celite pad, and washed with EtOAc (20 mL). The filtrate was concentrated under reduced pressure to get crude. The crude residue was purified by flash column chromatography, using 2% MeOH/DCM as eluent to get desired compound, which was re-purified on prep-HPLC to afford (*R*)-2-(6-(3-fluoropyrrolidin-1-yl)pyridin-3-yl)-5-(pyridin-3-yl)-2*H*-thieno[3,2-*c*]pyrazole (4.3 mg, 1.8%) as an off white solid. ^{1}H NMR (DMSO-d_6, 400 MHz): δ 9.05 (s, 1H), 8.63 (d, *J* = 2.4 Hz, 1H), 8.57 (d, *J* = 4.0 Hz, 1H), 8.21 (d, *J* = 8.0 Hz, 1H), 8.10 (s, 2H), 8.07 (s, 1H), 8.02 (dd, *J* = 2.4 Hz, *J* = 8.8 Hz, 1H), 7.51 - 7.48 (m, 1H), 6.69 (d, *J* = 9.2 Hz, 1H), 5.5 (d, *J* = 48.0 Hz, 1H),

3.80 – 3.45 (m, 4H), 2.35 - 2.15 (m, 2H). ^{19}F NMR: -174.73 ppm. LCMS (ESI): m/z = 366.2 $[M+H]^+$. HPLC Purity: 97.44%

LMD-033

Scheme:

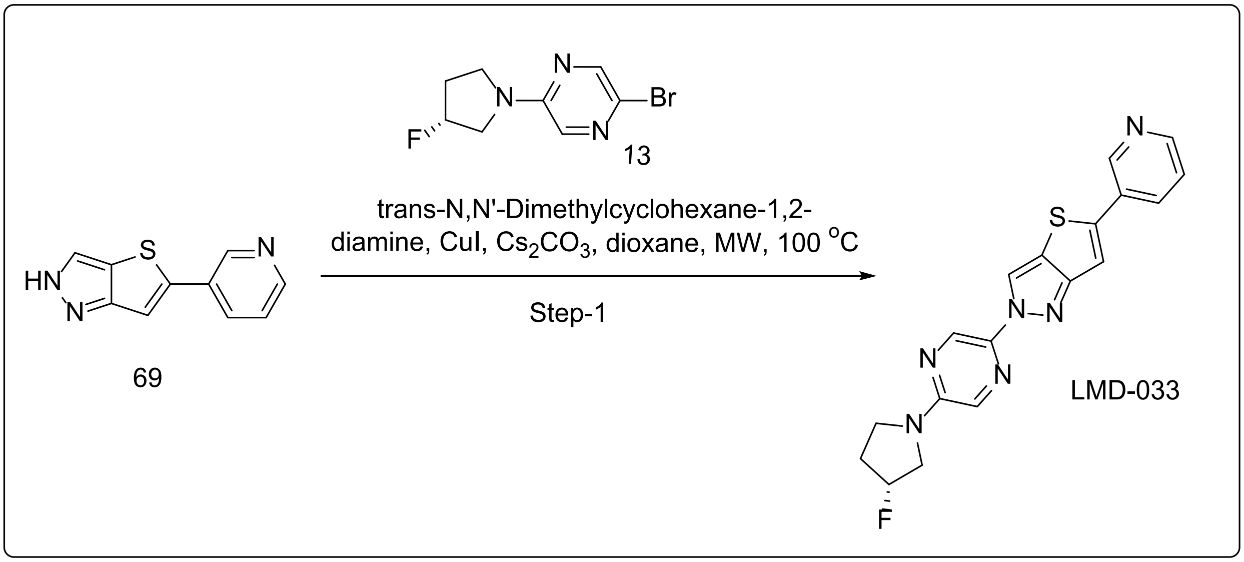

Step-1: Synthesis of (*R*)-2-(5-(3-fluoropyrrolidin-1-yl) pyrazin-2-yl)-5-(pyridin-3-yl)-2*H*-thieno[3,2-*c*] pyrazole (LMD-033):

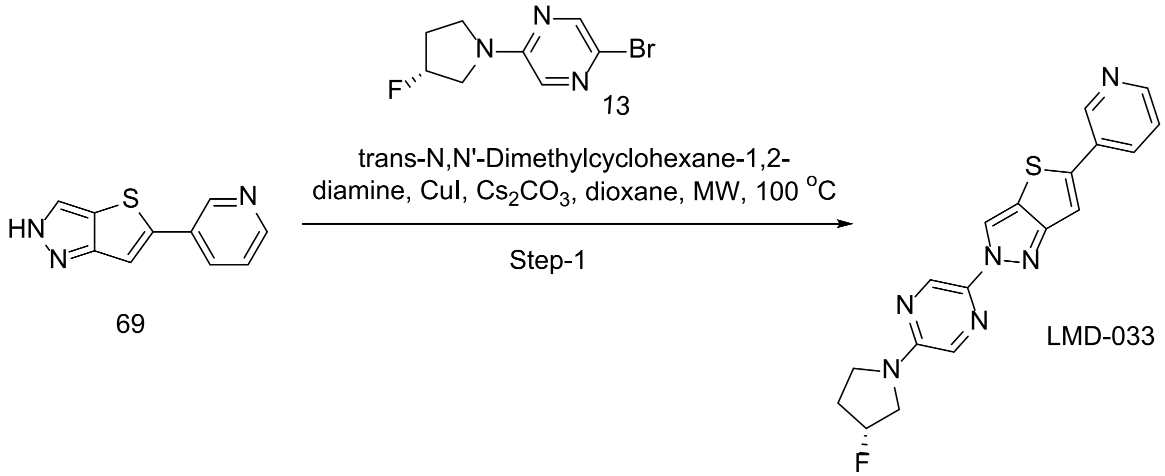

To a stirred solution of 5-(pyridin-3-yl)-2*H*-thieno[3,2-*c*] pyrazole (220 mg, 1.0 eq, 894 µmol) and (*R*)-2-bromo-5-(3-fluoropyrrolidin-1-yl) pyrazine (180 mg, 1.0 eq, 894 µmol) in 1,4-Dioxane (2 mL) was added Cs_2CO_3 (874 mg, 3.0 eq, 2.68 mmol) and purged with argon gas 10 mins. After that, trans-*N*, *N*'-dimethyl cyclohexane (25.4 mg, 0.2 eq, 122 µmol)) was added followed by Cu(I)I (23 mg, 0.2 eq, 122 µmol) and irradiated to 100 °C in MW for 1 h. The reaction progress was monitored by TLC. After completion of the reaction, filtered through celite pad, and washed with

EtOAc (20 mL). The filtrate was concentrated under reduced pressure to get crude. The crude residue was purified by flash column chromatography, using 2% MeOH/DCM as eluent to get crude compound, which was re-purified on prep-HPLC to afford (*R*)-2-(5-(3-fluoropyrrolidin-1-yl) pyrazin-2-yl)-5-(pyridin-3-yl)-2*H*-thieno[3,2-*c*] pyrazole (6 mg, 1.5%) as an off white solid. ^{1}H NMR ($CDCl_3$, 400 MHz): δ 8.99 (s, 1H), 8.84 (s, 1H), 8.59 (d, *J* = 4.8 Hz, 1H), 7.98-7.96 (m, 2H), 7.89 (s, 1H), 7.76 (s, 1H), 7.39-7.36 (m, 1H), 5.51 (d, *J* = 55.6 Hz, 1H), 3.99 – 3.66 (m, 4H), 2.53 – 2.12 (m, 2H). ^{19}F NMR: -176.08 ppm. LCMS (ESI): 367.18 m/z $[M+H]^+$. HPLC purity: 98.79%

LMD-034

Scheme:

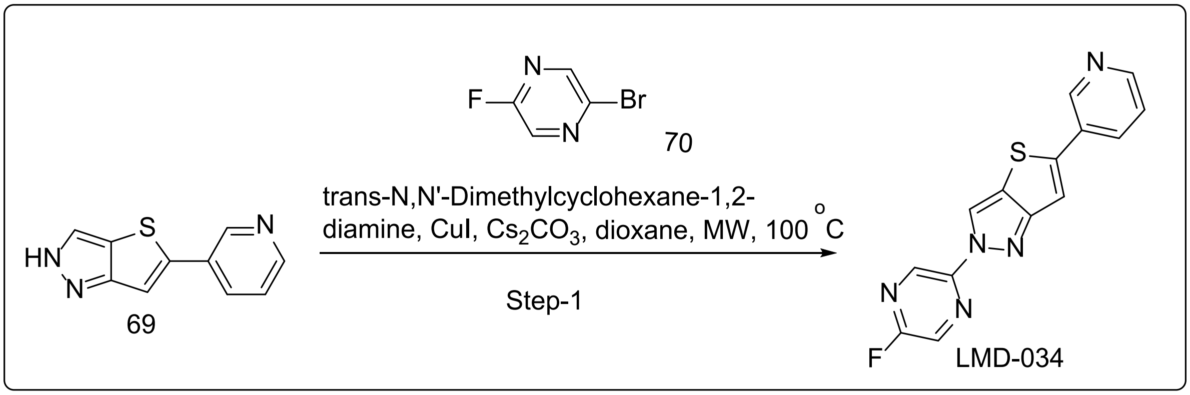

Step-1: Synthesis of 2-(5-fluoropyrazin-2-yl)-5-(pyridin-3-yl)-2*H*-thieno[3,2-*c*] pyrazole (LMD-034):

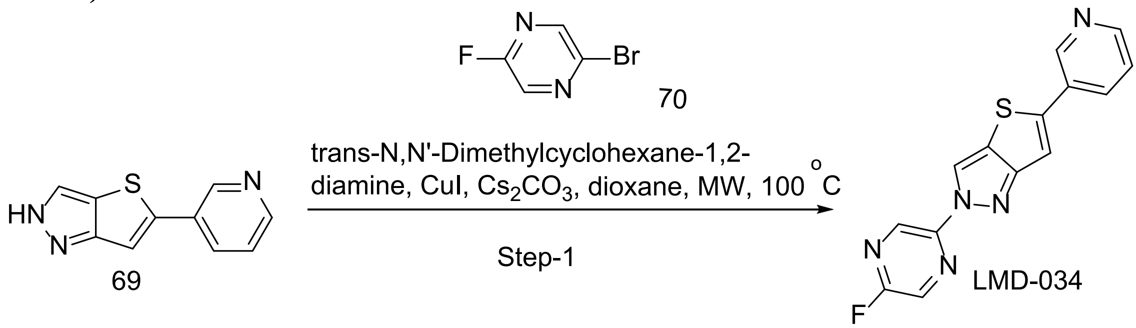

To a stirred solution of 5-(pyridin-3-yl)-2*H*-thieno[3,2-*c*] pyrazole (320 mg, 1.0 eq, 894 µmol) and 2-bromo-5-fluoropyrazine (180 mg, 1.0 eq, 894 µmol) in 1,4-Dioxane (3 mL) was added Cs_2CO_3 (874 mg, 3.0 eq, 2.68 mmol) and purged with argon gas for 10 min. After that, trans-*N*, *N*'-Dimethyl cyclohexane (25.4 mg, 0.2 eq, 179 µmol) was added followed by CuI (60 mg, 0.2 eq, 179 µmol) under argon atmosphere. The resulting reaction mixture was irradiated to 100 ºC in MW for 1 h. The reaction progress was monitored by TLC. After completion of the reaction,

filtered through celite pad, and washed with EtOAc (20 mL). The filtrate was concentrated under reduced pressure to get crude. The crude residue was purified by flash column chromatography, using 2% MeOH/DCM as eluent to get compound, which was re-purified on prep-HPLC to afford 2-(5-fluoropyrazin-2-yl)-5-(pyridin-3-yl)-2*H*-thieno[3,2-*c*] pyrazole (6 mg, 1.27%) as an off white solid. ^{1}H NMR (DMSO-d_6, 400 MHz): δ 9.02 (s, 1H), 8.96 (s, 1H), 8.65 (d, *J* = 8.0 Hz, 1H), 8.61 (d, *J* = 4.8 Hz, 1H), 8.31 (s, 1H), 8.20 (d, *J* = 8.0 Hz, 1H), 8.17 (s, 1H), 7.54 - 7.51 (m, 1H). ^{19}F NMR: -87.45 ppm. LCMS (ESI): m/z = 298.2 $[M+H]^+$. HPLC purity: 95.24%

LMD-051

Scheme:

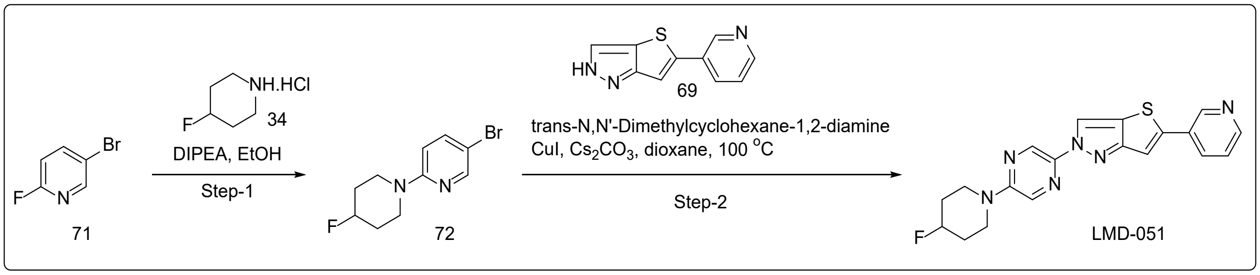

Step-1: Synthesis of 5-bromo-2-(4-fluoropiperidin-1-yl) pyridine (72):

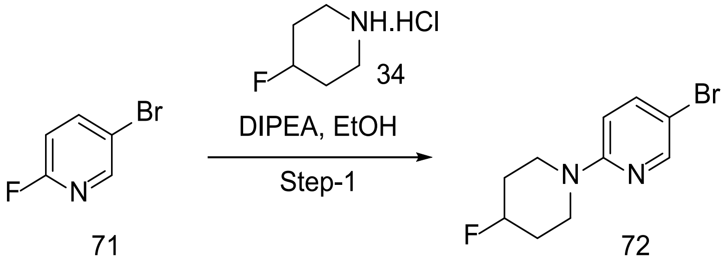

To a stirred solution of 5-bromo-2-fluoropyridine (500 mg, 1 eq, 2.81 mmol) in EtOH (5 ml) was added 4-fluoropiperidine.HCl (352 mg, 3.0 eq, 3.41 mmol) and DIPEA (1.56 mL, 3.0 eq, 8.52 mmol) at 0 °C and heated at 130 °C for 12 h. The progress of the reaction was monitored by TLC. After completion of the reaction, reaction was evaporated under reduced pressure to get the crude. The crude residue was purified on column chromatography by using 20% EtOAc/hexane as eluent to afford 5-bromo-2-(4-fluoropiperidin-1-yl) pyridine (150 mg, 20.3 %) as an off white solid. LCMS (ESI): 259.08 m/z $[M+H]^{+.}$

Step-2: Synthesis of 2-(5-(4-fluoropiperidin-1-yl) pyrazin-2-yl)-5-(pyridin-3-yl)-2*H*-thieno[3,2-*c*] pyrazole (LMD-051):

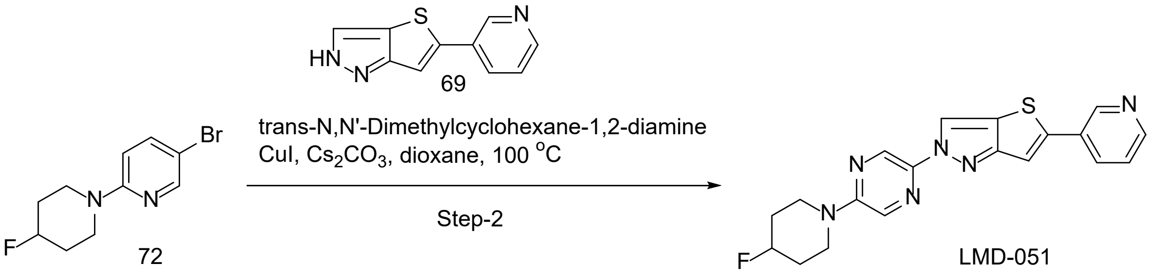

To a stirred solution of 5-bromo-2-(4-fluoropiperidin-1-yl) pyridine (150 mg, 1.0 eq, 579 µmol), and 5-(pyridin-3-yl)-2*H*-thieno [3,2-*c*] pyrazole (117 mg, 1.0 eq, 579 µmol)] in 1-4 Dioxane (4 mL) was added Cs_2CO_3 (566 mg, 3.0 eq, 1.74 mmol) and degassed with argon for 10 min. After that, Cu(I)I (22 mg, 0.2 eq, 116 µmol) was added, followed by trans-*N*, *N*'-Dimethyl cyclohexane (16.6 mg, 0.2 eq, 116 µmol) and heated at 100 °C in MW for 1 h. The reaction progress was monitored by TLC. After completion of the reaction, filtered through celite pad, washed with EtOAc (10 mL). The filtrate was concentrated under reduced pressure to get crude. The crude residue was purified by column chromatography using 2% MeOH/DCM as eluent to get desired compound, which was re-purified on prep-HPLC to afford 2-(5-(4-fluoropiperidin-1-yl) pyrazin-2-yl)-5-(pyridin-3-yl)-2*H*-thieno[3,2-*c*] pyrazole (15 mg, 6.8%) as an off white solid. ^{1}H NMR (DMSO-*d*$_6$, 400 MHz): δ 8.98 (d, *J* = 1.2 Hz, 1H), 8.71 (s, 1H), 8.59 (d, *J* = 3.6 Hz, 1H), 8.32 (s, 1H), 8.16 - 8.14 (m, 2H), 8.06 (s, 1H), 7.53 – 7.50 (m, 1H), 4.92 (d, *J* = 52.4 Hz, 1H), 3.85 - 3.78 (m, 2H), 3.65 - 3.58 (m, 2H), 2.04 - 1.92 (m, 2H), 1.81 – 1.73 (m, 2H). ^{19}F NMR: -177.38 ppm LCMS (ESI): m/z = 381.28 [M+H]$^+$. HPLC purity: 97.84%

LMD-052 & 073

Scheme:

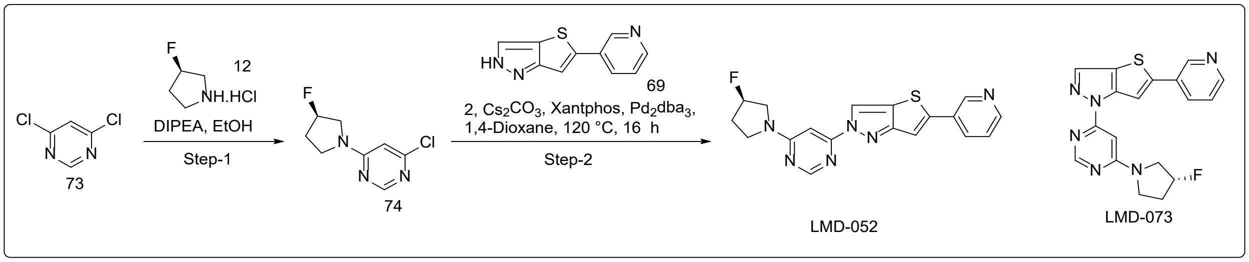

Step-1: Synthesis of (*R*)-4-chloro-6-(3-fluoropyrrolidin-1-yl) pyrimidine (74):

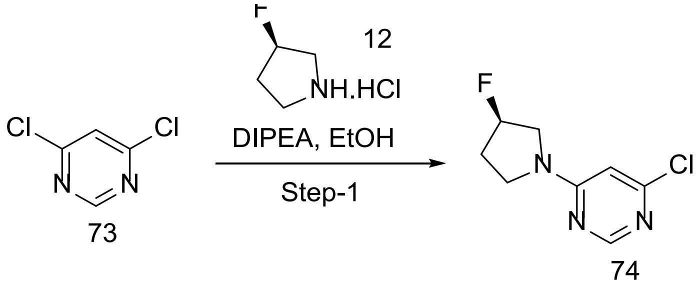

To a stirred solution of 4,6-dichloropyrimidine (1.0 g, 1.0 eq, 6.8 mmol) and (*R*)-3-fluoropyrrolidine hydrochloride (843 mg, 1.0 eq, 6.8 mmol), in EtOH (10 mL) in a seal tube was added DIPEA (3.59 mL, 3.0 e q, 20.1 mmol) at room temperature and heated at 100 °C for 2 h. The reaction progress was monitored by TLC. After completion of the reaction, reaction mixture was evaporated under reduced pressure to get the crude. The crude residue was purified on column chromatography using 15% EtOAc/hexane as eluent to afford (*R*)-4-chloro-6-(3-fluoropyrrolidin-1-yl) pyrimidine (1.0 g, 73%) as an off white solid. ^{1}H NMR (DMSO-*d*$_6$, 400 MHz): δ 8.34 (s, 1H), 6.67 (s, 1H), 5.55 - 5.36 (m, 1H), 3.83 - 3.57 (m, 4H), 2.40 - 2.01 (m, 2H).

Step-2: Synthesis of (*R*)-2-(6-(3-fluoropyrrolidin-1-yl) pyrimidin-4-yl)-5-(pyridin-3-yl)-2*H*-thieno [3, 2-*c*] pyrazole (LMD-052 & 073):

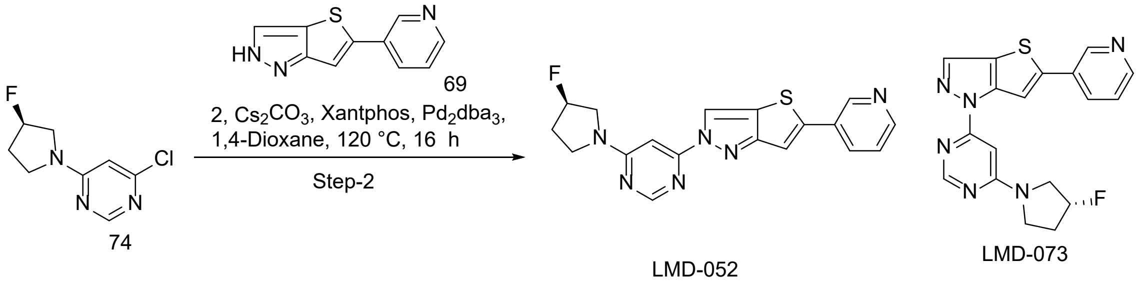

To a stirred solution of (*R*)-4-chloro-6-(3-fluoropyrrolidin-1-yl)pyrimidine (100 mg, 1.0 eq, 0.5 mmol), 5-(pyridin-3-yl)-2*H*-thieno[3,2-*c*]pyrazole (150 mg, 1.5 eq, 0.74 mmol) in 1,4-dioxane (5 mL) was Cs_2CO_3 (485 mg, 3.0 eq, 1.49 mmol), and purged with argon for 20 min. After that, Xantphos (57.4 mg, 0.2 eq, 0.01 mmol) was added followed by Pd_2dba_3 (44.2 mg, 0.1 eq, 0.05 mmol) in a sealed tube under argon atmosphere and heated at 120 °C for 16 h. The reaction progress was monitored by TLC. After completion of the reaction, filtered through celite pad, washed with EtOAc (30 mL). The filtrate was concentrated under reduced pressure to get crude. The crude residue was purified by column chromatography using 30% EtOAc/hexane, as eluent to get desired compound, which was re-purified on prep-HPLC to afford (*R*)-2-(6-(3-

fluoropyrrolidin-1-yl) pyrimidin-4-yl)-5-(pyridin-3-yl)-2*H*-thieno[3,2-*c*]pyrazole (**LMD-052**) (18.6 mg, 10.22%) as an off-white solid and (*R*)-1-(6-(3-fluoropyrrolidin-1-yl) pyrimidin-4-yl)-5-(pyridin-3-yl)-1*H*-thieno [3, 2-*c*] pyrazole (**LMD-073**) (15.2 mg, 8.35%) as an off-white solid.

LMD-052 -Analytical data

^{1}H NMR ($CDCl_3$, 400 MHz): δ 8.98 (s, 1H), 8.59 (t, *J* = 8.8 Hz, 2H), 8.18 (s, 1H), 8.00-7.98 (dd, *J* = 1.6 Hz, 8.0 Hz, 1H), 7.94 (s, 1H), 7.38 - 7.35 (m, 1H), 6.89 (bs, 1H), 5.42 (d, *J* = 52.4 Hz, 1H), 4.08 - 3.69 (m, 4H), 2.53 - 2.10 (m, 2H). ^{19}F NMR: -177.17 ppm. LCMS (ESI): m/z = 367.5 $[M+H]^{+}$. HPLC purity: 98.56%

LMD-073 -Analytical data

^{1}H NMR ($CDCl_3$, 400 MHz): δ 8.98 (d, *J* = 2.0 Hz, 1H), 8.79 (s, 1H), 8.64 (d, *J* = 4.8 Hz, 1H), 8.52 (s, 1H). 7.97 (t, *J* = 6.0 Hz, 1H), 7.42 - 7.39 (m, 2H), 7.02 (bs, 1H), 5.44 (d, *J* = 53.6 Hz, 1H), 4.11 (bs, 1H), 3.90 - 3.60 (m, 4H), 2.48 - 2.10 (m, 2H). ^{19}F NMR: -177.23 ppm. LCMS (ESI): m/z: 367.0 $[M+H]^{+}$. HPLC purity: 97.82%

LMD-061, 070

Scheme:

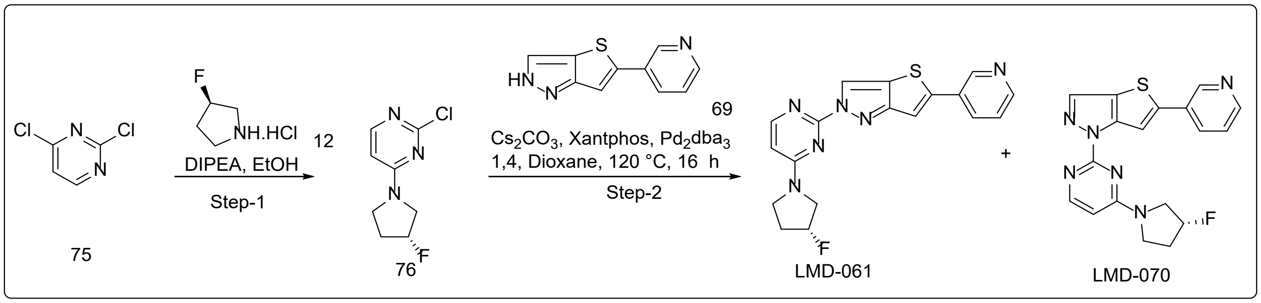

Step-1: Synthesis of (*R*)-2-chloro-4-(3-fluoropyrrolidin-1-yl) pyrimidine (76):

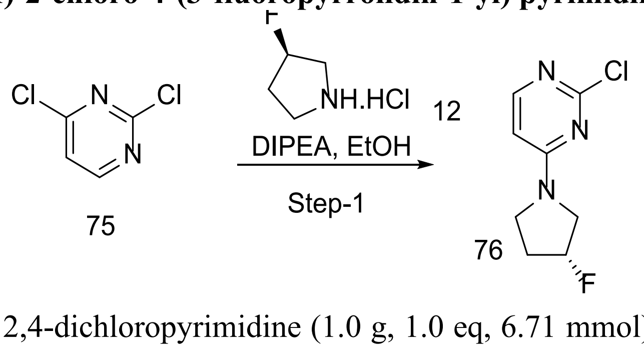

To a stirred solution of 2,4-dichloropyrimidine (1.0 g, 1.0 eq, 6.71 mmol) in EtOH (20 mL) in a seal tube was added (*R*)-3-fluoropyrrolidine hydrochloride (838 mg, 1.0 eq, 6.71 mmol), followed by *N*-ethyldiisopropylamine (3.59 mL, 3.0 eq, 20.27 mmol) at room temperature and heated at 130

°C for 16 h. The progress of the reaction was monitored by TLC. After completion, the solvent was removed under reduced pressure to get crude. The crude residue was taken up in dichloromethane (20 mL), was washed with saturated NH_4Cl solution (20 mL) and water (20 mL). The organic layer was dried over Na_2SO_4, and concentrated under reduced pressure to get crude. The residue was purified on column chromatography using 20% EtOAc/hexane to afford the title compound (*R*)-2-chloro-4-(3-fluoropyrrolidin-1-yl) pyrimidine (800 mg, 59.11%) as an off white solid. ^1H NMR (DMSO-d_6, 400 MHz): δ 8.07 (d, *J* = 4.8 Hz, 1H), 6.58 - 6.55 (m, 1H), 5.35 - 5.75 (m, 1H), 3.85-3.68 (m, 3H), 3.47 - 3.40 (m, 1H), 2.35 - 2.06 (m, 2H). LCMS (ESI): (m/z): 202.1 $[M+H]^+$.

Step-2: Synthesis of (*R*)-2-(4-(3-fluoropyrrolidin-1-yl) pyrimidin-2-yl)-5-(pyridin-3-yl)-2*H*-thieno[3,2-*c*] pyrazole (LMD-061) & (*R*)-1-(4-(3-fluoropyrrolidin-1-yl) pyrimidin-2-yl)-5-(pyridin-3-yl)-1*H*-thieno[3,2-*c*] pyrazole (LMD-070):

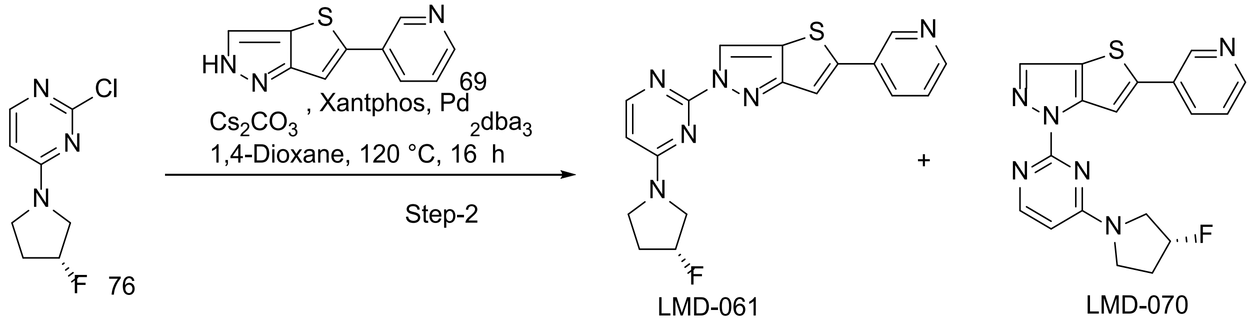

To a stirred solution of 2-chloro-4-[(*R*)-3-fluoro-1-pyrrolidinyl]pyrimidine (100 mg, 1.0 eq, 0.5 mmol) in 1,4-dioxane (15 mL) was added 7-(3-pyridyl)-6-thia-2,3-diazabicyclo[3.3.0]octa-1,4,7-triene (100 mg, 1.0 eq, 0.5 mmol) followed by Cs_2CO_3 (485 mg, 3.0 eq, 1.49 mmol) in a sealed tube and degassed with argon gas for 10 min. After that, Pd_2dba_3 (45 mg, 0.1 eq, 0.05 mmol) was added followed by Xantphos (57.4 mg, 0.2 eq, 0.1 mmol) under argon atmosphere. The resulting mixture was heated to 120 °C for 16 h. The progress of the reaction was monitored by TLC. After completion, the reaction was filtered through celite pad and washed with EtOAc (10 mL). The filtrate was concentrated under reduced pressure to get crude. The crude product was purified by flash column chromatography, using 30% EtOAc/hexane as eluent to afford the compound. The obtained compound was re-purified by using prep-HPLC to afford (*R*)-2-(4-(3-fluoropyrrolidin-1-yl) pyrimidin-2-yl)-5-(pyridin-3-yl)-2*H*-thieno[3,2-*c*]pyrazole (31.5 mg, 17.33%) (**LMD-061**) as

an off white solid & (*R*)-1-(4-(3-fluoropyrrolidin-1-yl)pyrimidin-2-yl)-5-(pyridin-3-yl)-1*H*-thieno[3,2-*c*]pyrazole (**LMD-070**) (11.2 mg, 6.16%) as an off white solid.

LMD-061_Analytical data: ^{1}H NMR ($CDCl_3$, 400 MHz): δ 8.98 (s, 1H), 8.59 (d, *J* = 3.6 Hz, 1H), 8.31 (d, *J* = 6.0 Hz, 1H), 8.05 (s, 1H), 7.99 (s, 1H), 7.94 (d, *J* = 7.6 Hz, 1H), 7.41 - 7.38 (m, 1H), 6.24 (s, 1H), 5.46 (d, *J* = 52.4 Hz, 1H), 4.40 – 3.60 (m, 4H), 2.60 - 2.00 (m, 2H). ^{19}F NMR: -177.19 ppm. LCMS(ESI): m/z = 367.2 $[M+H]^+$. HPLC purity: 99.52%

LMD-070-Analytical data: ^{1}H NMR ($CDCl_3$, 400 MHz): δ 8.94 (s, 1H), 8.75 (s, 1H), 8.60 (d, *J* = 3.6 Hz, 1H), 8.28 (d, *J* = 5.6 Hz, 1H), 7.96 (d, *J* = 8.0 Hz, 1H), 7.43 (s, 1H), 7.39 - 7.36 (m, 1H), 6.29 (s, 1H), 5.43 (d, *J* = 54.0 Hz, 1H), 4.30 - 4.3.60 (m, 4H), 2.57 - 2.10 (m, 2H). ^{19}F NMR: -177.375 ppm. LCMS(ESI): m/z = 367.3 $[M+H]^+$. HPLC purity: 97.75%

LMD-068

Scheme:

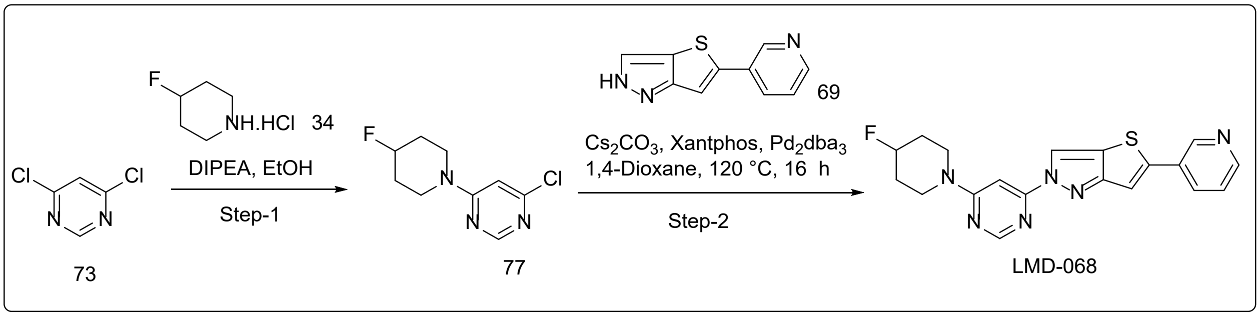

Step-1: 4-chloro-6-(4-fluoropiperidin-1-yl) pyrimidine (77):

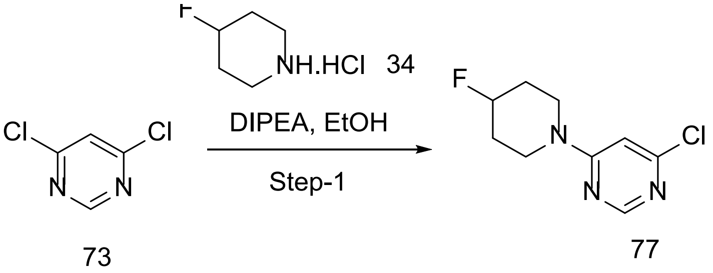

To a stirred solution of 2,4-dichloropyrimidine (1.0 g, 1.0 eq, 6.75 mmol) and 4-fluoropiperidine. Hydrogen chloride (0.94 g, 1.0 eq, 6.75 mmol) in EtOH (10 mL) was added *N*-ethyldiisopropylamine (2.6 g, 3.0 eq, 20.1 mmol) at 0 °C and allowed to stir at room temperature for 1 h. The progress of the reaction was monitored by TLC. After completion, concentrated under reduced pressure to get the crude. The crude was purified on silica gel using 30% EtOAc in hexane as eluent to afford 4-chloro-6-(4-fluoropiperidin-1-yl) pyrimidine (0.75 g, 51.81%) as a white

solid. ^{1}H NMR (DMSO-d_6, 400 MHz): δ 8.03 (d, J = 6.0 Hz, 1H), 6.42 (d, J = 6.4 Hz, 1H), 3.84 (br s, 2H), 3.67 - 3.60 (m, 2H), 1.99 - 1.80 (m, 4H). ^{19}F NMR: -183.56 ppm. MS (ESI) m/z: 216.17 $[M+H]^{+}$;

Step-2: Synthesis of 2-(6-(4-fluoropiperidin-1-yl) pyrimidin-4-yl)-5-(pyridin-3-yl)-2*H*-thieno[3,2-*c*] pyrazole (LMD-068):

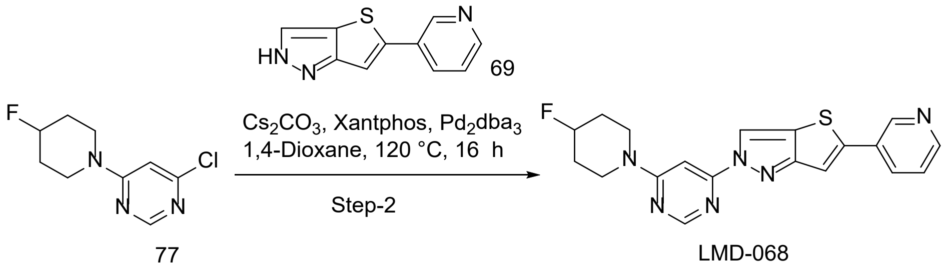

To a stirred solution of 4-chloro-6-(4-fluoropiperidin-1-yl)pyrimidine (100 mg, 1.0 eq, 0.465 mmol) and 7-(3-pyridyl)-6-thia-2,3-diazabicyclo[3.3.0]octa-1,4,7-triene (140 mg, 1.5 eq, 0.697 mmol) in 1,4-dioxane (4 mL) was added Cs_2CO_3 (453 mg, 3.0 eq, 1.39 mmol), and degassed with argon gas for 20 min. After that, Xantphos (53 mg, 0.2 eq, 0.09 mmol), was added followed by Pd_2dba_3 (42 mg, 0.1 eq, 0.04 mmol) and heated at 120 °C for 12 h. The reaction progress was monitored by TLC. After completion, the reaction mixture was filtered through celite pad and washed with EtOAc (10 mL). The filtrate was concentrated under reduced pressure to get the crude. The crude was purified by using silica gel column chromatography over silica gel using 2% MeOH/DCM as eluent to get the compound, which was re-purified by using prep-HPLC to afford 2-(6-(4-fluoropiperidin-1-yl) pyrimidin-4-yl)-5-(pyridin-3-yl)-2*H*-thieno[3,2-*c*] pyrazole (4 mg, 2.27%) as a white compound. ^{1}H NMR (DMSO-d_6, 400 MHz): δ 8.96 (s, 1H), 8.59 (d, J = 4.4 Hz, 1H), 8.30 (d, J = 6.4 Hz, 1H), 8.16 - 8.13 (m, 2H), 8.09 (s, 1H), 7.53 - 7.50 (m, 1H), 6.85 (d, J = 6.0 Hz, 1H), 4.96 (d, J = 49.2 Hz, 1H), 3.91 - 3.75 (m, 4H), 2.10 - 1.95 (m, 2H), 1.90 - 1.75 (m, 2H). ^{19}F NMR: -178.44 ppm. MS (ESI) m/z: 381.6 $[M+H]^{+}$. HPLC purity: 99.33%

LMD-069

Scheme:

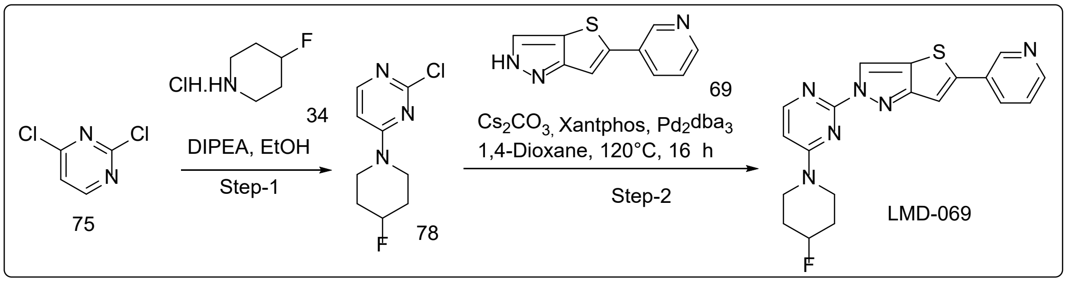

Step-1: Synthesis of 2-chloro-4-(4-fluoro-1-piperidyl) pyrimidine (78):

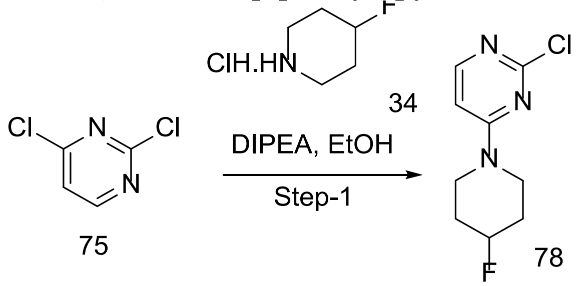

To a stirred solution of 2,4-dichloropyrimidine (1.0 g, 1.0 eq, 6.71 mmol) in EtOH (10 mL) was added 4-fluoropiperidine-hydrogen chloride (1/1) (0.93 g, 1.0 eq, 6.71 mmol) followed by DIPEA (3.5 mL, 3.0 eq, 20.1 mmol) at 0 °C and heated at 130 °C for 16 h. The progress of the reaction was monitored by TLC. After completion of the reaction, reaction was evaporated under reduced pressure to get the crude. The crude residue was purified on column chromatography by using 20% EtOAc/*n*-hexane as eluent to afford 2-chloro-4-(4-fluoro-1-piperidyl) pyrimidine (0.80 g, 55%) as a white solid. LCMS (ESI): m/z = 216.13 $[M+H]^+$.

Step-2: Synthesis of 2-(4-(4-fluoropiperidin-1-yl) pyrimidin-2-yl)-5-(pyridin-3-yl)-2*H*-thieno[3,2-*c*] pyrazole (LMD-069):

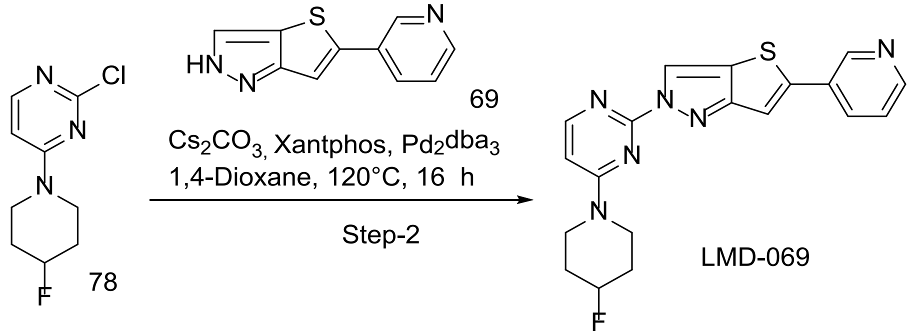

To a stirred solution of 2-chloro-4-(4-fluoro-1-piperidyl)pyrimidine (150 mg, 1.0 eq, 696 µmol) and 7-(3-pyridyl)-6-thia-2,3-diazabicyclo[3.3.0]octa-1,4,7-triene (210 mg, 1.5 eq, 1.04 mmol) in 1,4-dioxane (4 mL) was added Cs_2CO_3 (680 mg, 3.0 eq, 2.09 mmol), and purged with argon for 20 min. After that, Pd_2dba_3 (63.7 mg, 0.1 eq, 69.6 µmol) was added followed by Xantphos (80.5 mg, 0.2 eq, 139 µmol) under argon atmosphere and heated at 120 °C for 16 h. The progress of the reaction was monitored by TLC. After completion, the reaction mixture was filtered through celite pad and washed with EtOAc (2 x 10 mL). The filtrate was washed with water (2 x 10 mL) and brine solution (10 mL). The organic layer was dried over Na_2SO_4, and concentrated under reduced pressure to get crude. The crude residue was purified on column chromatography using 5%

MeOH/DCM as eluent to get the title compound, which was again re-purified by prep-HPLC to afford 2-(4-(4-fluoropiperidin-1-yl) pyrimidin-2-yl)-5-(pyridin-3-yl)-2*H*-thieno[3,2-*c*] pyrazole (4 mg, 1.5%) as an off white solid. ^{1}H NMR (DMSO-d_6, 400 MHz): 9.00 (d, *J* = 2.4 Hz, 1H), 8.61 (d, *J* = 4.8 Hz, 1H), 8.57 (s, 1H), 8.26 (s, 1H), 8.23 (s, 1H), 8.20 (d, *J* = 8.0 Hz, 1H), 7.53 - 7.50 (m, 1H), 7.18 (s, 1H), 4.96 (d, *J* = 52.4 Hz, 1H), 3.86 - 3.75 (m, 4H), 2.01 - 1.92 (m, 2H), 1.85 - 1.65 (m, 2H). ^{19}F NMR: -178.26 ppm. LCMS (ESI): *m/z* = 381.5 [M+H]$^+$. HPLC Purity: 95.79%

LMD-054

Scheme

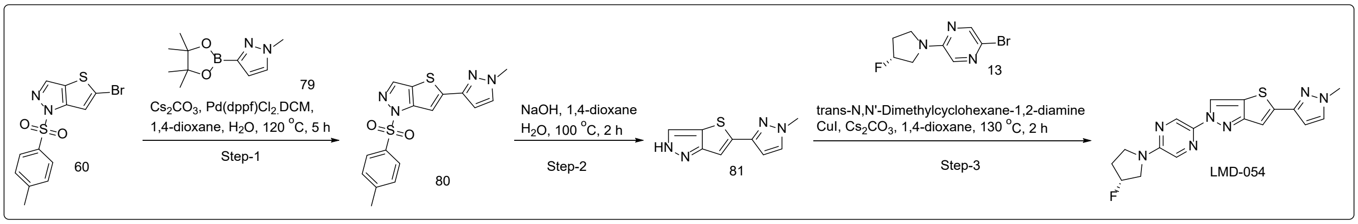

Step-1: **Synthesis of 5-(1-methyl-1*H*-pyrazol-3-yl)-1-tosyl-1*H*-thieno[3,2-*c*]pyrazole (80):**

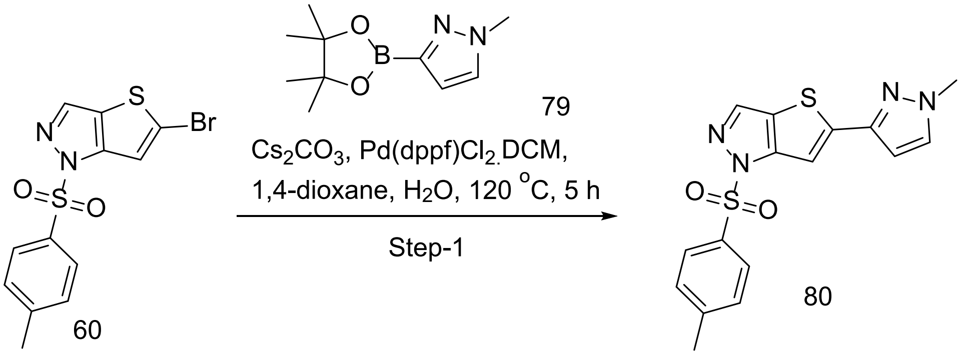

To a stirred solution 5-bromo-1-tosyl-1*H*-thieno[3,2-*c*]pyrazole (1.0 g, 1.0 eq, 2.8 mmol) and 1-methyl-3-(4,4,5,5-tetramethyl-1,3,2-dioxaborolan-2-yl)-1*H*-pyrazole (525 mg, 0.9 eq, 2.53 mmol), in 1,4-Dioxane (10 mL), H_2O (3 mL) was added Cs_2CO_3 (2.73 g, 3.0 eq, 8.43 mmol) at room temperature and purged with argon gas for 20 min. After that, Pd(dppf)Cl_2.DCM (114 mg, 0.05 eq, 0.14 mmol) was added under argon atmosphere and heated at 120 °C for 5 h. The reaction progress was monitored by TLC. After completion of the reaction, filtered through celite pad and washed with EtOAc (100 mL). The filtrate was washed with water (200 mL) and brines solution (100 mL). The organic layer was dried over Na_2SO_4, and concentrated under reduced pressure to get crude residue. The residue was purified on column chromatography using 10% EtOAc/hexane as eluent to afford 5-(1-methyl-1*H*-pyrazol-3-yl)-1-tosyl-1*H*-thieno[3,2-*c*]pyrazole (1.8 g, crude) as a gummy brown solid. LCMS (ESI): m/z = 359.2 [M+H]$^{+.}$

Step-2: Synthesis of 5-(1-methyl-1*H*-pyrazol-3-yl)-2*H*-thieno [3, 2-*c*] pyrazole (81):

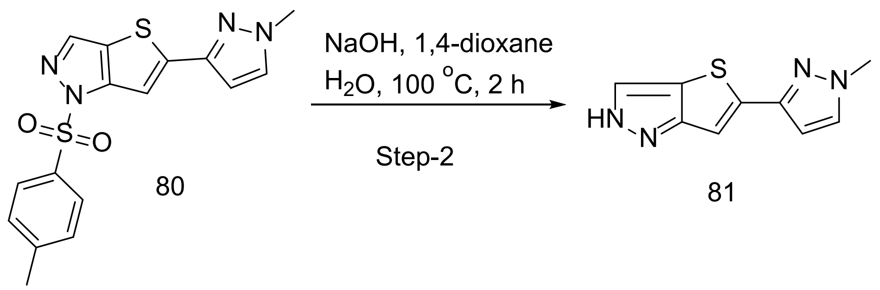

To a stirred solution of 5-(1-methyl-1*H*-pyrazol-3-yl)-1-tosyl-1*H*-thieno[3,2-*c*]pyrazole (1.8 g, 1.0 eq, 5.03 mmol) in 1,4-Dioxane (18 mL), water (18 mL) was added NaOH (603 mg, 3.0 eq, 15.08 mmol) at 0 °C and heated at 100 °C for 12 h. The progress of the reaction was monitored by TLC. After completion, the reaction was evaporated under reduced pressure to get the crude. The crude residue was purified on column chromatography using 10% MeOH/DCM as eluent to afford 5-(1-methyl-1*H*-pyrazol-3-yl)-2*H*-thieno [3, 2-*c*] pyrazole (650 mg, 63%) as an off white solid.

^{1}H NMR (DMSO-d_6, 400 MHz): δ 7.82 (br s, 1H), 7.75 (s, 1H), 7.57 - 7.48 (m, 1H), 7.41 (s, 1H), 6.72 (s, 1H), 3.86 (s, 1H). LCMS (ESI): m/z = 205.01 $[M+H]^{+.}$

Step-3: Synthesis of (*R*)-2-(5-(3-fluoropyrrolidin-1-yl) pyrazin-2-yl)-5-(1-methyl-1*H*-pyrazol-3-yl)-2*H*-thieno [3, 2-*c*] pyrazole (LMD-054):

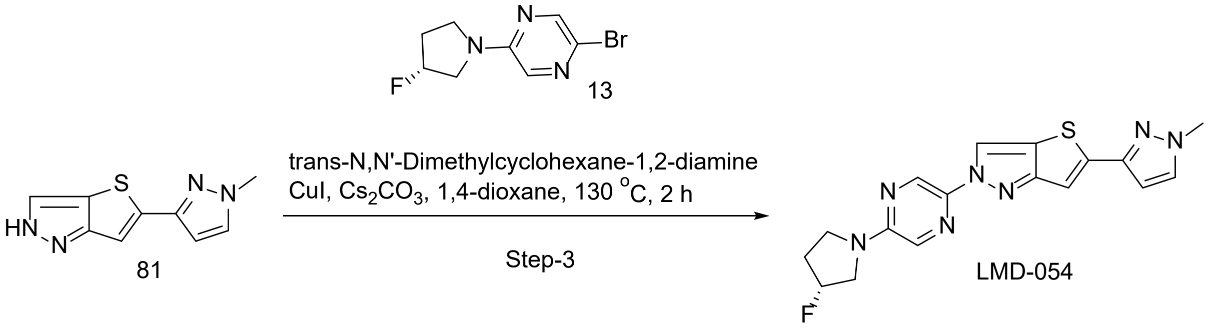

To a stirred solution of 5-(1-methyl-1*H*-pyrazol-3-yl)-2*H*-thieno[3,2-*c*] pyrazole (200 mg, 1.0 eq, 0.98 mmol) and (*R*)-2-bromo-5-(3-fluoropyrrolidin-1-yl) pyrazine (288 mg, 1.2 eq, 1.2 mmol) in 1,4-dioxane (2 mL) was added Cs_2CO_3 (955 mg, 3.0 eq, 2.94 mmol) and purged with argon for 10 min. After that, Cu(I)I (13.7 mg, 0.2 eq, 0.2 mmol) was added, followed by trans-*N*, *N*'-Dimethyl cyclohexane (27.83 mg, 0.2 eq, 0.2 mmol) and irradiated at 100 °C in MW for 1 h. The reaction progress was monitored by TLC. After completion of the reaction, filtered through celite pad, washed with EtOAc (25 mL). The filtrate was concentrated under reduced pressure to get crude. The crude residue was purified by column chromatography using 2% MeOH/DCM as eluent to

get desired compound, which was re-purified on prep-HPLC to afford (*R*)-2-(5-(3-fluoropyrrolidin-1-yl) pyrazin-2-yl)-5-(1-methyl-1*H*-pyrazol-3-yl)-2*H*-thieno [3,2-*c*] pyrazole (20 mg, 5.5%) as an off white solid. ^{1}H NMR (DMSO-d_6, 400 MHz): δ 8.69 (s, 1H), 8.04 (s, 1H), 7.96 (s, 1H), 7.83 (s, 1H), 7.79 (d, *J* = 1.6 Hz, 1H), 6.75 (d, *J* = 2.0 Hz, 1H), 5.45 (d, *J* = 52.8 Hz, 1H), 3.88 (s, 3H), 3.84 - 3.63 (m, 3H), 3.57 - 3.50 (m, 1H), 2.35 - 2.15 (m, 2H). ^{19}F NMR: -174.91 ppm LCMS (ESI): m/z = 370.3 $[M+H]^+$. HPLC purity: 99.66%

LMD-066

Scheme:

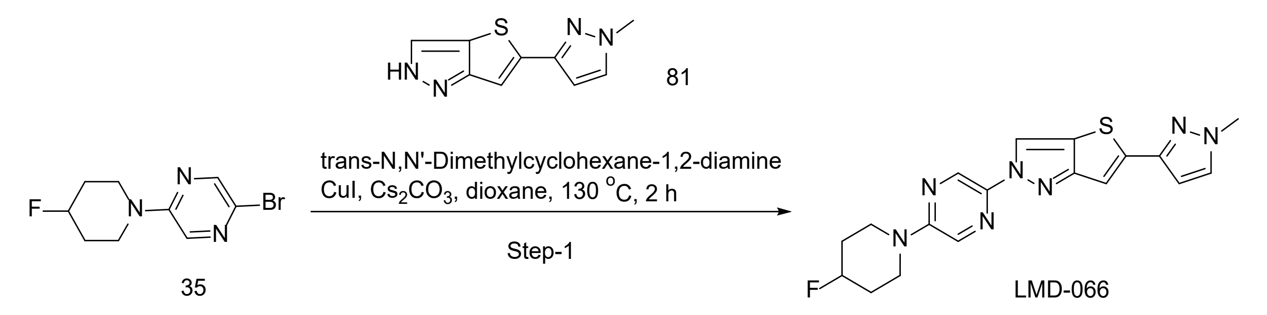

Step-1: 2-(5-(4-fluoropiperidin-1-yl) pyrazin-2-yl)-5-(1-methyl-1*H*-pyrazol-3-yl)-2H-thieno[3,2-*c*] pyrazole (LMD-066):

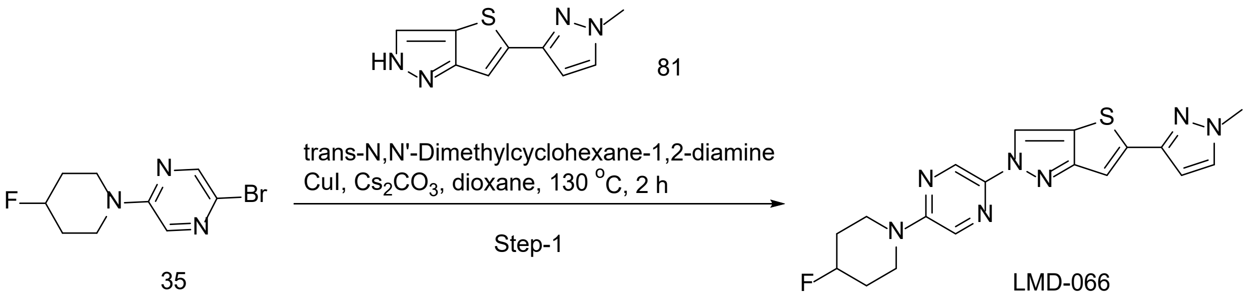

To a solution of 5-(1-methyl-1*H*-pyrazol-3-yl)-2*H*-thieno[3,2-*c*] pyrazole (120 mg, 1.0 eq, 0.58 mmol) and 2-bromo-5-(4-fluoropiperidin-1-yl) pyrazine (228 mg, 1.5 eq, 0.89 mmol) in 1,4-dioxane was added Cs_2CO_3 (573 mg, 3.0 eq, 1.76 mmol) and purged with argon gas for 10 min. After that, CuI (6 mg, 0.05 eq, 0.03 mmol) was added followed by (*1R*,*2R*)-1,2-bis(methylamino)cyclohexane (22 mg, 0.2 eq, 0.12 mmol) under argon atmosphere and irradiated at 130 °C for 2 h in microwave. The progress of the reaction was monitored by TLC. After completion, filtered through celite pad and washed with EtOAc (10 mL). The filtrate was concentrated to get the crude, which was purified on silica gel using 2% MeOH/DCM as eluent to get the compound, which was re-purified by using prep-HPLC to afford 2-(5-(4-fluoropiperidin-1-yl) pyrazin-2-yl)-5-(1-methyl-1*H*-pyrazol-3-yl)-2*H*-thieno[3,2-*c*] pyrazole (16 mg, 9.04%) as an

off-white color solid. ^{1}H NMR (DMSO-d_6, 400 MHz): δ 8.70 (s, 1H), 8.32 (s, 1H), 8.06 (s, 1H), 7.82 - 7.79 (m, 2H), 6.74 (d, *J* = 2.0 Hz, 1H), 5.02 - 4.85 (d, *J* = 53.2 Hz, 1H), 3.88 (s, 3H), 3.82 - 3.78 (m, 2H), 3.62 - 3.58 (m, 2H), 2.07 - 1.92 (m, 2H), 1.80 - 1.73 (m, 2H). ^{19}F NMR: -177.34 ppm. MS (ESI): m/z = 384.1 [M+H]$^+$. HPLC purity: 99.01%

LMD-067 & 072

Scheme:

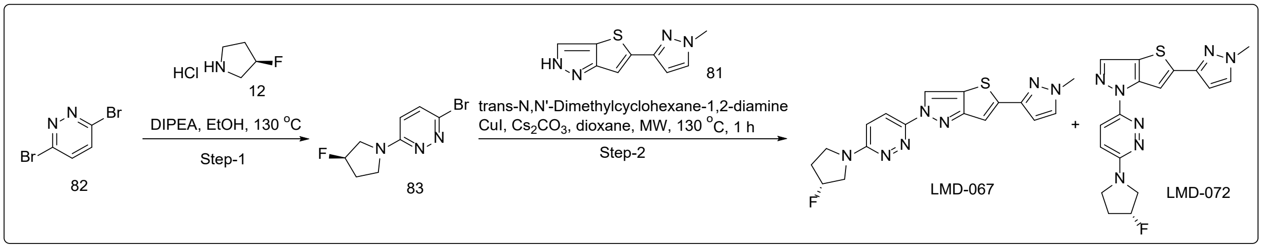

Step-1: Synthesis of (*R*)-3-bromo-6-(3-fluoropyrrolidin-1-yl) pyridazine (83):

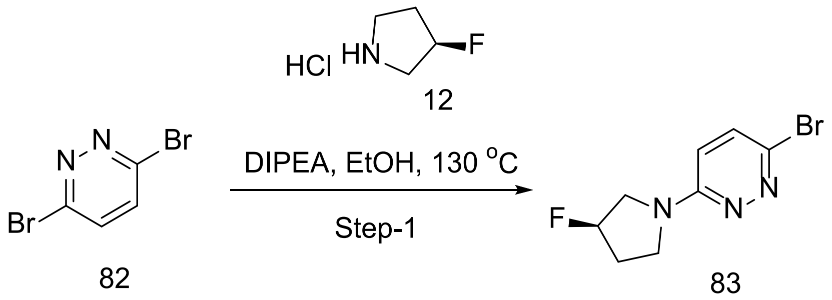

To stirred solution of 3,6-dibromopyridazine (1.0 g, 1.0 eq, 4.2 mmol), in EtOH (10 mL) in a seal tube was added (*R*)-3-fluoropyrrolidine. HCl (1.06 g, 2.0 eq, 8.41 mmol), followed by *N*-diisopropylethylamine (2.72 g, 5.0 eq, 21.0 mmol) at room temperature and heated at 130 °C for 16 h. The reaction progress was monitored by TLC. After completion of the reaction, the solvent was removed under reduced pressure to get crude. The crude residue was taken up in DCM (40 mL), washed with ammonium chloride solution (20 mL) and water (20 mL). The organic layer was dried over Na_2SO_4, and concentrated under reduced pressure to get residue. The residue was purified on column chromatography using 30%EtOAc/hexane as eluent to afford the (*R*)-3-bromo-6-(3-fluoropyrrolidin-1-yl) pyridazine (0.80 g, 77.3 %) as an off yellow solid. ^{1}H NMR (DMSO-d_6, 400 MHz): δ 7.60 (d, *J* = 9.6 Hz, 1H), 6.96 (d, *J* = 9.6 Hz, 1H), 5.48 (d, *J* = 53.2 Hz, 1H), 3.85 - 3.40 (m, 4H), 2.40 - 2.10 (m, 2H). MS (ESI): m/z = 246 [M]$^+$

Step-2: Synthesis of (*R*)-2-(6-(3-fluoropyrrolidin-1-yl) pyridazin-3-yl)-5-(1-methyl-1*H*-pyrazol-3-yl)-2*H*-thieno[3,2-*c*] pyrazole (LMD-067) and (*R*)-1-(6-(3-fluoropyrrolidin-1-yl) pyridazin-3-yl)-5-(1-methyl-1*H*-pyrazol-3-yl)-1*H*-thieno[3,2-*c*] pyrazole (LMD-072):

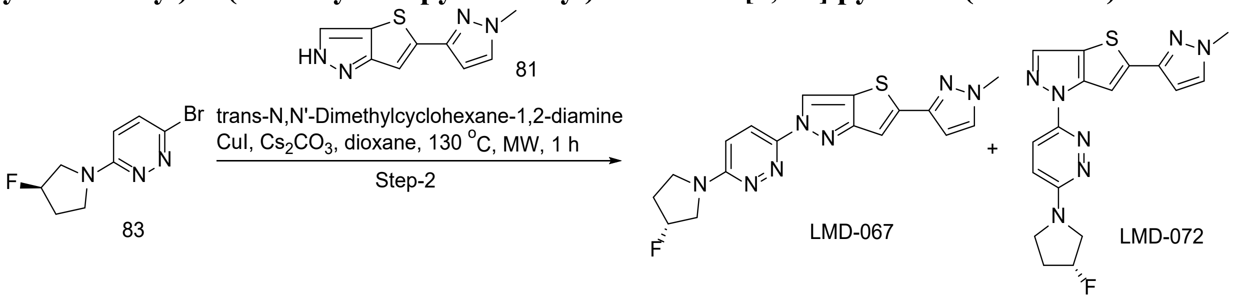

To a stirred solution of 5-(1-methyl-1*H*-pyrazol-3-yl)-2*H*-thieno[3,2-*c*] pyrazole (100 mg, 1.0 eq, 0.49 mmol) and (*R*)-3-bromo-6-(3-fluoropyrrolidin-1-yl) pyridazine (144 mg, 1.2 eq, 0.59 mmol) in 1,4-Dioxane (4 mL) was added Cs_2CO_3 (479 mg, 3.0 eq, 1.47 mmol) and purged with argon gas 10 min. After that, trans-*N*, *N*'-dimethyl cyclohexane (14 mg, 0.2 eq, 0.01 µmol) was added followed by CuI (19.4 mg, 0.2 eq, 0.01 mmol) and irradiated to 130 °C in MW for 1 h. The reaction progress was monitored by TLC. After completion of the reaction, filtered through celite pad, and washed with EtOAc (20 mL). The filtrate was concentrated under reduced pressure to get crude. The crude residue was purified by flash column chromatography, using 5% MeOH/DCM as eluent to get crude compound, which was re-purified on prep-HPLC to afford (*R*)-2-(6-(3-fluoropyrrolidin-1-yl)pyridazin-3-yl)-5-(1-methyl-1*H*-pyrazol-3-yl)-2*H*-thieno[3,2-*c*]pyrazole (**LMD-067)** (26 mg, 14%) as an off white solid and (*R*)-1-(6-(3-fluoropyrrolidin-1-yl)pyridazin-3-yl)-5-(1-methyl-1*H*-pyrazol-3-yl)-1*H*-thieno [3,2-*c*] pyrazole **(LMD-072)** (7.8 mg, 4.3%) as an off white solid. **LMD-067:** ^{1}H NMR (DMSO-d_6, 400 MHz): δ 8.10 (s, 1H), 8.01 (d, *J* = 9.6 Hz, 1H), 7.95 (s, 1H), 7.80 (d, *J* = 2.0 Hz, 1H), 7.24 (d, *J* = 9.6 Hz, 1H), 6.87 (d, *J* = 2.0 Hz, 1H), 5.55 (d, *J* = 53.6 Hz, 1H), 3.90 (s, 3H), 3.80 - 3.61 (m, 4H), 2.38 - 2.18 (m, 2H).^{19}F NMR: -174.83 ppm
MS (ESI): m/z = 370.3 $[M+H]^+$
HPLC purity: 96.81%

LMD-072: ^{1}H NMR (DMSO-d_6, 400 MHz): δ 8.84 (s, 1H), 8.02 (d, *J* = 9.2 Hz, 1H), 7.80 (s, 1H), 7.52 (s, 1H), 7.22 (d, *J* = 9.6 Hz, 1H), 6.80 (d, *J* = 2.0 Hz, 1H), 5.51 (d, *J* = 52.8 Hz, 1H), 3.89 (s, 3H), 3.88 - 3.66 (m, 3H), 3.58 - 3.51 (m, 1H), 2.32 - 2.07 (m, 2H). ^{19}F NMR: -174.98 ppm
MS (ESI): m/z = 370.2 $[M+H]^+$. HPLC purity: 95.17%

LMD-074, 077

Scheme:

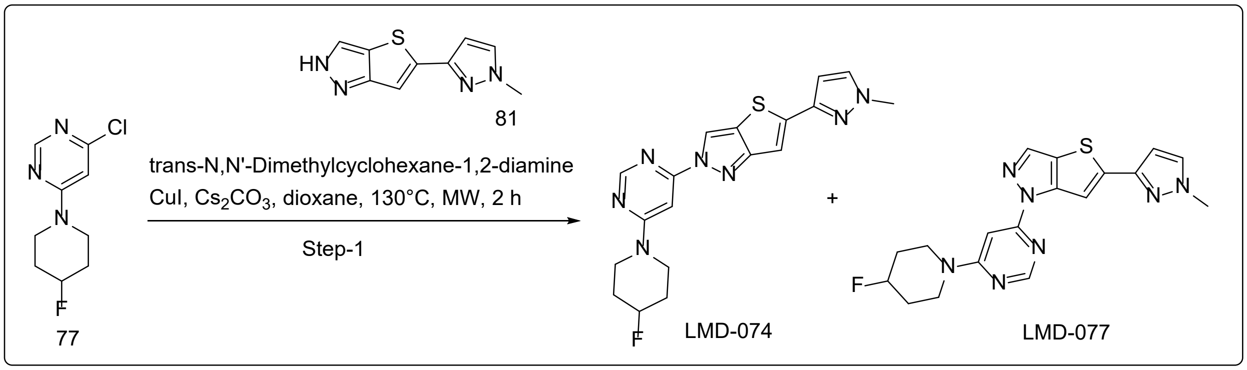

Step-1: Synthesis of 2-(6-(4-fluoropiperidin-1-yl) pyrimidin-4-yl)-5-(1-methyl-1*H*-pyrazol-3-yl)-2*H*-thieno[3,2-*c*] pyrazole (LMD-074) and 1-(6-(4-fluoropiperidin-1-yl) pyrimidin-4-yl)-5-(1-methyl-1*H*-pyrazol-3-yl)-1*H*-thieno[3,2-*c*] pyrazole (LMD-077):

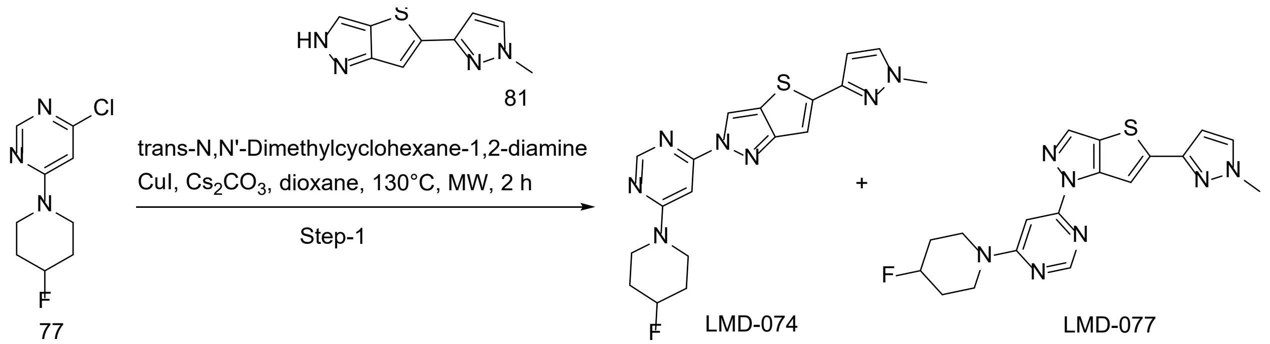

To a stirred solution of 5-(1-methyl-1*H*-pyrazol-3-yl)-2*H*-thieno[3,2-*c*] pyrazole (100 mg, 1.0 eq, 0.49 mmol) and 4-chloro-6-(4-fluoropiperidin-1-yl) pyrimidine (126 mg, 1.2 eq, 0.59 mmol) in 1,4-dioxane (5 mL) was added Cs_2CO_3 (477 mg, 3.0 eq, 1.47 mmol), and degassed with argon gas for 10 min. After that, trans-*N*, *N*'-dimethyl cyclohexane (14 mg, 0.2 eq, 0.1 mmol) was added followed by Cu(I)I (5 mg, 0.05 eq, 0.024 mmol) under argon atmosphere and irradiated under microwave at 130 °C for 2 h. The reaction progress was monitored by TLC. After completion of the reaction, filtered through celite pad, and washed with EtOAc (10 mL). The filtrate was concentrated under reduced pressure to get crude. The crude residue was repurified by prep-HPLC to afford 2-(6-(4-fluoropiperidin-1-yl) pyrimidin-4-yl)-5-(1-methyl-1*H*-pyrazol-3-yl)-2*H*-thieno[3,2-*c*] pyrazole (**LMD-074**) (8 mg, 4.26% yield) as an off white solid and 1-(6-(4-fluoropiperidin-1-yl) pyrimidin-4-yl)-5-(1-methyl-1*H*-pyrazol-3-yl)-1*H*-thieno[3,2-*c*] pyrazole (**LMD-077**) (18 mg, 9.59%) as a pale-yellow solid.

LMD-074:

^{1}H NMR (DMSO-d_6, 400 MHz): δ 8.29 (d, *J* = 6.0 Hz, 1H), 8.07 (s, 1H), 7.86 (s, 1H), 7.79 (d, *J* = 2.0 Hz, 1H), 6.83 (d, *J* = 6.0 Hz, 1H), 6.71 (d, *J* = 2.4 Hz, 1H), 5.00 (d, *J* = 65.2 Hz, 1H), 3.88 (s, 3H), 3.87 - 3.77 (m, 4H), 2.04 - 1.93 (m, 2H), 1.82 - 1.74 (m, 2H). ^{19}F NMR: -178.43 ppm

LCMS (ESI): m/z = 384.3 $[M+H]^+$

HPLC purity: 98.73%.

LMD-077:

^{1}H NMR (DMSO-d_6, 400 MHz): δ 8.86 (s, 1H), 8.24 (d, *J* = 6.0 Hz, 1H), 7.80 (d, *J* = 2.0 Hz, 1H), 7.50 (s, 1H), 6.85 (d, *J* = 6.4 Hz, 1H), 6.80 (d, *J* = 2.4 Hz, 1H), 5.00 (m, 1H), 3.88 (s, 3H), 3.87 - 3.78 (m, 4H), 1.99 - 1.90 (m, 2H), 1.84 - 1.60 (m, 2H). ^{19}F NMR: -178.42 ppm

LCMS (ESI): m/z = 384.6 $[M+H]^+$

HPLC purity: 98.60%

LMD-075 & 076

Scheme:

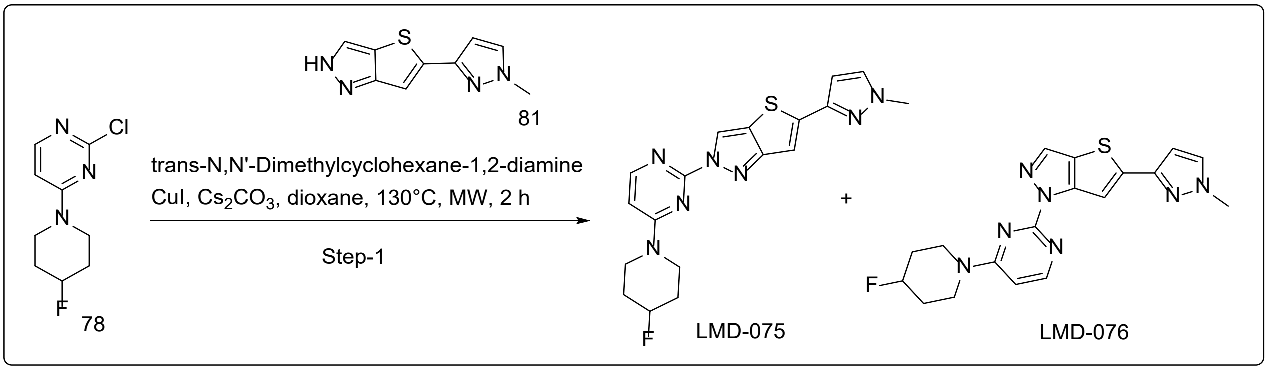

Step-1: Synthesis of 2-(4-(4-fluoropiperidin-1-yl) pyrimidin-2-yl)-5-(1-methyl-1*H*-pyrazol-3-yl)-2*H*-thieno[3,2-*c*] pyrazole (LMD-075) and 1-(4-(4-fluoropiperidin-1-yl) pyrimidin-2-yl)-5-(1-methyl-1*H*-pyrazol-3-yl)-1*H*-thieno[3,2-*c*] pyrazole (LMD-076):

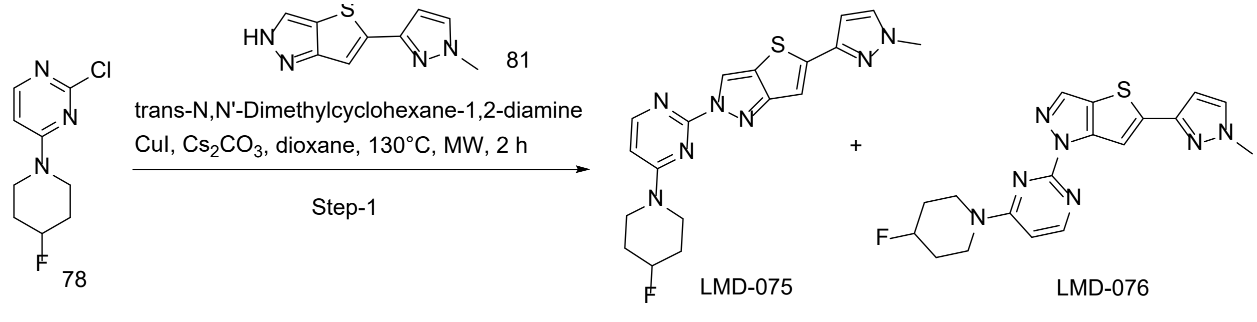

To a stirred solution of 5-(1-methyl-1*H*-pyrazol-3-yl)-2*H*-thieno[3,2-*c*] pyrazole (100 mg, 1.0 eq, 0.49 mmol) and 2-chloro-4-(4-fluoropiperidin-1-yl) pyrimidine (126 mg, 1.2 eq, 0.59 mmol) in 1,4-dioxane (5 mL) was added Cs_2CO_3 (477 mg, 3.0 eq, 1.47 mmol), and degassed with argon gas for 10 min. After that, trans-*N*, *N*'-dimethyl cyclohexane (13.91 mg, 0.2 eq, 0.1 mmol) was added followed by Cu(I)I (5 mg, 0.05 eq, 0.024 mmol) under argon atmosphere and irradiated under microwave at 130 °C for 2 h. The reaction progress was monitored by TLC. After completion of the reaction, filtered through celite pad, and washed with EtOAc (10 mL). The filtrate was concentrated under reduced pressure to get crude. The crude residue was purified by prep-HPLC to afford 2-(4-(4-fluoropiperidin-1-yl)pyrimidin-2-yl)-5-(1-methyl-1*H*-pyrazol-3-yl)-2*H*-thieno[3,2-*c*]pyrazole (**LMD-075**) (13.61 mg, 6.92%) as an off white solid and 1-(4-(4-fluoropiperidin-1-yl)pyrimidin-2-yl)-5-(1-methyl-1*H*-pyrazol-3-yl)-1*H*-thieno[3,2-*c*]pyrazole (**LMD-076**) (7.02 mg, 3.74%) as an off white solid.

LMD-075:

^{1}H NMR (DMSO-*d*$_6$, 400 MHz): δ 8.82 (s, 1H), 8.47 (s, 1H), 7.81 (d, *J* = 2.4 Hz, 1H), 7.48 (s, 1H), 7.23 (s, 1H), 6.82 (d, *J* = 2.4 Hz, 1H), 4.95 (d, *J* = 49.2 Hz, 1H), 3.89 (s, 3H), 3.83 (m, , 2H), 3.80 – 3.40 (m, 4H), 2.01 - 1.91 (m, 2H), 1.85 - 1.75 (m, 2H).

^{19}F NMR: -178.33 ppm

LCMS (ESI): m/z = 384.3 $[M+H]^{+.}$

HPLC purity: 99.62%.

LMD-076:

^{1}H NMR (DMSO-*d*$_6$, 400 MHz): δ 8.55 (s, 1H), 8.16 (s, 1H), 7.97 (s, 1H), 7.79 (d, *J* = 2.4 Hz, 1H), 7.15 (s, 1H), 6.79 (d, *J* = 2.4 Hz, 1H), 4.95 (d, *J* = 54.4 Hz, 1H), 3.88 (s, 3H), 3.87 - 3.81 (m, 2H), 3.80 - 3.70 (m, 2H), 2.07 - 1.90 (m, 2H), 1.85 - 1.75 (m, 2H).

^{19}F NMR: -178.24 ppm

LCMS (ESI): m/z = 384.5 $[M+H]^+$

HPLC purity: 99.51%.

2 *In vitro* assays

2.1 Preliminary 2-point screening assay

Table S2.1 Results of the 2-point screening assays of competing compounds at 300 nM and 30 nM *vs*. [^{3}H]asyn-44. The value in the table represents the % [^{3}H]asyn-44 remaining. Blue text depicts leads selected for K_i determination (Table S2.2).

Compounds	30 nM	300 nM
LMD-001	72	39
LMD-002	57	21
LMD-005	112	64
LMD-006	53	2
LMD-009	45	17
LMD-013	87	58
LMD-014	45	20
LMD-015	46	21
LMD-016	91	48
LMD-017	43	25
LMD-019	66	27
LMD-022	40	15
LMD-023	99	50
LMD-024	72	13
LMD-026	56	25
LMD-027	69	34
LMD-028	81	38
LMD-029	34	12
LMD-031	66	24
LMD-032	38	25
LMD-033	51	33
LMD-034	73	28
LMD-036	65	23
LMD-039	57	27
LMD-040	54	28
LMD-041	77	13
LMD-044	38	4
LMD-045	36	13
LMD-046	43	8
LMD-049	89	35
LMD-051	38	4
LMD-052	27	2
LMD-054	58	15
LMD-060	48	29
LMD-061	56	29
LMD-062	43	32
LMD-063	64	33
LMD-064	46	26
LMD-066	76	51
LMD-067	86	28
LMD-068	50	34
LMD-069	49	31
LMD-070	43	22
LMD-072	48	33
LMD-073	71	29

**LMD-074**	55	45
**LMD-075**	41	19
**LMD-076**	64	22
**LMD-077**	67	20

2.2 *K*i Determination

Table S2.2 Inhibition constant (K_i) values *vs*. [^{3}H]asyn-44 of selected compounds.

**Compound**	**K_i (nM)**
**LMD-006**	12
**LMD-022**	16
**LMD-024**	54
**LMD-029**	13
**LMD-041**	58
**LMD-044**	9.1
**LMD-045**	13
**LMD-046**	12
**LMD-051**	9.2
**LMD-052**	6
**LMD-054**	24

2.3 Summary of Structural Variants and Their 2-Point Screening and K_i Profiles

Table S2.3.1 Summary of 2-point screening and K_i assay results for common pyridinone scaffold variants.

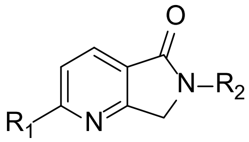

**Comp.**	**R$_1$**	**R$_2$**	**30nM**	**300nM**	**K_i**	**$t_{1/2}$**
LMD-016	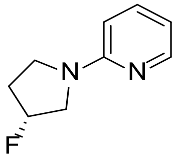	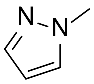	91%	48%	/	/
LMD-017	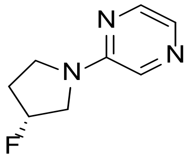	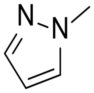	43%	25%	/	/

LMD-019	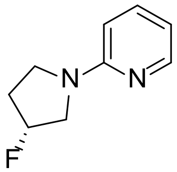	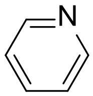	66%	27%	/	/
**LMD-022**	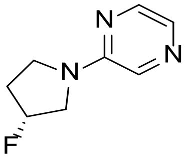	H	40%	15%	16 nM	75 mins
LMD-023	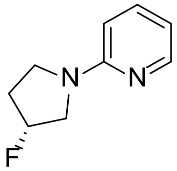	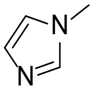	99%	50%	/	/
LMD-064	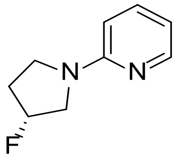	H	46%	26%	/	/

Table S2.3.2 Summary of 2-point screening and K_i assay results for common pyrazole scaffold variants.

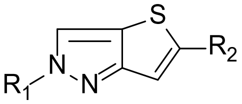

**Comp.**	**R₁**	**R₂**	**30nM**	**300nM**	**K_i**	**$t_{1/2}$**
LMD-028	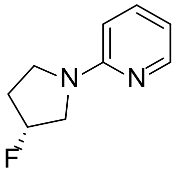	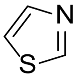	81%	38%	/	/
**LMD-029**	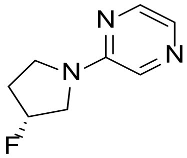	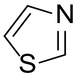	34%	12%	13 nM	/
LMD-031	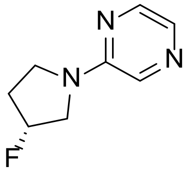	H	66%	24%	/	/

LMD-032	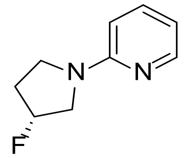	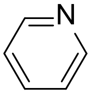	38%	25%	/	7.05 mins
LMD-033	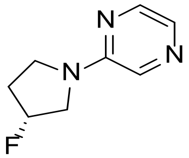	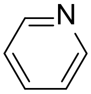	51%	33%	/	/
LMD-034	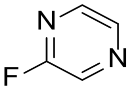	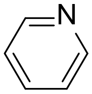	73%	28%	/	/
**LMD-046**	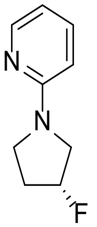	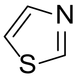	43%	8%	12 nM	unstable
**LMD-051**	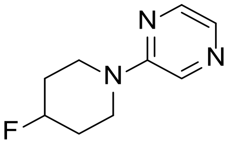	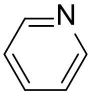	38%	4%	9.2 nM	17 mins
**LMD-052**	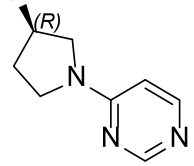	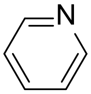	27%	2%	6 nM	7.6 mins
LMD-068	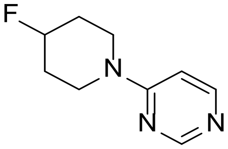	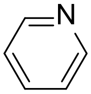	50%	34%	/	/
LMD-073	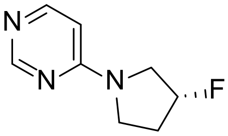	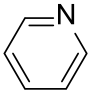	71%	29%	/	/

Table S2.3.3 Summary of 2-point screening and K_i assay results for common pyridine scaffold variants.

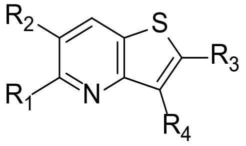

Comp.	R_1	R_2	R_3	R_4	30nM	300nM	K_i	$t_{1/2}$
LMD-001	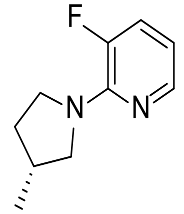	H	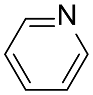	H	72%	39%	/	/
LMD-002	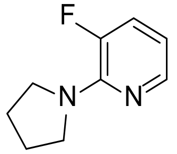	H	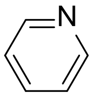	H	57%	21%	/	/
LMD-005	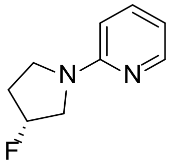	H	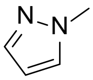	H	112 %	64%	/	/
**LMD-006**	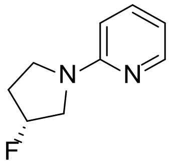	H	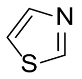	H	53%	2%	12 nM	6.81 mins
LMD-009	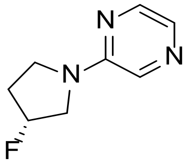	H	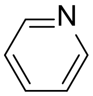	H	45%	17%	/	/
LMD-011	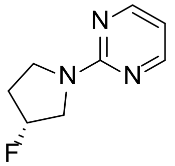	H	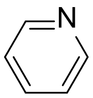	H	/	/	/	/
LMD-013	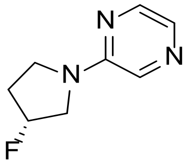	H	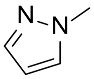	H	87%	58%	/	/
LMD-014	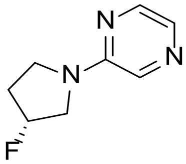	H	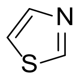	H	45%	20%	/	/
LMD-015	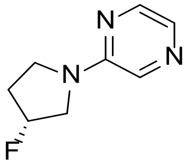	H	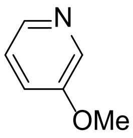	H	46%	21%	/	/

**LMD-024**	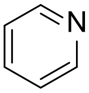	H	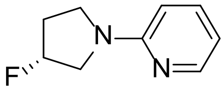	H	72%	13%	54 nM	/
LMD-026	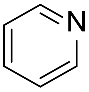	H	H	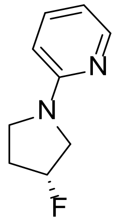	56%	25%	/	/
LMD-027	H	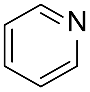	H		69%	34%	/	/
LMD-036	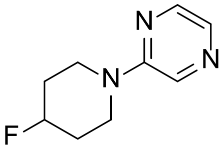	H	H	H	65%	25%	/	/
LMD-039	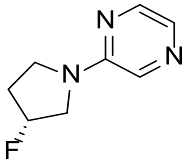	H	H	H	57%	27%	/	/
**LMD-041**	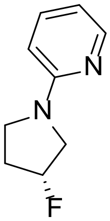	H	H	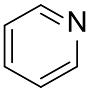	77%	13%	58 nM	/
**LMD-044**	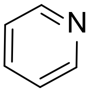	H	H	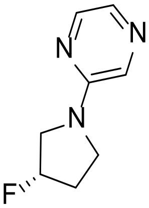	38%	4%	9.1 nM	6.37 mins
**LMD-045**	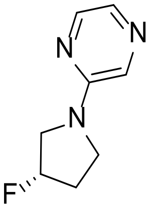	H	H	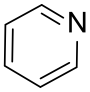	36%	13%	13 nM	10.5 mins
LMD-049	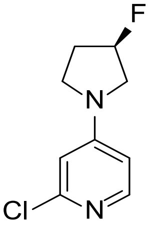	H	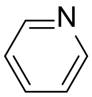	H	89%	35%	/	/
LMD-060	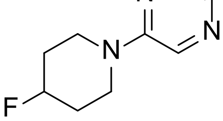	H	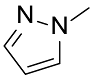	H	48%	29%	/	/

LMD-062	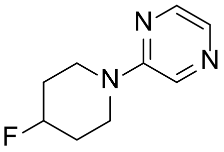	H	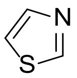	H	43%	32%	/	/
LMD-063	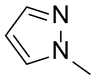	H	H	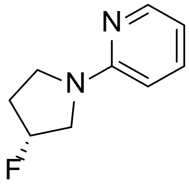	64%	33%	/	/

3 Chromatographic Characteristics of LMD compounds

3.1 Chromatographic Analysis of Metabolic Stability of LMD Compounds in Human Liver Microsomes

Figure S3.1. Representative chromatograms showing **LMD-006** (top, RT = 1.946 min) and internal standard (bottom, RT = 1.834 min) from human liver microsome stability assays. Quantification was performed using peak area ratios, with LMD-006 showing a progressive decrease in signal over time. The compound exhibited 60.2%, 88.1%, and 95.7% disappearance at 5, 15, and 30 minutes, respectively, relative to time zero.

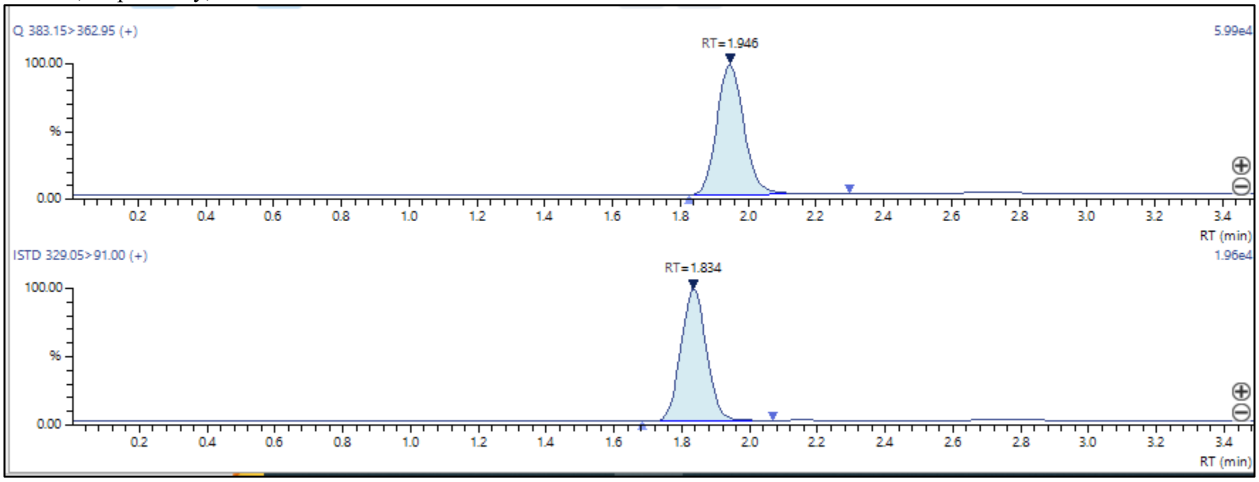

Figure S3.2. Representative chromatograms showing **LMD-022** (top, RT = 2.035 min) and internal standard (bottom, RT = 1.834 min) from human liver microsome stability assays. Quantification was based on peak area ratios, with LMD-022 showing minimal degradation over time. The compound exhibited 7.6%, 17.8%, and 24.6% disappearance at 5, 15, and 30 minutes, respectively, relative to time zero.

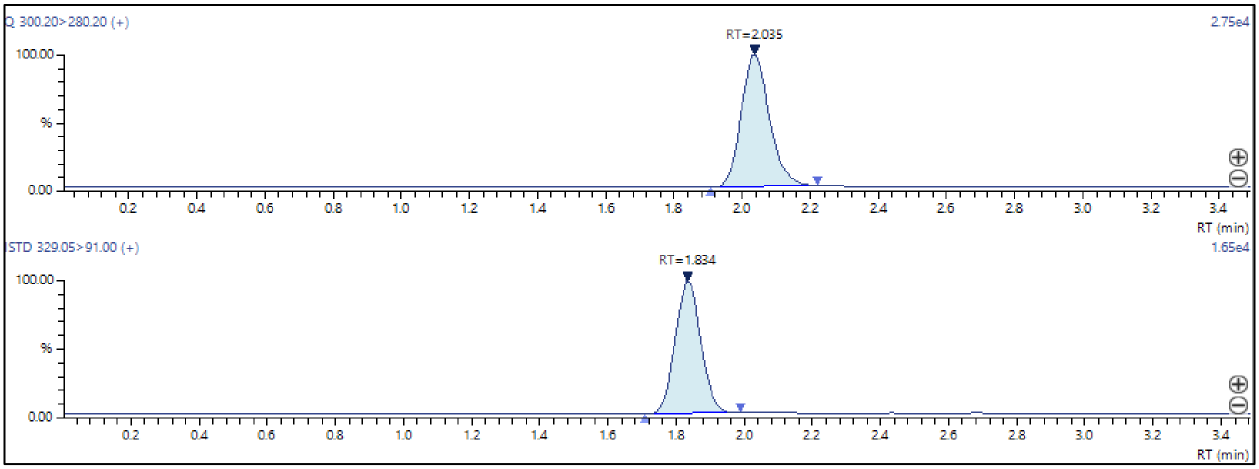

Figure S3.3. Representative chromatograms showing **LMD-032** (top, RT = 1.831 min) and internal standard (bottom, RT = 1.545 min) from human liver microsome stability assays. Quantification was performed using area ratios, with LMD-032 showing a time-dependent decrease in signal. The compound exhibited a 46.7%, 79.2%, and 95.0% disappearance at 5, 15, and 30 minutes, respectively, relative to time zero.

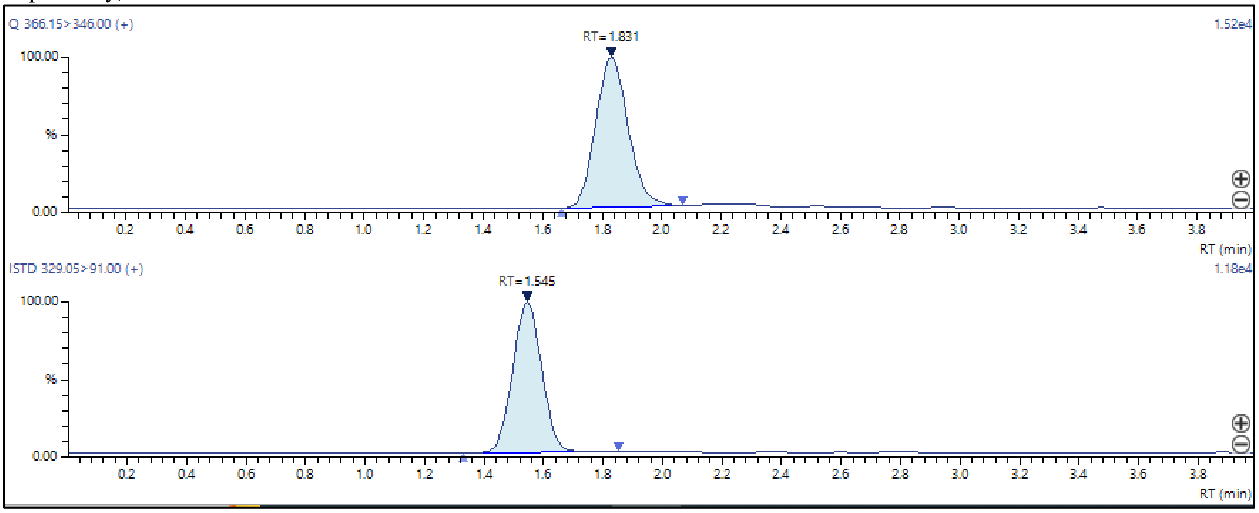

Figure S3.4. Representative chromatograms showing **LMD-044** (top, RT = 2.019 min) and internal standard (bottom, RT = 1.540 min) from human liver microsome stability assays. Quantification was performed using peak area ratios. LMD-044 showed significant degradation over time, with 54.3%, 88.3%, and 96.3% disappearance at 5, 15, and 30 minutes, respectively, relative to the time zero.

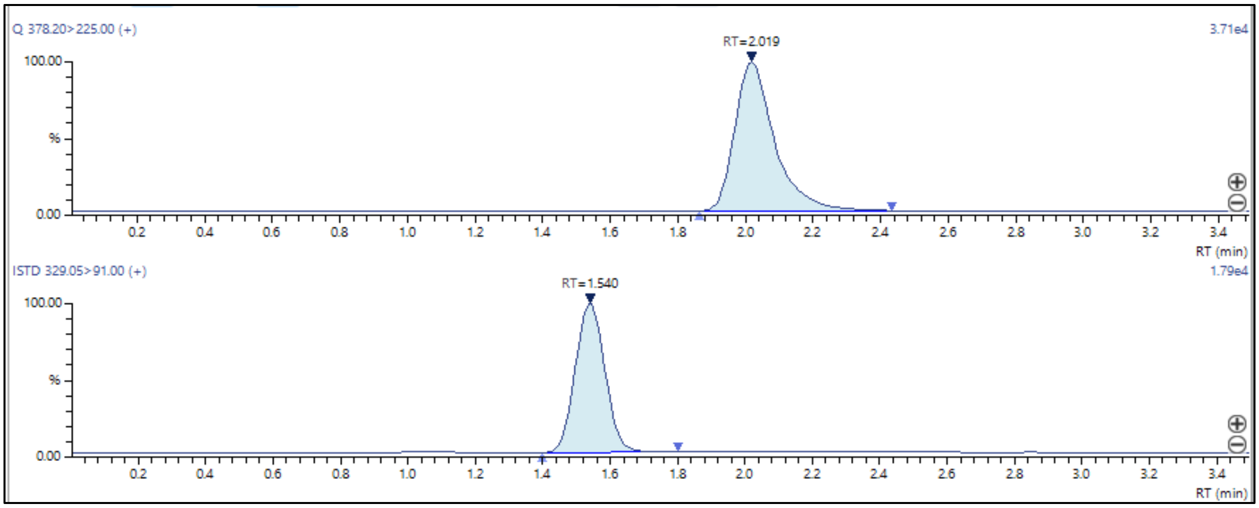

Figure S3.5. Representative chromatograms showing **LMD-045** (top, RT = 2.171 min) and internal standard (bottom, RT = 1.833 min) from human liver microsome stability assays. Area ratios were used to calculate the percentage of parent compound remaining over time. LMD-045 showed 34.7%, 65.6%, and 86.5% disappearance at 5, 15, and 30 minutes, respectively, relative to time zero.

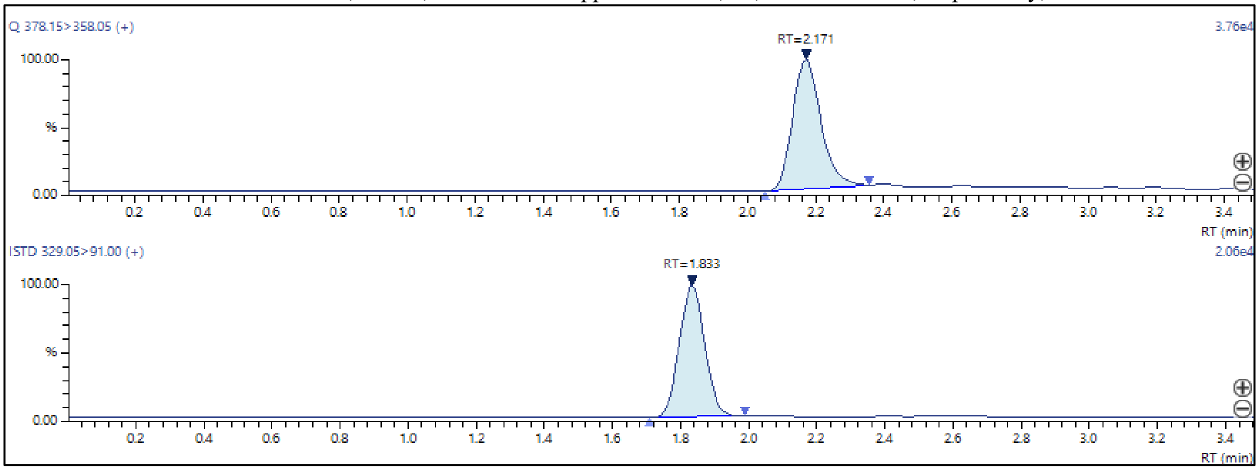

Figure S3.6. Representative chromatograms showing **LMD-046** (top, RT = 1.969 min) and internal standard (bottom, RT = 1.543 min) from human liver microsome stability assays. Area ratios were used to quantify compound stability over time. LMD-046 showed rapid metabolic degradation, with 96.6% and 97.9% disappearance at 5 and 15 minutes, respectively, relative to time zero.

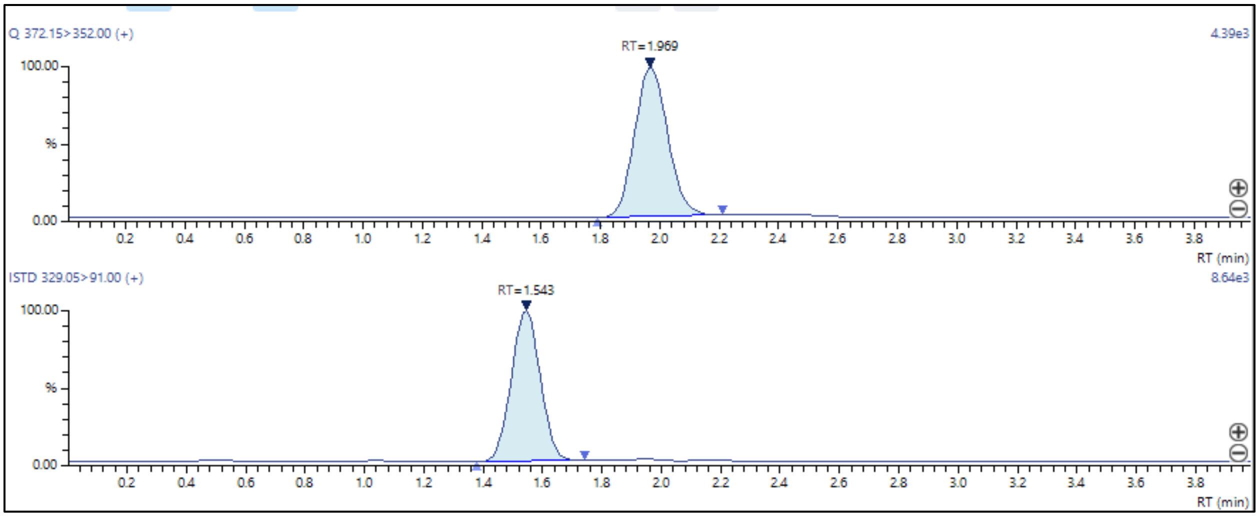

Figure S3.7. Representative chromatograms showing LMD-051 (top, RT = 2.577 min) and internal standard (bottom, RT = 1.543 min) from human liver microsome stability assays. Peak area ratios were used to monitor compound degradation over time. LMD-051 demonstrated moderate metabolic clearance, with 24.2%, 64.3%, and 69.7% disappearance at 5, 15, and 30 minutes, respectively, relative to time zero.

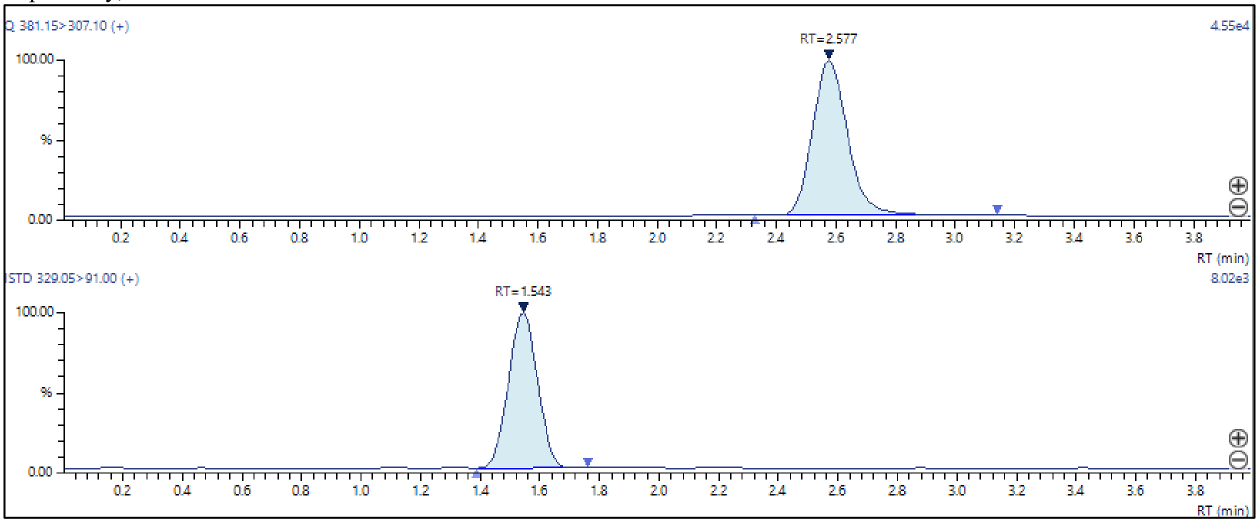

Figure S3.8. LC–MS/MS chromatograms of LMD-052 and internal standard following microsomal incubation. Representative chromatograms showing LMD-052 (top, RT = 2.345 min) and internal standard (bottom, RT = 1.539 min) from human liver microsome stability assays. Area ratios were calculated at various time points to assess metabolic degradation. LMD-052 exhibited 60.3%, 82.8%, and 94.4% disappearance at 5, 15, and 30 minutes, respectively, relative to time zero.

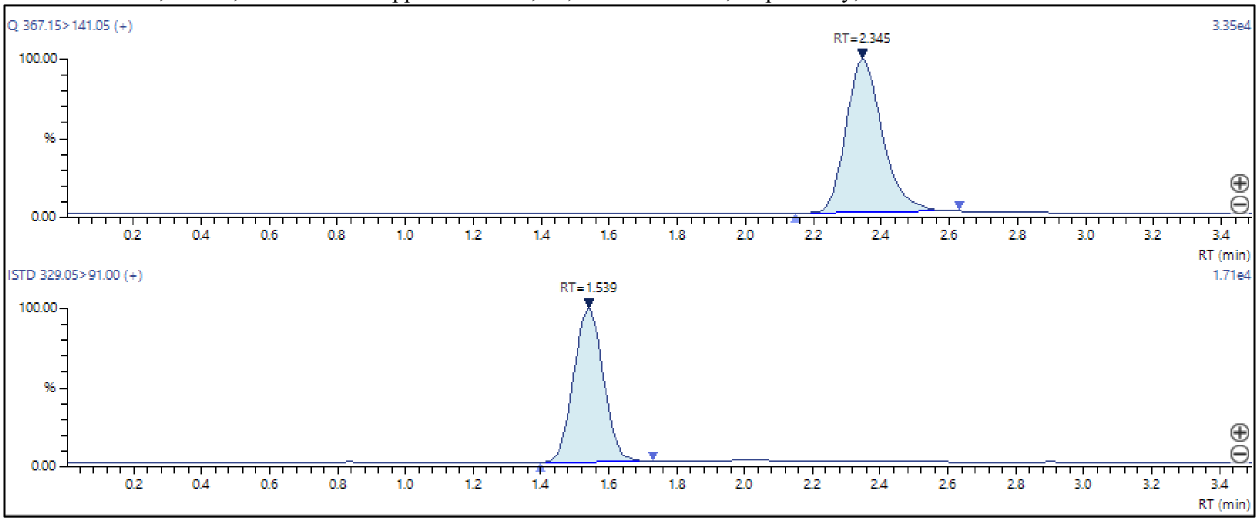

3.2 Chromatographic Analysis of LMD and Control Compounds in the MDR1-MDCK Permeability Assay

Figure S3.2.1: Representative chromatograms of LMD-022 and internal standard from the MDR1-MDCK permeability assay. The upper panel shows the LC-MS/MS peak for LMD-022 with a retention time (RT) of 2.049 minutes. The lower panel shows the internal standard (IS) labetalol with an RT of 1.864 minutes. Calculated recovery for LMD-022 was 61.9% in the A→B direction and 120% in the B→A direction. Column: Atlantis dC18, 3.1 µm, 4.6 × 50 mm; eluent: gradient of 0.1% formic acid in water and acetonitrile (starting at 95:5); flow: 0.80 mL/min.

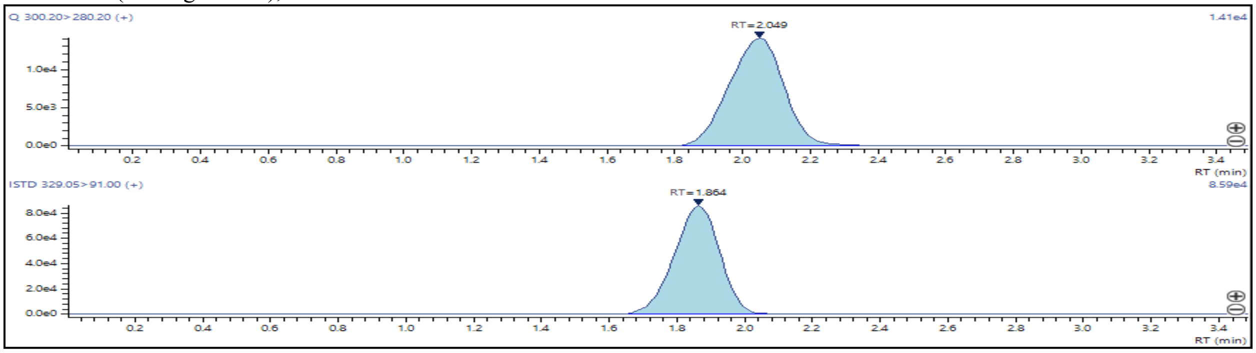

Figure S3.2.2. Representative chromatograms and recovery data for Digoxin in the MDR1-MDCK permeability assay. The upper panel shows the LC-MS/MS peak for Digoxin with a RT of 1.871 minutes. The lower panel shows the IS labetalol with an RT of 1.672 minutes. Calculated recovery for Digoxin was 79.7% in the A→B direction and 108% in the B→A direction. Column: Atlantis dC18, 3.1 µm, 4.6 × 50 mm; eluent: gradient of 0.1% formic acid in water and acetonitrile (starting at 90:10); flow: 0.80 mL/min.

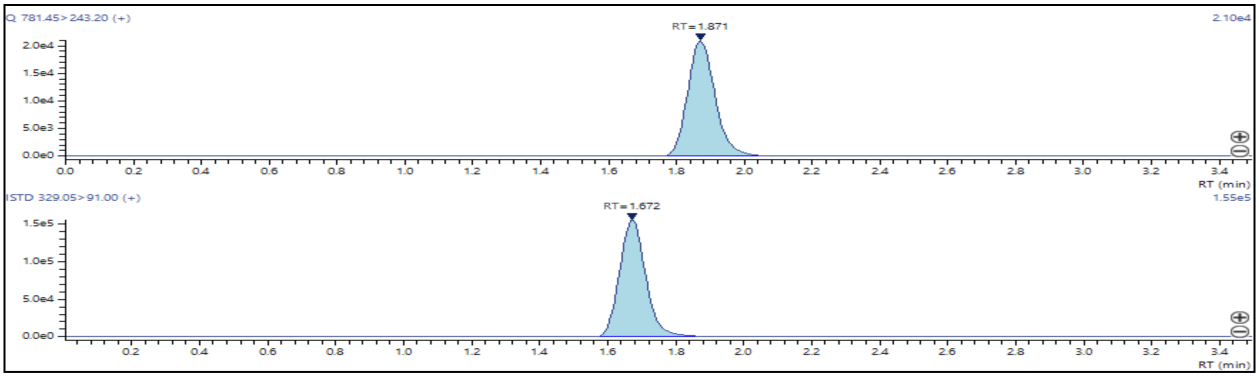

Figure S3.2.3. Representative chromatograms and recovery data for Propranolol in the MDR1-MDCK permeability assay. The upper panel shows the LC-MS/MS peak for Propranolol with a RT of 1.713 minutes. The lower panel shows the IS loperamide with an RT of 1.890 minutes. Calculated recovery for Propranolol was 95.0% in the A→B direction and 116% in the B→A direction. Column: Atlantis dC18, 3.1 µm, 4.6 × 50 mm; eluent: gradient of 0.1% formic acid in water and acetonitrile (starting at 90:10); flow: 0.80 mL/min

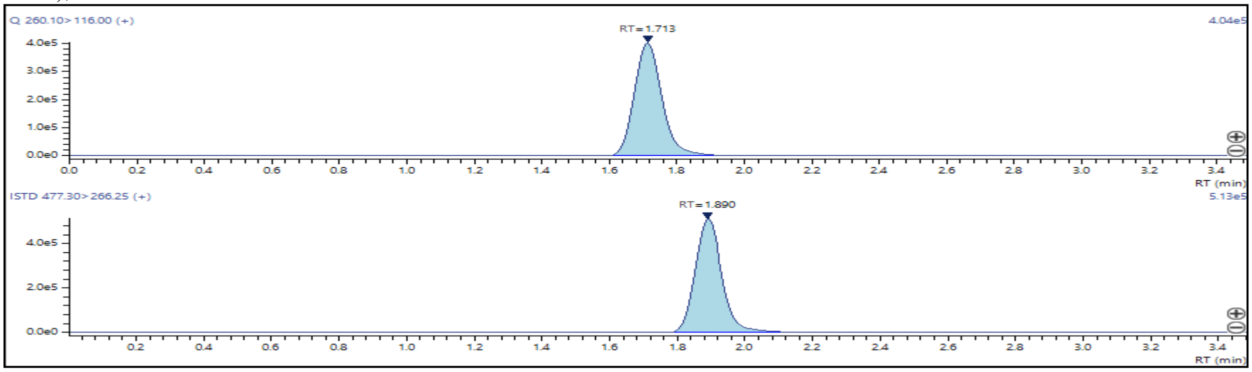

4 Characterization by ^{1}H NMR spectroscopy

^{1}H NMR spectrum (400 MHz) of **LMD-001**. Chemical shifts (δ) are reported in parts per million (ppm). The molecular structure is shown in the inset.

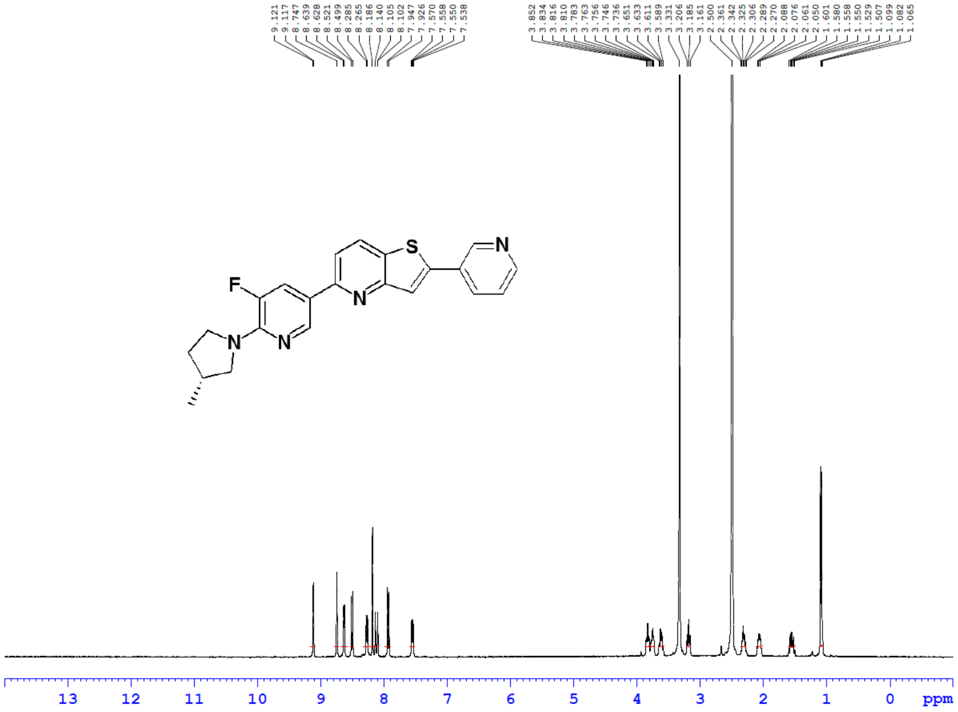

^{1}H NMR spectrum (400 MHz, $CDCl_3$) of **LMD-002**. Chemical shifts (δ) are reported in parts per million (ppm). The molecular structure is shown in the inset.

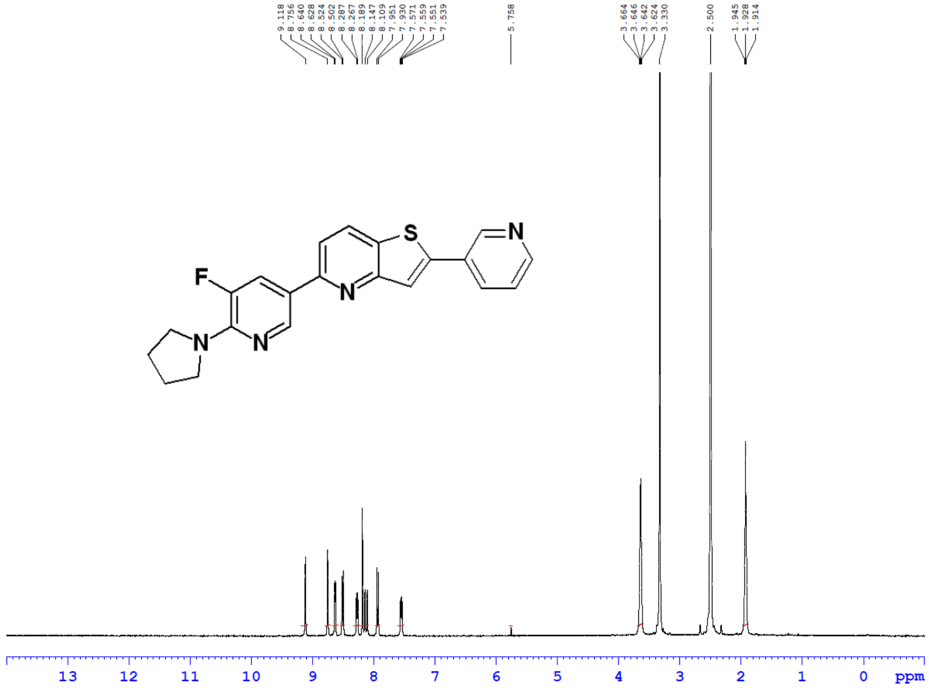

^{1}H NMR spectrum (400 MHz) of **LMD-005**. Chemical shifts (δ) are reported in parts per million (ppm). The molecular structure is shown in the inset.

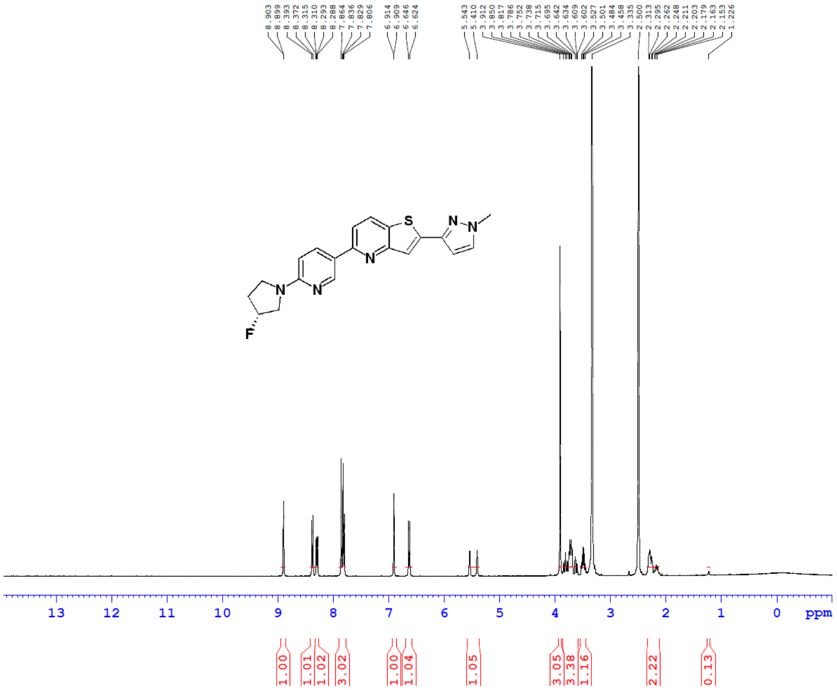

^{1}H NMR spectrum (400 MHz) of **LMD-006**. Chemical shifts (δ) are reported in parts per million (ppm). The molecular structure is shown in the inset.

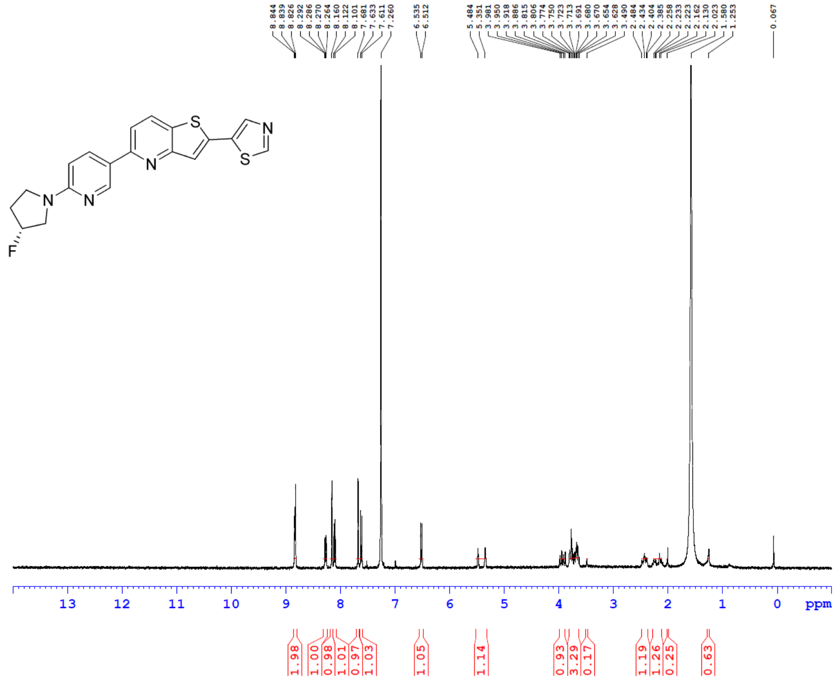

^{1}H NMR spectrum (400 MHz) of **LMD-009**. Chemical shifts (δ) are reported in parts per million (ppm). The molecular structure is shown in the inset.

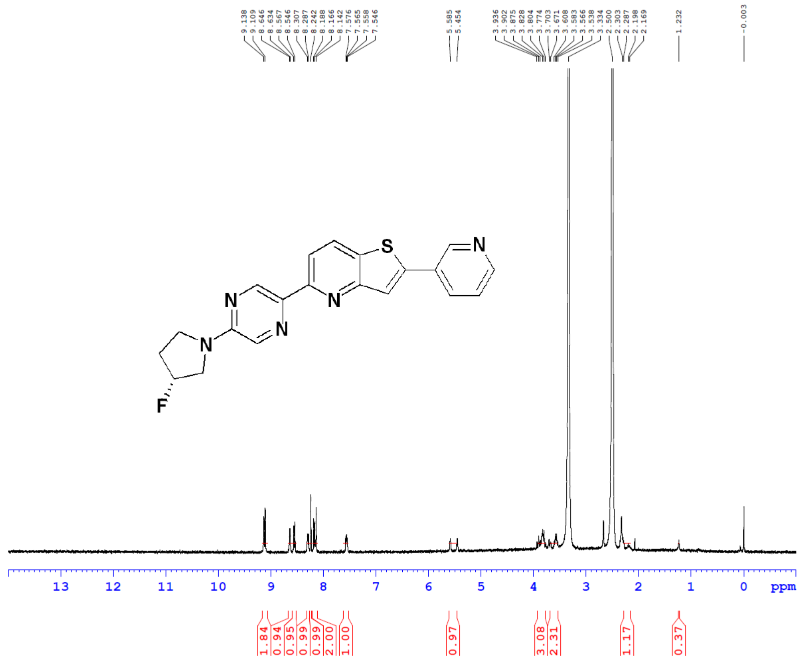

^{1}H NMR spectrum (400 MHz) of **LMD-011**. Chemical shifts (δ) are reported in parts per million (ppm). The molecular structure is shown in the inset.

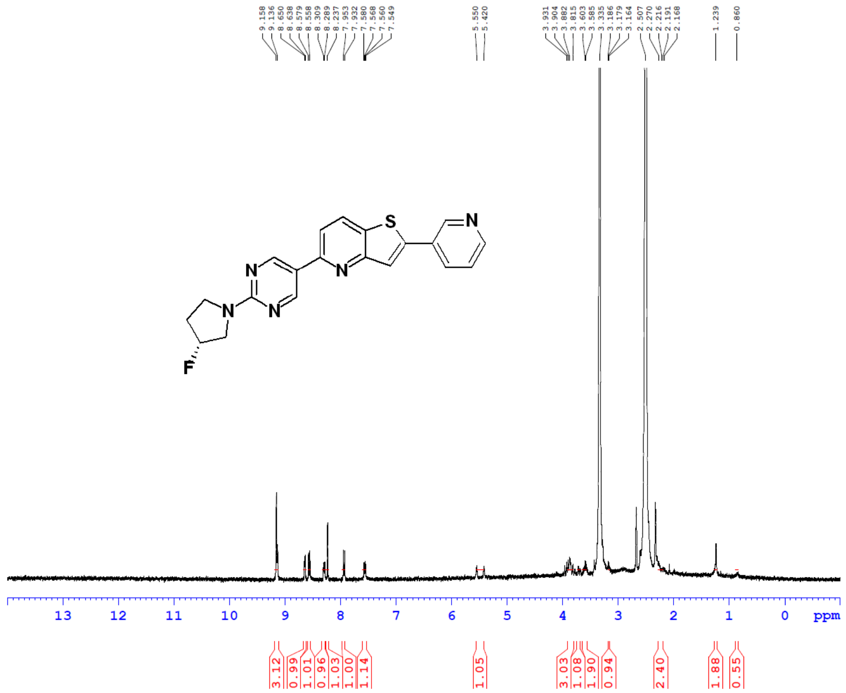

^{1}H NMR spectrum (400 MHz) of **LMD-013**. Chemical shifts (δ) are reported in parts per million (ppm). The molecular structure is shown in the inset.

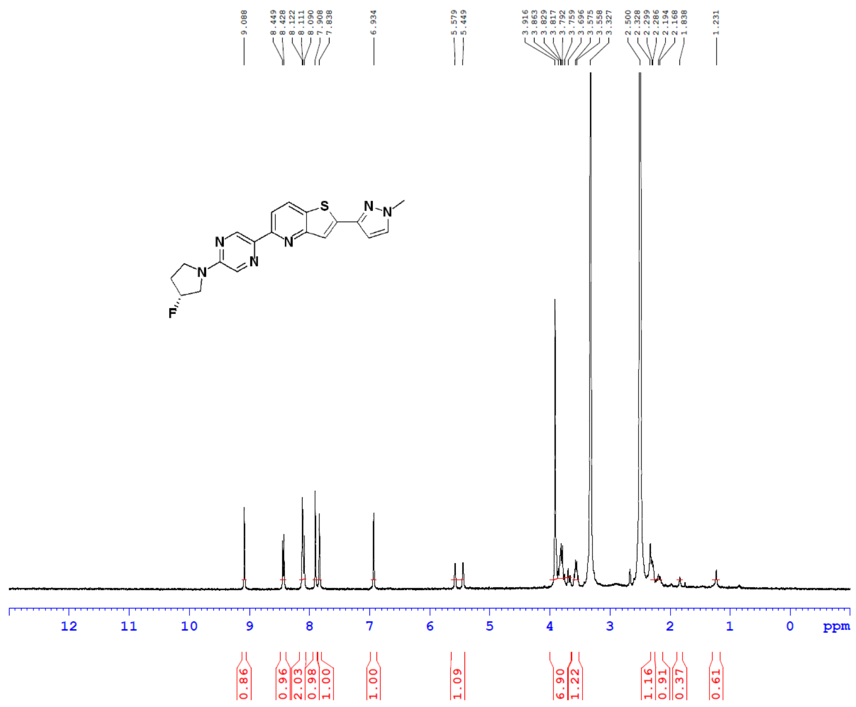

^{1}H NMR spectrum (400 MHz) of **LMD-014**. Chemical shifts (δ) are reported in parts per million (ppm). The molecular structure is shown in the inset.

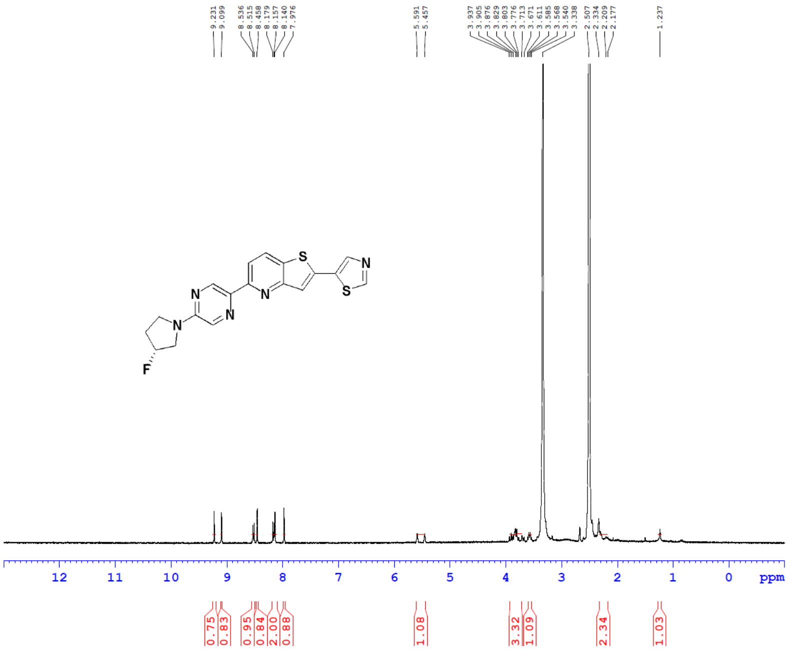

^{1}H NMR spectrum (400 MHz) of **LMD-015**. Chemical shifts (δ) are reported in parts per million (ppm). The molecular structure is shown in the inset.

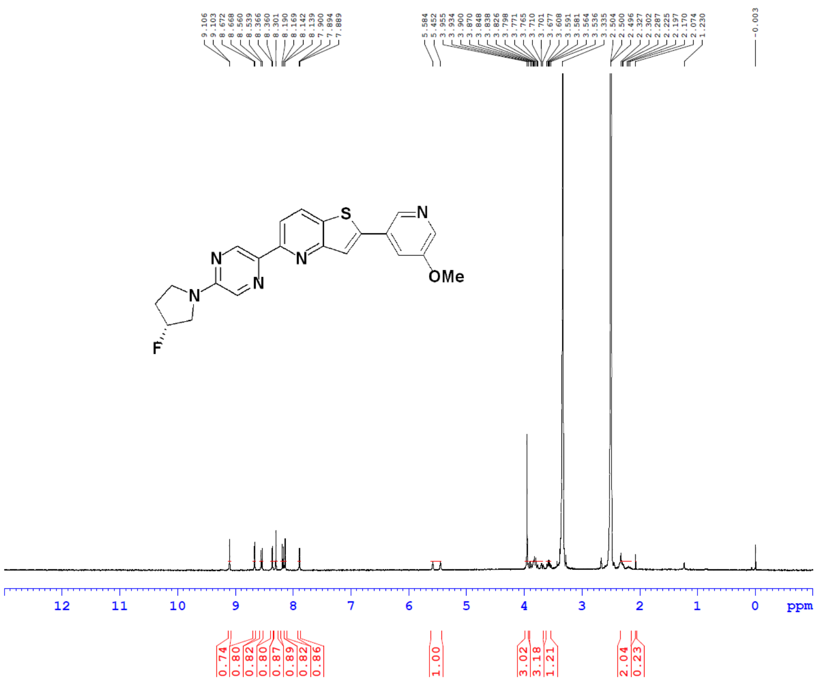

^{1}H NMR spectrum (400 MHz) of **LMD-016**. Chemical shifts (δ) are reported in parts per million (ppm). The molecular structure is shown in the inset.

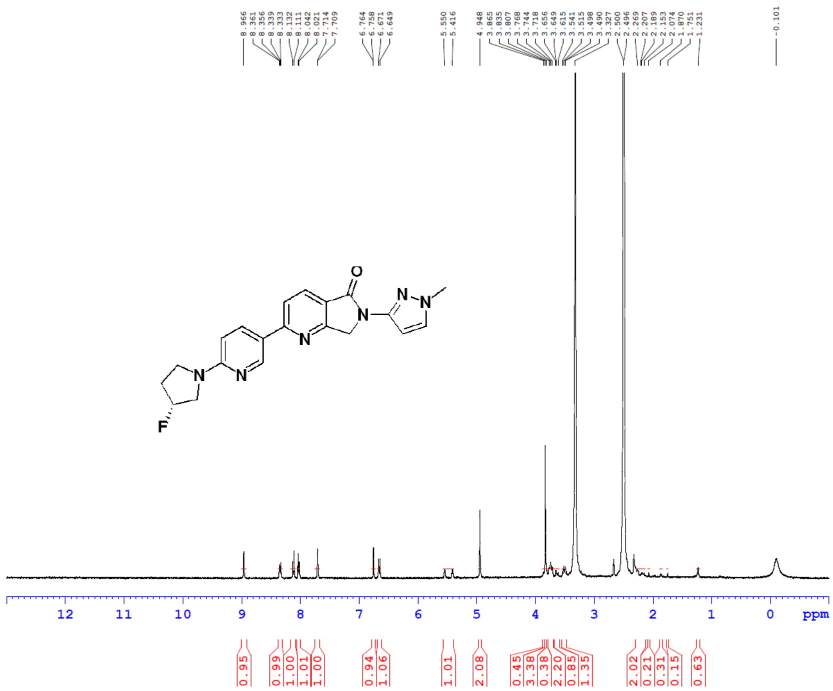

^{1}H NMR spectrum (400 MHz) of **LMD-017**. Chemical shifts (δ) are reported in parts per million (ppm). The molecular structure is shown in the inset.

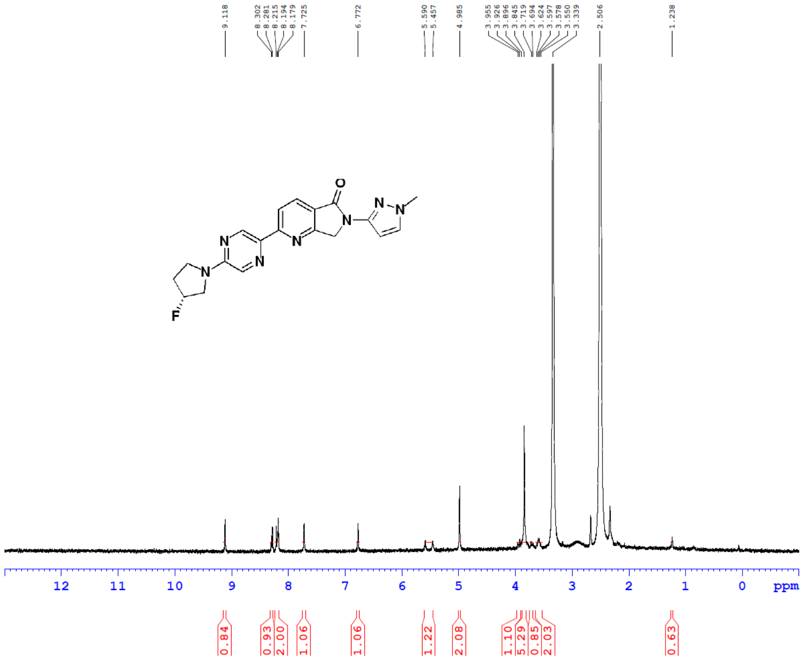

^{1}H NMR spectrum (400 MHz) of **LMD-019**. Chemical shifts (δ) are reported in parts per million (ppm). The molecular structure is shown in the inset.

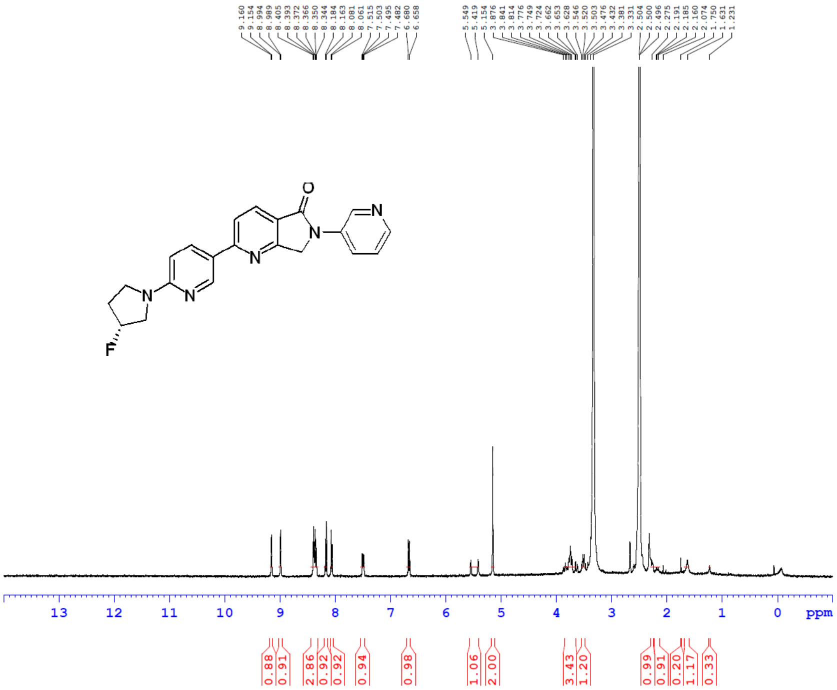

^{1}H NMR spectrum (400 MHz) of **LMD-022**. Chemical shifts (δ) are reported in parts per million (ppm). The molecular structure is shown in the inset.

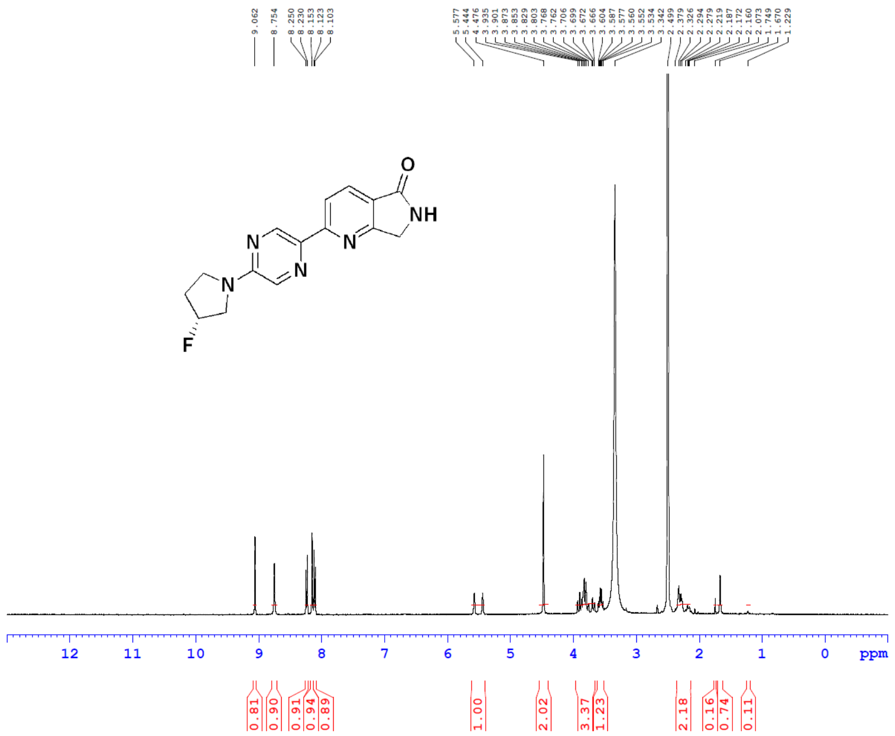

^{1}H NMR spectrum (400 MHz) of **LMD-023**. Chemical shifts (δ) are reported in parts per million (ppm). The molecular structure is shown in the inset.

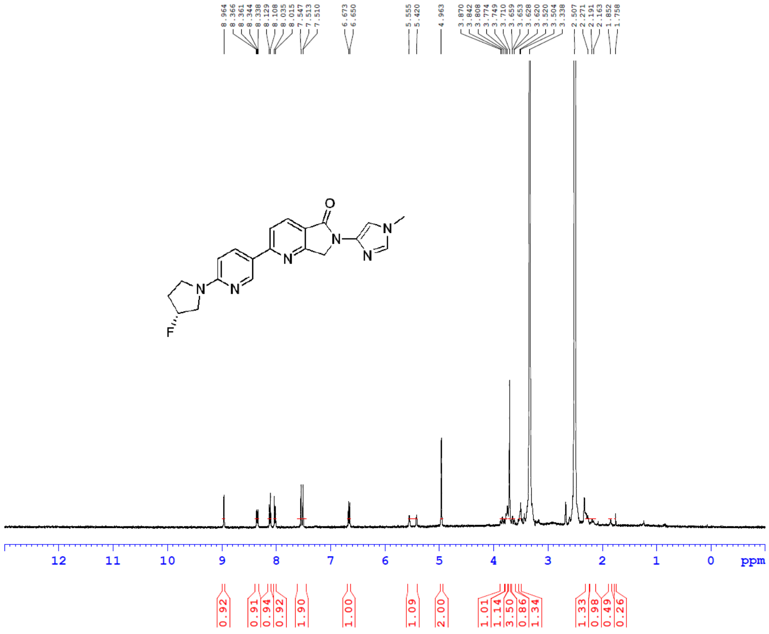

^{1}H NMR spectrum (400 MHz) of **LMD-024**. Chemical shifts (δ) are reported in parts per million (ppm). The molecular structure is shown in the inset.

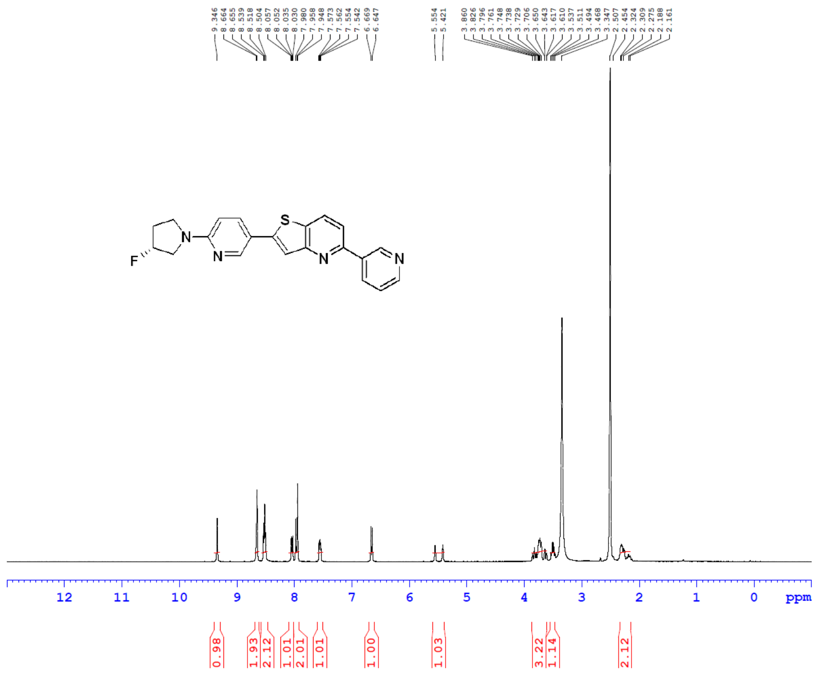

^{1}H NMR spectrum (400 MHz) of **LMD-026**. Chemical shifts (δ) are reported in parts per million (ppm). The molecular structure is shown in the inset.

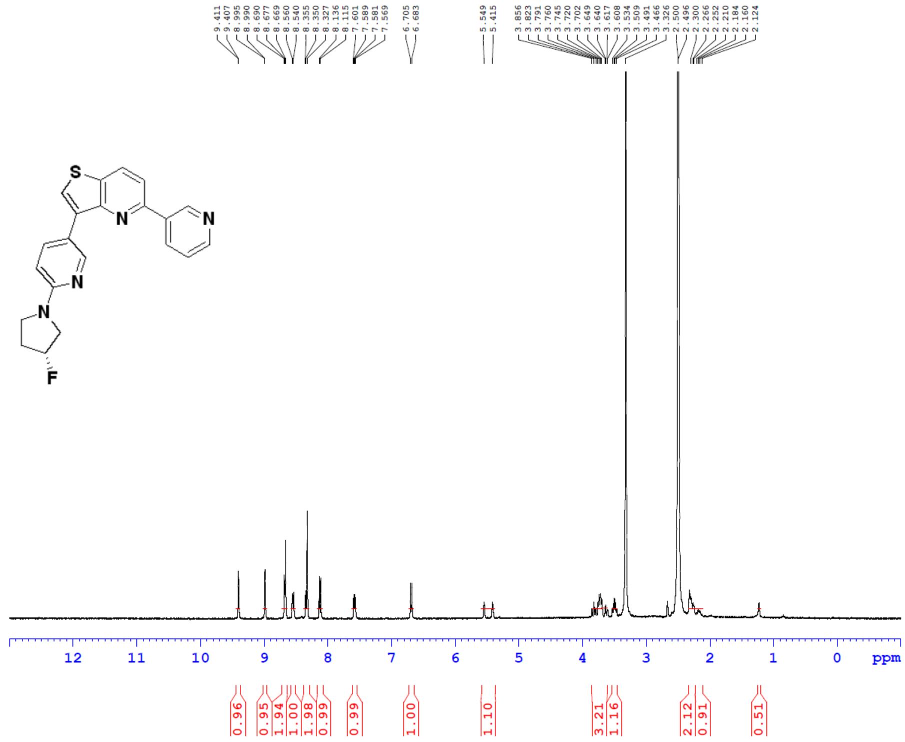

^{1}H NMR spectrum (400 MHz) of **LMD-028**. Chemical shifts (δ) are reported in parts per million (ppm). The molecular structure is shown in the inset.

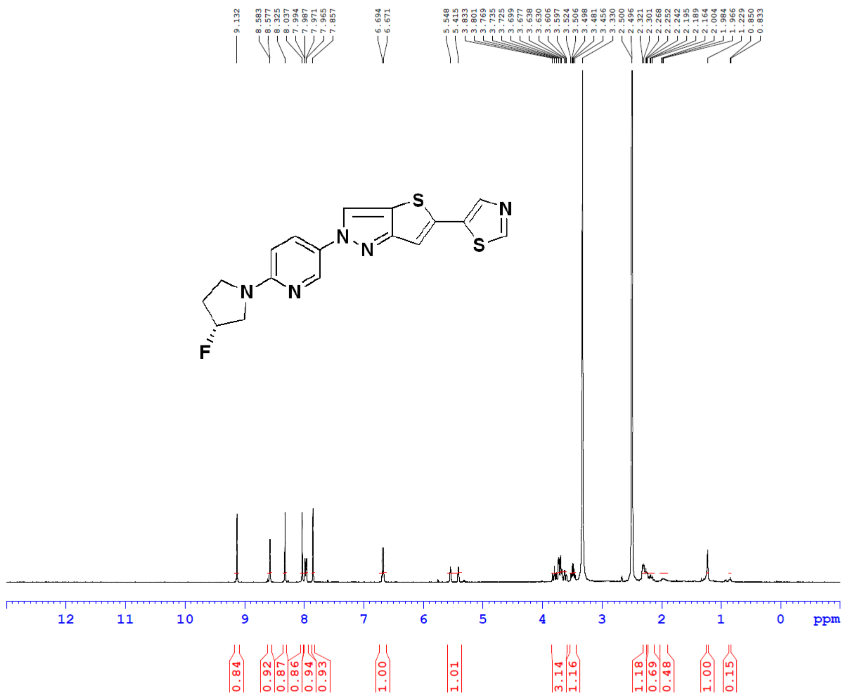

^{1}H NMR spectrum (400 MHz) of **LMD-029**. Chemical shifts (δ) are reported in parts per million (ppm). The molecular structure is shown in the inset.

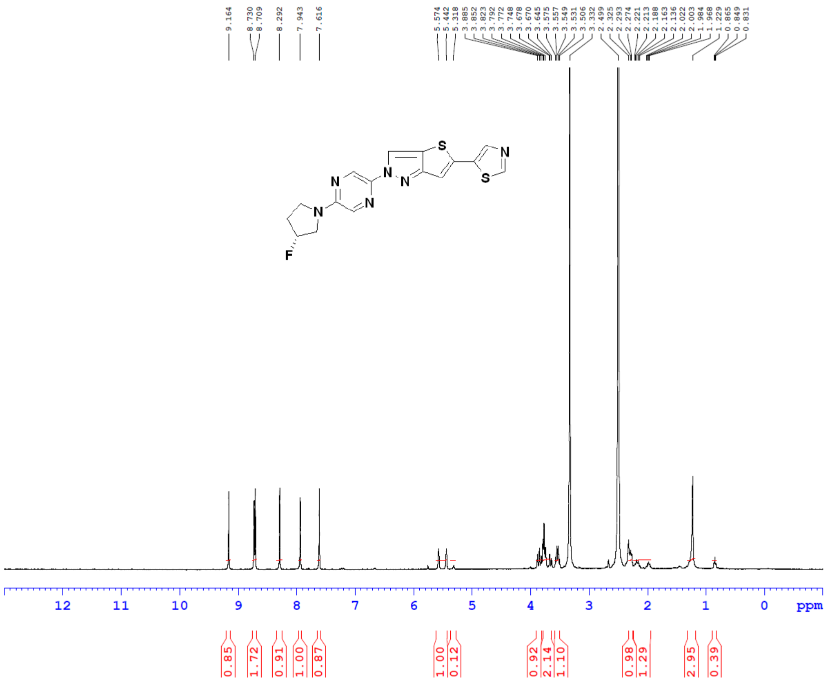

^{1}H NMR spectrum (400 MHz) of **LMD-031**. Chemical shifts (δ) are reported in parts per million (ppm). The molecular structure is shown in the inset.

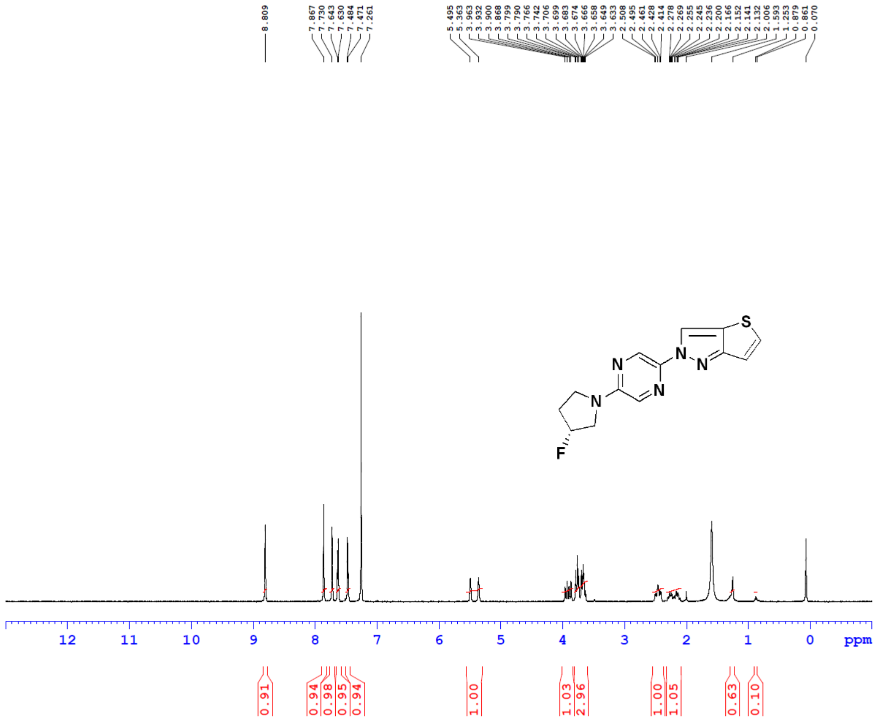

^{1}H NMR spectrum (400 MHz) of **LMD-032**. Chemical shifts (δ) are reported in parts per million (ppm). The molecular structure is shown in the inset.

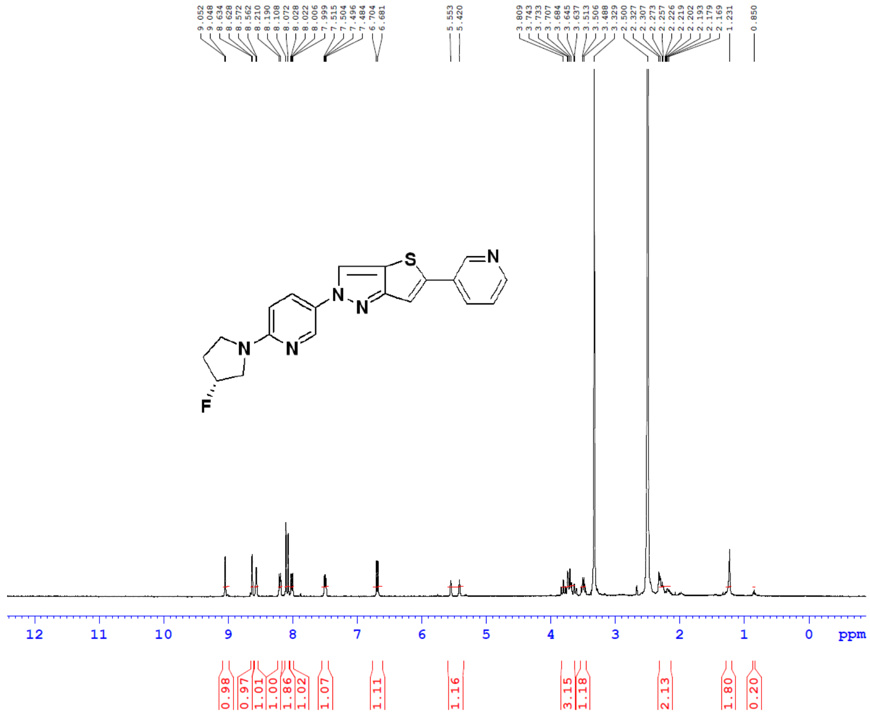

^{1}H NMR spectrum (400 MHz) of **LMD-033**. Chemical shifts (δ) are reported in parts per million (ppm). The molecular structure is shown in the inset.

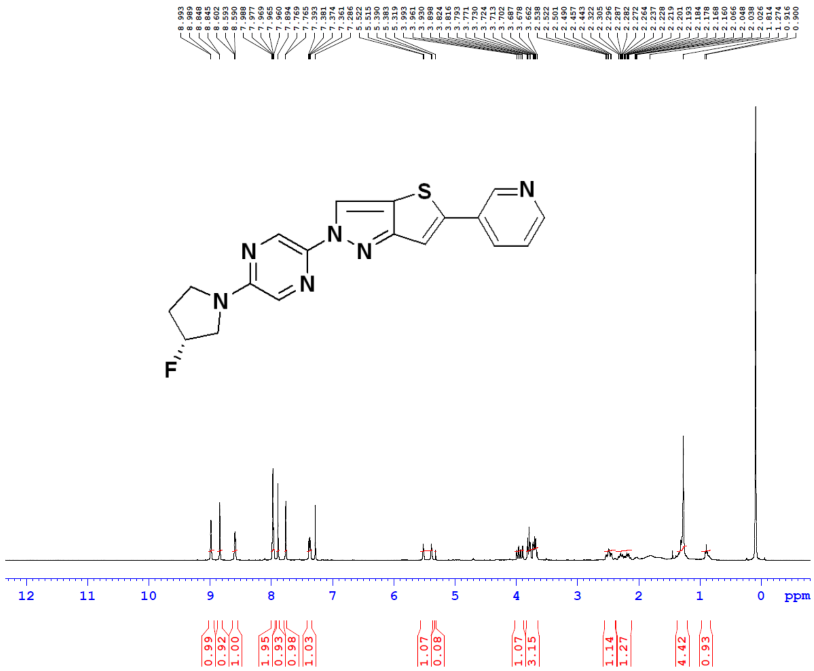

^{1}H NMR spectrum (400 MHz) of **LMD-034**. Chemical shifts (δ) are reported in parts per million (ppm). The molecular structure is shown in the inset.

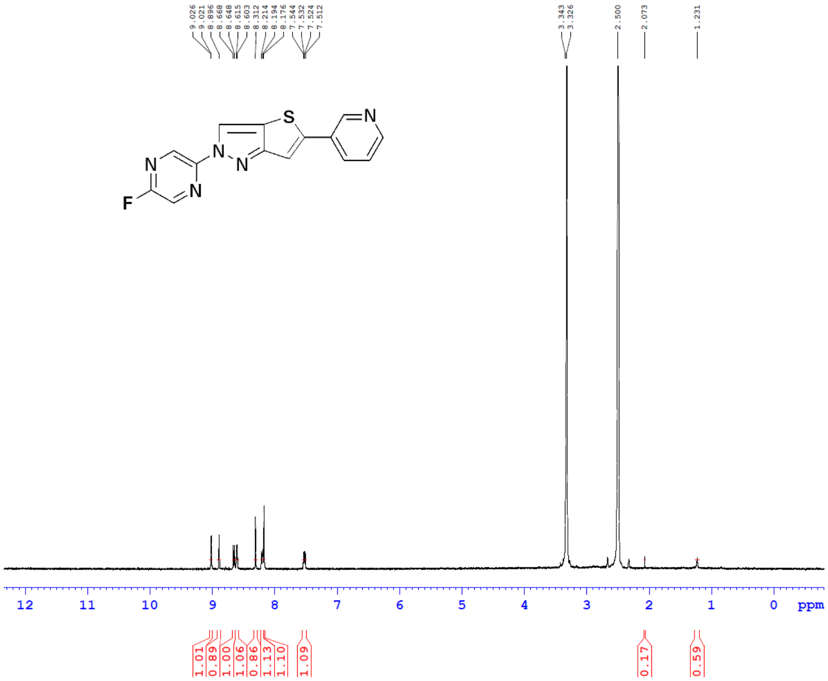

^{1}H NMR spectrum (400 MHz) of **LMD-036**. Chemical shifts (δ) are reported in parts per million (ppm). The molecular structure is shown in the inset.

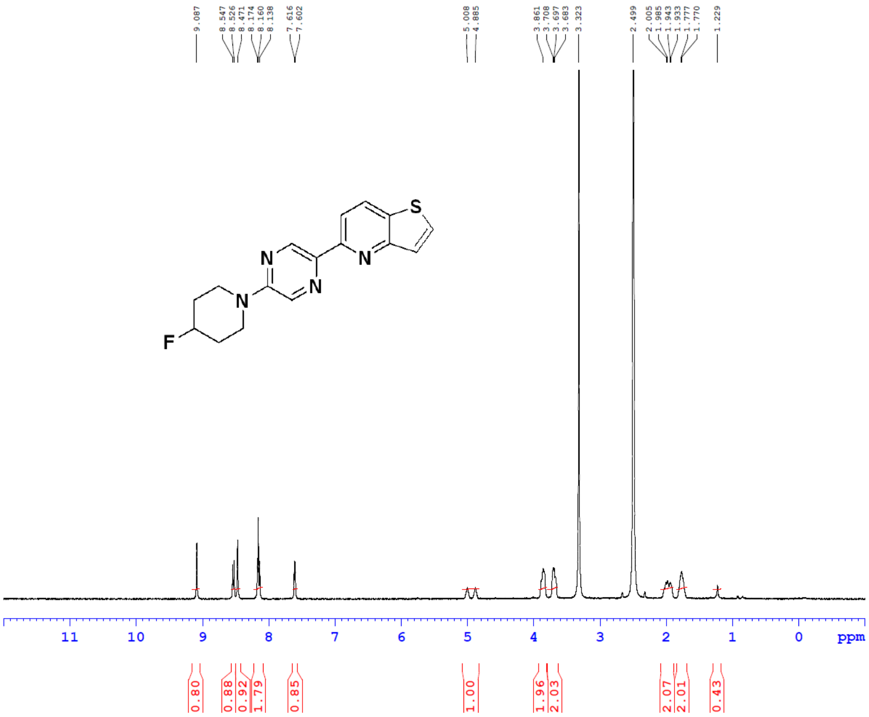

^{1}H NMR spectrum (400 MHz) of **LMD-039**. Chemical shifts (δ) are reported in parts per million (ppm). The molecular structure is shown in the inset.

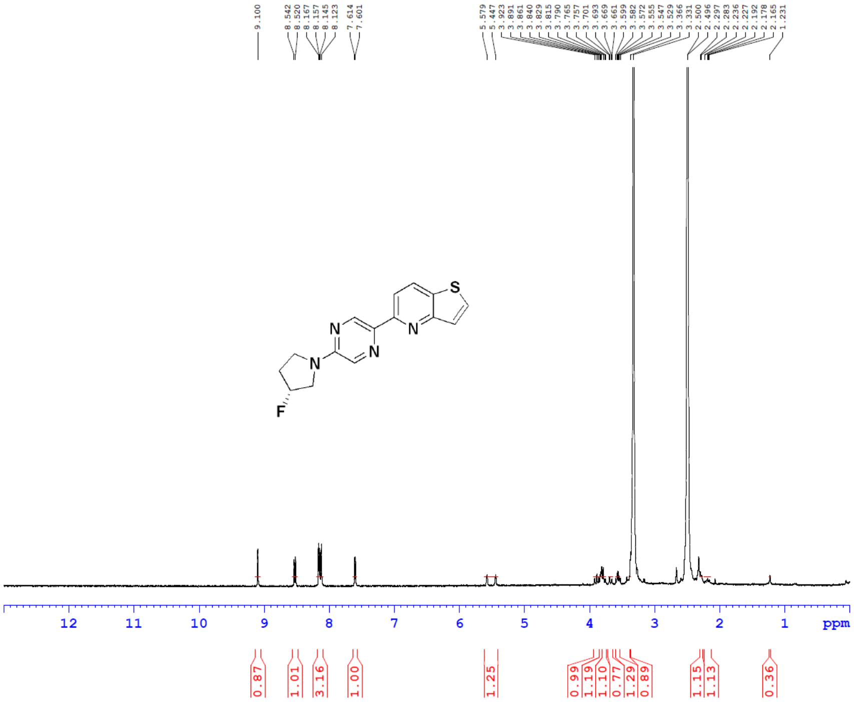

^{1}H NMR spectrum (400 MHz) of **LMD-040**. Chemical shifts (δ) are reported in parts per million (ppm). The molecular structure is shown in the inset.

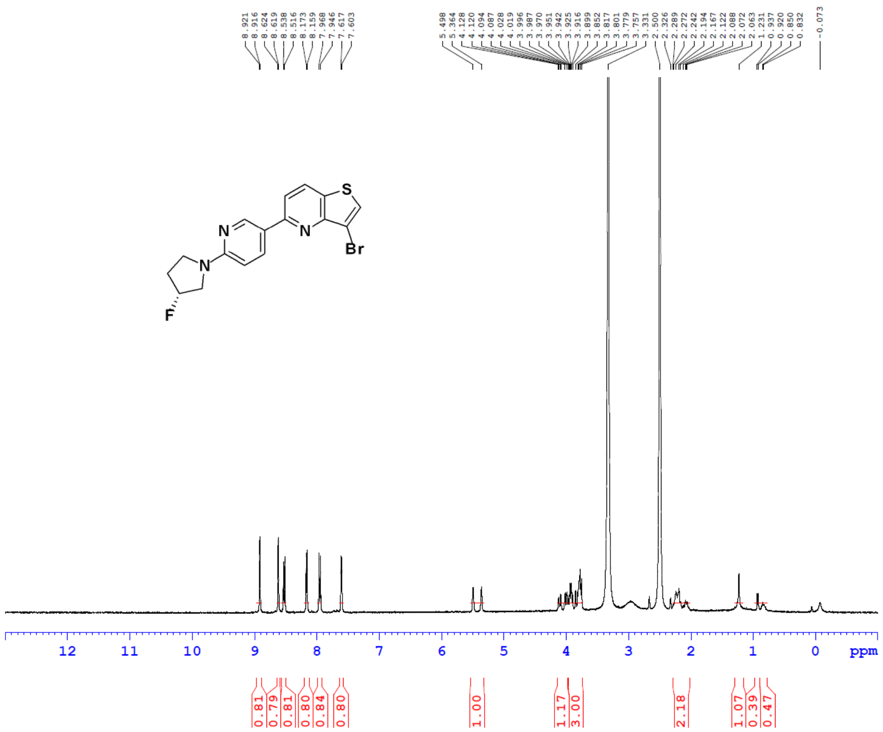

^{1}H NMR spectrum (400 MHz) of **LMD-041**. Chemical shifts (δ) are reported in parts per million (ppm). The molecular structure is shown in the inset.

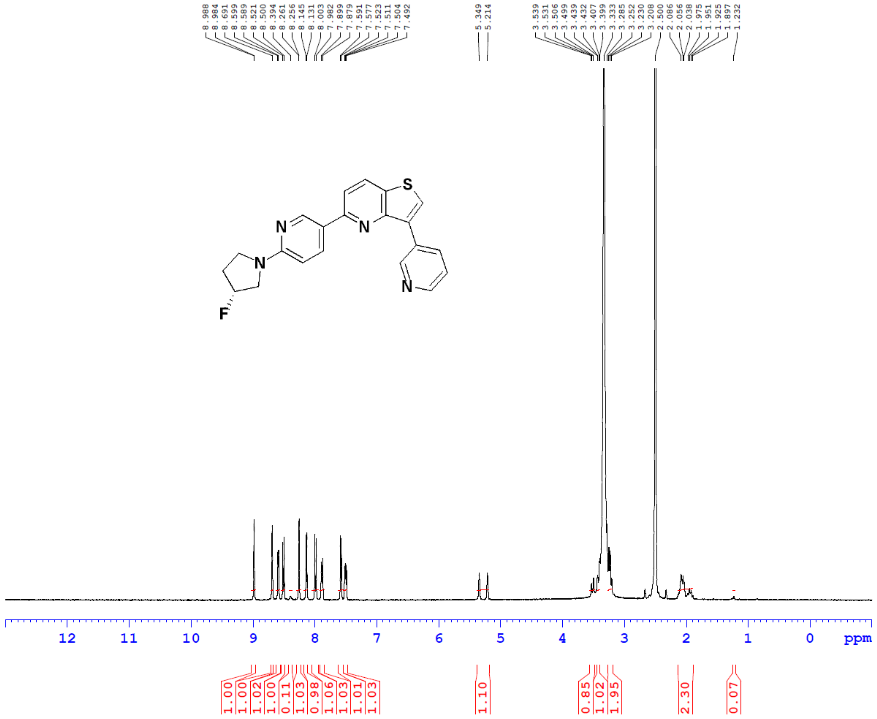

^{1}H NMR spectrum (400 MHz) of **LMD-044**. Chemical shifts (δ) are reported in parts per million (ppm). The molecular structure is shown in the inset.

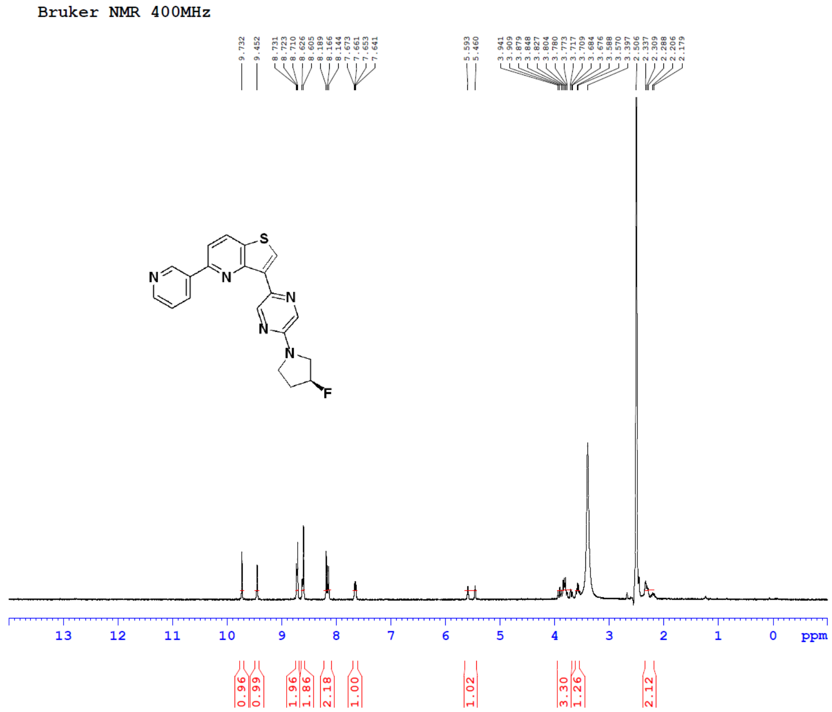

^{1}H NMR spectrum (400 MHz) of **LMD-046**. Chemical shifts (δ) are reported in parts per million (ppm). The molecular structure is shown in the inset.

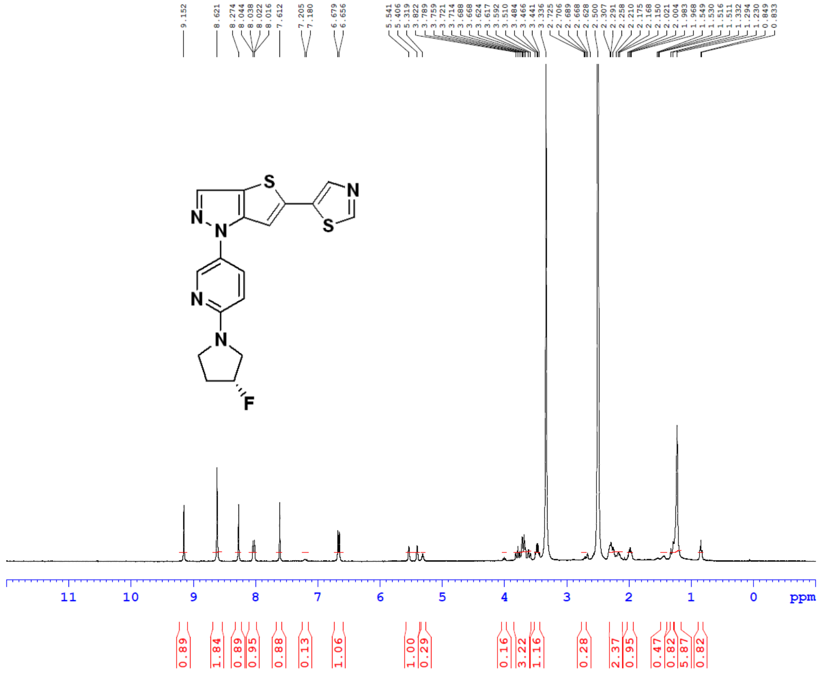

^{1}H NMR spectrum (400 MHz) of **LMD-049**. Chemical shifts (δ) are reported in parts per million (ppm). The molecular structure is shown in the inset.

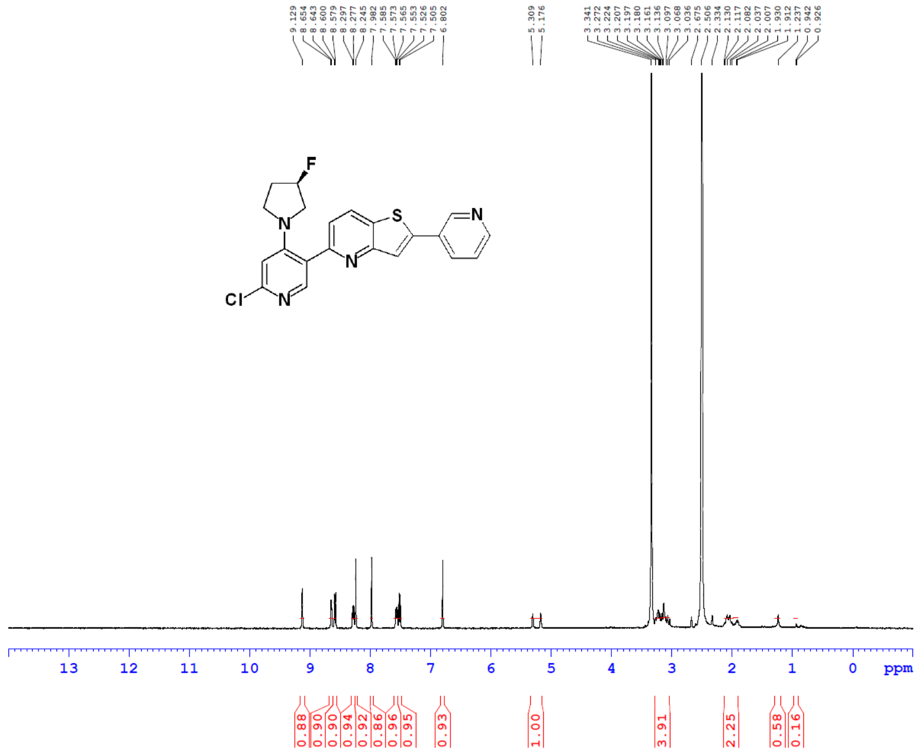

^{1}H NMR spectrum (400 MHz) of **LMD-051**. Chemical shifts (δ) are reported in parts per million (ppm). The molecular structure is shown in the inset.

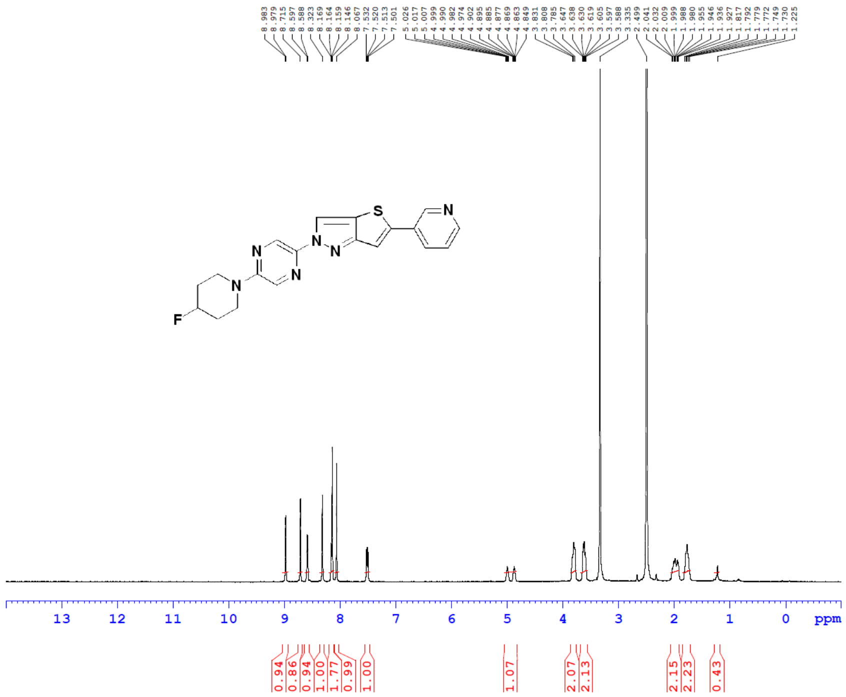

^{1}H NMR spectrum (400 MHz) of **LMD-052**. Chemical shifts (δ) are reported in parts per million (ppm). The molecular structure is shown in the inset.

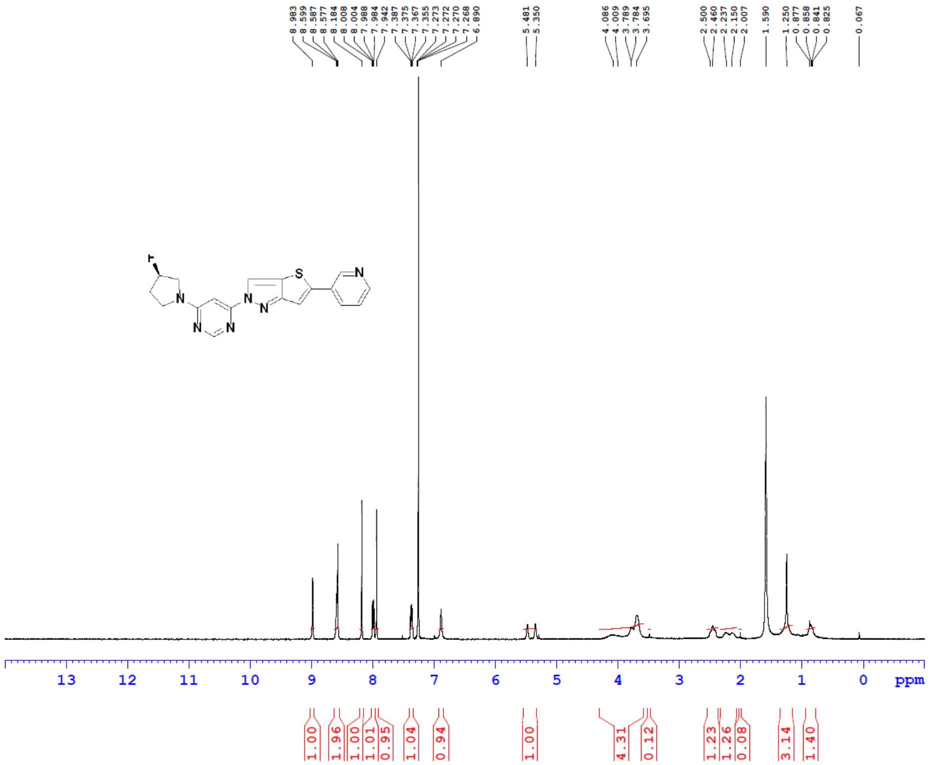

^{1}H NMR spectrum (400 MHz) of **LMD-054**. Chemical shifts (δ) are reported in parts per million (ppm). The molecular structure is shown in the inset.

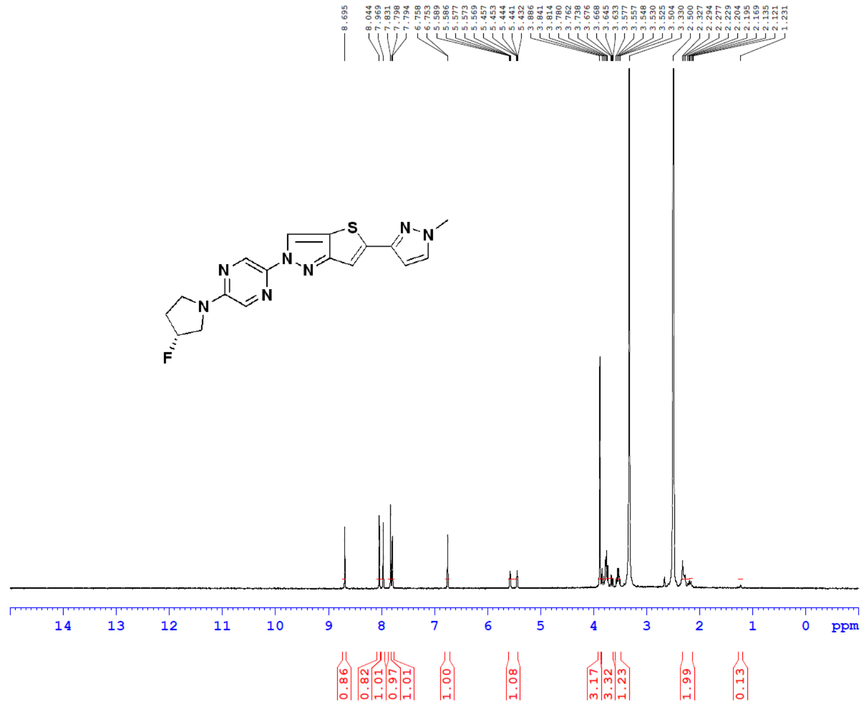

^{1}H NMR spectrum (400 MHz) of **LMD-060**. Chemical shifts (δ) are reported in parts per million (ppm). The molecular structure is shown in the inset.

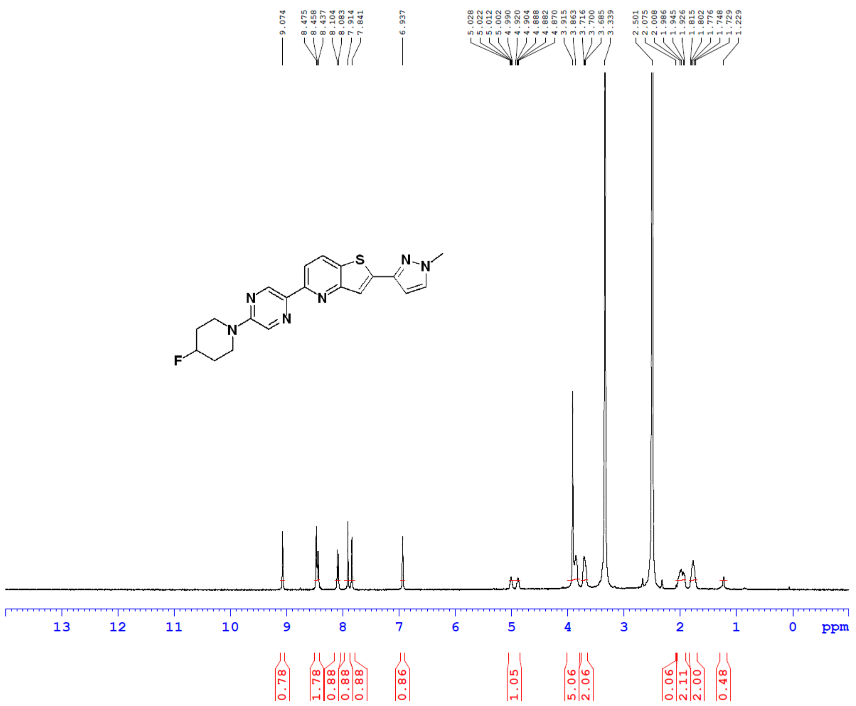

^{1}H NMR spectrum (400 MHz) of **LMD-061**. Chemical shifts (δ) are reported in parts per million (ppm). The molecular structure is shown in the inset.

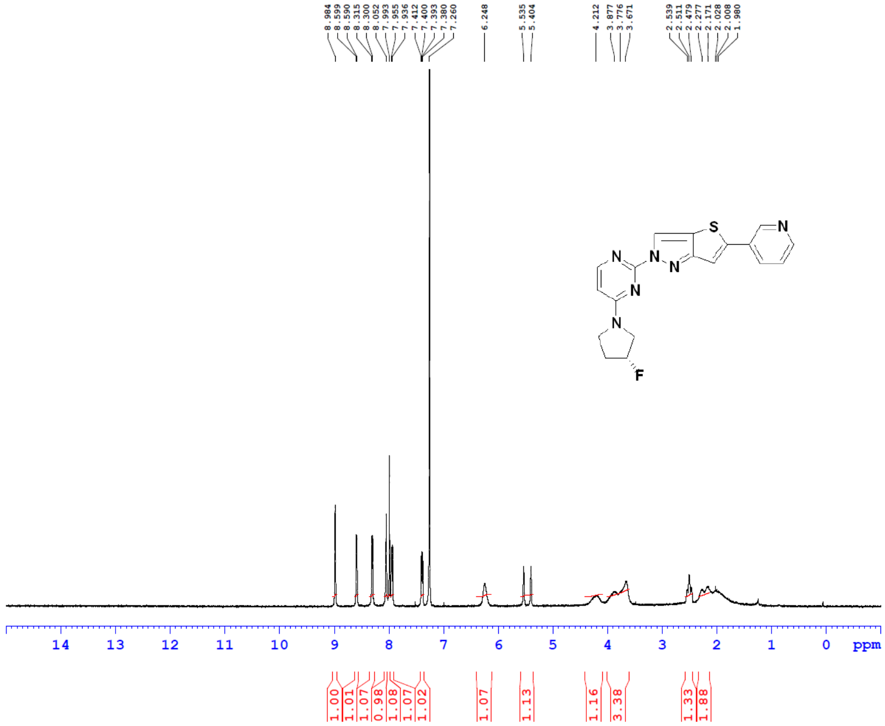

^{1}H NMR spectrum (400 MHz) of **LMD-062**. Chemical shifts (δ) are reported in parts per million (ppm). The molecular structure is shown in the inset.

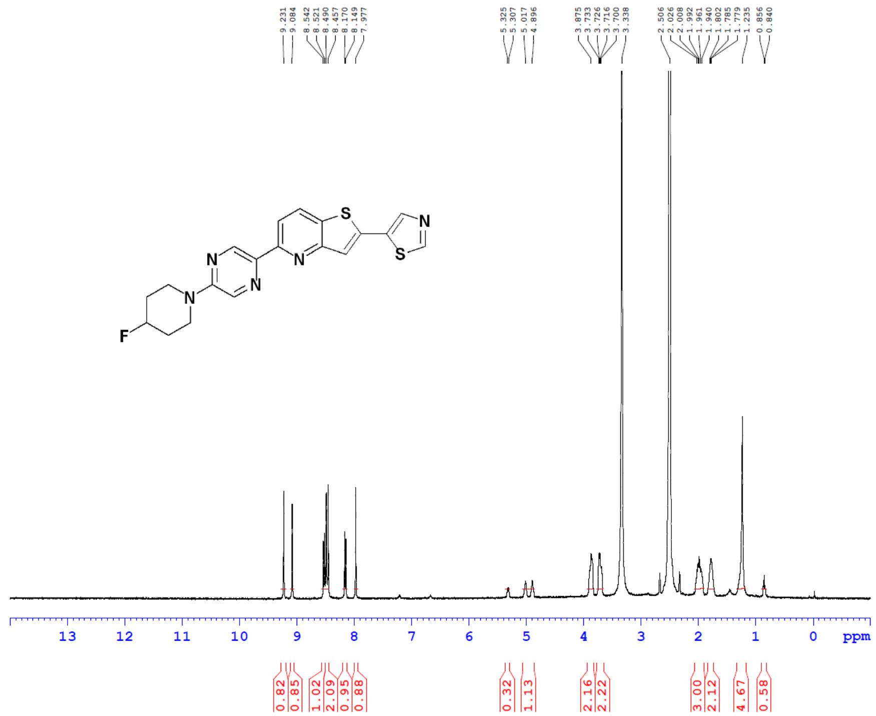

^{1}H NMR spectrum (400 MHz) of **LMD-063**. Chemical shifts (δ) are reported in parts per million (ppm). The molecular structure is shown in the inset.

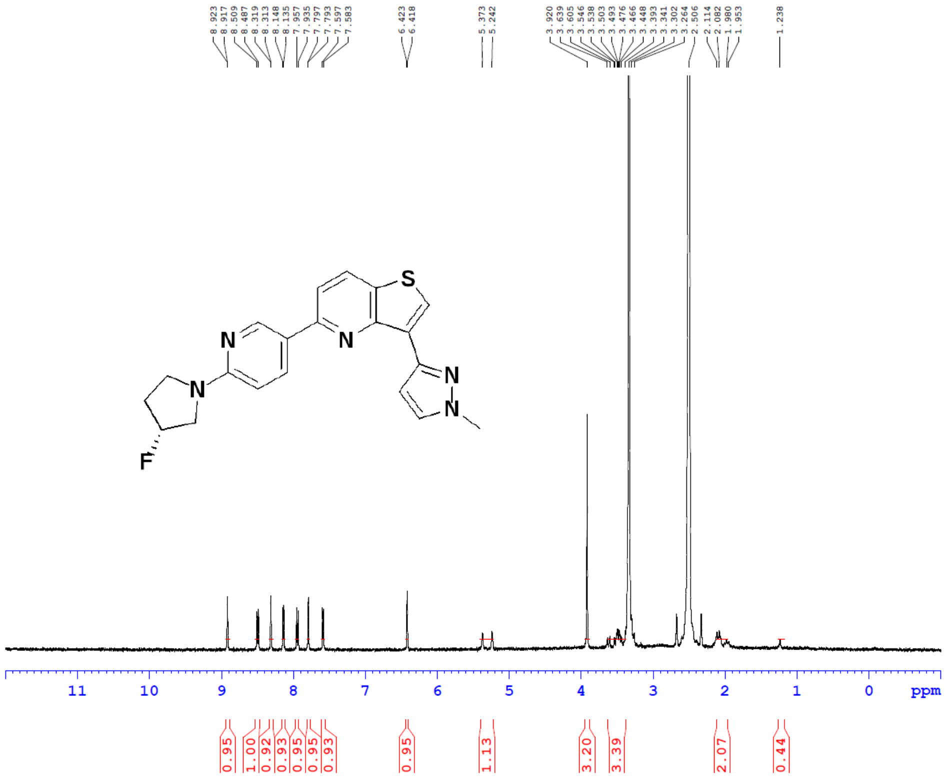

^{1}H NMR spectrum (400 MHz) of **LMD-064**. Chemical shifts (δ) are reported in parts per million (ppm). The molecular structure is shown in the inset.

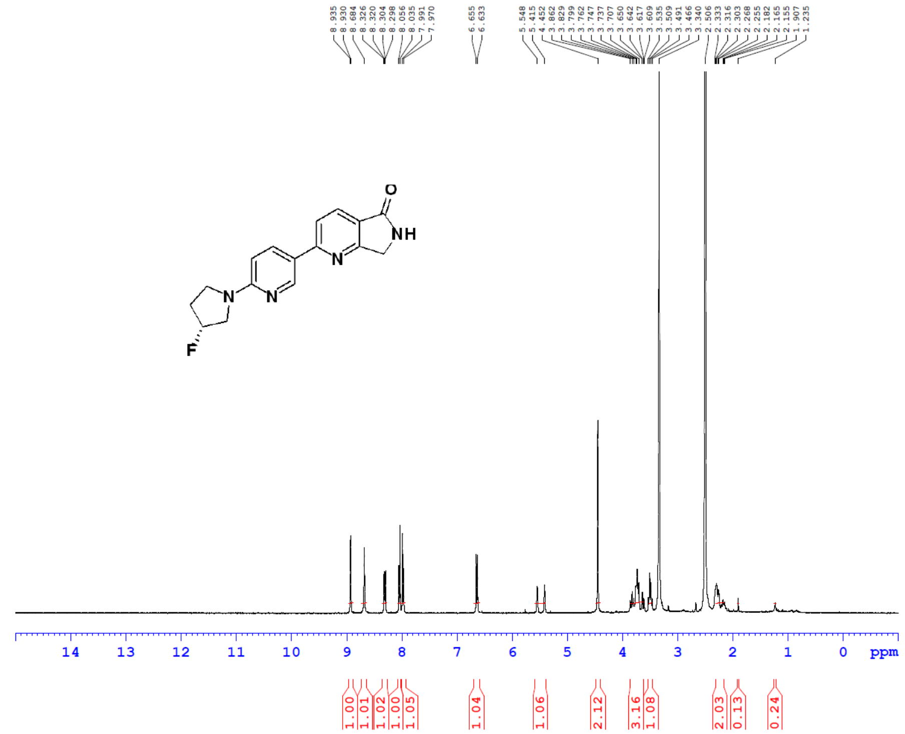

^{1}H NMR spectrum (400 MHz) of **LMD-066**. Chemical shifts (δ) are reported in parts per million (ppm). The molecular structure is shown in the inset.

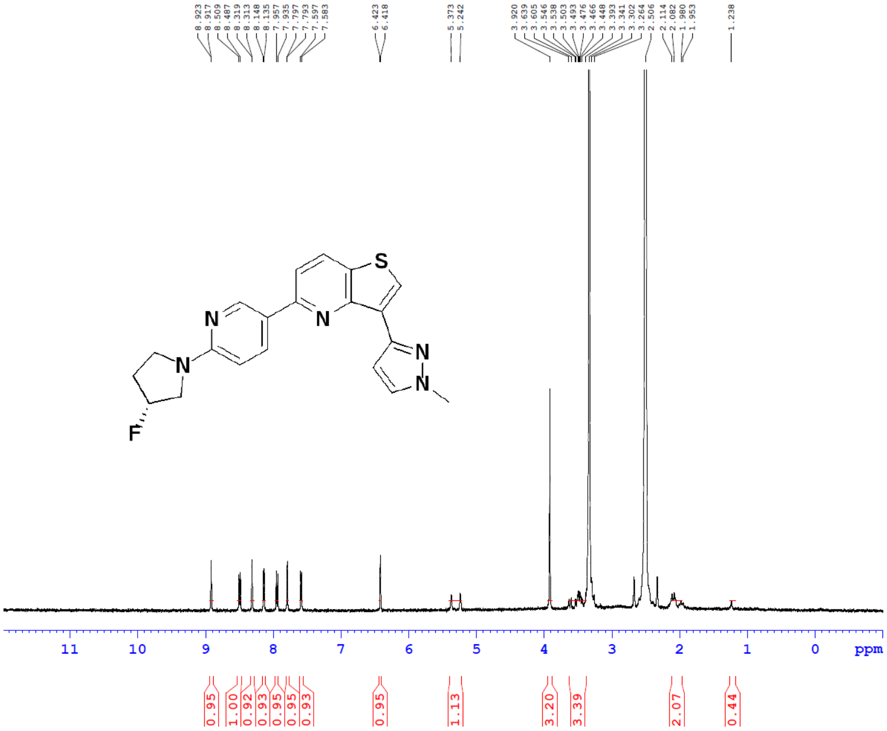

^{1}H NMR spectrum (400 MHz) of **LMD-067**. Chemical shifts (δ) are reported in parts per million (ppm). The molecular structure is shown in the inset.

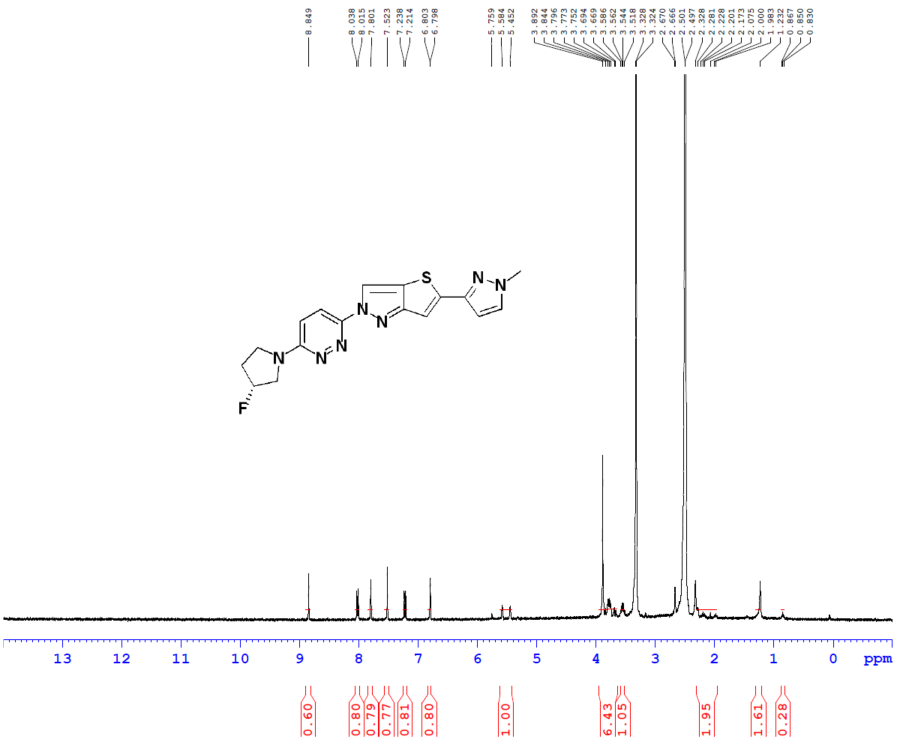

^{1}H NMR spectrum (400 MHz) of **LMD-068**. Chemical shifts (δ) are reported in parts per million (ppm). The molecular structure is shown in the inset.

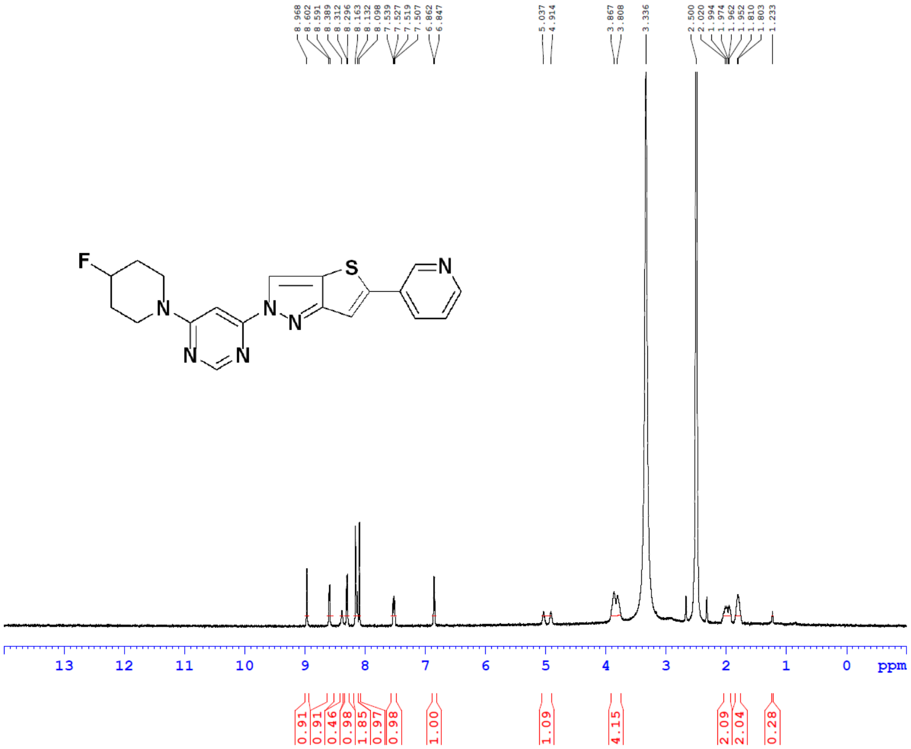

^{1}H NMR spectrum (400 MHz) of **LMD-069**. Chemical shifts (δ) are reported in parts per million (ppm). The molecular structure is shown in the inset.

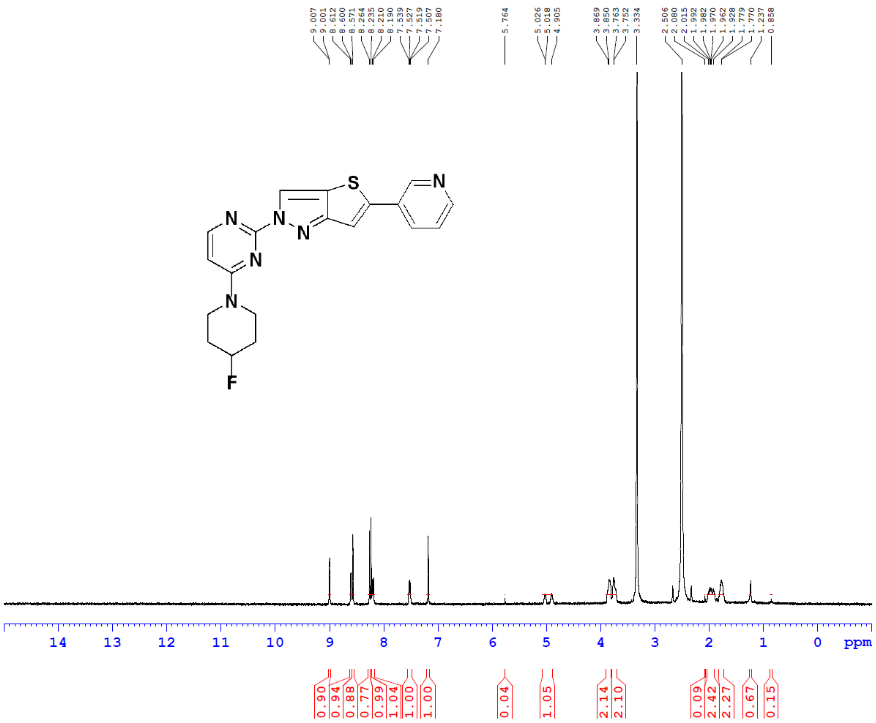

^{1}H NMR spectrum (400 MHz) of **LMD-070**. Chemical shifts (δ) are reported in parts per million (ppm). The molecular structure is shown in the inset.

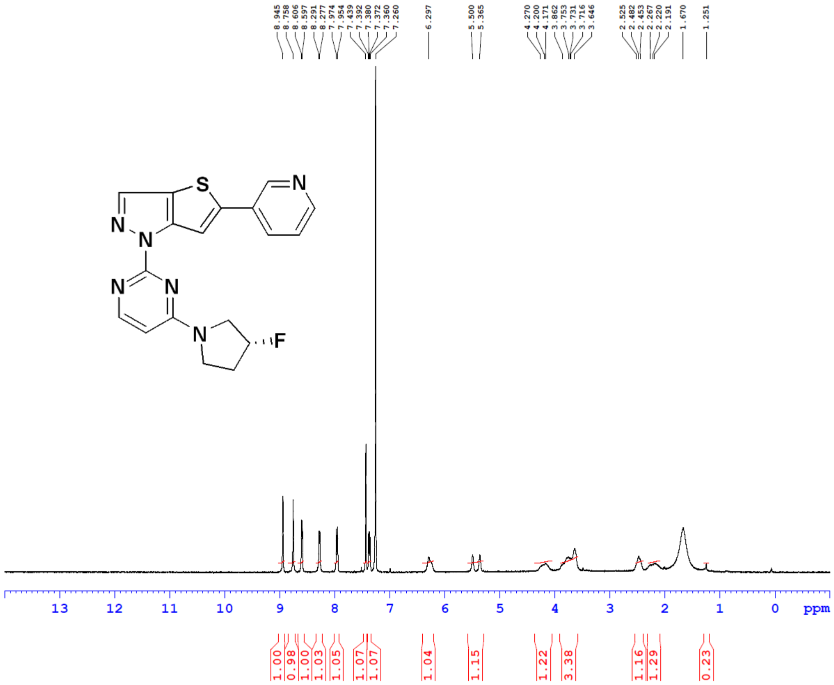

^{1}H NMR spectrum (400 MHz) of **LMD-072**. Chemical shifts (δ) are reported in parts per million (ppm). The molecular structure is shown in the inset.

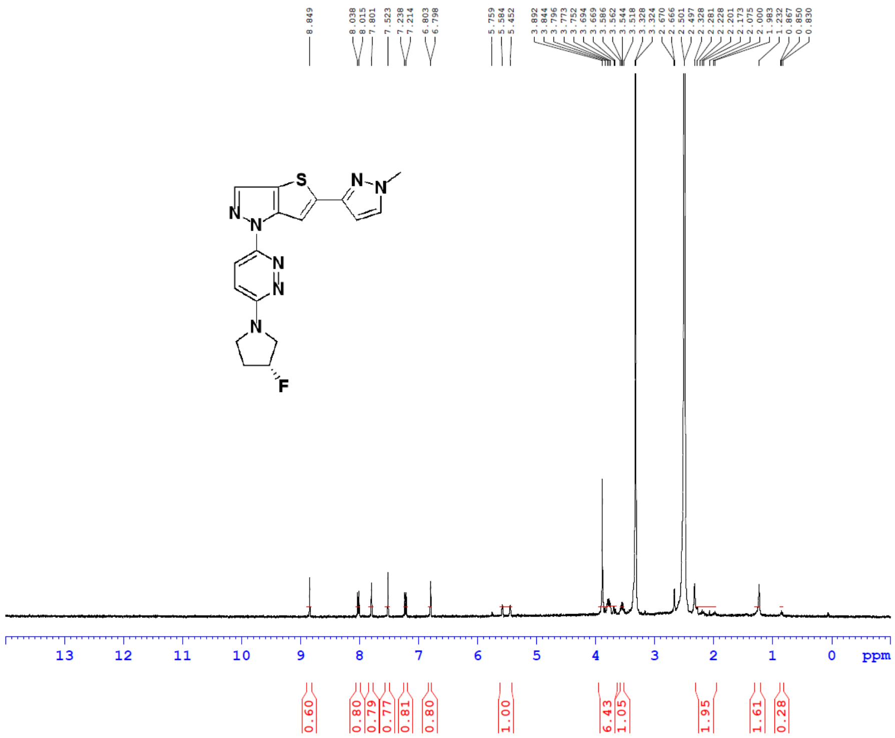

^{1}H NMR spectrum (400 MHz) of **LMD-073**. Chemical shifts (δ) are reported in parts per million (ppm). The molecular structure is shown in the inset.

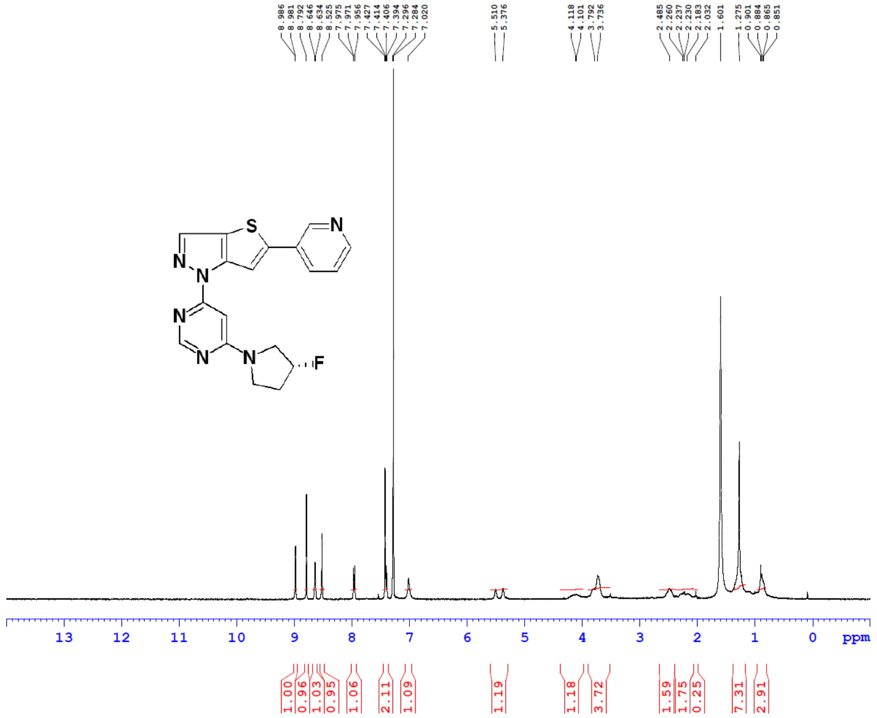

^{1}H NMR spectrum (400 MHz) of **LMD-074**. Chemical shifts (δ) are reported in parts per million (ppm). The molecular structure is shown in the inset.

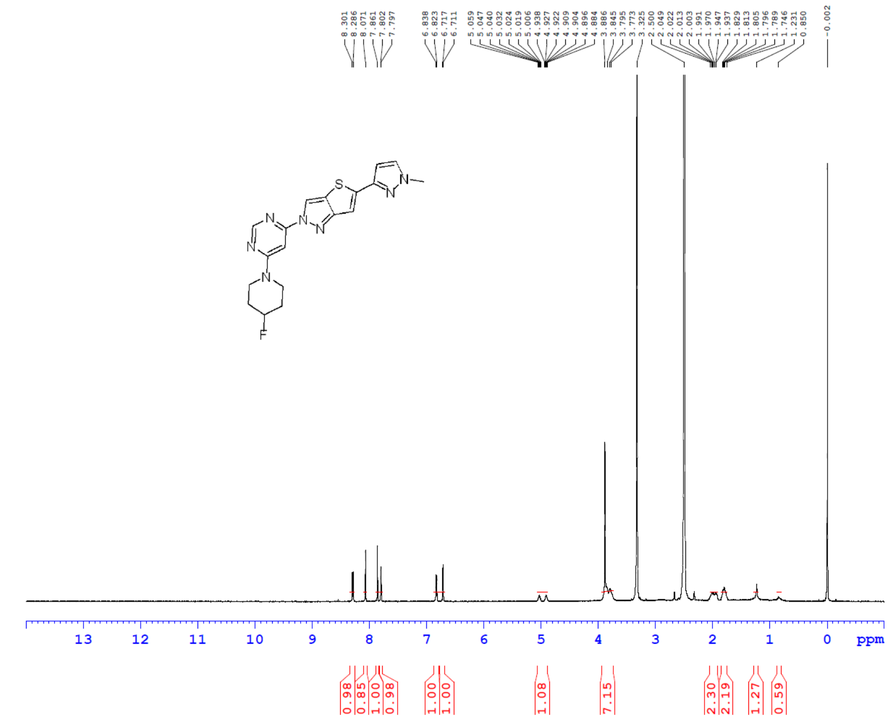

^{1}H NMR spectrum (400 MHz) of **LMD-075**. Chemical shifts (δ) are reported in parts per million (ppm). The molecular structure is shown in the inset.

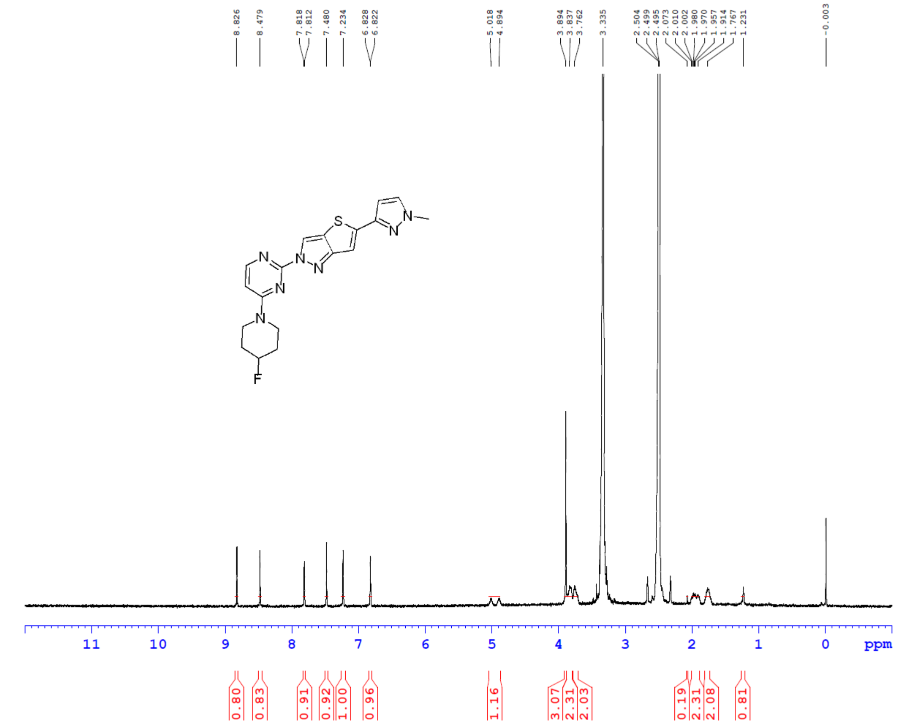

^{1}H NMR spectrum (400 MHz) of **LMD-076**. Chemical shifts (δ) are reported in parts per million (ppm). The molecular structure is shown in the inset.

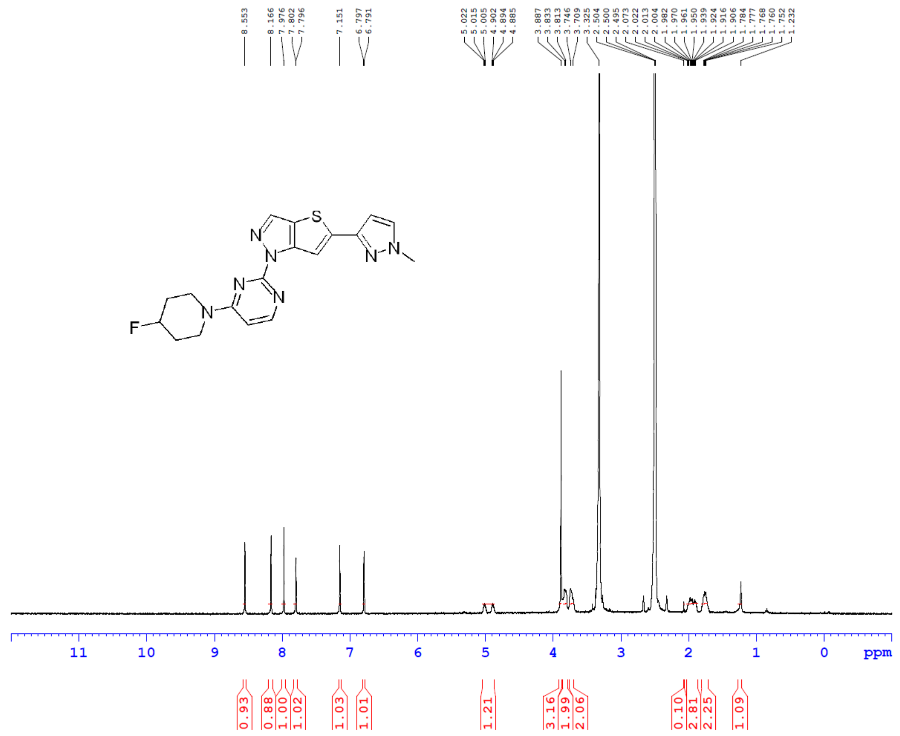

^{1}H NMR spectrum (400 MHz) of **LMD-077**. Chemical shifts (δ) are reported in parts per million (ppm). The molecular structure is shown in the inset.

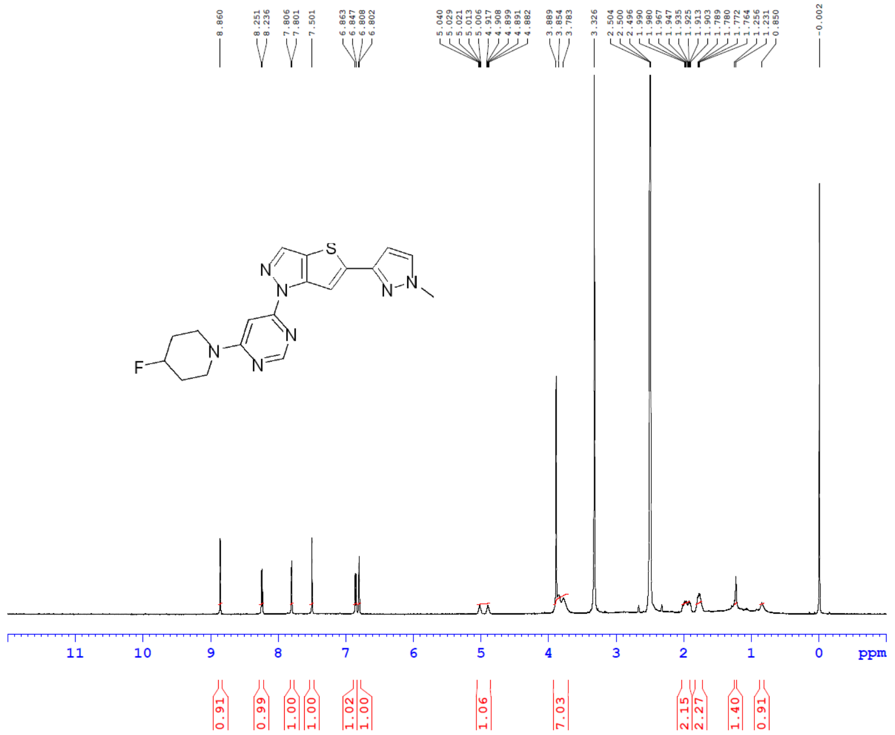

Supplement: Supplementary file 1 [file cells-14-01531-s001.zip › cells-3701356-supplementary.pdf]
